# Human Metabolism of Sirolimus Revisited

**DOI:** 10.3390/metabo15070489

**Published:** 2025-07-20

**Authors:** Baharak Davari, Touraj Shokati, Alexandra M. Ward, Vu Nguyen, Jost Klawitter, Jelena Klawitter, Uwe Christians

**Affiliations:** 1 Department of Pharmaceutical Sciences, Skaggs School of Pharmacy and Pharmaceutical Sciences, University of Colorado Anschutz Medical Campus, Aurora, CO 80045, USA; baharak.davari@cuanschutz.edu (B.D.); alexandra.ward@cuanschutz.edu (A.M.W.); vu.t.nguyen@cuanschutz.edu (V.N.); 2 iC42 Clinical Research and Development, Department of Anesthesiology, School of Medicine, University of Colorado Anschutz Medical Campus, Aurora, CO 80045, USA; touraj.shokati@hepquant.com (T.S.); jost.klawitter@cuanschutz.edu (J.K.); jelena.klawitter@cuanschutz.edu (J.K.)

**Keywords:** sirolimus, human liver microsomes (HLM), metabolite structures, fragmentation patterns, high-resolution time-of-flight mass spectrometry

## Abstract

**Background:** Sirolimus (SRL, rapamycin) is a clinically important mTOR inhibitor used in immunosuppression, oncology, and cardiovascular drug-eluting devices. Despite its long-standing FDA approval, the human metabolic profile of SRL remains incompletely characterized. SRL is primarily metabolized by CYP3A enzymes in the liver and intestine, but the diversity, pharmacokinetics, and biological activity of its metabolites have been poorly explored due to the lack of structurally identified standards. **Methods:** To investigate SRL metabolism, we incubated SRL with pooled human liver microsomes (HLM) and isolated the resulting metabolites. Structural characterization was performed using high-resolution mass spectrometry (HRMS) and ion trap MS^n^. We also applied Density Functional Theory (DFT) calculations to assess the energetic favorability of metabolic transformations and conducted molecular dynamics (MD) simulations to model metabolite interactions within the CYP3A4 active site. **Results:** We identified 21 unique SRL metabolites, classified into five major structural groups: O-demethylated, hydroxylated, didemethylated, di-hydroxylated, and mixed hydroxylated/demethylated derivatives. DFT analyses indicated that certain demethylation and hydroxylation reactions were energetically preferred, correlating with metabolite abundance. MD simulations further validated these findings by demonstrating the favorable orientation and accessibility of key sites within the CYP3A4 binding pocket. **Conclusions:** This study provides a comprehensive structural map of SRL metabolism, offering mechanistic insights into the formation of its metabolites. Our integrated approach of experimental and computational analyses lays the groundwork for future investigations into the pharmacodynamic and toxicodynamic effects of SRL metabolites on the mTOR pathway.

## 1. Introduction

Sirolimus (SRL, rapamycin) was first isolated from *Streptomyces hygroscopicus* [1] and has potent cellular antiproliferative properties [2], including the inhibition of T-cell activation and proliferation, resulting in immunosuppression [3,4,5]. SRL is an inhibitor of the mechanistic target of rapamycin (mTOR) [6,7], which results, among other effects, in the inhibition of cell proliferation, cell growth, and modulation of cell metabolism [8,9,10]. SRL initially received marketing approval from the United States Food and Drug Administration (FDA) in 1999 as an immunosuppressant in renal transplant recipients [11] and, in 2015, for the treatment of lymphangioleiomyomatosis, a rare progressive disease that affects the lungs, kidneys, and the lymphatic system [12]. Moreover, it has been approved as a proliferation inhibitor coated on cardiovascular drug-eluting devices for the treatment of coronary and peripheral artery stenosis [13,14,15]. SRL has also been studied as an immunosuppressant after the transplantation of organs other than the kidney [16], for the treatment of immune diseases [17,18,19,20], and as an anti-cancer drug [21,22,23,24,25,26]. As such, it was approved by the FDA as albumin-bound sirolimus for intravenous injection for the treatment of locally advanced unresectable or metastatic malignant perivascular epithelioid cell tumors in 2021 [27] and as a topical formulation for the treatment of facial angiofibroma associated with tuberous sclerosis complex in 2022 [28]. SRL has a narrow therapeutic index, meaning that small variations in drug concentration can lead to either subtherapeutic effects or toxicity [29,30]. Maintaining optimal dosing is challenging due to interindividual variability in metabolism, drug interactions, and patient-specific factors such as liver function [30,31]. Therapeutic drug monitoring is often required to ensure efficacy while minimizing adverse effects [30,32]. We hypothesize that SRL’s metabolites may contribute to its pharmacological activity [31]. While SRL itself is well studied, its metabolites remain less characterized in terms of their biological effects. If these metabolites exhibit immunosuppressive activity or toxicity, they could influence the drug’s overall efficacy and safety. However, before assessing their impact, it is essential to first identify and characterize these metabolites.

**Figure 1:**
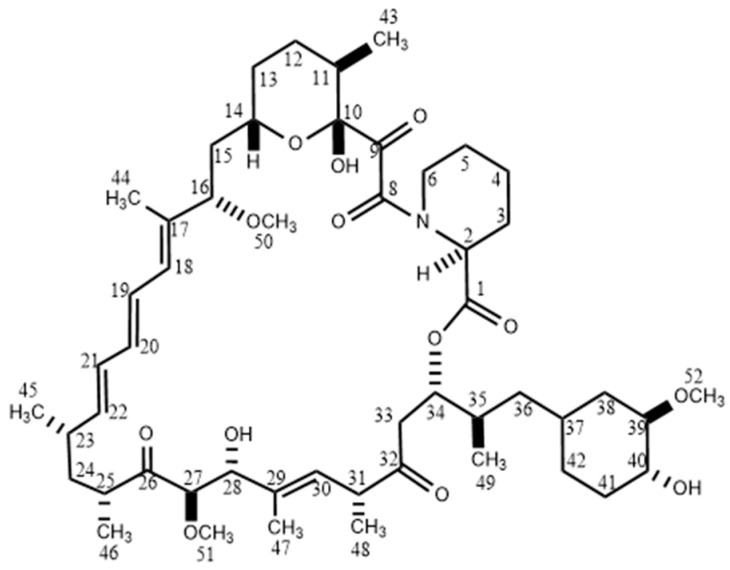
Chemical structure of Sirolimus. Numbering of the molecule follows the IUPAC nomenclature.

SRL (C_51_H_79_NO_13_, molecular weight: 914.18 Da) is a triene macrolide lactone that consists of a 31-membered ring containing four trans-double bonds, three of which are conjugated (Figure 1). SRL is a hydrophobic drug that has low stability in aqueous solutions and is soluble in organic solvents such as methanol, acetonitrile, ether, and halogenated hydrocarbons [4,33], though its stability is increased in a mildly acidic environment.

SRL is a known substrate for cytochrome P450 (CYP450) enzymes and the P-glycoprotein efflux pump [21,34]. It is primarily metabolized by CYP3A4 [23,34], but to a lesser extent it is also metabolized by CYP3A5 [35] and CYP2C8 [36]. At least seven human SRL metabolites have previously been identified [36,37,38]. However, these metabolite structures were identified based on incomplete MS/MS fragmentation patterns and were not appropriately confirmed using high-resolution mass spectrometry. At least in the case of the structurally related mTOR inhibitor everolimus, this led to the publication of incorrect metabolite structures, as discussed by Boernsen et al. [39]. To date, the most complete SRL metabolite pattern generated by human liver microsomes (HLM) was described by Jacobsen et al. [36], albeit in this published meeting abstract, the authors failed to provide any details of how they arrived at the metabolite structures.

The pharmacokinetics, pharmacodynamics, and toxicodynamics of SRL metabolites remain incompletely understood, leaving critical gaps in our knowledge of their clinical relevance. While SRL is widely used, the behavior of its metabolites and their biological activity, potential contributions to efficacy, and role in adverse effects are still largely unknown. Answers to these questions remain elusive, in part due to the inability to purchase SRL metabolites commercially, aside from the single metabolite 16-O-desmethyl sirolimus. SRL metabolites pose several challenges in their generation. First, SRL is a large molecule with many chiral centers, making generating SRL metabolites, even once structurally identified, synthetically a challenging process. Second, many human metabolites are in low concentrations in vivo and in vitro when generated with HLM. Third, SRL metabolites are chromatographically difficult to separate. Fourth, they are unstable in aqueous solutions and pure solvents, as SRL itself is, and quickly degrade into the open-ring seco derivatives, refs. [38,40] making storage of isolated metabolites difficult.

To identify the structures of novel SRL metabolites, the present study employed ion trap mass spectrometry (MS^n^) and high-resolution quadrupole time-of-flight (QTOF) mass spectrometry, coupled with in-depth fragmentation pattern analysis of SRL to compare its pattern to those of the metabolites. This same approach has successfully elucidated the human metabolism of various SRL derivatives—such as everolimus, myolimus (SAR943), zotarolimus, and temsirolimus [39,41,42,43].

In some specific cases, the fragmentation pattern analysis would not be sufficient to fully elucidate the absolute metabolite structure, but it could narrow down the site of modification to two carbon positions. In these cases, quantum mechanical/molecular mechanics (QM/MM) can be a powerful tool to determine the probabilistic site of metabolism. SRL, which contains multiple potential hydroxylation sites, benefits significantly from these methods. By analyzing conformational changes and key interactions (e.g., hydrogen bonding and π–π stacking), we could further pinpoint the atoms closest to CYP3A4’s catalytic center, indicating an increased likelihood of oxidation events. Specifically, Density Functional Theory (DFT) was used to elucidate electronic structures and molecular dynamics (MD) were employed to examine binding orientations, affinities, and sites of metabolism (SOM) prediction.

## 2. Materials and Methods

### 2.1. Chemicals, Reagents, and Enzymes

Unless mentioned otherwise, the solvents and reagents used in this study were purchased from Fisher Scientific (Fair Lawn, NJ, USA) and, whenever available, solvents were of a high-performance liquid chromatography (HPLC) grade. SRL was purchased from LC Laboratories (Woburn, MA, USA). Pooled HLMs from 200 different individuals were from Xenotech (Kansas City, KS, USA). The activities of each individual CYP enzyme and the protein concentration of the HLMs had been characterized by the manufacturer. NADP and other constituents of the NADPH-generating system (see below) were purchased from Sigma Aldrich (St Louis, MO, USA). SRL stock solutions (1 mg/mL) were prepared in 0.1% formic acid/acetonitrile (1/4, *v*/*v*). All of the stock solutions and isolated metabolite fractions were stored at −70 °C or below until further analysis.

### 2.2. Formation Kinetics of SRL Metabolites

Apparent enzyme kinetic parameters for the major SRL metabolites were assessed. The NADPH-generating system contained 5 mM of EDTA, 25 mM of MgCl_2_, 45 mM of isocitric acid, Na^+^/K^+^ phosphate buffer (0.1 M, pH 7.4), and 1.75 units/mL of isocitrate dehydrogenase. Before the addition of the SRL, the NADPH-generating system was preincubated at 37 °C (type C24 incubator shaker, New Brunswick Scientific, Edison, NJ, USA) for 5 min. Incubation time (0, 10, 15, 20, 30, 45, 60, 90, and 120 min), HLM protein concentration (0.025, 0.05, 0.1, 0.2, 0.4, and 0.8 mg/mL), and SRL concentration (3.25, 7.5, 15, 30, 60, 90, and 120 µM) were optimized; all parameters were tested in quadruplicate. The final volume of the reaction solution was 500 μL, and the reaction was quenched by the addition of a 1:1 volume of ice-cold acetonitrile. Following protein precipitation, samples were briefly vortexed and subsequently centrifuged at 4 °C and 13,000× *g* for 10 min. The supernatant was transferred to a 2-mL HPLC glass vial and analyzed using high-performance liquid chromatography/tandem mass spectrometry (LC-MS/MS). Representative control incubations without HLMs, without drug, or without the NADPH-generating system (all in quadruplicate) were carried out under the same conditions with every SRL HLM metabolism batch. Representative extracted ion chromatograms of the control samples are shown on page 13 of the Supplementary Materials.

### 2.3. Large-Scale Preparation of SRL Metabolites Using Pooled HLMs

For the identification of major and minor SRL metabolites as well as isolation of the necessary quantities for fragmentation-based structural analysis, the generation of SRL metabolites using HLMs was scaled up. The optimized incubation conditions included 30 µM of SRL, 0.4 mg/mL of HLM, and one-hour incubation at 37 °C, which resulted in the highest yield of most metabolites. The final reaction volume was 100 mL. The reaction was quenched by adding an equal volume of ice-cold acetonitrile, followed by centrifugation at 4 °C and 13,000× *g* for 10 min. The supernatant was then transferred to a separatory funnel and extracted with dichloromethane. The organic phase was separated, evaporated under a stream of nitrogen at 37 °C, and reconstituted in 1.0 mL of 0.1% formic acid/acetonitrile (1/4, *v*/*v*).

### 2.4. Separation, Isolation, and Mass Spectrometry Detection of SRL and Its Metabolites

Metabolite separation and isolation were carried out using a semi-preparative HPLC system consisting of the following series 1200 HPLC components: an HPLC autosampler (G1365B) combined with a binary pump (G1361A), a fraction collector (G1364B), diode array detector (G1315B) (all Agilent Technologies, Palo Alto, CA, USA), and a column compartment (TS-430, Phenomenex, Torrance, CA, USA). One hundred µL of the HLM-generated metabolite extract was injected into the HPLC system. Metabolites were separated using four Zorbax Eclipse XDB-C8 semi-preparative columns (9.4 mm× 250 mm, 5 µm) connected in series. The HPLC flow rate was 3.5 mL/min, and the mobile phases consisted of water (mobile phase A) and acetonitrile (mobile phase B). The following gradient was used; 0–2 min 55% mobile phase B, 2.1–69 min 60% mobile phase B, 69.1–75 min 73% mobile phase B, 75.1–82 min 95% mobile phase B, 82.1–83 min 55% mobile phase B, and 83.1–90 min 55% mobile phase B. The total run was 90 min. The column compartment temperature was set to 65 °C. Twenty-seven fractions were collected automatically during each run using the fraction collector. Corresponding fractions were pooled, and each individual pooled fraction extracted using dichloromethane. The organic layer was isolated, and the fractions were dried under a flow of nitrogen at 37 °C. The isolated fractions were reconstituted in water with 0.1% formic acid/acetonitrile (1/4, *v*/*v*). If there were any impurities in the isolated fractions, a repurification step was performed on the same semi-preparative HPLC system.

A total of 10 µL of the metabolite extract was injected onto a series of three 250 mm × 4.6 mm analytical columns (Zorbax Eclipse XDB-C8, 5 µm particle size, Agilent Technologies, Palo Alto, CA, USA). The columns were maintained at a temperature of 65 °C. The HPLC flow rate was 1 mL/min, and the mobile phases consisted of water + 0.1% formic acid (mobile phase A) and acetonitrile + 0.1% formic acid (mobile phase B). The gradient elution program was set up as follows: 0–3 min 45% mobile phase B, 3.1–10 min 60% mobile phase B, 10.1–55 min 65% mobile phase B, 55.1–57 min 98% mobile phase B, 57–70 min 98% mobile phase B, 70.1–72 min 45% mobile phase B, and 72.1–80 min 45% mobile phase B. The total run time was 80 min. Structural identification of SRL metabolites was performed in positive electrospray ionization (ESI) mode on a 5600+ high-resolution QTOF mass spectrometer (“Triple-TOF”, Sciex, Concord, ON, Canada).

The fragmentation patterns of SRL and its metabolites for structural identification were evaluated after direct infusion into the QTOF mass spectrometer. The optimized QTOF operating parameters were as follows: ion spray voltage, 5500 kV; ion source heater, 600 °C; declustering potential, 40 eV; collision energy, 60 eV; collision energy spread, 15 eV; curtain gas, 35 psi; ion source gas 1, 55 psi; and ion source gas 2, 55 psi. The information-dependent acquisition (IDA) method consisted of a TOF-MS survey scan (*m*/*z* = 100–1000) followed by TOF-MS/MS scans (*m*/*z* 50–1000) with an accumulation time of 0.5 s and 1 s, respectively.

### 2.5. Density Functional Theory Calculations

Previous literature has not explored the role of hydrogen abstraction on the double-bond conjugated system of SRL. This aspect of SRL metabolism has remained largely unexamined, despite its potential implications for metabolite stability, reactivity, and biological activity. By addressing this gap, our study provides a novel perspective on SRL’s metabolic pathways, offering deeper insights into its structural transformations and their possible functional consequences.

Density Functional Theory (DFT) calculations were conducted on SRL and its metabolites, including radicals generated from demethylation and hydrogen abstraction, as well as products formed through hydrogenation and hydroxylation, to determine their ground-state energies [44]. The ground-state energy values were summed to predict the ΔG values for demethylation, hydrogen abstraction, hydrogenation, and hydroxylation processes. These calculations were carried out using the Jaguar module within the Schrödinger Suite (Release 2024-2: Jaguar, Schrödinger, New York, NY, USA) [45]. DFT calculations were used for optimization of all metabolite structures and resulting ground-state energy calculations through UB3LYP/6-31G* in gas phase [46,47,48,49,50]. In the case of the radicals, the |S^2^| was monitored for any degree of spin contamination with the open-shell unrestricted formulation.

### 2.6. Molecular Dynamic Simulations

Molecular Dynamic (MD) simulations were carried out with the Desmond Module within the same Schrödinger Suite. The MD simulations were performed using the published CYP3A4 crystal structure (2V0M) with an added SRL ligand in the active site [51]. Atomic partial charges and initial starting geometry for SRL were obtained through DFT calculations using HF/6-31G*. OPLS4 force fields were used as implemented by Desmond, and atomic charges for the CYP3A4 heme unit and the axial cysteine residue were also obtained through the DFT calculations mentioned above. The cutoff for non-bonded interactions were set to 15 Å and the time step was set to Langevin dynamics with an integration timestep of 1.0 fs.

The calculations were separated into two sections: one examining demethylation and the other hydrogen abstraction through relative positioning of the SRL in distance from the heme iron atom to determine the likelihood of the chemical event occurring. For the demethylation, there were three groups where the methyl group was placed within 3–5 Å of the heme’s Fe of SRL within the active site of CYP3A4. The three groups referred to Group 1 where C(27) was closest to the heme Fe, Group 2 was C(16), and Group 3 was C(39). For the hydrogen abstraction, there were also three distinctions of orientation of SRL in the pocket as defined by the relative closeness in placement of groups of SRL carbons. Group 4 was C(23)–C(25), C(45), and C(46) closest, Group 5 was C(18)–C(22) closest, and Group 6 was C(11), C(12), and C(15) closest. The system was built with TIP3P water solvation.

The SRL-CYP3A4 complex structure was relaxed to an energetic minimum. The ligand binding energy through MMGBSA energy was calculated for said evaluation to be between SRL and CYP3A4. From there, simulated annealing was done to further relax the system to a lower energetic point, if it was not already there. This was done over 12 ps, during which time the temperature was raised from 0 to 300 K at 3ps, then reduced back to 0K at the end of the time frame. Constraints were applied during this process, using a harmonic oscillator of the heme Fe to the methoxy O atoms of 200 kcal/mol angstrom squared as well as the same harmonic oscillator for all trans-SRL bonds. The resulting ligand binding energy was taken for comparison to the previous value. Afterwards, the system was equilibrated for 50 ps, with a harmonic oscillator between the methoxy O and heme Fe, but at 50 kcal/mol A2 this time, and was checked for equilibration afterwards through convergence and RSMD measurements. The resulting system ran for 5 ns at 300 K.

The distance and converted RDF plots were obtained and presented in both demethylation and abstraction cases to present the predicted distance between the atoms of interest to display the chance of the chemical event, as the Fe atom would perform either reaction.

### 2.7. Statistics and Data Analysis

For data analysis, we utilized Sciex Analyst Software (version TF1.7.1) to process and interpret mass spectrometry data. Structural analysis and the assessment of calculated mass errors were conducted using a combination of ChemDraw (version 19.0, PerkinElmer, Waltham, MA, USA) for molecular visualization and structural elucidation and Excel (version 365, Microsoft, Redmond, WA, USA) for quantitative analysis and data organization. Kinetics data was fitted using the GraphPad Prism Enzyme Kinetics Module (Version 10.0.2, for Windows, GraphPad Software, Boston, MA, USA). 

## 3. Results

### 3.1. HPLC-MS of SRL

SRL and its metabolites were detected in the positive ionization mode. The sodium adduct [M+Na]^+^ ions displayed the highest signal intensities under the selected conditions. Other detected minor ions were [M+NH_4_]^+^ and [M+K]^+^ (please see the Supplementary Materials, page 8). In general, the formation of adducts could be modified. The modifications depend on various parameters, including HPLC and electrospray ionization conditions. The second most abundant adduct ion was [M+K]^+^, which could be suppressed by optimizing the cone voltage and increasing the ion source temperature. Moreover, compared to [M+Na]^+^, neither the potassium nor the ammonium adducts yielded the detailed fragmentation patterns necessary for metabolite identification due to their low intensities. Hence, structural analysis was based on the sodium adducts, which were most intense and stable under the selected electrospray ionization-MS/MS conditions. Following ionization, the loss of 18 amu (H_2_O) was a common fragmentation result, which is rarely informative for structural identification and therefore is not further discussed in the present study, except for 23/24-hydroxy SRL (*vide infra*).

**Figure 2:**
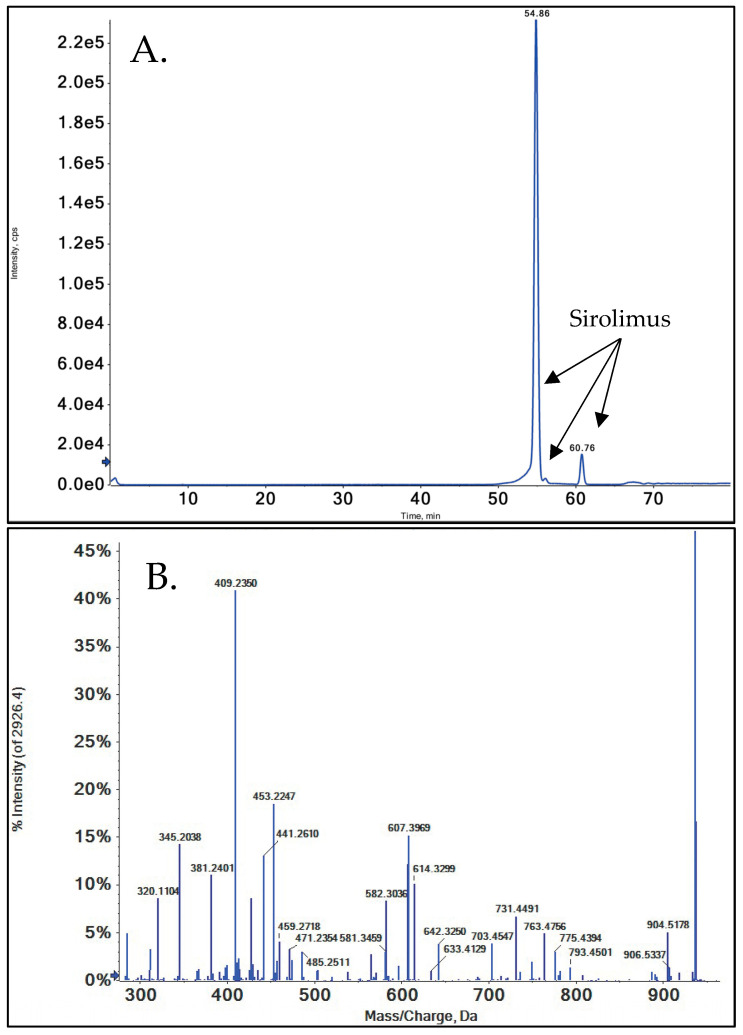
Representative high-resolution (**A**) total ion chromatogram (TIC) and (**B**) MS/MS spectrum of sirolimus (*m*/*z* = 936.5444).

A representative collision-induced dissociation spectrum used for structural identification recorded using QTOF mass spectrometry is shown in (Figure 2). The twenty-one sirolimus metabolites that were identified in the present study are listed in (Table 1).

**Figure 3:**
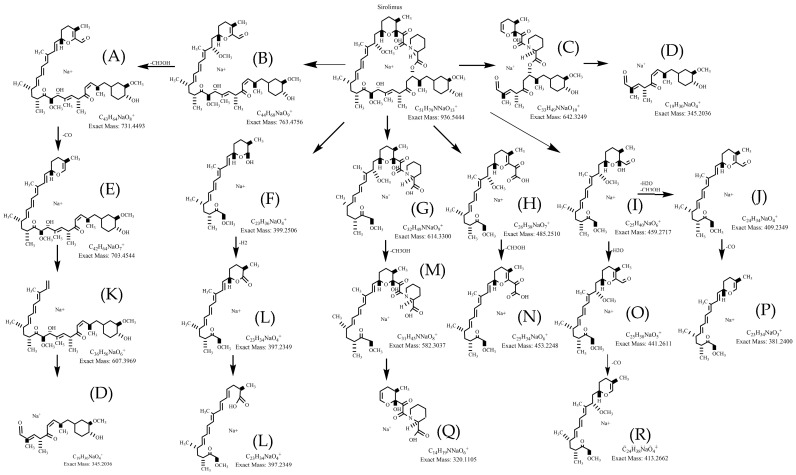
Proposed fragmentation pattern of sirolimus. Major fragmentation pathways of sirolimus (A–R, fragments described in detail in Table 2). Only the key fragments used for the identification of the metabolite structures are shown. The fragmentation pathways were assessed using ion trap MS^n^.

The fragmentation pattern of SRL was systematically evaluated, and it was determined that all of the major fragments were the result of α-cleavage, aligning with previous reports [39,41,42]. In a first step, the relationship of the different fragments detectable in MS/MS spectra was interrogated using MS^n^ experiments. Based on said relationships, fragment structures and pathways were proposed (Figure 3). The theoretical exact mass of the proposed fragment structures was then compared with the measured exact mass obtained by high-resolution QTOF. A difference of less than 5.0 ppm between the measured and theoretical exact mass of a hypothetical fragment structure was considered confirmatory [38]. In most cases, the Δppm was less than 4.0 in the present study (Table 2).

QTOF MS spectra, proposed fragmentation patterns, fragment structures, mass accuracy calculations, and in-depth structural analyses for SRL and all of its detected and isolated metabolites generated by HLM in vitro are shown in the Supplementary Materials. As representative examples, the main metabolites are discussed below.

### 3.2. O-Demethylation

**Figure 4:**
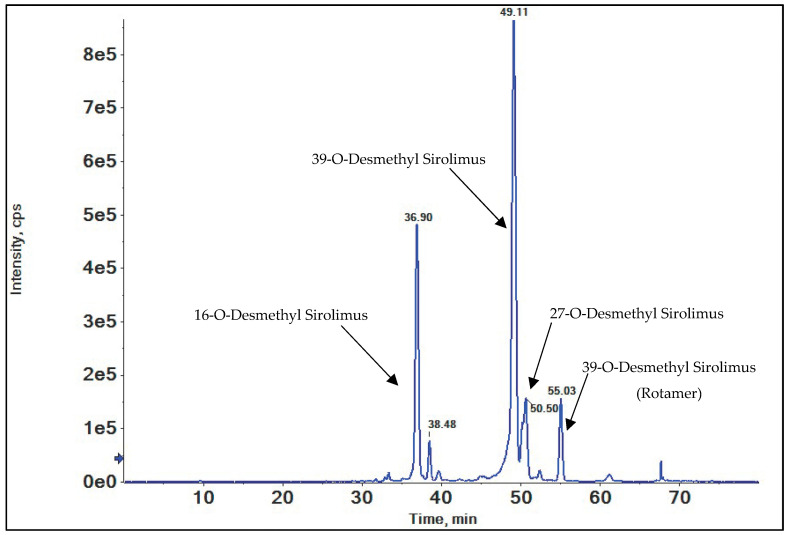
Representative extracted ion chromatogram (EIC) of demethylated sirolimus metabolites (*m*/*z* = 922.5287).

Based on prior studies of SRL and structurally related compounds, O-demethylation of the methoxy groups was anticipated [41,42,52]. SRL has three potential sites for O-demethylation: C(16), C(27), and C(39). The extracted ion chromatograms (EIC) for the *m*/*z* = 922.5287 ([M+Na]^+^) of demethylated SRL indeed displayed distinct chromatographic peaks corresponding to demethylation at these three positions (Figure 4).

#### 3.2.1. 16-O-Desmethyl SRL

**Figure 5:**
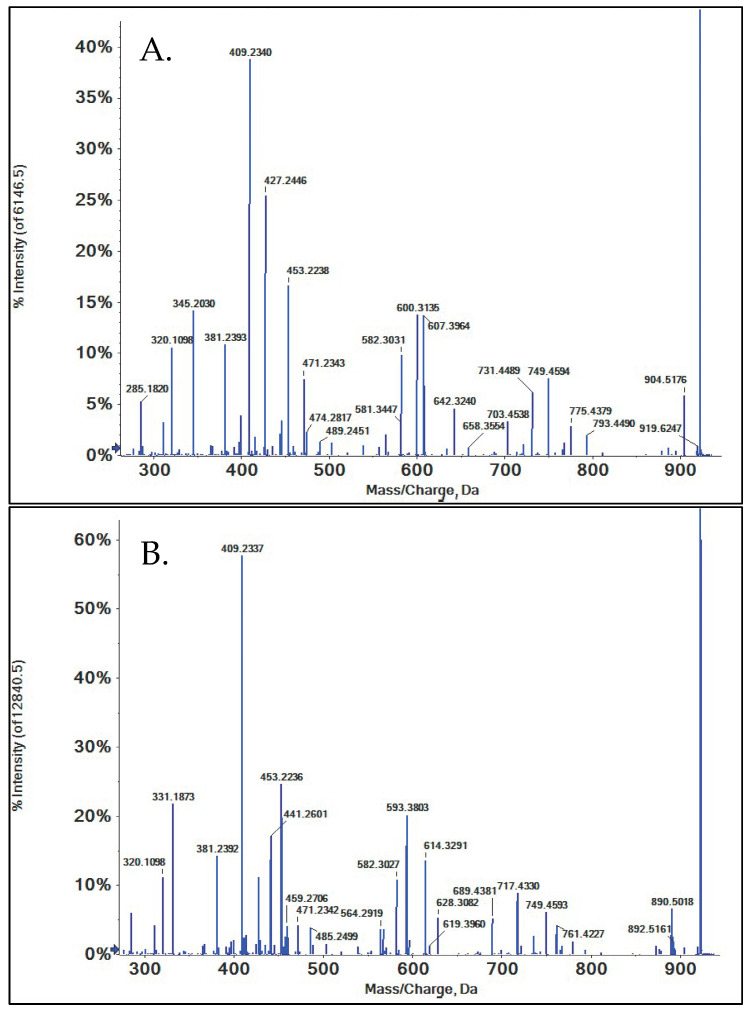

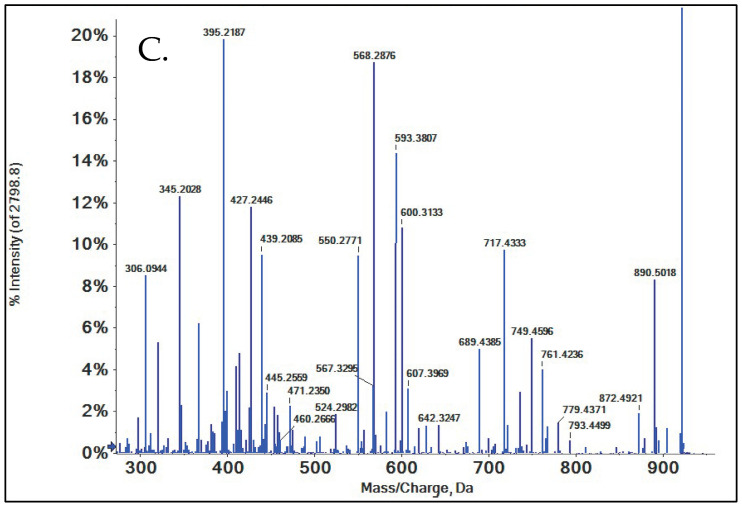
Representative mass spectra of the demethylated sirolimus metabolites (*m*/*z* = 922.5287). (**A**) 16-O-desmethyl Sirolimus, (**B**) 39-O-desmethyl Sirolimus, (**C**) 27-O-desmethyl Sirolimus.

The compound eluting at a retention time of 36.90 min in the extracted ion chromatogram (EIC) (Figure 4) exhibited a molecular ion [M+Na]^+^ of *m*/*z* = 922.5287, indicating the loss of a methyl group from SRL ([M+Na]^+^ *m*/*z* = 936.5444) (Figure 4). The high-resolution mass spectrum of this metabolite is displayed in (Figure 5A). A comparison of the MS/MS spectra of this metabolite with those of SRL confirmed 16-O-demethylation. Specifically, the metabolite displayed fragments at *m*/*z* = 607.3969 and *m*/*z* = 311.1982 which are the characteristic fragments of 16-O-desmethyl SRL. The presence of *m*/*z* = 607.3969, excludes demethylation at both C(27) and C(39). Additionally, fragments at *m*/*z* = 345.2036, *m*/*z* = 381.2400, and *m*/*z* = 409.2349 matched those of SRL, further excluding demethylation at position C(39). The calculated mass error (Δppm) for the observed key fragments was consistently below 3 ppm, confirming the structure of the metabolite as 16-O-desmethyl SRL. For further details, please refer to the Supplementary Materials, pages 17–23.

#### 3.2.2. 39-O-Desmethyl SRL

The metabolite eluting at a retention time of 49.11 min in the EIC exhibited a molecular ion [M+Na]^+^ of *m*/*z* = 922.5287, consistent with the loss of a methyl group (Figure 4). This metabolite was identified as 39-O-desmethyl SRL (please refer to Supplementary Materials, pages 24–30). It displayed the same fragmentation pattern as SRL, except for fragments at *m*/*z* = 628.3092 and *m*/*z* = 331.1880, which confirmed O-demethylation at position C(39). Another peak, eluting at a retention time of 55.03 min, was identified as a rotamer of 39-O-desmethyl SRL. This rotamer exhibited an identical fragmentation pattern to the metabolite at 49.11 min, including the characteristic fragments at *m*/*z* = 628.3092 and *m*/*z* = 331.1880. The high-resolution mass spectrum of this metabolite is presented in (Figure 5B).

#### 3.2.3. 27-O-Desmethyl SRL

The MS spectrum of the metabolite peak eluting at a retention time of 50.50 min (Figure 4) displayed a molecular ion [M+Na]^+^ of *m*/*z* = 922.5887, indicating the loss of a methyl group. The QTOF mass spectrum of this metabolite is presented in (Figure 5C). The presence of fragments at *m*/*z* = 642.3249 and *m*/*z* = 345.2036 excluded demethylation at position C(39). A comparison of the MS/MS spectra of SRL and the metabolite revealed that the SRL fragment at *m*/*z* = 607.3969 corresponded to a metabolite fragment at *m*/*z* = 593.3813, confirming the metabolite as 27-O-desmethyl SRL. For additional details, refer to Supplementary Materials, pages 31–36.

### 3.3. Hydroxylation

**Figure 6:**
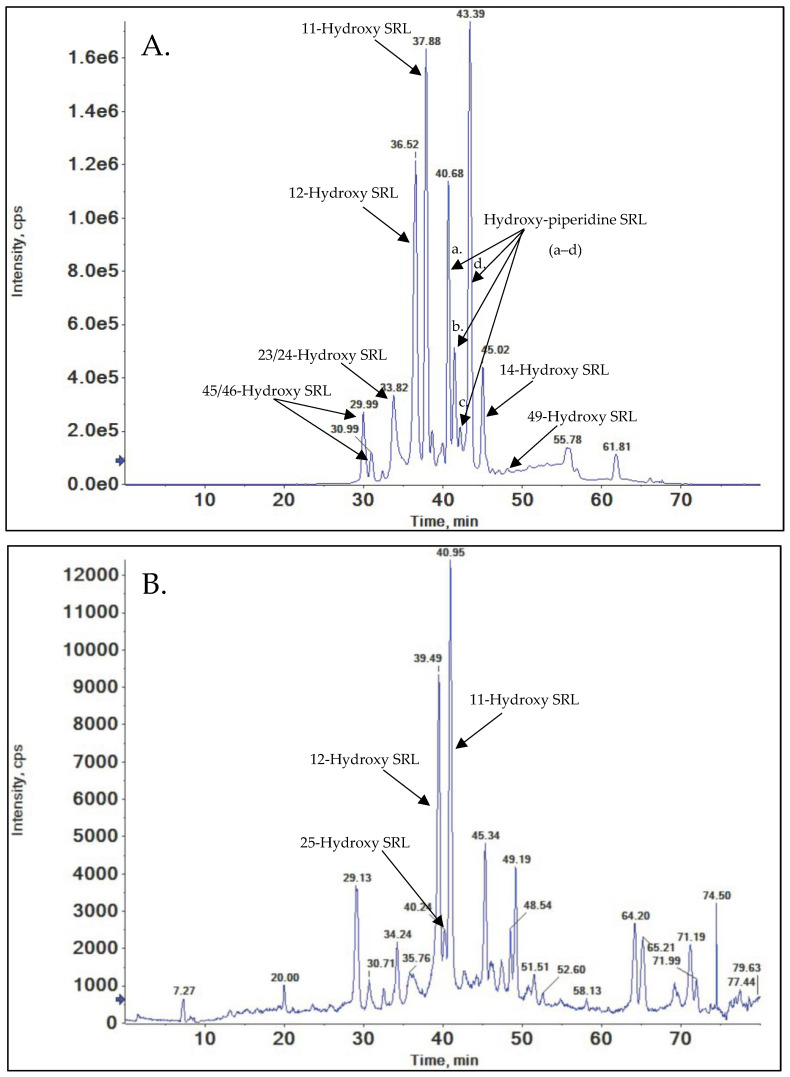
(**A**) Representative Extracted Ion chromatogram (EIC) of hydroxylated sirolimus metabolites (*m*/*z* = 952.5393). (**B**) Representative Extracted Ion chromatogram (EIC) of hydroxylated sirolimus metabolites (*m*/*z* = 952.5393) to further separate 25-hydroxy Sirolimus.

Previous studies have identified the major sites of hydroxylation in SRL as C(3, 4, 5 and 6) on the piperidine ring and C(11, 12, 14, 23, 24, 25, 45, 46, and 49) [36,37,38,39]. The extracted ion chromatogram (EIC) for *m*/*z* = 952.5393 indicated hydroxylation at a single position on SRL (Figure 6A,B). The probability of forming 23/24-hydroxy SRL and 45/46-hydroxy SRL, as well as the precise prediction of the site of metabolism, were investigated using quantum chemical calculations and molecular dynamic techniques. These findings will be discussed in greater detail in Section 3.10.

#### 3.3.1. 45/46-Hydroxy SRL

The first two peaks in the EIC for hydroxylated metabolites, with retention times of 29.99 and 30.99 min, were identified as 45/46-hydroxy SRL (Figure 6A). Compared to SRL fragments, the presence of *m*/*z* = 623.3918 (+16 Da relative to the corresponding SRL fragment at *m*/*z* = 607.3969) suggests hydroxylation at carbon positions 23, 24, 45, 46, or 49. The fragment at *m*/*z* = 345.2036 excluded 49-hydroxy SRL. Additionally, most fragments containing C(23) and C(26) displayed a −32 Da difference, indicating the loss of methanol, a characteristic feature of SRL fragments with methyl group hydroxylation (see Supplementary Materials, pages 55–61). This evidence supports hydroxylation at either C(45) or C(46). However, the MS/MS and MS^n^ fragmentation patterns were insufficient to distinguish between C(45) and C(46) as the hydroxylation site. It is also plausible that the peak at 30.99 min represents a mixture of both metabolites that could not be chromatographically separated.

#### 3.3.2. 23/24-Hydroxy SRL

The metabolite detected at 33.82 min (Figure 6A) exhibited fragments that included C(23) and C(24), with an *m*/*z* 16 Da higher than the corresponding SRL fragments (e.g., *m*/*z* = 630.3249 compared to SRL at *m*/*z* = 614.3300, and *m*/*z* = 457.2561 compared to SRL at *m*/*z* = 441.2611). A characteristic feature of this metabolite was that hydroxylated fragments showed corresponding fragments after a water loss with higher intensity than the fragments without water loss (e.g., *m*/*z* = 630.3249 and *m*/*z* = 612.3144; *m*/*z* = 598.2986 and *m*/*z* = 580.2875; *m*/*z* = 457.2556 and *m*/*z* = 439.2449). For further details, refer to the Supplementary Materials, pages 62–70. The MS/MS and MS^n^ fragmentation patterns were unable to distinguish between C(23) and C(24) as the hydroxylation site. However, the broad and split peak at a retention time of 33.82 min suggested that it may be caused by more than one metabolite.

#### 3.3.3. 12-Hydroxy-SRL

The compound eluting at 36.52 min in the EIC (Figure 6A) with an observed molecular ion of [M+Na]^+^ at *m*/*z* = 952.5393 exhibited an *m*/*z* 16 Da higher than SRL, consistent with hydroxylation at a single position. The MS/MS spectrum revealed characteristic fragment ions at *m*/*z* = 711.4442, *m*/*z* = 389.2293, and *m*/*z* = 357.2031, which are specific to 12-hydroxy SRL (see Supplementary Materials, pages 71–77). These fragments were previously reported by Streit et al. [38] and attributed to the additional hydroxy group facilitating α-cleavage of adjacent C-C bonds. High-resolution mass spectrometry further confirmed the structures of these characteristic fragments, with calculated mass errors between the measured and theoretical exact masses consistently ≤ 1.7 Δppm.

#### 3.3.4. 25-Hydroxy-SRL

This metabolite co-eluted with 12-hydroxy SRL, necessitating a more isocratic approach for improved separation. The method was then adjusted by slightly modifying the gradient, allowing 25-hydroxy SRL to elute as a distinct peak and enabling its structural identification. The metabolite eluting at 40.24 min in the EIC (Figure 6B) displayed a molecular ion [M+Na]^+^ at *m*/*z* = 952.5393, 16 Da higher than SRL, consistent with single-site hydroxylation. Diagnostic fragments for C(25) hydroxylation were observed at *m*/*z* = 443.2392 and *m*/*z* = 447.2718 (see Supplementary Materials, pages 78–85). Characteristic fragments, including *m*/*z* = 327.1922 and *m*/*z* = 447.2718, arose from α-cleavage adjacent to C(25), mirroring fragmentation patterns reported for 25-hydroxy metabolites of SAR943 and zotarolimus [41,42]. High-resolution mass spectrometry confirmed these structures, with Δppm < 5 between the measured and theoretical exact masses.

#### 3.3.5. 11-Hydroxy-SRL

The metabolite fraction collected at 37.88 min (Figure 6A) was characterized by a molecular ion [M+Na]^+^ at *m*/*z* = 952.5393, indicating a single hydroxylation event. All of the fragments containing the C(11) moiety appeared at *m*/*z* values +16 Da above their corresponding SRL fragments, confirming hydroxylation at C(11). Key diagnostic fragments included *m*/*z* = 327.1927, *m*/*z* = 357.2036, and *m*/*z* = 723.4806; the latter arose from α-cleavage of C–C bonds due to 11-hydroxylation and lacked a corresponding SRL fragment (see Supplementary Materials, pages 86–92). Notably, this characteristic fragment identifying hydroxylation at C(11) was also described for the structurally related zotarolimus [42]. The high-resolution mass spectrometry data supported this assignment, with a Δppm of 0.1 between the theoretical and measured exact mass for the *m*/*z* = 723.4806 fragment.

#### 3.3.6. Hydroxy-Piperidine SRL

The piperidine ring offers four unique positions for hydroxylation (C(3), C(4), C(5), and C(6)). However, pinpointing the exact site of hydroxylation was not possible due to the absence of distinct MS/MS and MS^n^ fragmentation signatures from the piperidine moiety, even post-hydroxylation. This phenomenon has also been noted in metabolites of other SRL derivatives hydroxylated on the piperidine ring [41,42,52].

In the present work, SRL exhibited four separate peaks corresponding to hydroxy-piperidine metabolites at retention times of 40.68 (a), 41.45 (b), 42.17 (c), and 43.39 (d) minutes ([M+Na]^+^ *m*/*z* = 952.5393) (Figure 6A). The key diagnostic fragment indicating hydroxylation on the piperidine ring is *m*/*z* = 336.1050 (see Supplementary Materials, pages 93–101), which is 16 Da higher than the corresponding SRL fragment at *m*/*z* = 320.1105.

#### 3.3.7. 14-Hydroxy SRL

The metabolite eluting at 45.02 min had an [M+Na]^+^ ion at *m*/*z* = 952.5393 (Figure 6A), indicating single-site hydroxylation. All of the fragments encompassing the C(11)–C(14) region showed a +16 Da shift relative to the corresponding SRL fragments, suggesting hydroxylation in this segment (see Supplementary Materials, pages 102–108). A notable fragment at *m*/*z* = 612.3133, which lacks an equivalent SRL fragment, arises from the α-cleavage of C–C bonds driven by hydroxylation at C(14), a finding also recently reported for zotarolimus [42]. High-resolution mass spectrometry (Δppm = 1.6) corroborated the identity of this fragment. Furthermore, the presence of fragments at *m*/*z* = 607.3969 and *m*/*z* = 612.3133 rules out hydroxylation at other positions, firmly establishing this metabolite as 14-hydroxy SRL.

#### 3.3.8. 49-Hydroxy SRL

This metabolite, detected at 48.11 min (Figure 6A), exhibited an [M+Na]^+^ ion at *m*/*z* = 952.5393, consistent with a single hydroxylation. Fragments that included the C(49) moiety were shifted by +16 Da relative to the corresponding SRL fragments (see Supplementary Materials, pages 109–115). The presence of *m*/*z* = 580.2869, which matched a major SRL fragment, effectively excluded most other hydroxylation sites. A unique fragment at *m*/*z* = 361.1978 pinpointed C(49) as the most likely hydroxylation position, supported by a Δppm of 3.0 between the measured and theoretical masses. A similar characteristic fragment was also reported for 49-hydroxy zotarolimus [42].

### 3.4. Second-Generation Metabolites

All of the above-described metabolites are modified in one position and are considered first generation SRL metabolites. Under the incubation conditions used in the present study, as the parent drug is available at high concentrations, it initially competes with the generated metabolites for the cytochrome P450 enzymes and prevents their further metabolism. With longer incubation times, however, the concentrations of first generation metabolites increase and are more likely to interact with cytochrome P450 enzymes, thus forming second-generation metabolites such as didesmethyl, desmethyl hydroxy, and dihydroxy metabolites, all of which were detected. In the present study, several structures of the highest abundance second-generation metabolites could be identified.

### 3.5. Di-Desmethylated Metabolites of SRL

These metabolites had an [M+Na]^+^ with *m*/*z* = 908.5131, indicating further demethylation of demethylated first generation metabolites.

#### 3.5.1. 16, 39-O-Didesmethyl SRL

**Figure 7:**
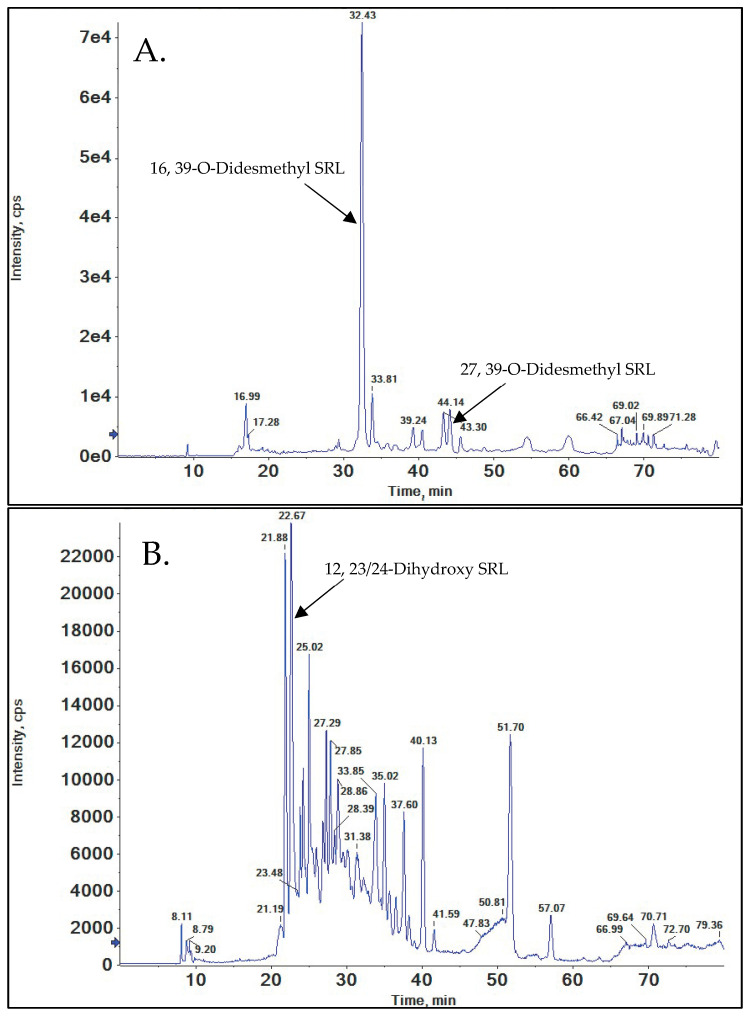

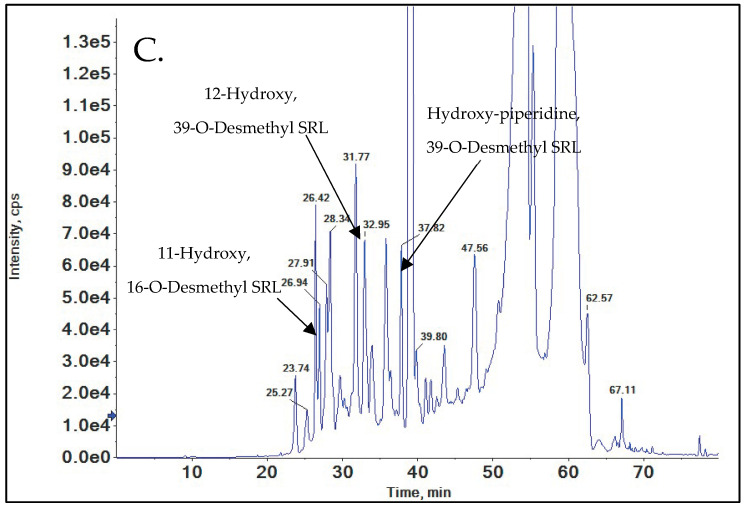
(**A**) Representative total ion chromatogram (TIC) of didesmethylated sirolimus metabolites, (**B**) di-hydroxylated sirolimus metabolites, and (**C**) hydroxy-demethylated sirolimus metabolites.

The peak eluting at 32.43 min (Figure 7A) exhibited the fragments characteristic of the demethylation of SRL at both positions C(16) and C(39) (please see Supplementary Materials, pages 40–45).

#### 3.5.2. 27, 39-O-Didesmethyl SRL

The metabolite detected at 44.14 min (Figure 7A), shows the characteristic fragments for both 27- and 39-O-demethylation of SRL. The characteristic fragments for 16-O-desmethylation match the corresponding SRL fragments. The specific fragments for 27, 39-O-didesmethyl SRL are discussed in the Supplementary Materials, pages 46–51.

### 3.6. Di-Hydroxylated Metabolites of SRL

The metabolite peaks with *m*/*z* = 968.5342 corresponds to dihydroxy SRL metabolites. We identified 12, 23/24-dihydroxy SRL based on the QTOF MS/MS spectra.

#### 12, 23/24-Dihydroxy SRL

The MS/MS spectrum showed a peak eluting at 22.67 min (Figure 7B). The proposed fragmentation pattern and the high-resolution mass spectra suggested that the metabolite underlying this peak is 12, 23/24 dihydroxy SRL. The MS/MS spectra show the corresponding characteristic fragments of 12-hydroxy SRL (*m*/*z* = 711.4442 and 389.2293), albeit with +16 Da due to the additional hydroxylation (*m*/*z =* 727.4390 and *m*/*z =* 405.2237). Further analysis of the fragmentation pattern of this metabolite pointed toward hydroxylation at C(23) or C(24) as the second hydroxylation position. For further details, please see the Supplementary Materials, pages 116–122.

### 3.7. Hydroxy, O-Desmethyl Metabolites of SRL

Metabolite peaks with *m*/*z =* 938.5236 (Figure 7C) corresponded to hydroxy-desmethyl metabolites of SRL.

#### 3.7.1. Hydroxy-Piperidine, 39-O-Desmethyl SRL

The QTOF MS/MS spectra of the metabolite eluting with a retention time of 37.82 min showed both the characteristic fragments of 39-O-desmethyl SRL with *m*/*z* = 331.1880 and of hydroxy-piperidine SRL with *m*/*z* = 336.1053. All other fragments were also consistent with hydroxylation at the piperidine moiety and O-demethylation at C(39). For more details, please see Supplementary Materials, pages 123–128.

#### 3.7.2. 12-Hydroxy, 39-O-Desmethyl SRL

The SRL metabolite eluting at 32.95 min showed fragments of *m*/*z* = 331.1879 and 642.2 in the QTOF MS/MS spectra, which are the characteristic fragments of 39-O-desmethyl SRL. In addition, the presence of *m*/*z* = 389.2300 and *m*/*z* = 697.4282 confirmed the hydroxylation at C(12). The 12-hydroxy SRL characteristic fragment with *m*/*z* = 711.4442 had a corresponding fragment in the MS/MS spectra of the present metabolite at *m*/*z* = 697.4282 due to demethylation at C(39). For more details, please see the Supplemental Materials, pages 129–133.

#### 3.7.3. 11-Hydroxy, 16-O-Desmethyl SRL

The metabolite with *m*/*z =* 938.5236 eluting at 26.94 min contained a fragment with *m*/*z* = 607.3971, which is typical for C(16) O-demethylation and excludes metabolic modification, both demethylation and hydroxylation, at carbons C(17)–C(49), C(51), and C(52). The detection of *m*/*z* = 369.2399 and 407.2192 further confirmed demethylation at C(16). The QTOF spectra of the present metabolite showed a characteristic fragment of *m*/*z =* 709.4633, indicating hydroxylation at C(11). This fragment corresponds to the characteristic fragment with *m*/*z =* 723.4806 in the 11-hydroxy SRL MS/MS spectra. For more details, please see the Supplementary Materials, pages 134–138.

### 3.8. Determination of V_max_, K_m_, and the Intrinsic Clearance (CL_int_) for the Formation of SRL Metabolites by Pooled HLM

**Figure 8:**
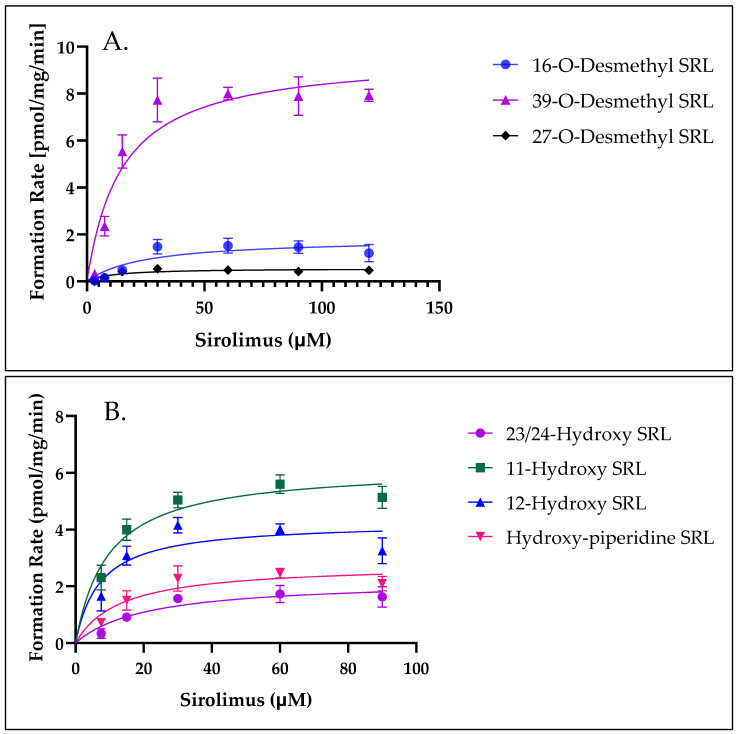
Michaelis–Menten plot for the formation of demethylated sirolimus metabolites (**A**) and hydroxylated metabolites (**B**). Each data point represents the 95% confidence interval (*n* = 4).

The formation rates of hydroxy SRL and desmethyl SRL were analyzed and fitted using Michaelis–Menten kinetics. This approach enables the determination of kinetic parameters such as V_max_ (maximum reaction velocity) and K_m_ (substrate concentration at half-maximal velocity), which provide insight into the enzymatic activity and substrate affinity for these metabolites. (Table 3, Figure 8). Our results indicated that, in addition to 16-O-desmethyl and 39-O-desmethyl SRL, 23/24-hydroxy, 12-hydroxy, 11-hydroxy, and piperidine-hydroxy SRL were formed by HLM using the present incubation conditions. To compare the formation of the various metabolites and to predict the human hepatic clearance, the apparent intrinsic clearances of the formation of the metabolites (CL_int_ = *V*_max_/*K*_m_) were calculated. The kinetic parameters for SRL metabolism are summarized in (Table 3). Notably, the major hydroxylated metabolites displayed comparable affinity and capacity for their formation indicating similar enzymatic efficiency.

Interestingly, the apparent K_m_ values for the demethylated SRL metabolites were nearly identical to those of the hydroxylated metabolites, suggesting equivalent affinity and catalytic capacity for both types of metabolic processes. Among the major metabolites, 11-hydroxy, 12-hydroxy, and 39-O-desmethyl SRL exhibited the highest formation rates, with V_max_ values of 6.2, 4.3, and 9.6 pmol/mg protein/min, respectively.

To estimate the extent of SRL metabolism and predict in vivo metabolism rates, the apparent intrinsic clearance (CL_int_ = V_max_/K_m_) was calculated. The total Cl_int_ derived from the formation of hydroxylated and demethylated SRL metabolites, was determined to be 2.35 µL/mg protein/min.

### 3.9. DFT Calculations

Our objective was to conduct a detailed investigation of demethylation and hydroxylation sites, leveraging DFT calculations to clarify the likelihood of biotransformation at these specific in vitro sites. The outcomes of a DFT computational mechanistic study of demethylation events are illustrated in (Table 4). A similar table with calculated energy for hydroxylated metabolites can be found in Supplementary Materials page 144. When examining demethylation, the order of preference follows: C16 first, then C39, and finally C27. Position C39 follows closely behind position C16 because while C16 is inherently more accessible, the energy recovered from hydrogen abstraction at position C39 contributes to its demethylation efficiency, elevating it to second in priority. Aligned with in vitro data, we observed a higher yield of formation of 16-O-desmethyl SRL and 39-O-desmethyl compared to 27-O-desmethyl. While position C27 might initially appear more favorable due to the presence of inductive side groups that enhance electron withdrawal and make the methyl group more susceptible to enzymatic removal, the overall ΔG (Gibbs free energy change) indicates a different order of preference. The energy required for demethylation at position C27 is higher, making it less favorable compared to other positions. In contrast, the ΔG for demethylation at positions C16 and C39 suggests that these sites require less energy, with position C39 in particular benefiting from the energy released during hydrogen abstraction. This insight underscores how thermodynamic considerations can influence metabolic pathways, even when structural factors might initially suggest an alternative preference.

### 3.10. Molecular Dynamics Simulations

To further elucidate SRL–CYP3A4 interactions, we conducted MD trajectory analyses for both hydroxylation and demethylation metabolites. We assessed the intermolecular distance between SRL’s key functional groups (e.g., methyl groups, hydroxylation sites) to the CYP3A4 heme cofactor to gauge and support the accuracy of site of modification predictions.

**Figure 9:**
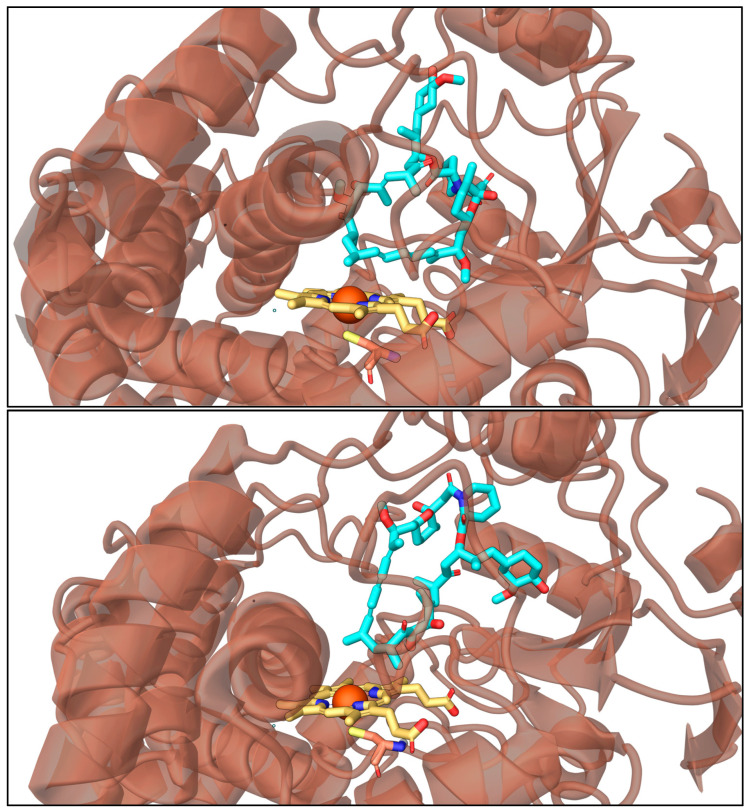
Representative 3D structures from the molecular dynamics (MD) simulations of SRL in the CYP3A4 binding pocket. The orange ribbon represents the CYP3A4 protein backbone. The heme cofactor is shown in yellow (carbon atoms) with an orange sphere representing the iron (Fe) atom. SRL is depicted with cyan (carbon), red (oxygen), and blue (nitrogen) atoms. This figure compares two distinct conformations observed during MD simulations: the non-flipped orientation (**top panel**) and the flipped orientation (**bottom panel**). The panels highlight the shift in spatial positioning of key functional groups, particularly the C23 and C24 regions of SRL, which alternate in proximity to the Fe atom depending on the orientation. This spatial shift may contribute to differences in metabolic accessibility at these sites. The figure is intended to provide a visual summary of conformational dynamics that may influence metabolite formation, as explored in the corresponding simulation analysis.

**Figure 10:**
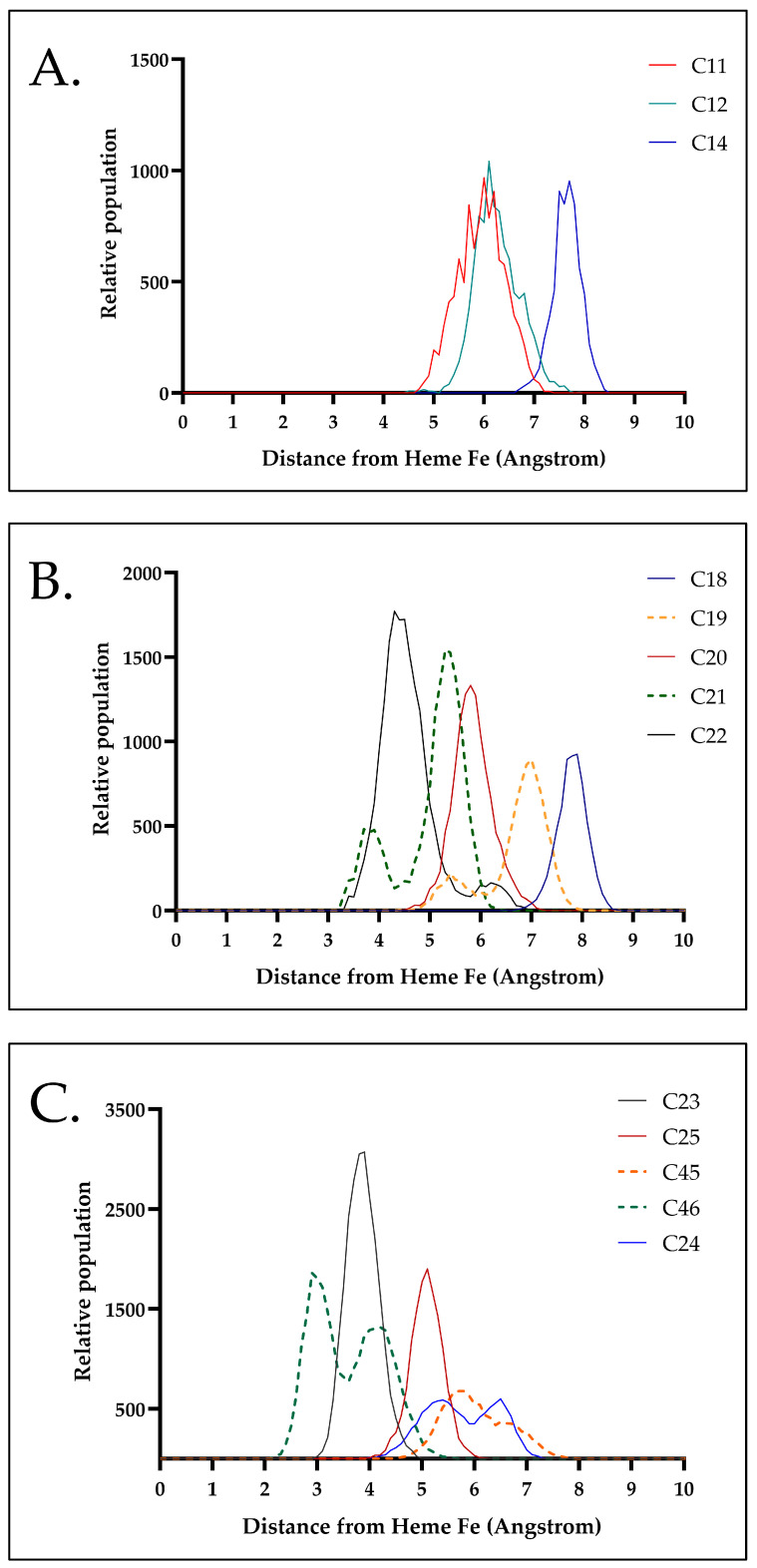
Distribution of the distance order for each S1, S2, and S3 SOM according to the docking poses with the CYP3A4 Fe. (**A**) S1 [C(11), C(12), C(14)], (**B**) S2 [C(18)–C(22)], (**C**) Flipped orientation is chosen since it is decisive for the location of the functional group toward the heme. The same figure for demethylation products can be found in Supplementary Materials page 143.

Intuitively, SRL can be oriented in the enzyme’s active site in two conformations, barring a C2 rotation. This is prevalent as it will affect how the macrolide ring and substituents position themselves relative to the heme cofactor and nearby residues. Because the CYP3A4 pocket is relatively small and SRL is a large molecule, we note that energetically accessible free rotation of SRL is limited, making the initial orientations potentially decisive for how functional groups are oriented toward the heme (Figure 9). To further investigate potential hydroxylation sites, we categorized them into three sets—S1 (C(11), C(12), C(14)), S2 (C(18)–C22), and S3 (C(23), C(24), C(25), C(45), C(46))—and monitored their interactions with the heme cofactor (Figure 10). We note that S1 and S2, unlike S3, cannot adopt a flipped orientation due to spatial constraints from the C(39)–OCH_3_ side chain. Thus, given the scope of this study, we explored one orientation for each case; however, we do acknowledge that more rigorous evaluation in the future may be required. In vitro findings have shown that most hydroxylation occurs at S1 and S3, whereas S2 is seldom hydroxylated. Consequently, our goal was to explore S2 in more detail to understand why hydroxylation is not observed in that region.

The results showed that S1 outcompeted S2, in that the distances for hydroxylation events were smaller in S1 than S2, alluding to favoring hydroxylation in that region rather than on the conjugated triene bonds (C(18)–C(22)) (Figure 10B).

Another ambiguity addressed by MD was a more in-depth investigation of hydroxylation at positions 23/24 and 45/46. For all of the previous studies focused on rapalogs’ metabolites identification, these sites have been reported together as pairs without additional analysis. Hydroxylation can occur at both C(45) and C(46) and methanol elimination cannot be excluded for either position. However, MS^n^ experiments could not distinguish between C(45)- and C(46)-hydroxylation due to the lack of specific diagnostic fragments. Consequently, pinpointing the exact hydroxylation site for these two carbons is not feasible by MS^n^ alone. Using MD simulations, we observed that C(46) lies closer to the CYP3A4 heme cofactor, suggesting a higher probability of hydroxylation at C(46) than at C(45) (Figure 10C). A similar approach was employed to clarify the hydroxylation sites at C(23) and C(24). In this case, water loss may occur at either position. The absence of site-specific fragments similarly precluded a definitive identification of the hydroxylation site via MS^n^. MD simulations indicated that C(23) remains in closer and more frequent proximity to the heme cofactor, implying a greater likelihood of hydroxylation at C(23) compared to C(24) (Figure 10C).

## 4. Discussion

There are significant and concerning discrepancies regarding SRL drug metabolism in preexisting literature. In contrast to other immunosuppressive drugs such as tacrolimus [53,54], everolimus [39], cyclosporine [55], and the structurally related anti-cancer drug temsirolimus [56], the manufacturer did not publish a definitive, comprehensive drug metabolism study. Instead, the full picture of SRL metabolism had to be pieced together from several diverse publications that describe partial aspects. Several of these publications claim SRL metabolite structures without showing data that prove beyond any reasonable doubt the validity of the proposed structures [36,38,57,58]. To further confusion, non-enzymatic degradation of sirolimus and its metabolites to the ring-open seco derivatives has incorrectly been considered part of the sirolimus metabolic pathways [40,41]. The interpretation of SRL metabolite data has been further complicated by the use of two different numbering nomenclatures in the literature. While originally the Chemical Abstracts nomenclature was used for SRL and its metabolites [37], later publications used the International Union of Pure and Applied Chemistry (IUPAC) nomenclature [59]. Both nomenclatures have been used for sirolimus in parallel. For example, while it is 39-O-desmethyl SRL according to IUPAC, the same metabolite is 41-O-desmethyl SRL according to the Chemical Abstract numbering nomenclature. The numbering of the SRL molecule and its metabolites according to the said two nomenclatures is compared in the Supplementary Materials, pages 139–141. The parallel use of two numbering nomenclatures for SRL and its metabolites has even confused government regulatory agencies such as the United States FDA. Its guideline “Class II Special Controls Guidance Document: Sirolimus Test Systems” [60] recommends testing of the following SRL metabolites in specificity studies of sirolimus test systems used for therapeutic drug monitoring: 41-O-desmethyl-, 7-O-desmethyl, 12-hydroxy-, 16-O-desmethyl, 39-O-desmethyl, 27, 39-O-didesmethyl-, and dihydroxy SRL. However, the first two metabolites follow the Chemical Abstract numbering nomenclature and the others the IUPAC nomenclature, so that in the FDA’s recommendation 41-O-desmthyl and 7-O-desmethyl are identical to 39-O-desmethyl and 16-O-desmethyl SRL, respectively. The list of the SRL metabolites included in said FDA guideline is based on an older review article [61] and seems arbitrary, since as shown in the present study, there are several other important hydroxylated metabolites such as 11-hydroxy, 24-hydroxy, 25-hydroxy, and 46-hydroxy SRL that also have been detected in the blood of transplant patients [20]. Moreover, the FDA guideline does not further specify dihydroxy SRL metabolites and, based on the present study, there are several hydroxylation positions, all of which could potentially be combined to result in various dihydroxy SRL metabolites. Therefore, it seemed necessary and timely to revisit the human metabolism of SRL in a comprehensive in vitro study including detailed evidence on how the structures of the SRL metabolites were identified.

SRL undergoes extensive fragmentation, as is evident in both spectra acquired from the QTOF and ion trap mass spectrometers (Figure 2). Therefore, mass spectrometry based on electrospray ionization in conjunction with collision-induced dissociation proved to be an appropriate choice for metabolite structure elucidation as this resulted in detailed, informative fragmentation. This was also found for the successful identification of the human metabolites of sirolimus derivatives [39,41,42]. In addition, only fragments were used for the identification of metabolites, the structures of which had been confirmed using high-resolution mass spectrometry by comparing their measured exact mass with the theoretical exact mass of the proposed structure. The predefined acceptable difference was ≤Δ5.0 ppm. Nuclear magnetic resonance (NMR) spectroscopy is considered the gold standard for structural identification but, as also discussed for the structural identification of the metabolites of sirolimus derivatives [39,41,42], was not an option in the present study. The limitations of NMR spectroscopy in comparison to mass spectrometry are its relatively low sensitivity, the required high purity of analytes, and that the analyte must be soluble and stable in pure organic solvents for extended periods of time while NMR spectra are acquired. As the present study showed, many metabolites were generated only in small quantities, particularly secondary metabolites. It was also often not possible to completely chromatographically separate metabolites, so the structural evaluation had to be carried out in mixtures. Moreover, it is well established that SRL and most likely its metabolites are not stable in pure organic solvents as is required for NMR spectroscopy [4].

Jacobsen et al. [36] described the structures of 12 SRL metabolites after incubation with human liver microsomes and Filler et al. [20] described the structures of 10 SRL metabolites in the blood of pediatric patients after kidney transplantation. However, neither study provided sufficient information regarding precisely how these structures were derived. The present study confirms these structures, with the exception of the 48-hydroxy SRL metabolites described by Jacobsen et al. [36], which most likely is an error by Jacobsen et al., and provides detailed MS^n^ and high-resolution mass spectrometry-based information to support the confirmed structures. In addition, the structures of six so-called second-generation di-hydroxylated, di-O-demethylated, and hydroxylated- O-demethylated metabolites were identified in the present study. These are significant, as their interference testing is required by the aforementioned FDA guideline. There were two primary reasons why the second-generation metabolites could be isolated and structurally identified in the present study. The gradient used for the semi-preparative and analytical HPLC assays was designed to also separate earlier eluting peaks, where the more hydrophilic second-generation metabolites could be expected. Additionally, the influence of microsomal protein concentrations, incubation time, and SRL concentrations on the rate of metabolite formation and the metabolite pattern generated was systematically studied. Specifically, an extended incubation time enabled the formation and detection of second-generation metabolites.

The ring-open seco degradation products of SRL and its metabolites described in the literature [38,40,62] are most likely artifacts generated during sample processing. During the present study, care was taken to avoid degradation caused by the acidification of solvents, and strict control of the evaporation steps was maintained. Accordingly, no seco degradation products were detected, confirming that the formation of seco derivatives of SRL metabolites is not a metabolic pathway as previously described [40,62].

Drugs like SRL, which have multiple potential hydroxylation sites, can greatly benefit from computational methods to precisely identify the site of metabolism. By characterizing conformational shifts, we located the atoms nearest to CYP3A4’s catalytic center as an indicator of higher oxidation likelihood. This is particularly relevant for predicting hydroxylation and demethylation events. Given the complexities of producing SRL metabolites—compounded by the limitations of purity, quantity, and stability that hinder techniques like NMR spectroscopy—these computational approaches present additional support for pinpointing the most probable site of metabolism.

Using DFT calculations, it is possible to predict the thermodynamic favorability of hydroxylation and demethylation. In the case of SRL metabolism, the chemical events are hydrogen abstraction (for hydroxylation) and methyl abstraction (for demethylation). These are followed by the formation of a radical intermediate, which ultimately transitions into the final product. Throughout this mechanism, reaction Gibbs free energy (ΔG kcal/mol) values are calculated to refine our understanding of the reaction’s thermodynamics. Importantly, the results support the formation of both 23-hydroxy and 24-hydroxy SRL, with a preference for 23-hydroxy SRL and of both 45-hydroxy and 46-hydroxy SRL, with a preference for 46-hydroxy SRL. Guided by MD simulations, we propose that the chromatographic peak observed at 29.99 min corresponds to 46-hydroxy SRL, which displays a higher conformational population. In contrast, the peak at 30.99 min is likely associated with 45-hydroxy SRL.

SRL metabolites are clinically important for several reasons. One of the rationales for the aforementioned FDA guideline is that several of the metabolites are known to cross react with the antibodies used in therapeutic drug monitoring assays supporting dose adjustments in transplant patients to keep the SRL blood concentrations within the therapeutic window [63,64,65]. At least 16-O-desmethyl SRL has inhibitory activity on cell proliferation similar to that of SRL [66], and it is used as “novolimus” coated on coronary drug-eluting stents [13]. The only other metabolites tested for their immunosuppressive activities are 39-O-desmethyl SRL, with 10% of the immunosuppressive activity of SRL, and a hydroxy SRL, most likely 11-hydroxy and/or 12-hydroxy SRL, with 7% activity as tested in a phytohemagglutinin-stimulated human lymphocyte proliferation assay [37]. None of the other metabolites have been tested, and except for 16-O-desmethyl SRL, the potential interactions of the metabolites with the mTOR pathway are largely unknown. Despite the well-documented efficacy of SRL, a significant gap remains in our understanding of how its metabolites contribute to clinically relevant proliferation inhibition and metabolic adverse effects. Given the structural diversity of these metabolites and their possible interactions with biological pathways, their impact on SRL’s pharmacodynamics/toxicodynamics cannot be overlooked.

While this study provides a comprehensive structural identification of 21 SRL metabolites using HLM, several limitations should be acknowledged. The biological activity and potential toxicological effects of the identified metabolites were not assessed and remain an important focus for future research. Although HLMs effectively capture major phase I metabolic processes, they do not encompass the full complexity of in vivo metabolism, such as phase II conjugation or extrahepatic pathways. Moreover, the use of pooled microsomes prevents the assignment of specific CYP isoforms to individual metabolic transformations, and the current work emphasizes structural elucidation over quantitative pharmacokinetics.

Building upon the workflows developed in this study, ongoing investigations in our laboratory are focused on elucidating the pharmacological and toxicological relevance of SRL metabolites. This research will advance our understanding of SRL’s metabolic landscape and support the development of more precise and personalized treatment strategies.

## 5. Conclusions

In the present study, twenty-one SRL metabolites were generated following incubation with pooled HLM, isolated using semi-preparative HPLC, and structurally identified through MS^n^ and high-resolution mass spectrometry. Compared to previous studies that reported only a limited subset of SRL metabolites, often without full structural confirmation, this study significantly expands the known metabolic landscape by identifying 21 structurally elucidated metabolites, including several previously uncharacterized second-generation derivatives.

MD simulations between SRL and CYP3A4 provided mechanistic insights into the positioning, binding orientation, and potential metabolic sites of SRL within the enzyme’s active site. These time-resolved simulations, especially when complemented by quantum mechanical calculations, allow for a more detailed understanding of SRL’s metabolic fate and the thermodynamic feasibility of specific reactions.

The biological activity of SRL metabolites, including their potential modulation of the mTOR pathway and associated toxicities, remains largely unexplored. The experimental workflows established in this study offer a robust platform for both the generation and structural identification of SRL metabolites. These methodologies lay the groundwork for future in vitro investigations aimed at systematically assessing the pharmacological and toxicological properties of individual metabolites, thereby contributing to a more comprehensive evaluation of SRL’s efficacy, safety, and metabolic implications.

## Figures and Tables

**Table 1:** Identified sirolimus metabolites.

Hydroxylated metabolites	46-Hydroxy sirolimus
	45-Hydroxy sirolimus
	23/24-Hydroxy sirolimus
	12-Hydroxy sirolimus
	25-Hydroxy sirolimus
	11-Hydroxy sirolimus
	Hydroxy-piperidine sirolimus (a, b, c, d)
	14-Hydroxy sirolimus
	49-Hydroxy sirolimus
Dihydroxy metabolites	12, 24-Dihydroxy sirolimus
Desmethyl metabolites	16-O-Desmethyl sirolimus
	27-O-Desmethyl sirolimus
	39-O-Desmethyl sirolimus
Didesmethyl metabolites	27, 39-O-Didesmethyl sirolimus
	16, 39-O-Didesmethyl sirolimus
Hydroxy-desmethyl metabolites	Piperidine-hydroxy-39-O-desmethyl sirolimus
	12-Hydroxy-39-O-desmethyl sirolimus
	11-Hydroxy-16-O-desmethyl sirolimus

**Table 2:** Comparison of the calculated and measured exact masses of sirolimus fragments utilized to identify metabolite structures.

	Theoretical Mass	Measured Mass	Δppm
Sirolimus	936.5444	936.5444	0.0
A	731.4493	731.4491	0.3
B	763.4756	763.4756	0.0
C	642.3249	642.3250	0.2
D	345.2036	345.2038	0.6
E	703.4544	703.4547	0.4
F	399.2506	399.2508	0.5
G	614.3300	614.3298	0.3
H	485.2510	485.2511	0.2
I	459.2717	459.2718	0.2
J	409.2349	409.2350	0.2
K	607.3969	607.3969	0.0
L	397.2349	397.2363	3.5
M	582.3037	582.3035	0.3
N	453.2248	453.2247	0.2
O	441.2611	441.2610	0.5
P	381.2400	381.2401	0.3
Q	320.1105	320.1104	0.3
R	413.2662	413.2662	0.0

**Table 3:** Apparent enzyme kinetic parameters for the conversion of sirolimus to hydroxylated and demethylated sirolimus metabolites by human liver microsomes. Experiments represent *n* = 4.

Metabolite	V_max_ (pmol/mg protein/min)	K_m_ (µM)	CL_int_ (µL/mg protein/min)
	[95% CI]	[95% CI]	
23/24-Hydroxy SRL	2.221	20.82	0.11
	[1.8–2.8]	[11.35–38.27]	
12-Hydroxy SRL	4.264	7.211	0.6
	[3.674–4.955]	[3.4–13.01]	
11-Hydroxy SRL	6.203	9.577	0.65
	[5.673–6.799]	[6.6–13.43]	
Hydroxy-Piperidine SRL	2.781	13.15	0.21
	[2.350–3.333]	[7.1–23.14]	
16-O-desmethyl SRL	1.828	24.37	0.08
	[1.452–2.393]	[12.20–50.43]	
39-O-desmthyl SRL	9.647	14.95	0.65
	[8.692–10.76]	[10.39–21.51]	
27-O-desmethyl SRL	0.552	9.647	0.06
	[0.47–0.65]	[8.7–10.76]	

**Table 4:** Calculated energy of a methyl abstraction at various carbon centers, energy resulting from hydrogenation, and overall reaction Gibbs free energy (ΔG).

	MO-CH_3_ → MO + CH_3_	MO + H → MOH	MO-CH_3_ + H → MO-H + CH_3_
Demethylation Site	ΔG_demethylation_ (kcal/mol)	ΔG_hydrogenation_ (kcal/mol)	ΔG_Overall_ (kcal/mol)
C16	84.43	−108.48	−24.05
C27	78.12	−90.82	−12.71
C39	85.62	−107.51	−21.89

## Data Availability

The original contributions of this study are contained within the article and its Supplementary Materials. For additional inquiries, please contact the corresponding author. Raw data supporting the conclusions of this study can be made available upon request from the authors.

## Supplementary Materials

The following supporting information can be downloaded at: https://www.mdpi.com/article/10.3390/metabo15070489/s1, List of identified metabolites (page 3), Sirolimus structure, QTOF spectrum, fragmentation pattern, Δppm, comment (4–12), Representative of Optimization Controls (13), Desmethyl sirolimus metabolites 14–36), Didesmethyl Sirolimus metabolites (37–51), Hydroxy Sirolimus Metabolites 52–115), Dihydroxy Sirolimus metabolites (116–122), Hydroxy-desmethyl Sirolimus Metabolites (123–138), Comparison of IUPAC vs. Common Nomenclature of Sirolimus Structure (139–141), Quantum Mechanical/Molecular Dynamics (142–144).
