# Supplementary material for: Human Metabolism of Sirolimus Revisited
Source: Metabolites. 2025 Jul 20;15(7):489. doi: 10.3390/metabo15070489 (PMC12299981; doi:10.3390/metabo15070489)
Supplement: Supplementary file 1 [file metabolites-15-00489-s001.zip › metabolites-3672322-supplementary.pdf]

Human Metabolism of Sirolimus Revisited

Baharak Davari, PhD*, Touraj Shokati, PhD*, Alexandra Ward, Vu Nguyen, PhD*, Jost Klawitter, PhD*, Jelena Klawitter, PhD*, and Uwe Christians, MD, PhD*

Table of Contents

Table of Contents	Page
List of identified metabolites	3
Sirolimus structure, QTOF spectrum, fragmentation pattern, Δppm, comment	4-12
Representative of Optimization Controls	13
Desmethyl sirolimus metabolites	14-16
16-O-Desmethyl Sirolimus	17-23
39-O-Desmethyl Sirolimus	24-30
27-O-Desmethyl Sirolimus	31-36
Didesmethyl Sirolimus metabolites	37-39
16, 39-O-didesmethyl Sirolimus	40-45
27, 39-O-didesmethyl Sirolimus	46-51
Hydroxy Sirolimus Metabolites	52-54
45/46-Hydroxy Sirolimus	55-61
23/24-Hydroxy Sirolimus	62-70
12-Hydroxy Sirolimus	71-77
25-Hydroxy Sirolimus	78-85
11-Hydroxy Sirolimus	86-92
Piperidine-Hydroxy Sirolimus	93-101
14-Hydroxy Sirolimus	102-108
49-Hydroxy Sirolimus	109-115
Dihydroxy Sirolimus metabolites	116
12,23-OH Dihydroxy Sirolimus	117-122
Hydroxy-desmethyl Sirolimus Metabolites	123
Piperidine-Hydroxy, 39-O-Desmethyl Sirolimus	124-128
12-Hydroxy, 39-O-Desmethyl Sirolimus	129-133
11-Hydroxy,16-O-Desmethyl Sirolimus	134-138
Comparison of IUPAC vs Common Nomenclature of Sirolimus Structure	139-141
Quantum Mechanical/Molecular Dynamics	142-144

Identified Metabolites of Sirolimus after Incubation with Human Liver Microsomes (HLM)

Metabolites		Exact Mass
Hydroxy Metabolites		
1)	46/45-Hydroxy sirolimus	952.5393
2)	23/24-Hydroxy sirolimus	
3)	12-Hydroxy sirolimus	
4)	25-Hydroxy sirolimus	
5)	11-Hydroxy sirolimus	
6)	Hydroxy Piperidine sirolimus(a, b, c, d)	
7)	14-Hydroxy sirolimus	
8)	49-Hydroxy sirolimus	
Dihydroxy Metabolites		
9)	(12,24)Dihydroxy sirolimus	968.5342
Desmethyl Metabolites		
10)	16-O-Desmethyl sirolimus	922.5287
11)	27-O-Desmethyl sirolimus	
12)	39-O-Desmethyl sirolimus	
Didesmethyl Metabolites		
13)	27,39-O-Didesmethyl sirolimus	908.5131
14)	16,39-O-Didesmethyl sirolimus	
Hydroxy, desmethyl Metabolites		
15)	Piperidine-Hydroxy-39-O-desmethyl sirolimus	938.5236
16)	12-Hydroxy-39-O-desmethyl sirolimus	
17)	11-Hydroxy-16-O-desmethyl sirolimus	

Structure and MS Fragmentation of Sirolimus

m/z = 936.5444

Structural Formula of Sirolimus

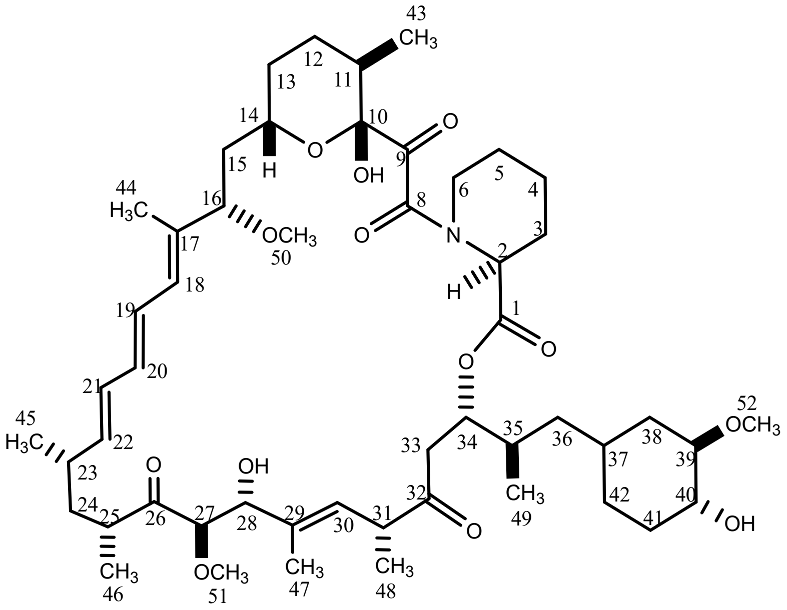

[Sirolimus-Na]+

Chemical Formula: $C_{51}H_{79}NNaO_{13}{}^{+}$

Exact Mass: 936.5444

Structural Formula of Sirolimus Isomers

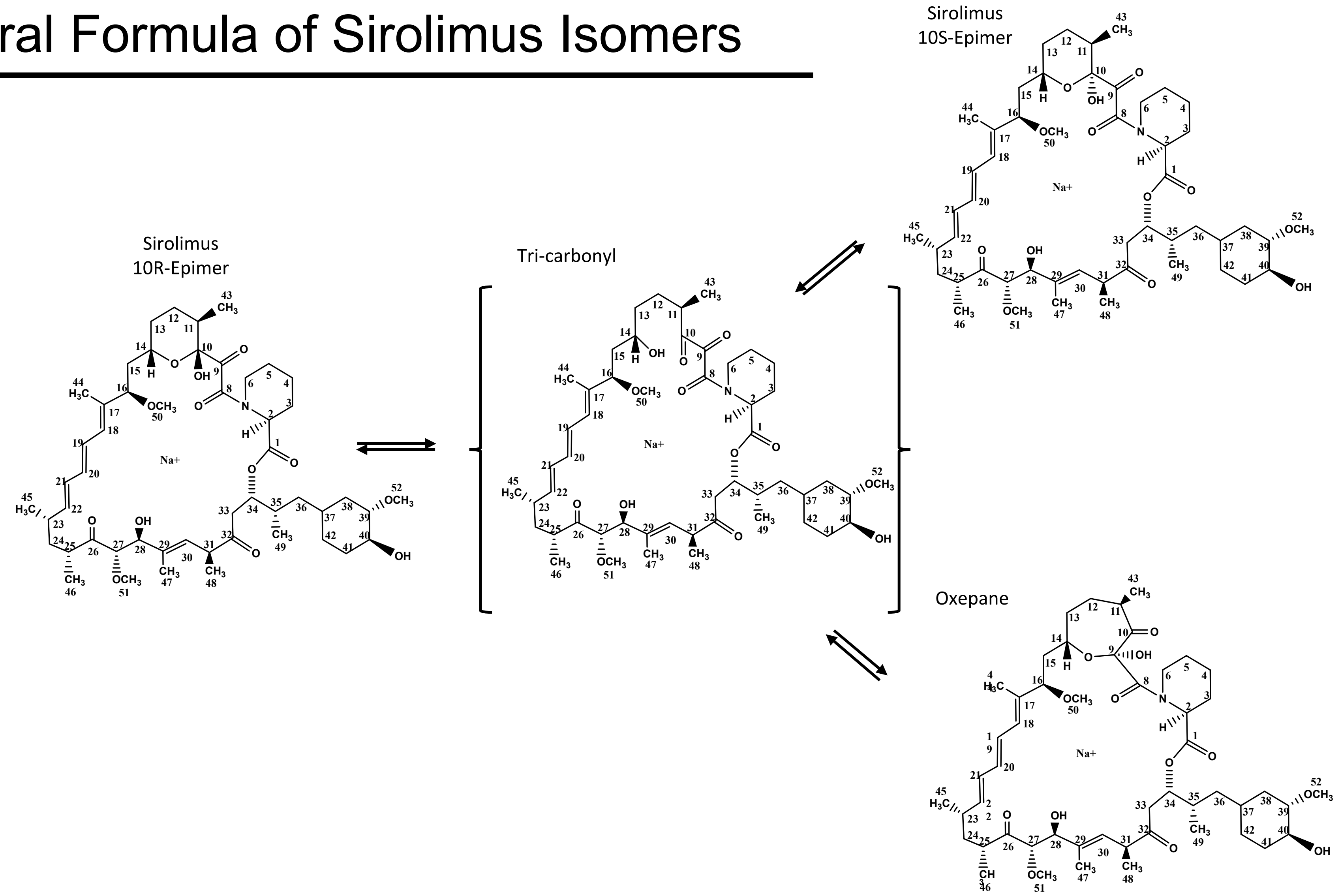

Total Ion Chromatogram of Sirolimus (0.5 µg/ml) Measured by TOF

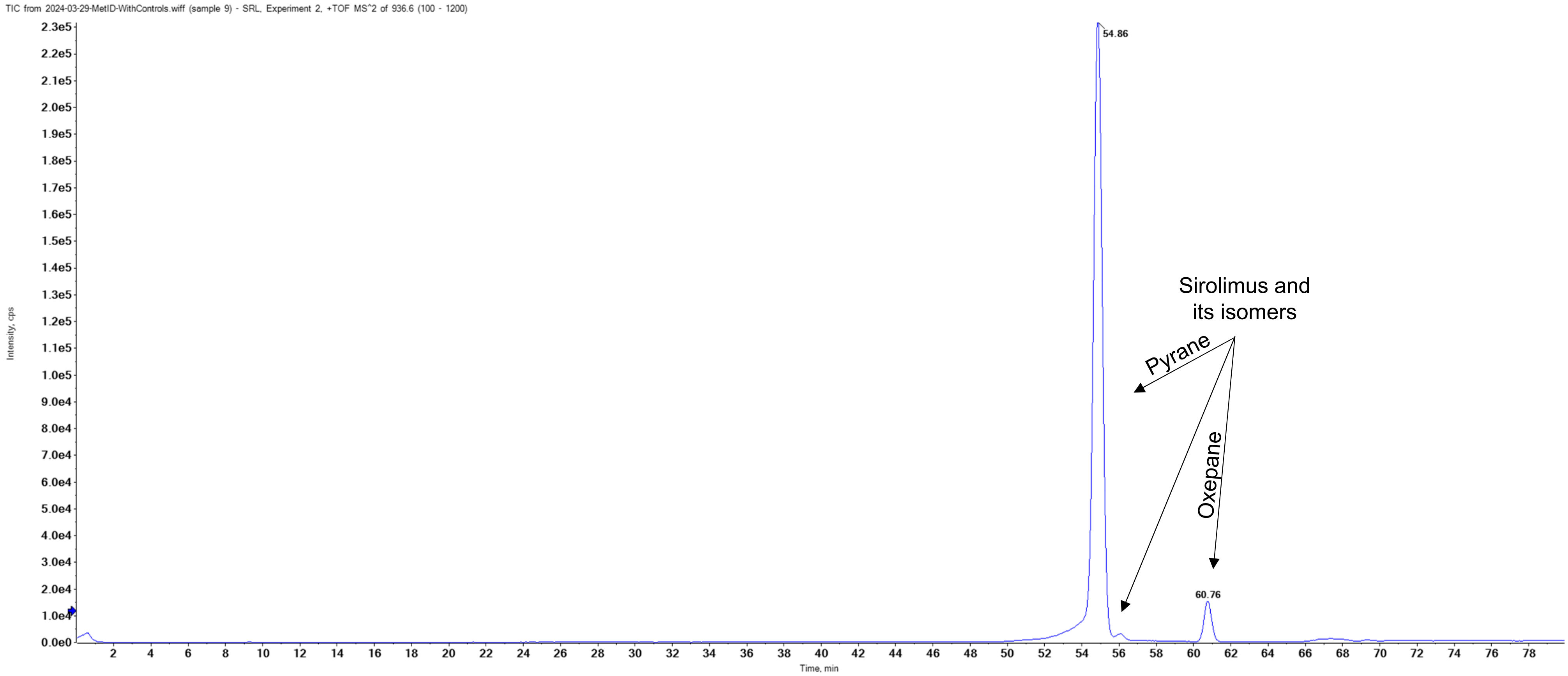

Exact Masses of Sirolimus Adducts and Their Isotopic Pattern Measured by TOF(Direct Infusion 3 mL/hr)

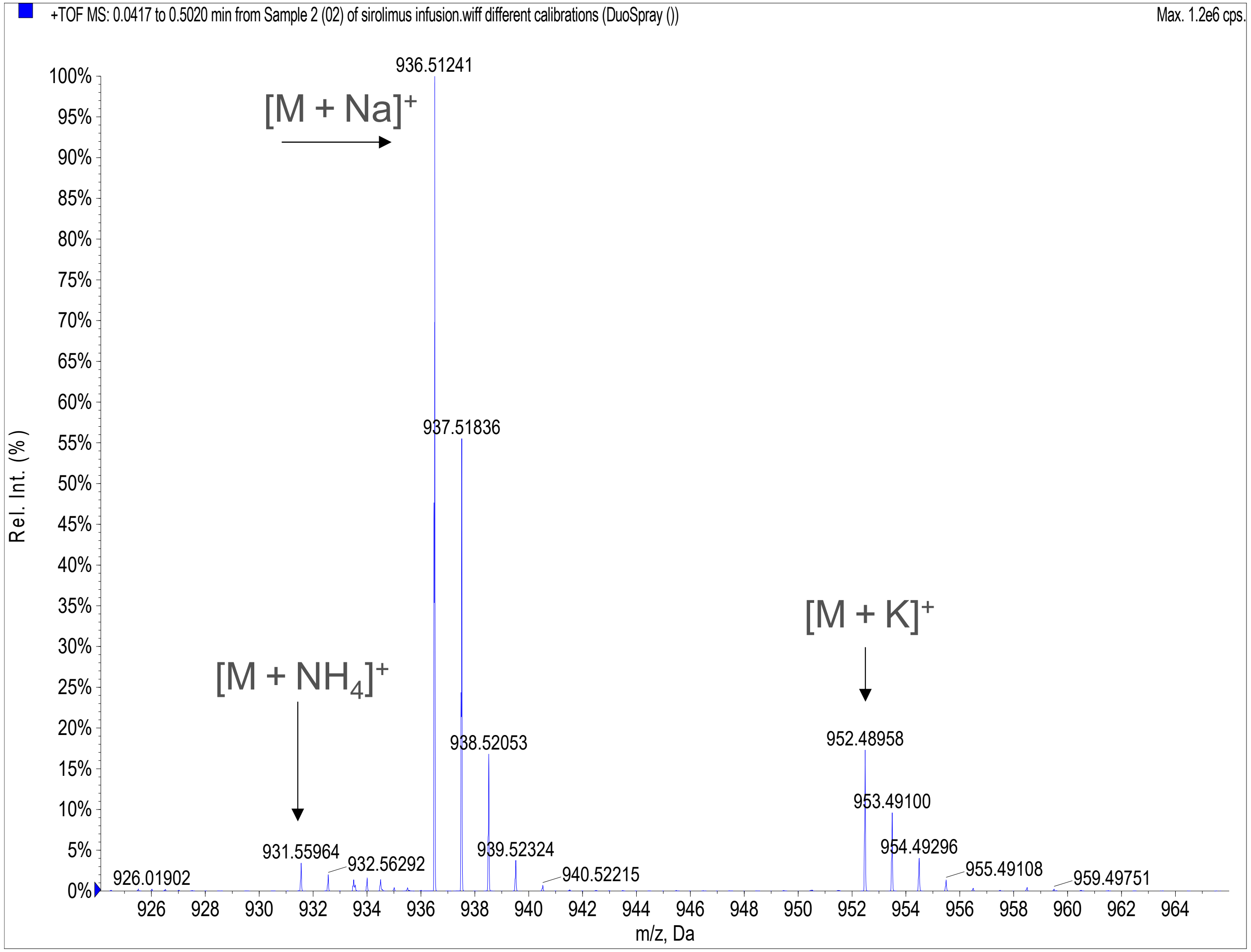

TOF Spectrum of Sirolimus, CE = 65 eV, DP = 110 V

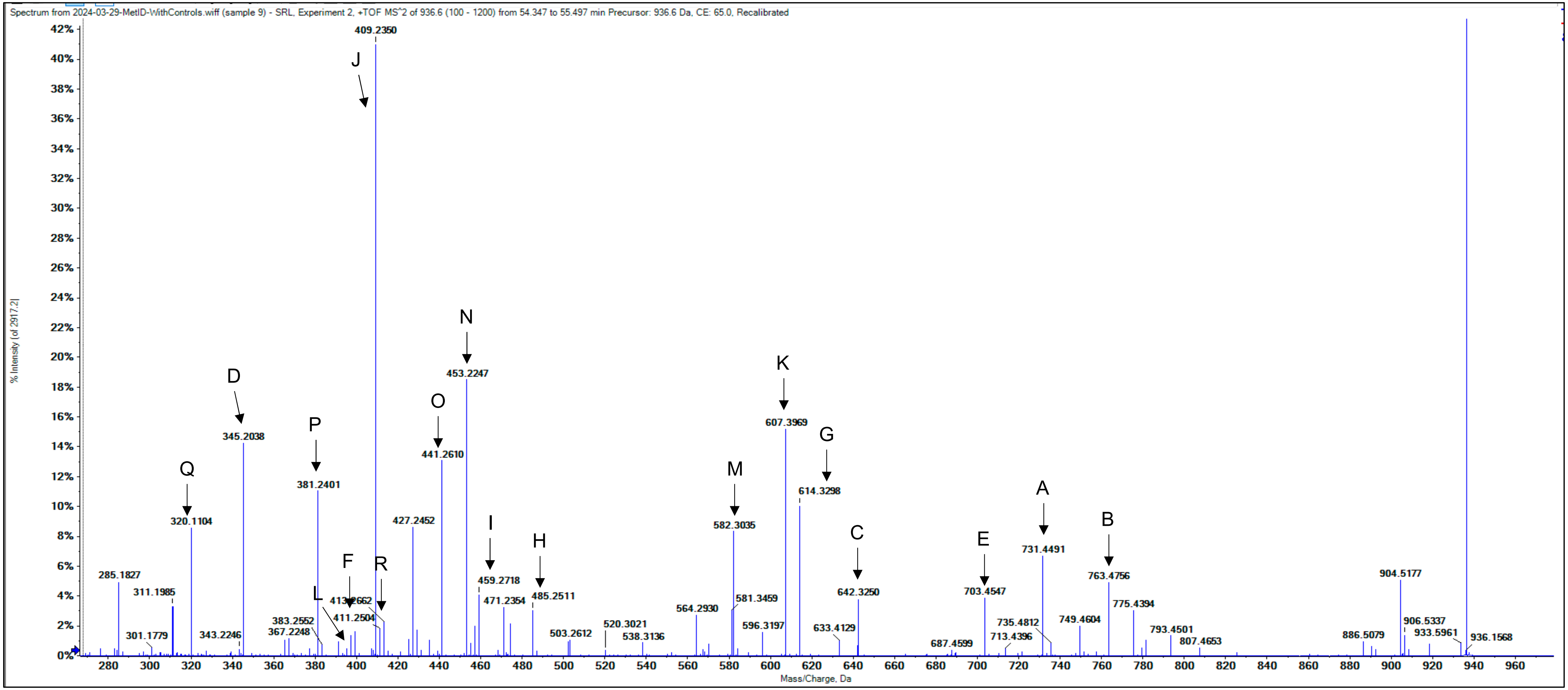

Sirolimus Fragmentation Pattern

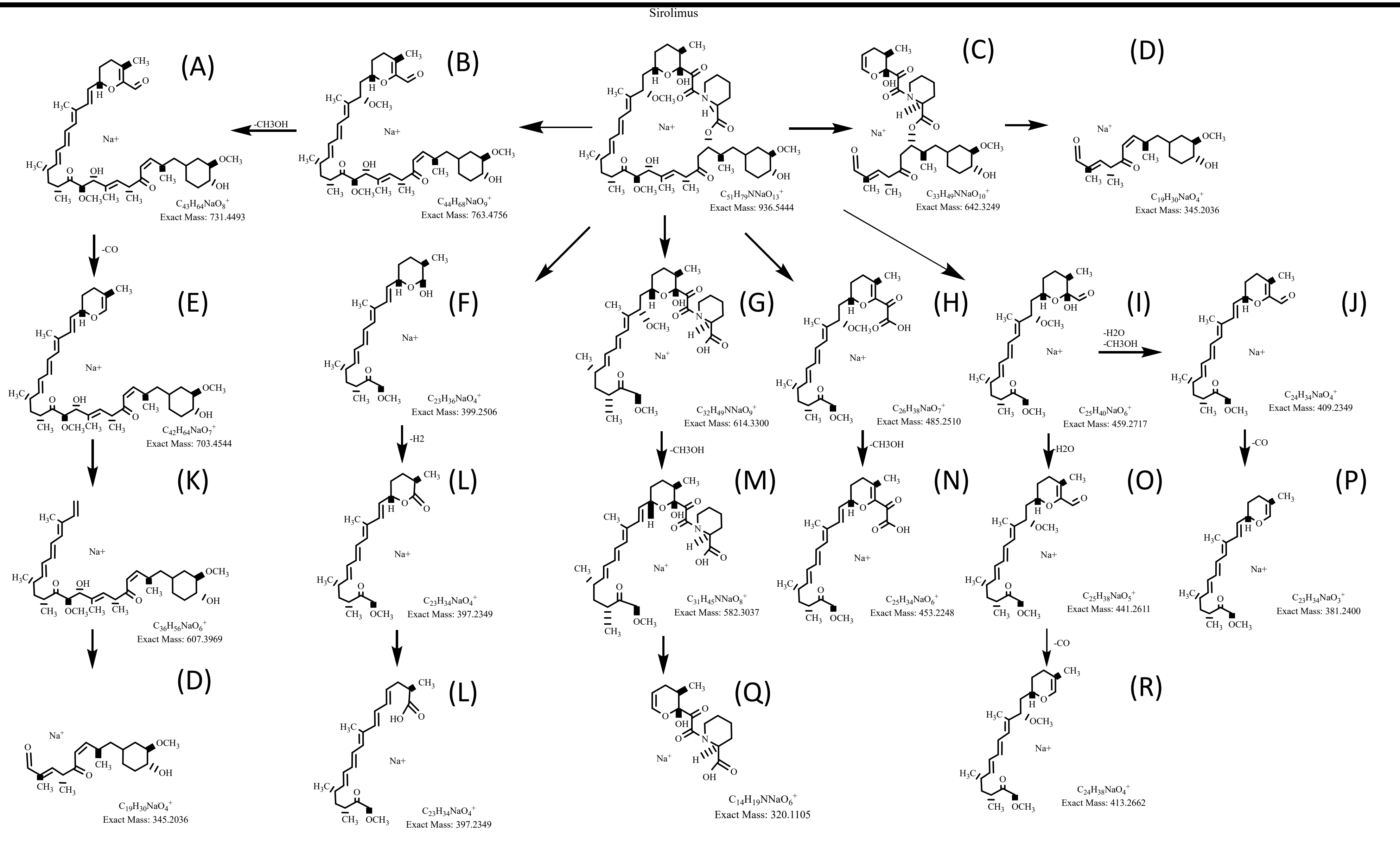

Sirolimus Fragmentation Pathway Continued

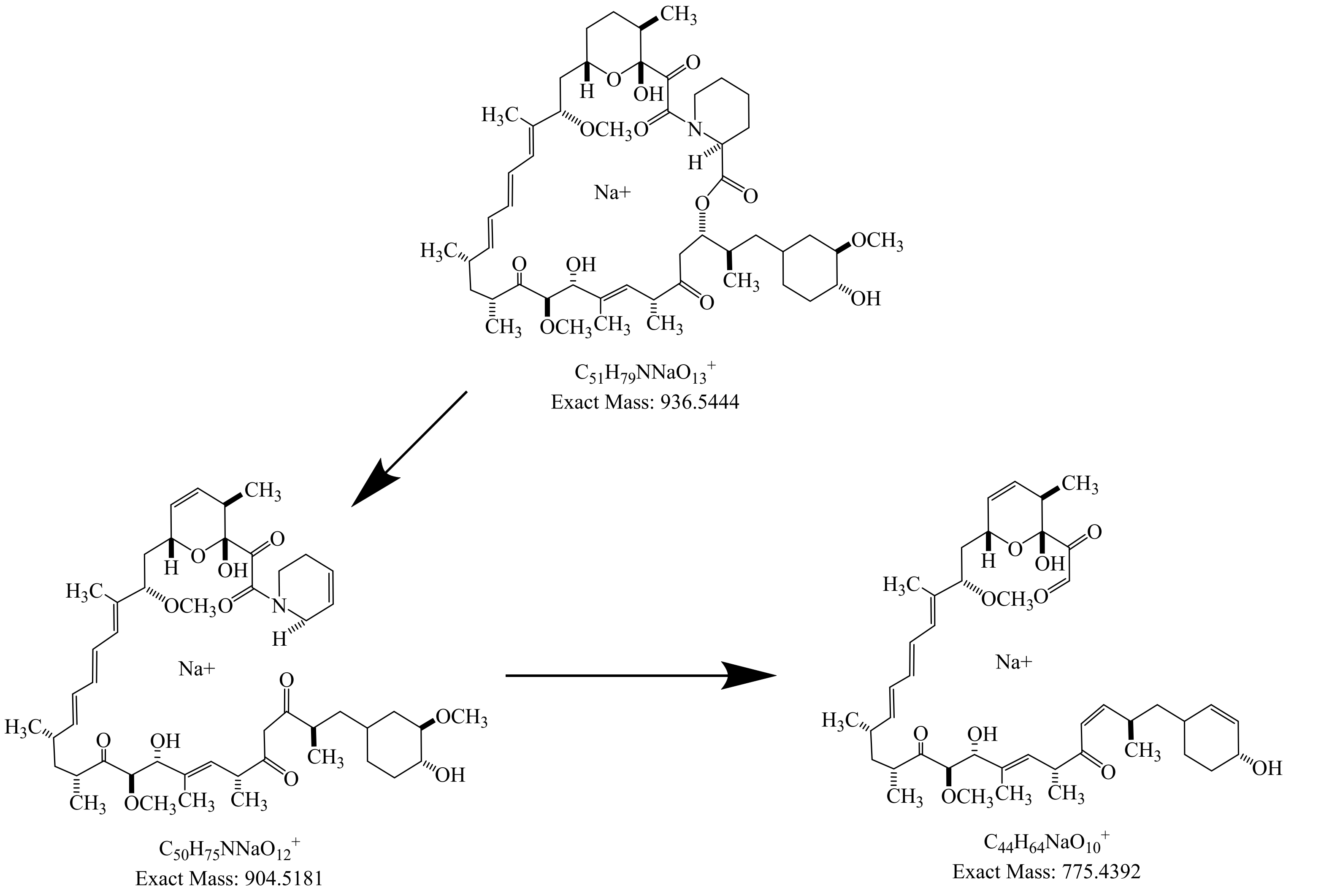

Δppm of Sirolimus Fragments Measured by TOF

	Theoretical mass	Measured mass	Dppm
**Sirolimus**	936.5444	936.5444	0.0
A	731.4493	731.4491	0.3
B	763.4756	763.4756	0.0
C	642.3249	642.3250	0.2
D	345.2036	345.2038	0.6
E	703.4544	703.4547	0.4
F	399.2506	399.2508	0.5
G	614.3300	614.3298	0.3
H	485.2510	485.2511	0.2
I	459.2717	459.2718	0.2
J	409.2349	409.2350	0.2
K	607.3969	607.3969	0.0
L	397.2349	397.2363	3.5
M	582.3037	582.3035	0.3
N	453.2248	453.2247	0.2
O	441.2611	441.2610	0.5
P	381.2400	381.2401	0.3
Q	320.1105	320.1104	0.3
R	413.2662	413.2662	0.0

Representative of Optimization Controls

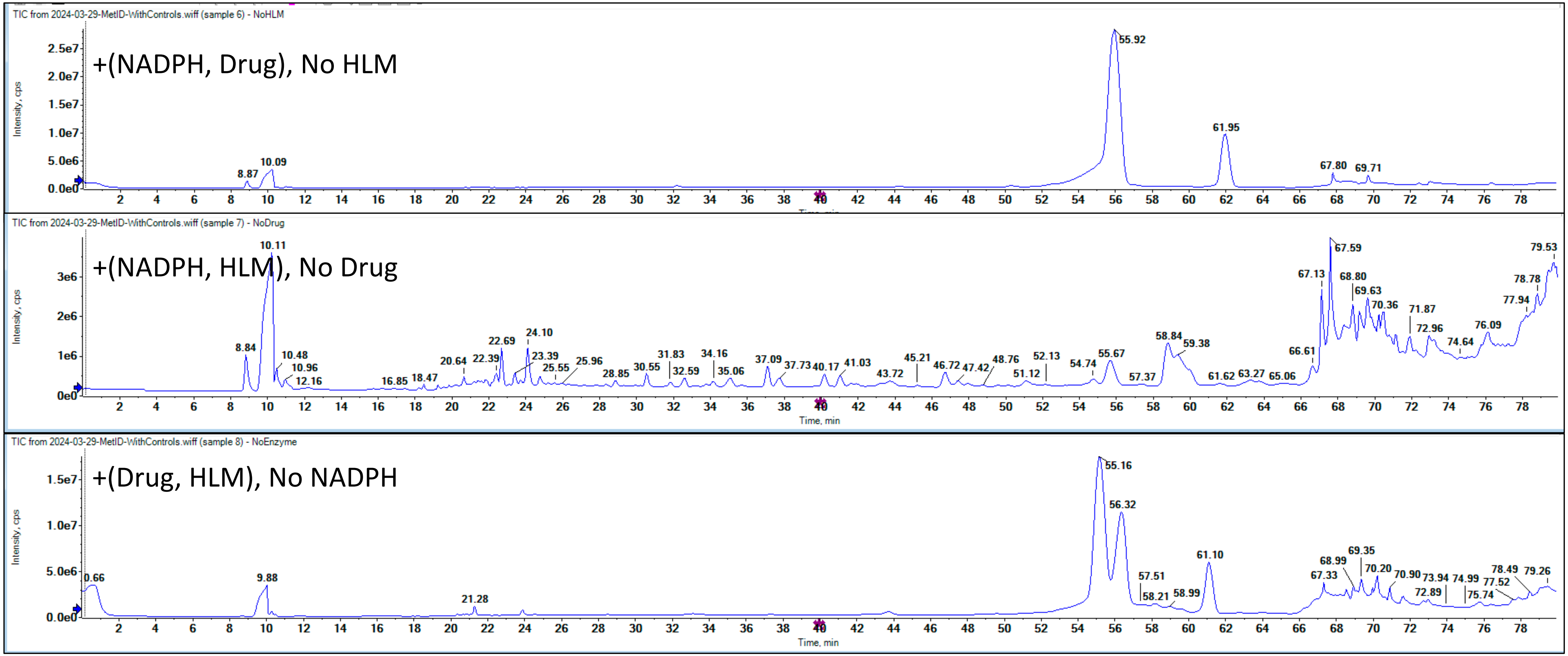

Desmethyl Sirolimus Metabolites

m/z = 922.5287

Desmethyl Sirolimus Metabolites, *m/z*=922.5287

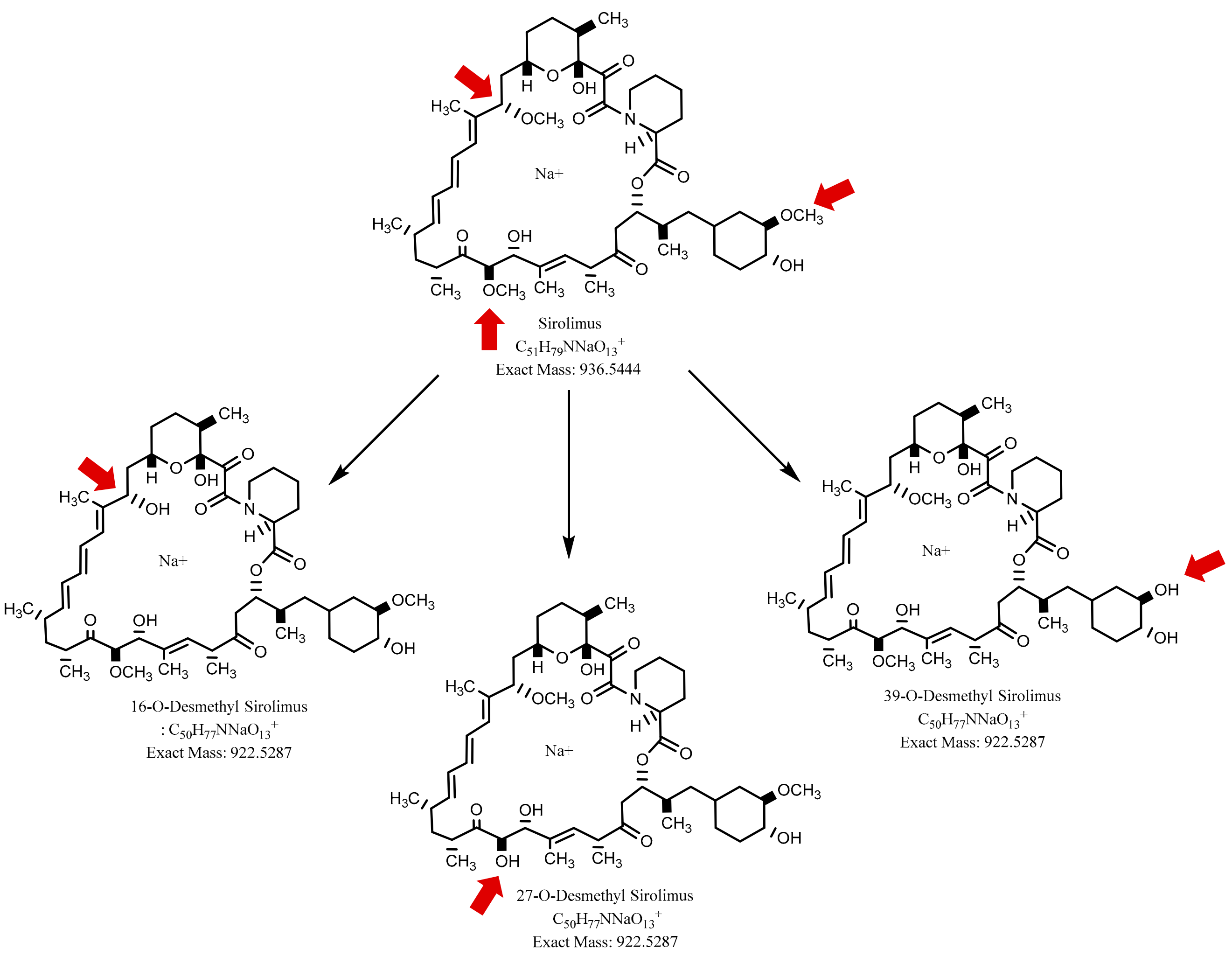

Desmethyl Sirolimus Metabolites
Extracted Ion Chromatogram(EIC), m/z = 922.5287

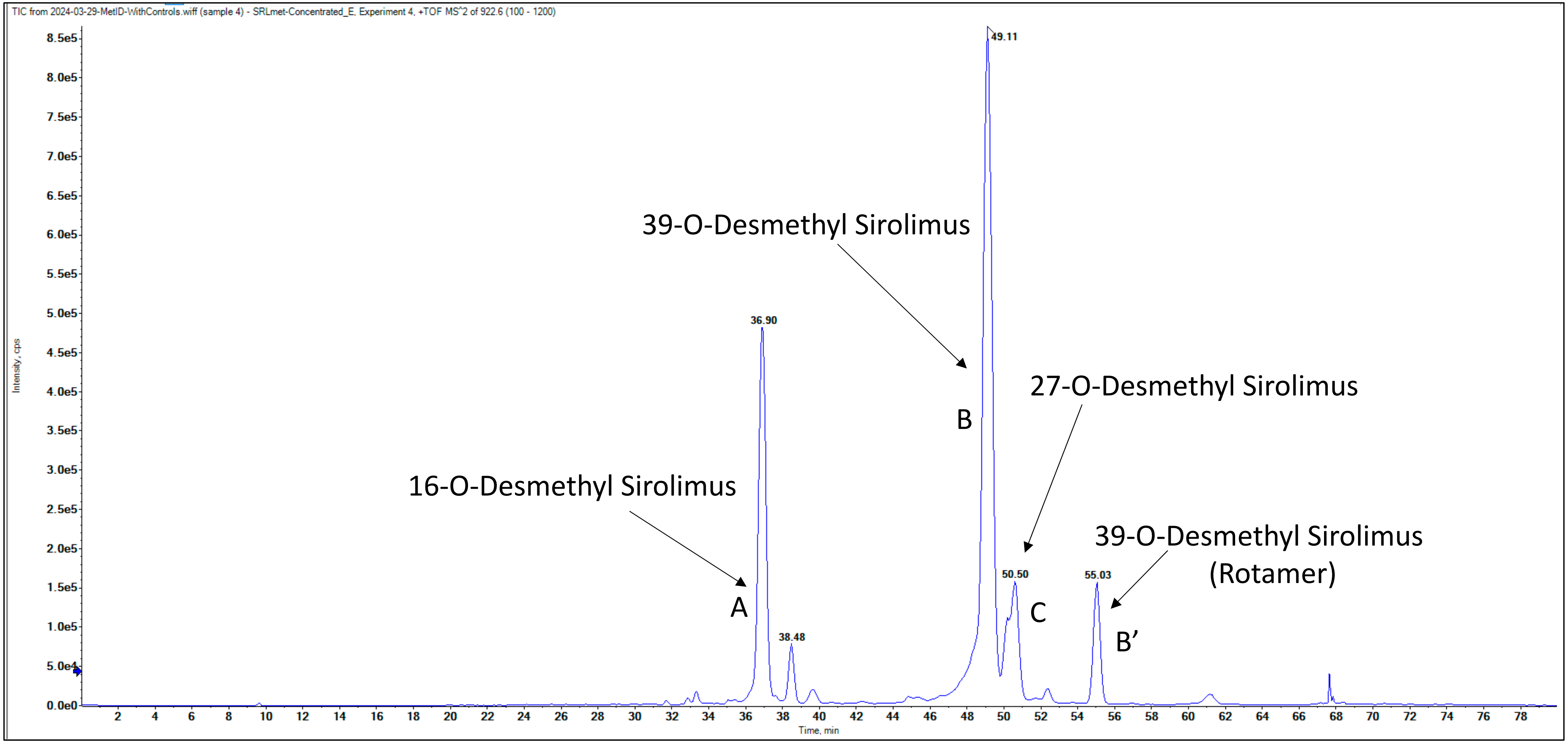

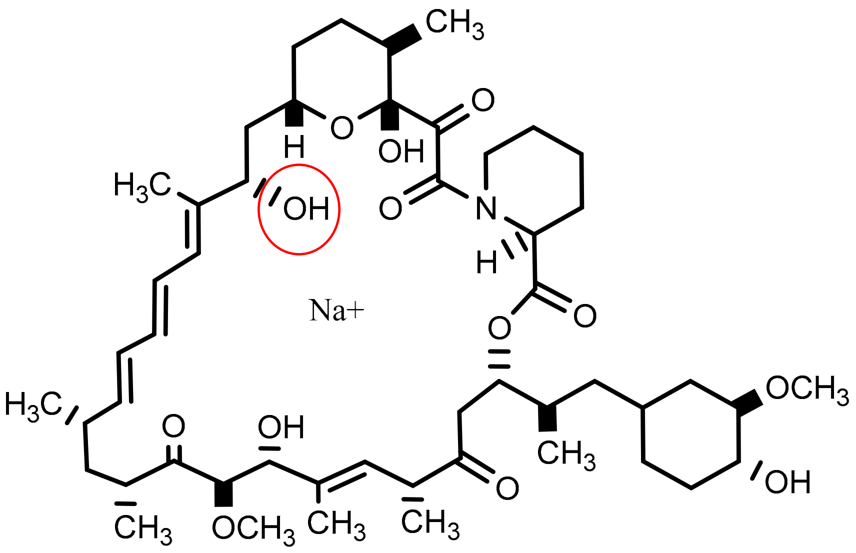

16-O-Desmethyl Sirolimus

: $C_{50}H_{77}NNaO_{13}^{+}$

Exact Mass: 922.5287

16-O-Desmethyl Sirolimus (*m/z* = 922.5287)

16-O-Desmethyl Sirolimus Metabolite
Extracted Ion Chromatogram(EIC), (*m/z* = 922.5287)

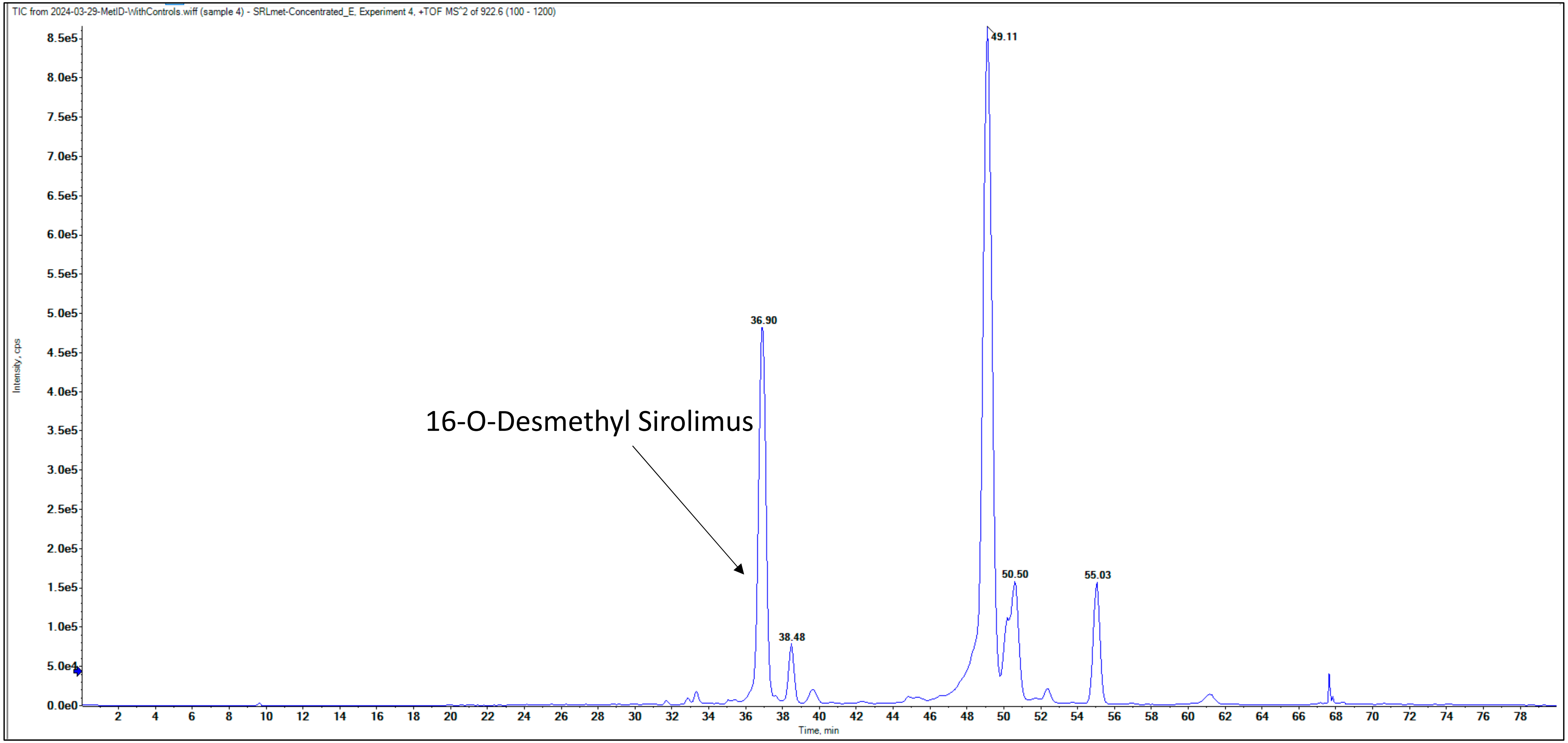

16-Desmethyl Sirolimus Chromatogram (Top)
Mass Spectrum, QTOF Fragmentation, CE=78eV, DP= 110V (Bottom)

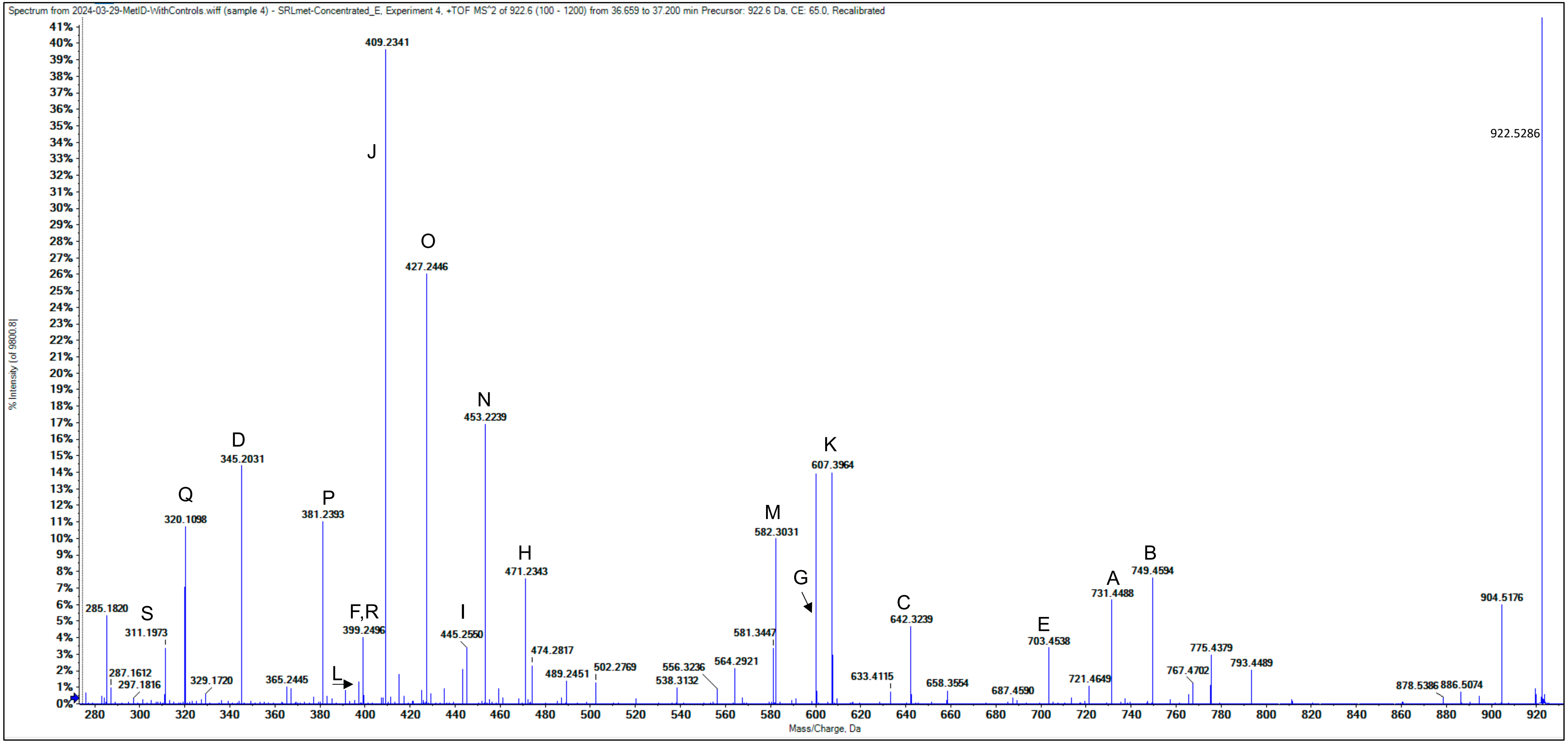

16-O-Desmethyl Sirolimus Fragmentation pattern

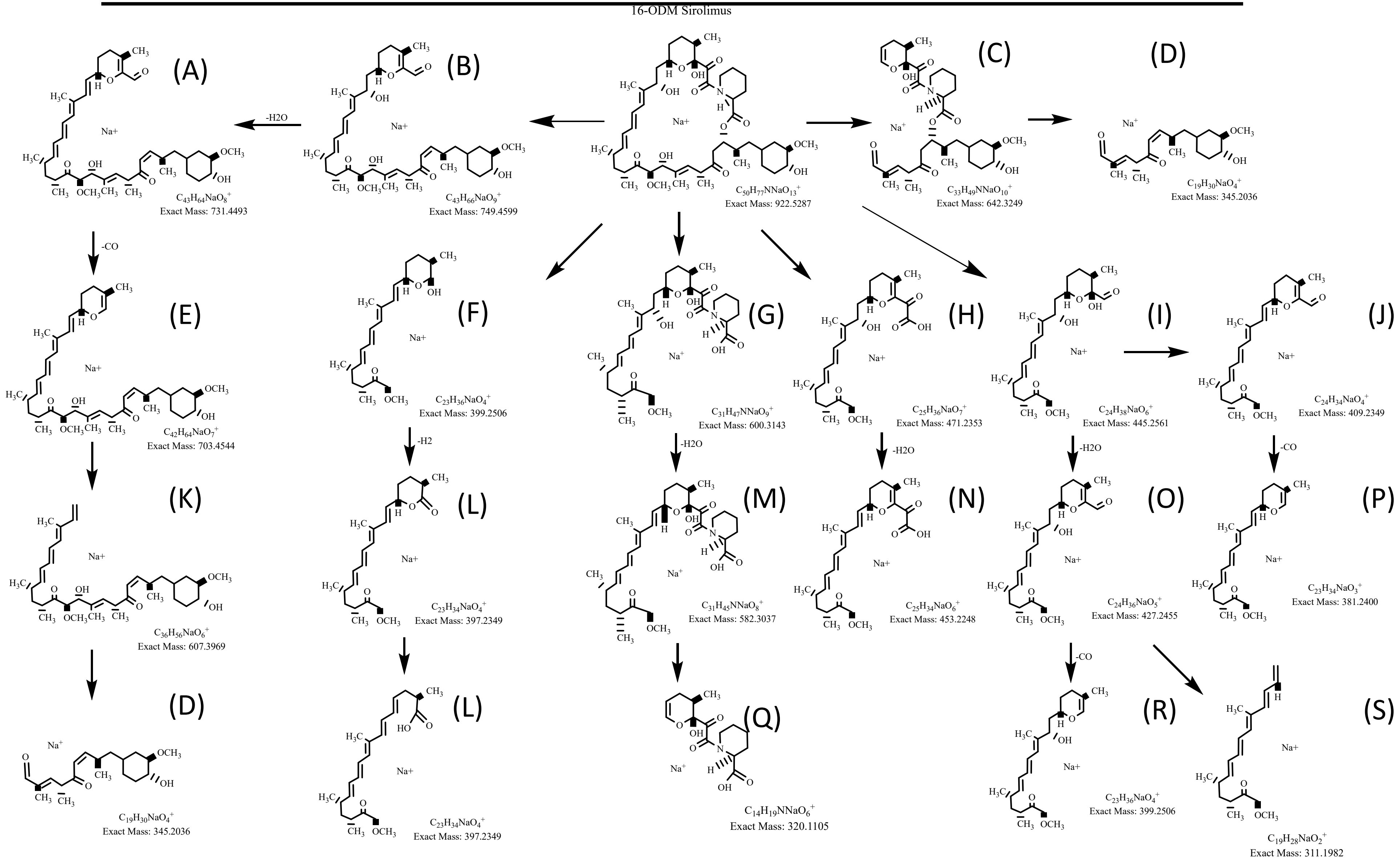

Characteristic Fragments of 16-O-Desmethyl Sirolimus

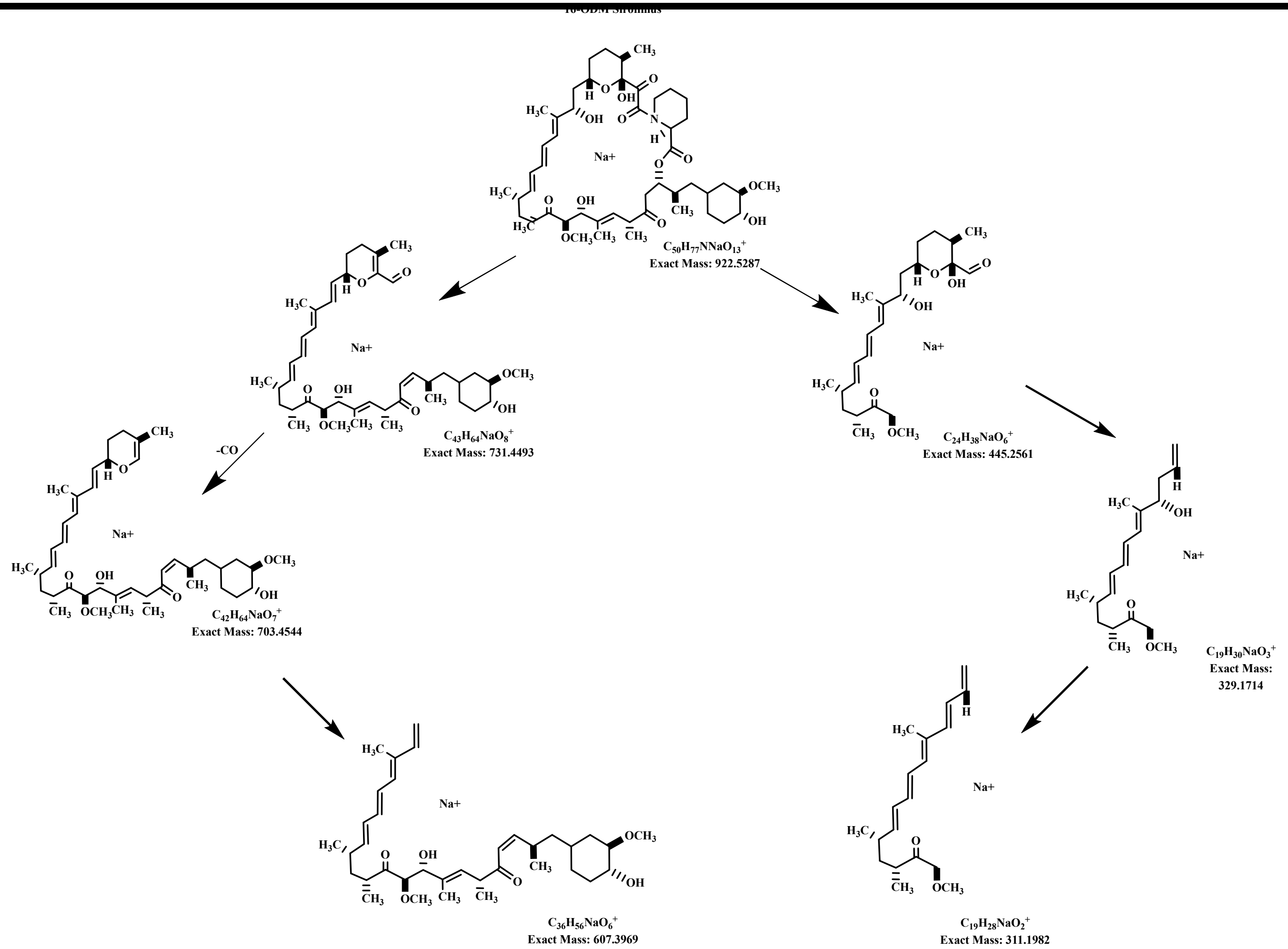

Δppm of 16-O-Desmethyl Sirolimus Fragments

	Theoretical mass	Measured mass	Δppm
**16-ODM Sirolimus**	922.5287	922.5286	0.1
A	731.4493	731.4488	0.7
B	749.4599	749.4594	0.7
C	642.3249	642.3239	1.5
D	345.2036	345.2031	1.5
E	703.4544	703.4538	0.9
F	399.2506	399.2496	2.5
G	600.3143	600.3136	1.2
H	471.2353	471.2343	2.2
I	445.2561	445.2550	2.4
J	409.2349	409.2341	2.0
K	607.3969	607.3964	0.8
L	397.2349	397.2341	2.1
M	582.3037	582.3031	1.1
N	453.2248	453.2239	1.9
O	427.2455	427.2446	2.1
P	381.2400	381.2393	1.9
Q	320.1105	320.1098	2.1
R	399.2506	399.2496	2.5
S	311.1982	311.1973	2.9

Structural confirmation of 16-O-Desmethyl Sirolimus

Fragment assignment	Sirolimus Fragments	16-O-desmethyl Sirolimus Fragments	Comments
A	731.4	731.4	Possible 16-27-39-O-desmethyl
B	763.5	749.5	Possible 16-27-39-O-desmethyl
C	642.3	642.3	Exclude 39-O-desmethyl
D	345.2	345.2	Exclude 39-O-desmethyl
E	703.5	703.5	Confirms 16-O-desmethyl
F	399.3	399.3	Possible 16-27-O-desmethyl
G	614.3	600.3	Possible 16-27-O-desmethyl
H	485.3	471.2	Possible 16-27-O-desmethyl
I	459.3	445.3	Possible 16-27-O-desmethyl
J	409.2	409.2	Possible 16-27-O-desmethyl
K	607.4	607.4	Confirms 16-O-desmethyl
L	397.2	397.2	Possible 16-27-O-desmethyl
M	582.3	582.3	Possible 16-27-O-desmethyl
N	453.2	453.2	Possible 16-27-O-desmethyl
O	427.2	427.2	Possible 16-27-O-desmethyl
P	381.2	381.2	Possible 16-27-O-desmethyl
Q	320.1	320.1	Not conclusive
R	413.3	399.3	Possible 16-27-O-desmethyl
S	311.2	311.2	Confirms 16-O-desmethyl
**Determinant Patterns**		**607.4**<br>**311.2**	**Characteristic fragments of 16-O-desmethyl sirolimus**

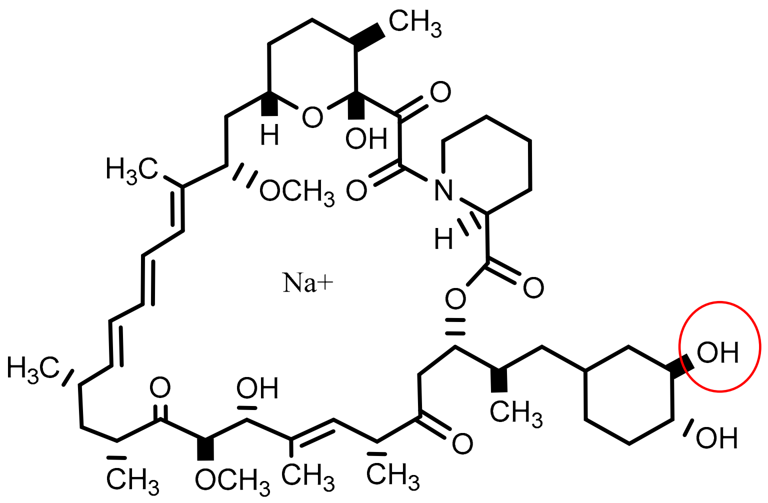

39-O-Desmethyl Sirolimus
$C_{50}H_{77}NNaO_{13}^{+}$
Exact Mass: 922.5287

39-O-Desmethyl Sirolimus (*m/z* = 922.5287)

39-O-Desmethyl Sirolimus Metabolite
Extracted Ion Chromatogram(EIC), (*m/z* = 922.5287)

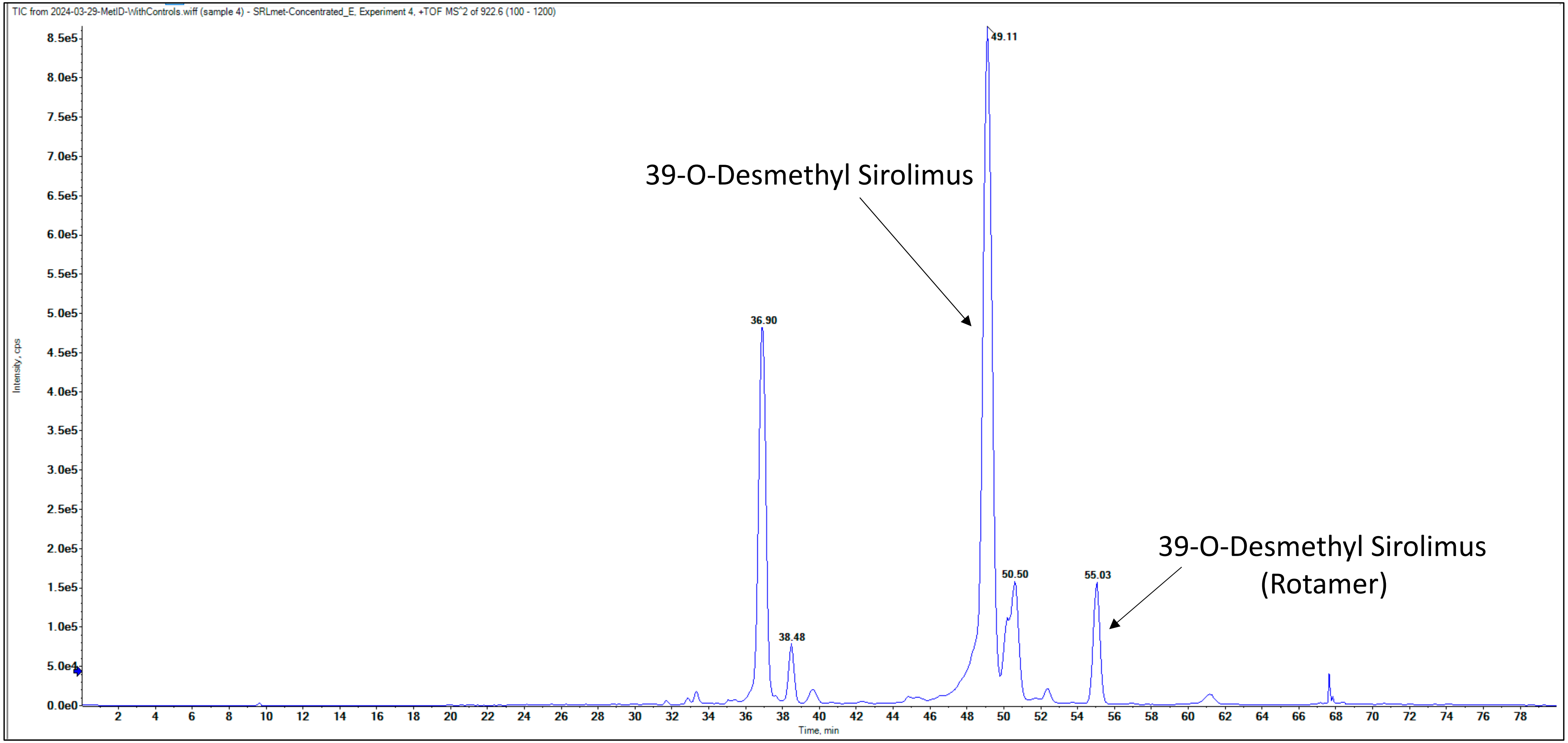

39-Desmethyl Sirolimus Chromatogram (Top)
Mass Spectrum, QTOF Fragmentation, CE=65eV, DP= 110V (Bottom)

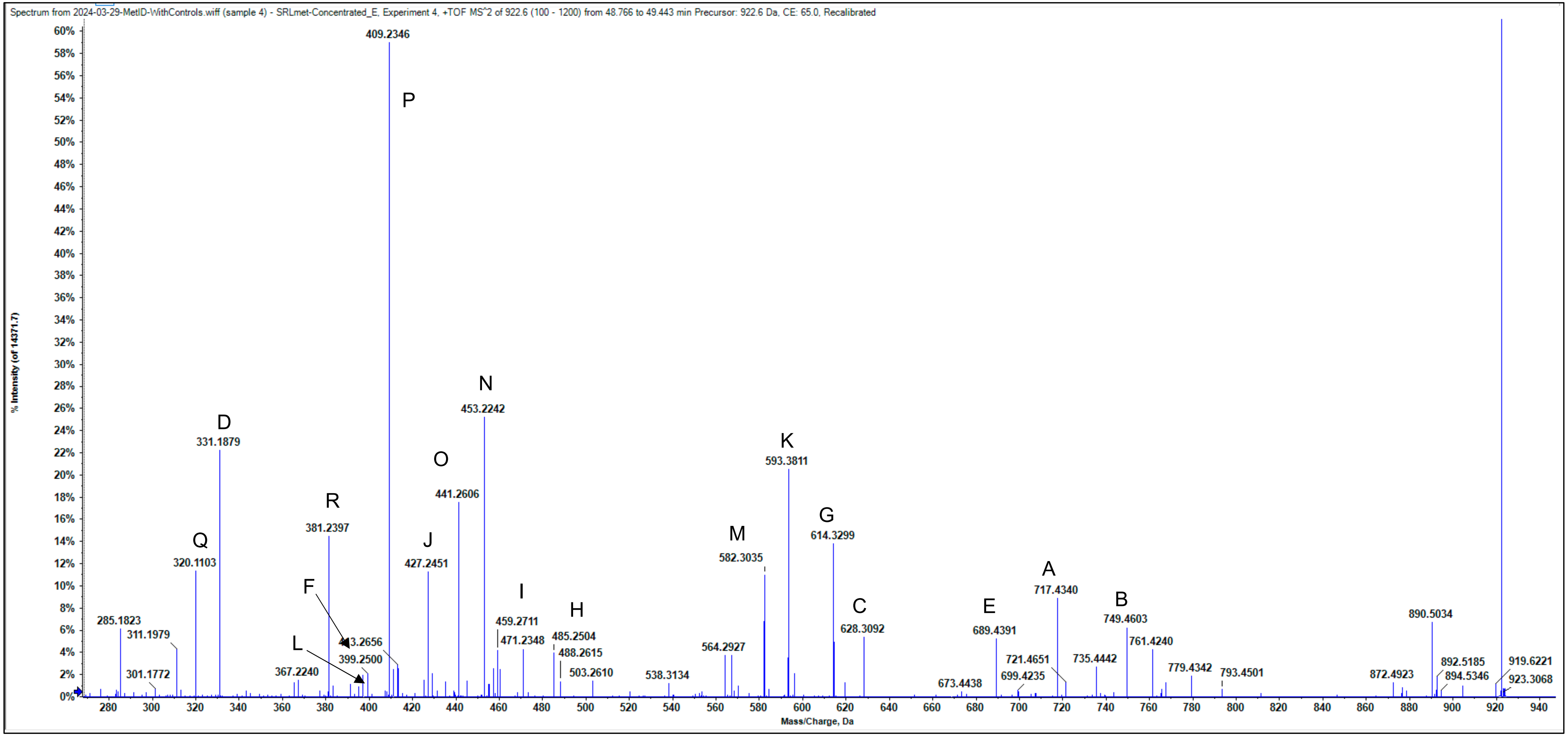

39-Desmethyl Sirolimus Fragmentation Pattern

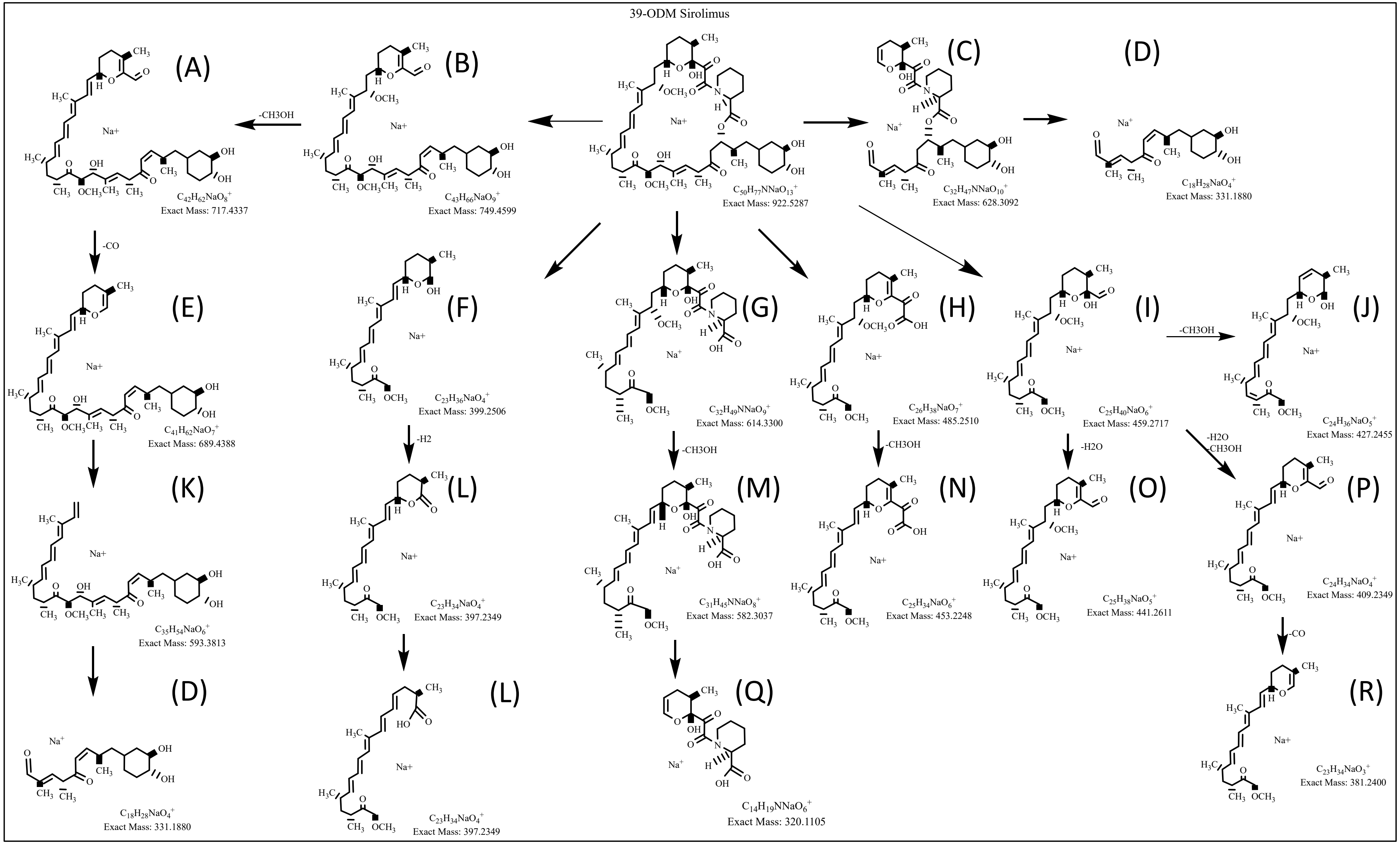

Characteristic Fragments of 39-O-Desmethyl Sirolimus

39-ODM Sirolimus

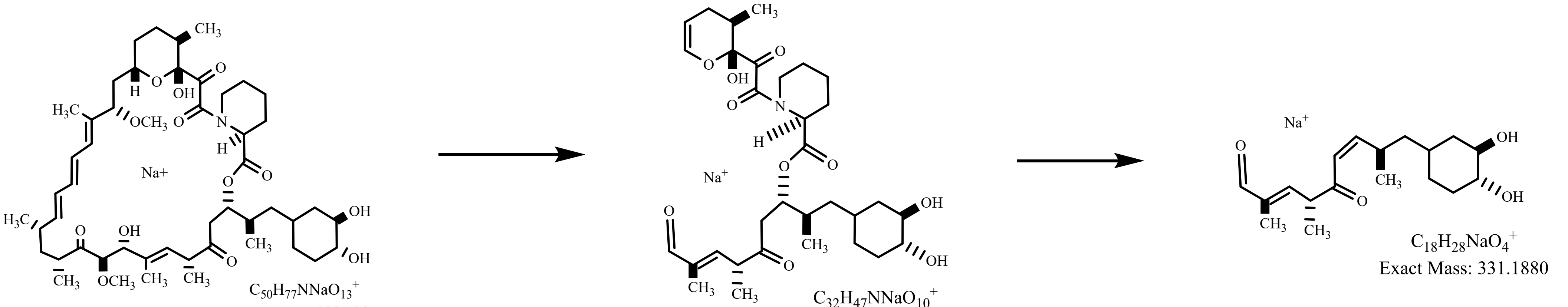

$C_{50}H_{77}NNaO_{13}^{+}$
Exact Mass: 922.5287

$C_{32}H_{47}NNaO_{10}^{+}$
Exact Mass: 628.3092

$C_{18}H_{28}NaO_{4}^{+}$
Exact Mass: 331.1880

Δppm of 39-Desmethyl Sirolimus Fragments

	Theoretical mass	Measured mass	Δppm
**39-ODM Sirolimus**	922.5287	922.5286	0.1
A	717.4337	717.4340	0.4
B	749.4599	749.4603	0.5
C	628.3092	628.3092	0.0
D	331.1880	331.1879	0.2
E	689.4388	689.4391	0.5
F	399.2506	399.2500	1.5
G	614.3300	614.3299	0.1
H	485.2510	485.2504	1.2
I	459.2717	459.2711	1.3
J	427.2455	427.2451	0.9
K	593.3813	593.3811	0.3
L	397.2349	397.2342	1.8
M	582.3037	582.3035	0.4
N	453.2248	453.2242	1.2
O	441.2612	441.2606	1.2
P	409.2349	409.2346	0.8
Q	320.1105	320.1103	0.5
R	381.2400	381.2397	0.8

39-O-Desmethyl Sirolimus Comments

Fragment assignment	Sirolimus	39-ODM	Comment
A	731.4	717.4	Possible 16-27-39-ODM
B	763.5	749.45	Possible 16-27-39-ODM
C	642.3	628.3	Confirms 39-ODM
D	345.2	331.2	Confirms 39-ODM
E	703.5	689.4	Possible 16-27-39-ODM
F	399.3	399.3	Possible 16-27-ODM
G	614.3	614.3	Possible 16-27-ODM
H	485.3	485.3	Possible 16-27-ODM
I	459.3	459.3	Possible 16-27-ODM
J	427.2	427.2	Possible 16-27-ODM
K	607.4	593.3	Possible 16-27-ODM
L	397.2	397.2	Possible 16-27-ODM
M	582.3	582.3	Possible 16-27-ODM
N	453.2	453.2	Possible 16-27-ODM
O	441.3	441.2	Possible 16-27-ODM
P	409.2	409.2	Possible 16-27-ODM
Q	320.1	320.1	Not conclusive
R	381.2	381.2	Possible 16-27-ODM
**Determinant Patterns**		**628.3**	**Characteristic fragments of 39-O-desmethyl sirolimus**
		**331.2**	

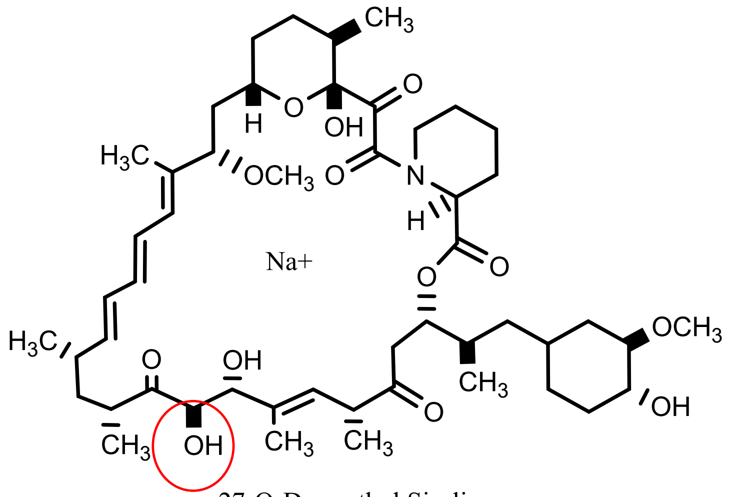

27-O-Desmethyl Sirolimus
$C_{50}H_{77}NNaO_{13}^{+}$
Exact Mass: 922.5287

27-O-Desmethyl Sirolimus (*m/z* = 922.5287)

27-O-Desmethyl Sirolimus Metabolite
Extracted Ion Chromatogram(EIC), (*m/z* = 922.5287)

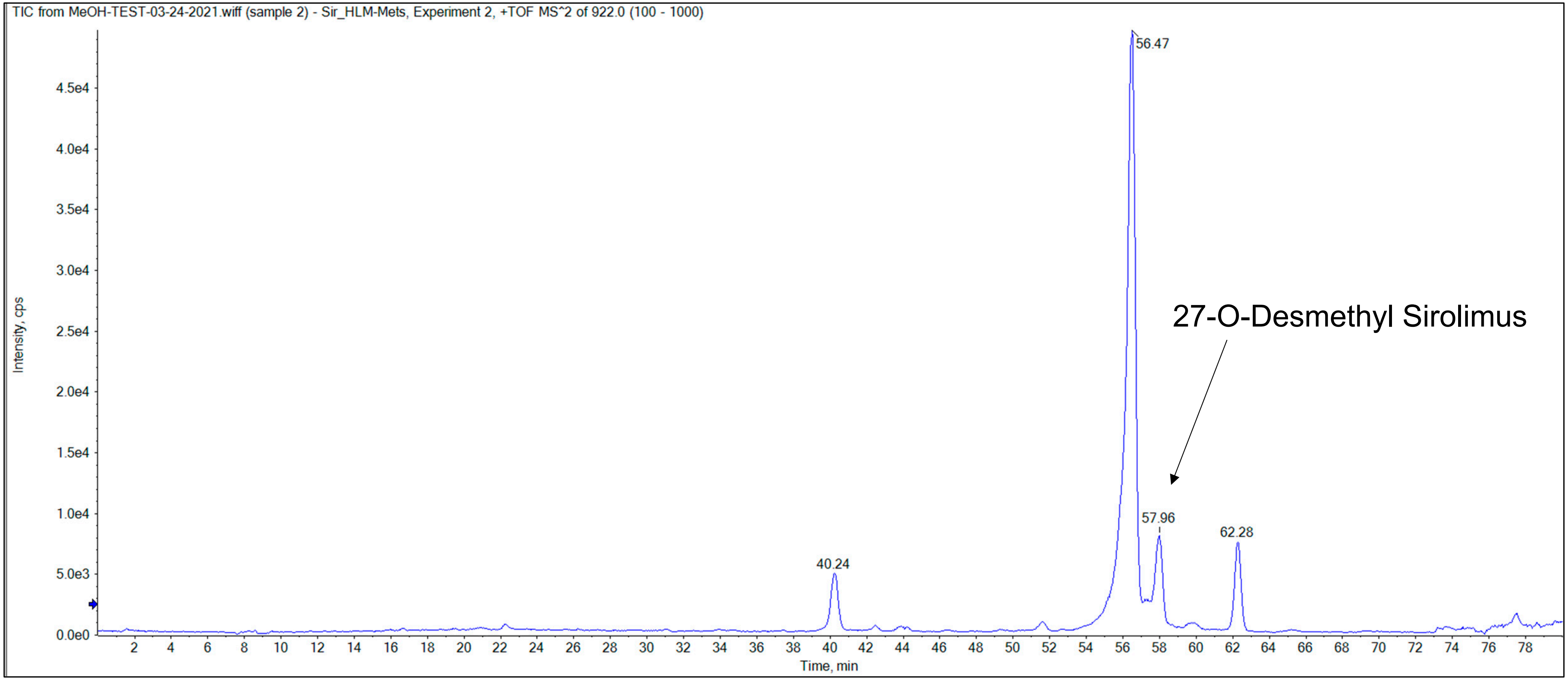

27-Desmethyl Sirolimus Chromatogram (Top)
Mass Spectrum, QTOF Fragmentation, CE=65eV, DP= 110V (Bottom)

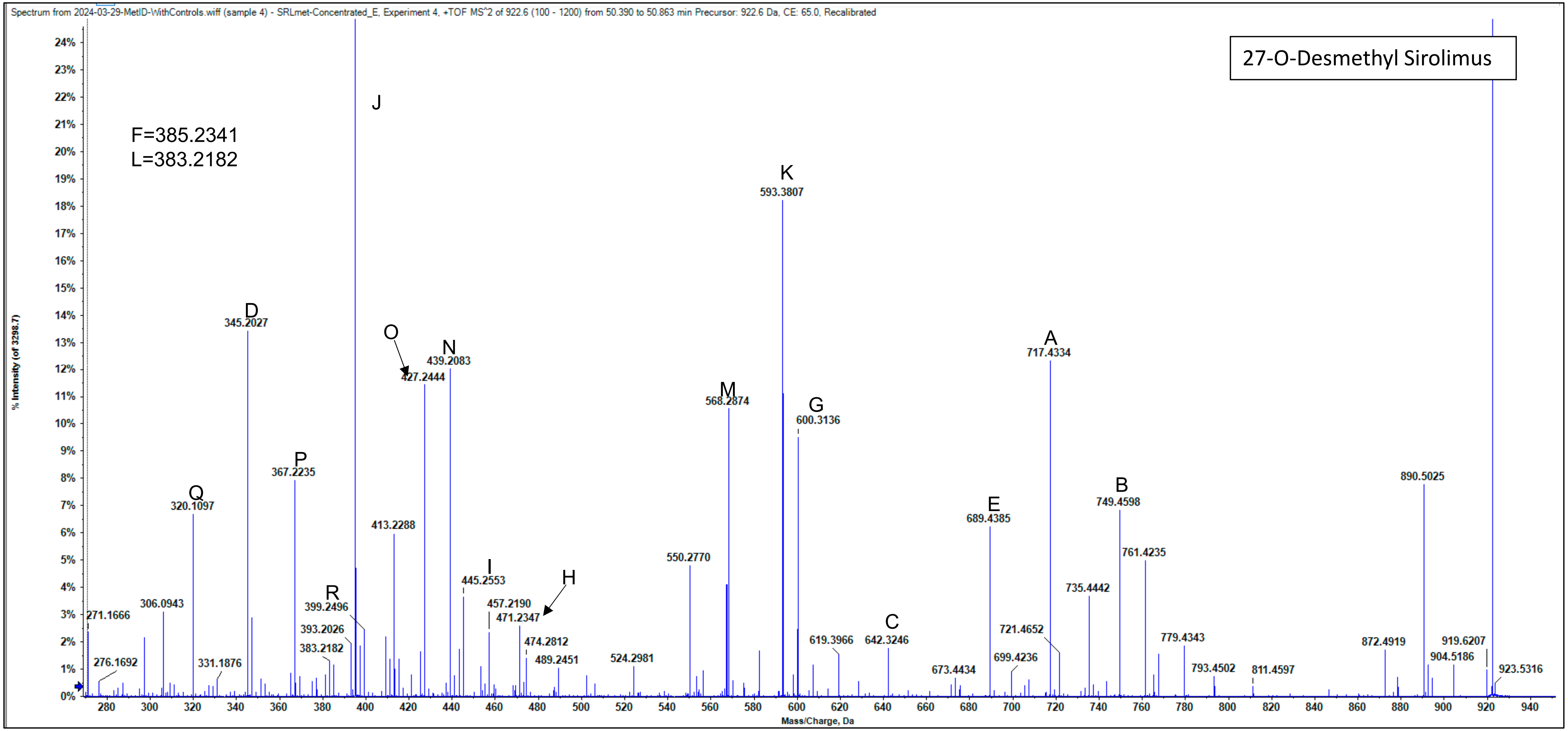

27-ODM Fragmentation Pattern

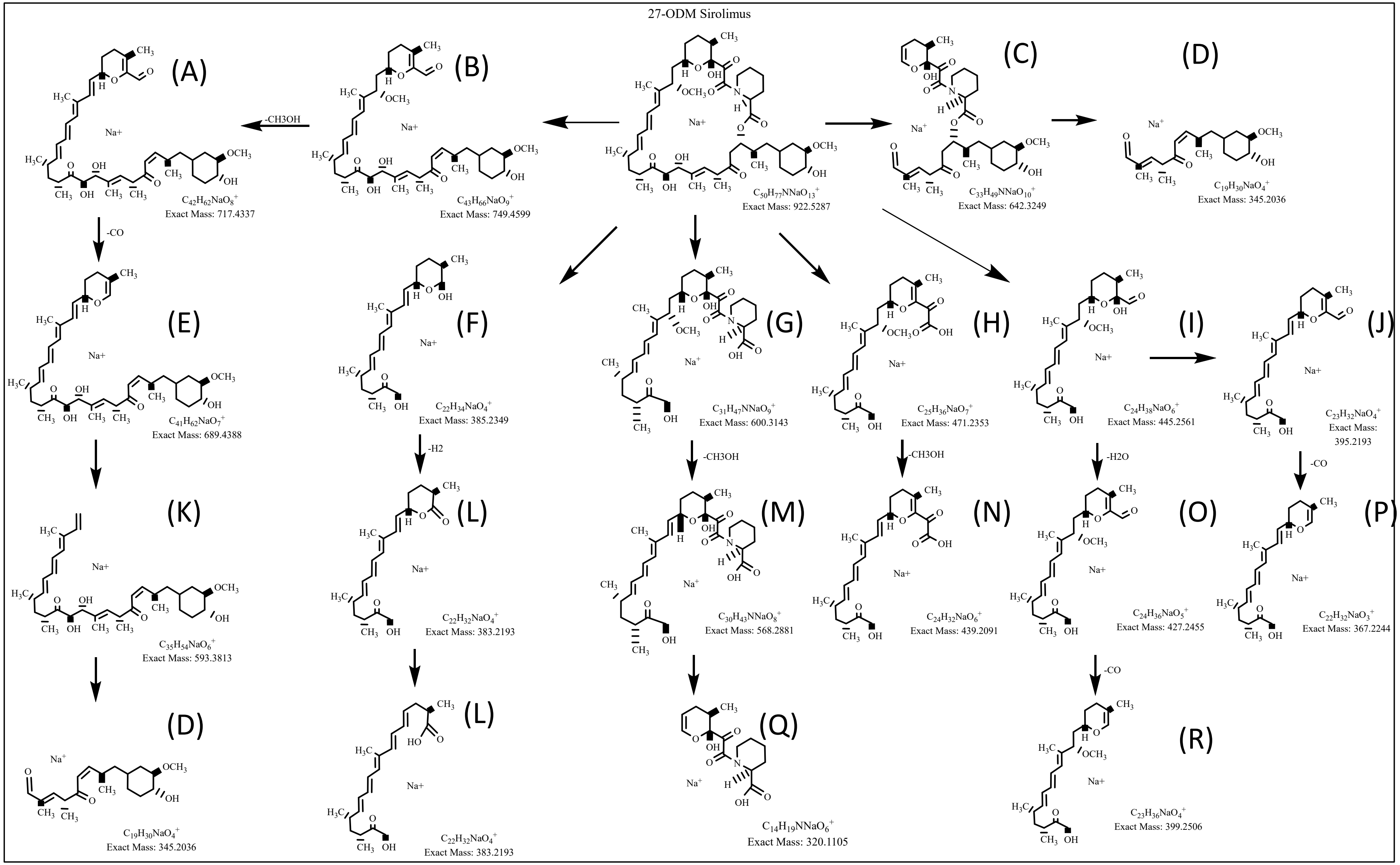

Δppm of 27-ODM Sirolimus Fragments

	Theoretical mass	Measured mass	Δppm
**27-ODM Sirolimus**	922.5287	922.5286	0.1
A	717.4337	717.4334	0.4
B	749.4599	749.4598	0.1
C	642.3249	642.3246	0.4
D	345.2036	345.2027	2.7
E	689.4388	689.4385	0.4
F	385.2349	385.2341	2.1
G	600.3143	600.3136	1.2
H	471.2353	471.2347	1.3
I	445.2561	445.2553	1.7
J	395.2193	395.2185	2.0
K	593.3813	593.3807	0.9
L	383.2193	383.2182	2.8
M	568.2881	568.2874	1.2
N	439.2091	439.2083	1.8
O	427.2455	427.2444	2.6
P	367.2244	367.2235	2.4
Q	320.1105	320.1097	2.4
R	399.2505	399.2496	2.0

27-O-Desmethyl Sirolimus Comments

Fragment assignment	Sirolimus	27-ODM	Comments
A	731.4	717.4	Possible 16-27-39-ODM
B	763.5	749.45	Possible 16-27-39-ODM
C	642.3	642.3	Exclude 39-ODM
D	345.2	345.2	Exclude 39-ODM
E	703.5	689.4	Possible 16-27-39-ODM
F	399.3	385.2	Possible 16-27-ODM
G	614.3	600.3	Possible 16-27-ODM
H	485.3	471.2	Possible 16-27-ODM
I	459.3	445.2	Possible 16-27-ODM
J	409.2	395.2	Confirms 27-ODM
K	607.4	593.3	Confirms 27-ODM
L	397.2	383.2	Possible 16-27-ODM
M	582.3	568.2	Possible 16-27-ODM
N	453.2	439.2	Possible 16-27-ODM
O	441.3	427.2	Possible 16-27-ODM
P	381.2	367.2	Possible 16-27-ODM
Q	320.1	320.1	Not conclusive
R	413.2	399.2	Possible 16-27-ODM
**the presence of 593.3 determines demetylation at position (C27)or (C39), but the characteristic fragment of 39-ODM are not present, therefore it can be concluded that position (C27) is being demethylated**			

Didesmethyl Sirolimus Metabolites

m/z = 908.5131

Didesmethyl Sirolimus Metabolites

Hypothetical, not identified !!

Not detected in *vivo* or *vitro*

Sirolimus metabolism

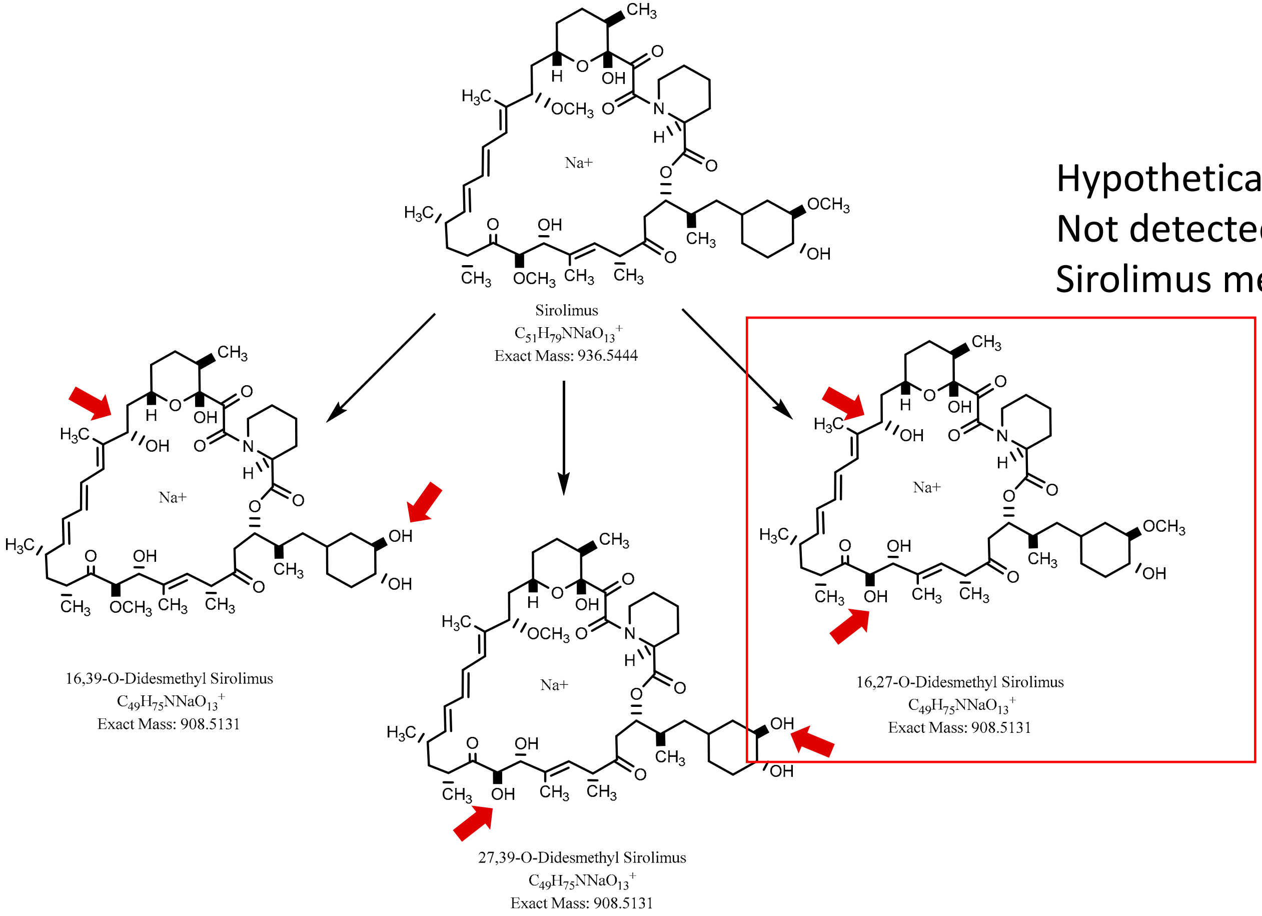

Didesmethyl Sirolimus Metabolites (m/z=908.5131 Extracted)

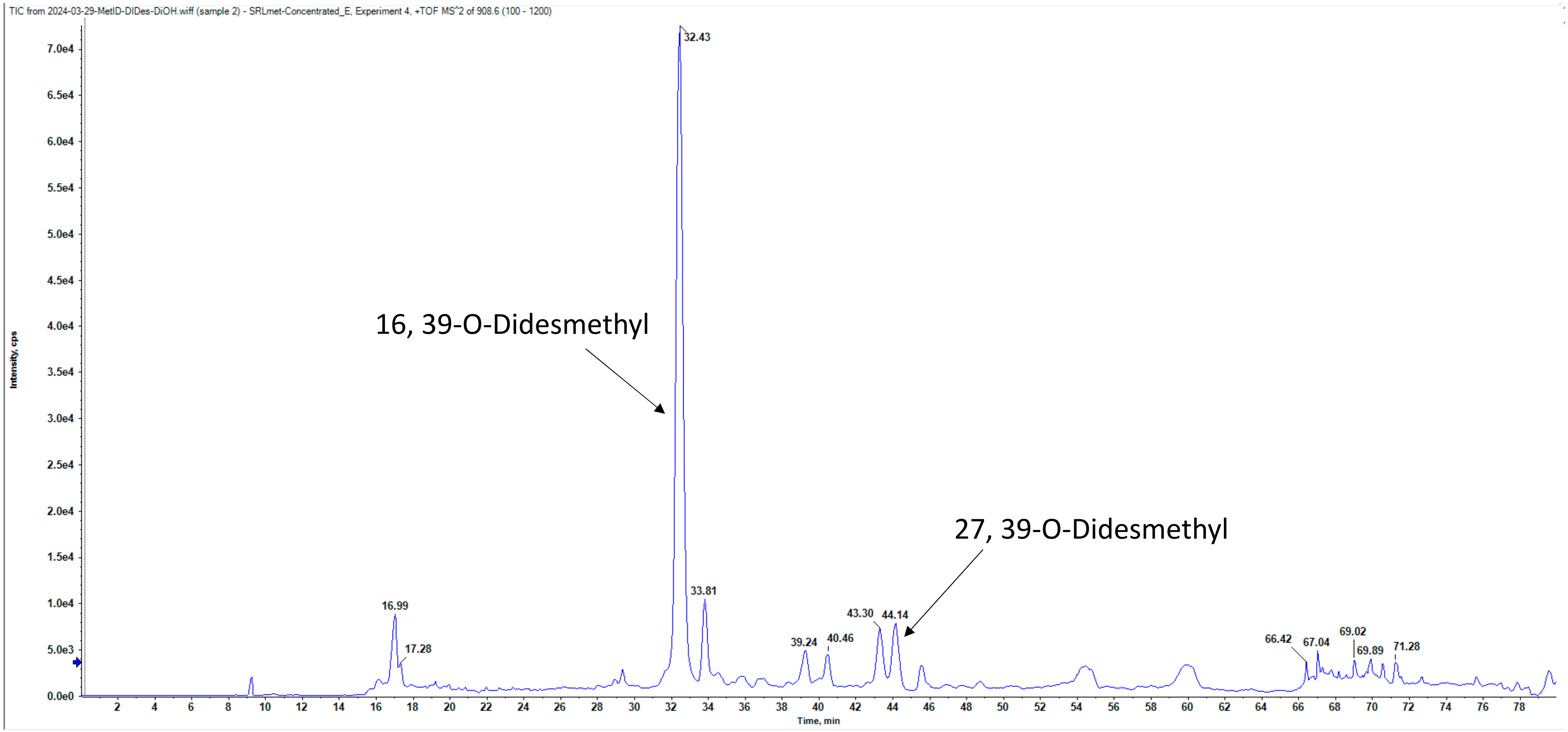

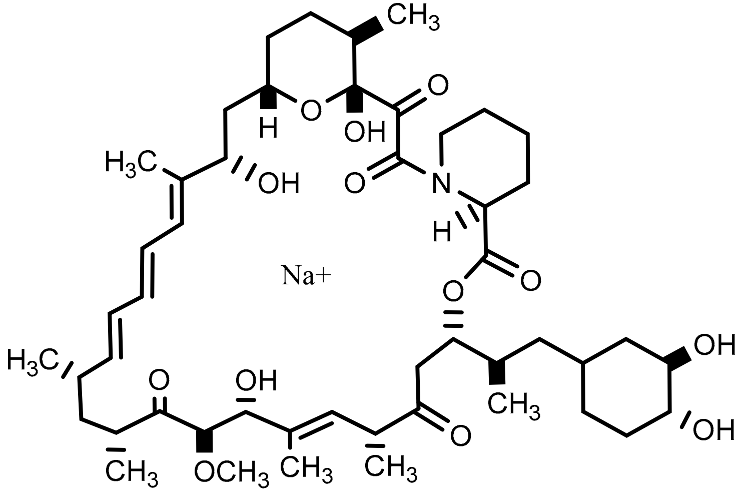

16-O-39-O-Didesmethyl Sirolimus

$C_{49}H_{75}NNaO_{13}^{+}$

Exact Mass: 908.51306

16,39-O-didesmethyl Sirolimus (*m/z* = 908.5131)

16,39-O-Didesmethyl Sirolimus Metabolites
Extracted Ion Chromatogram (EIC), m/z= 908.5131

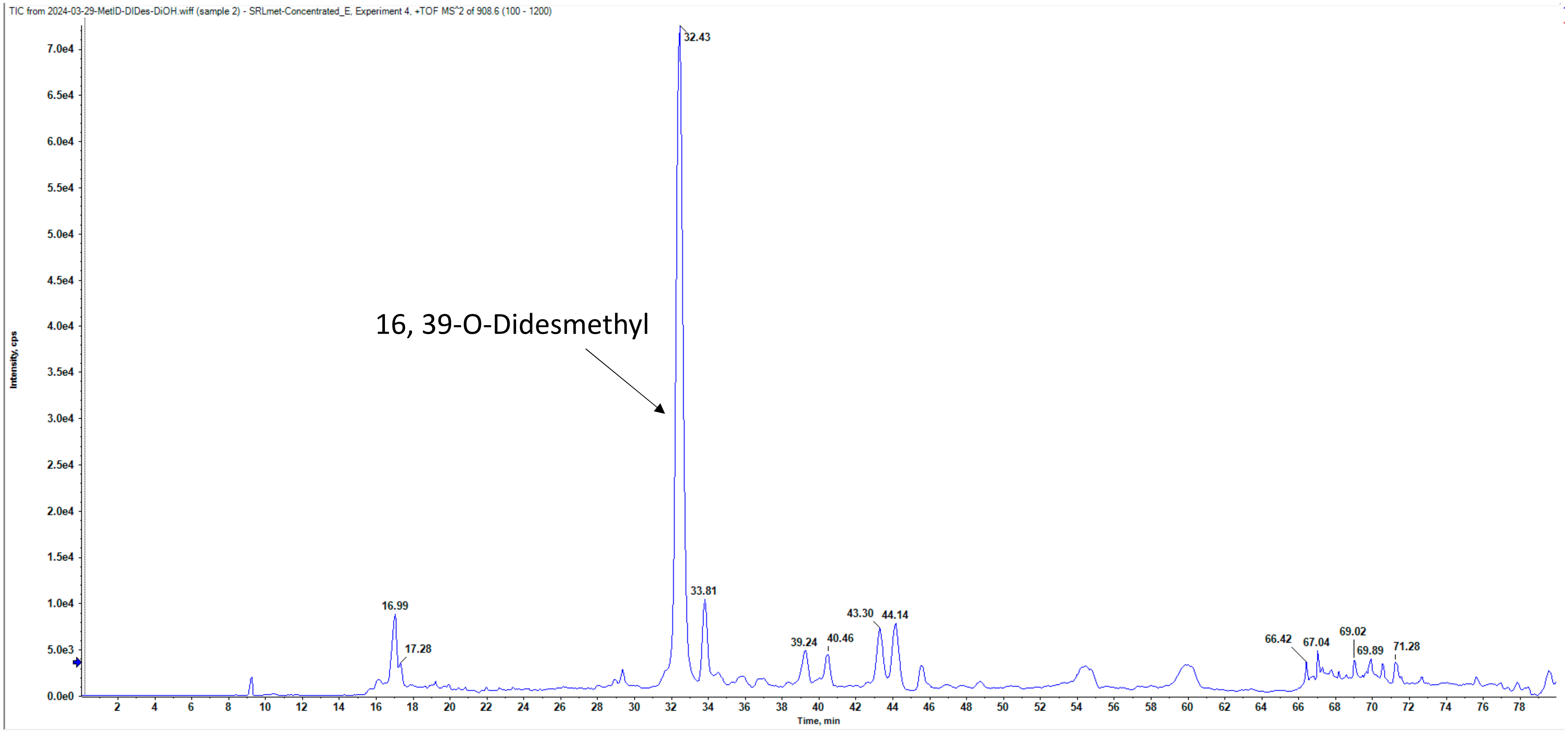

16,39-O-Didesmethyl Sirolimus Chromatogram (Top)
Mass Spectrum, QTOF Fragmentation, (908.5131 Extracted) (Bottom)

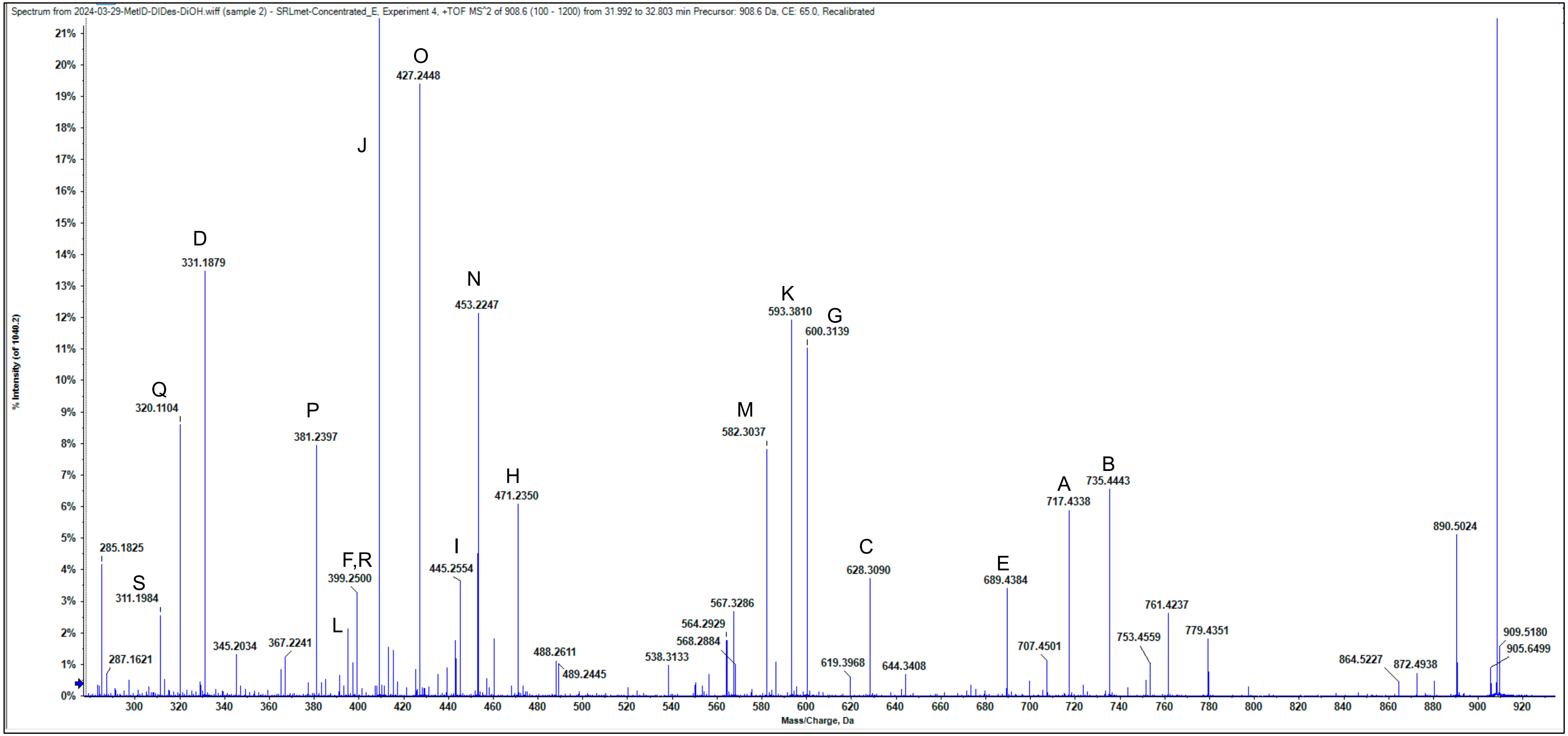

16,39-O-DDM Fragmentation Pattern

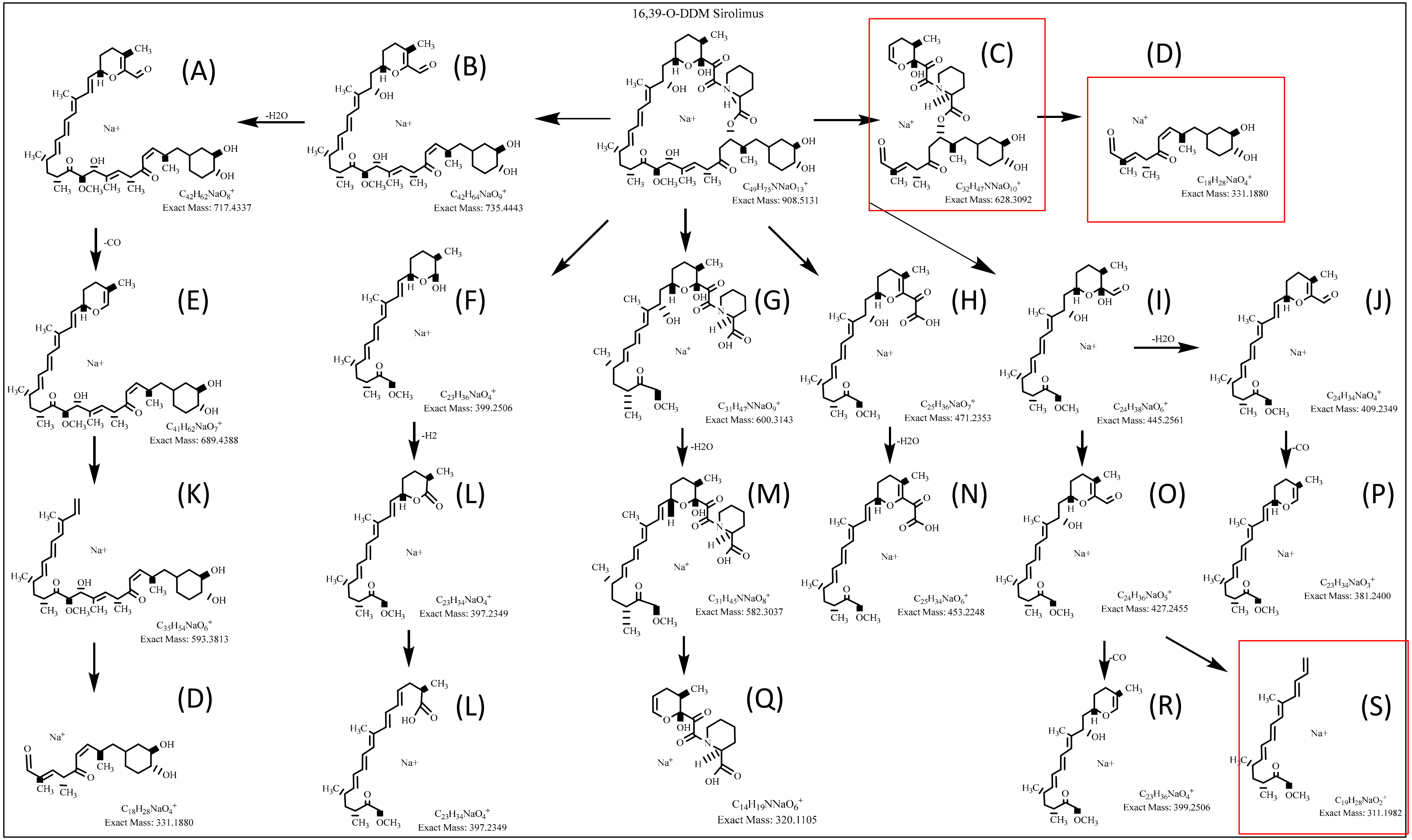

Δppm of 16,39-DDM Sirolimus Fragments

	**Theoretical mass**	**Measured mass**	**Δppm**
**16,39-DDM Sirolimus**	908.5131	908.5131	0.0
A	717.4337	717.4338	0.2
B	735.4443	735.4443	0.1
C	628.3092	628.3090	0.4
D	331.1880	331.1879	0.2
E	689.4388	689.4384	0.6
F	399.2506	399.2500	1.5
G	600.3143	600.3139	0.7
H	471.2353	471.2350	0.7
I	445.2561	445.2554	1.5
J	409.2349	409.2347	0.6
K	593.3813	593.3810	0.4
L	397.2349	397.2357	1.9
M	582.3037	582.3037	0.1
N	453.2248	453.2247	0.1
O	427.2455	427.2448	1.6
P	381.2400	381.2397	0.8
Q	320.1105	320.1104	0.2
R	399.2506	399.2500	1.5
S	311.19820	311.19840	0.6

16,39-O-Didesmethyl Sirolimus Comments

Fragment assignment16,39-DDM	Sirolimus	16,39-DDM Sirolimus	Comment
A	731.4	717.4	Possible 16-27-39-ODM
B	763.5	735.5	Possible 16-27-39-ODM
C	642.3	628.3	Confirms 39-ODM
D	345.2	331.2	Confirms 39-ODM
E	703.5	689.4	Possible 16-27-39 ODM
F	399.3	399.3	Possible 16-27-ODM
G	614.3	600.3	Possible 16-27-ODM
H	485.3	471.2	Possible 16-27-ODM
I	459.3	445.2	Possible 16-27-ODM
J	409.2	409.2	Possible 16-27-ODM
K	607.4	593.3	Possible 27-39-ODM
L	397.2	397.2	Possible 16-27-ODM
M	582.3	582.3	Possible 16-27-ODM
N	453.2	453.2	Possible 16-27-ODM
O	441.3	427.2	Possible 16-27-ODM
P	381.2	381.2	Possible 16-27-ODM
Q	320.1	320.1	Not conclusive
R	399.3	399.3	Possible 16-27-ODM
S	ND	311.1982	Confirms 16-ODM
**Determinant Patterns**		**331.2, 628.3**	**Characteristic fragments of 39-O-desmethyl sirolimus**
		**311**	**Characteristic fragments of 16-O-desmethyl sirolimus**

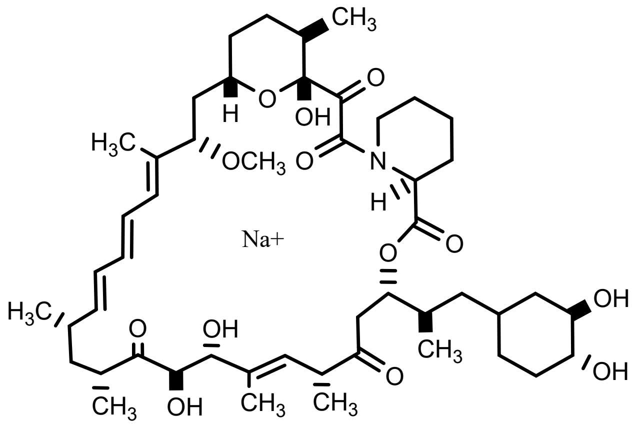

27-O-39-O-Didesmethyl Sirolimus
$C_{49}H_{75}NNaO_{13}^{+}$
Exact Mass: 908.51306

27,39-O-didesmethyl Sirolimus (*m/z* = 908.5131)

27,39-O-Didesmethyl Sirolimus Metabolites
Extracted Ion Chromatogram (EIC), m/z= 908.5131

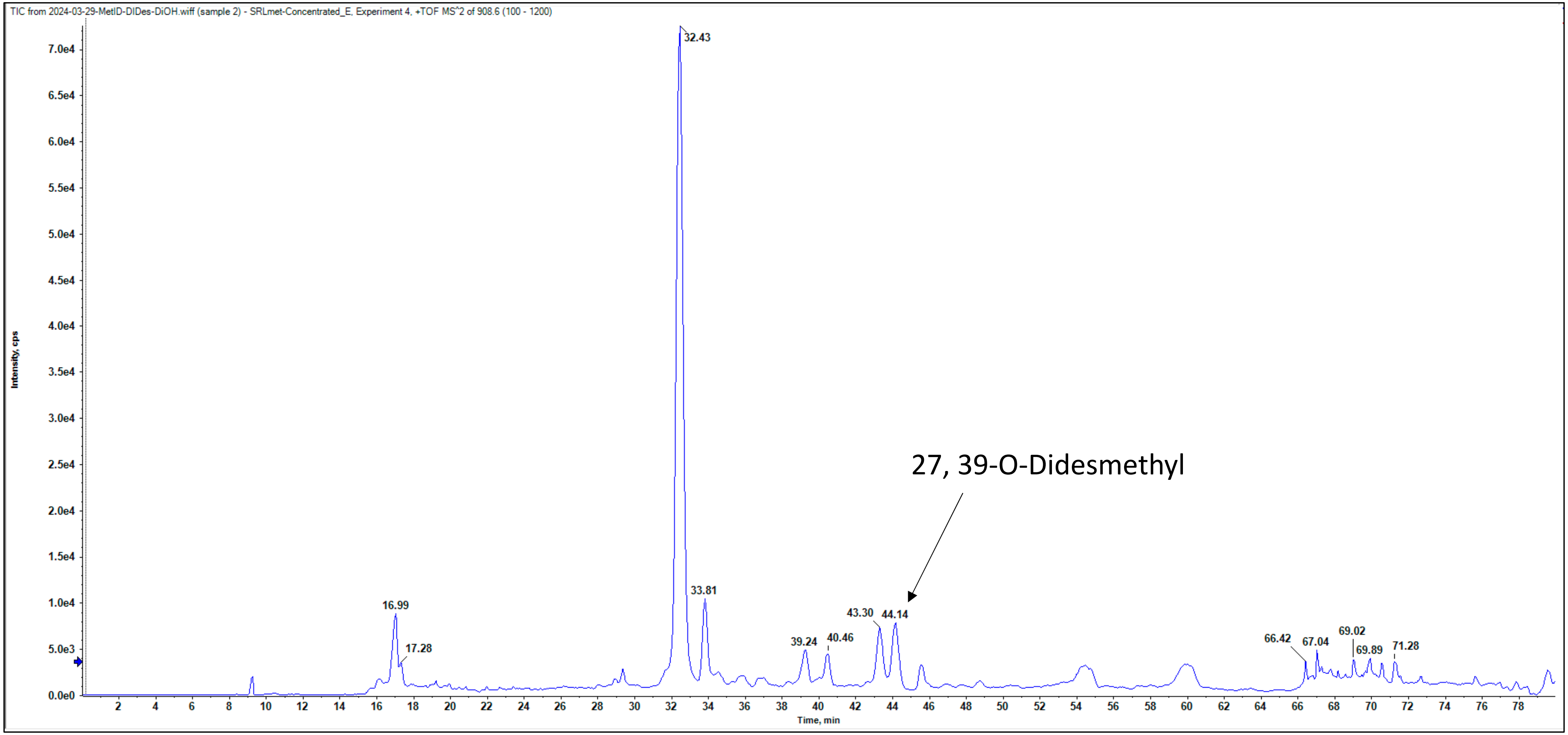

27,39-O-Didesmethyl Sirolimus Chromatogram Mass Spectrum, QTOF Fragmentation, (908.5131 Extracted)

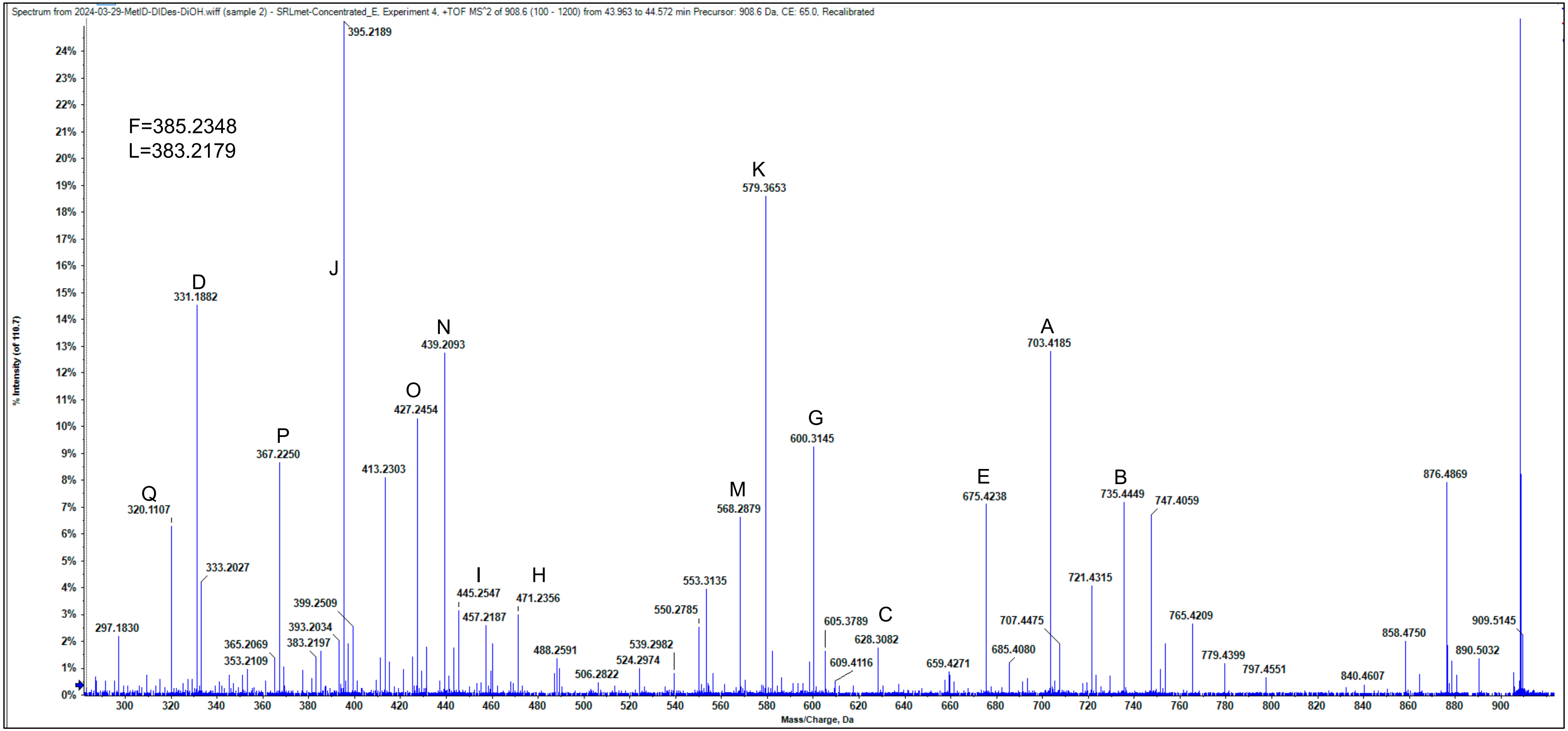

27,39-O-DDM Fragmentation Pattern

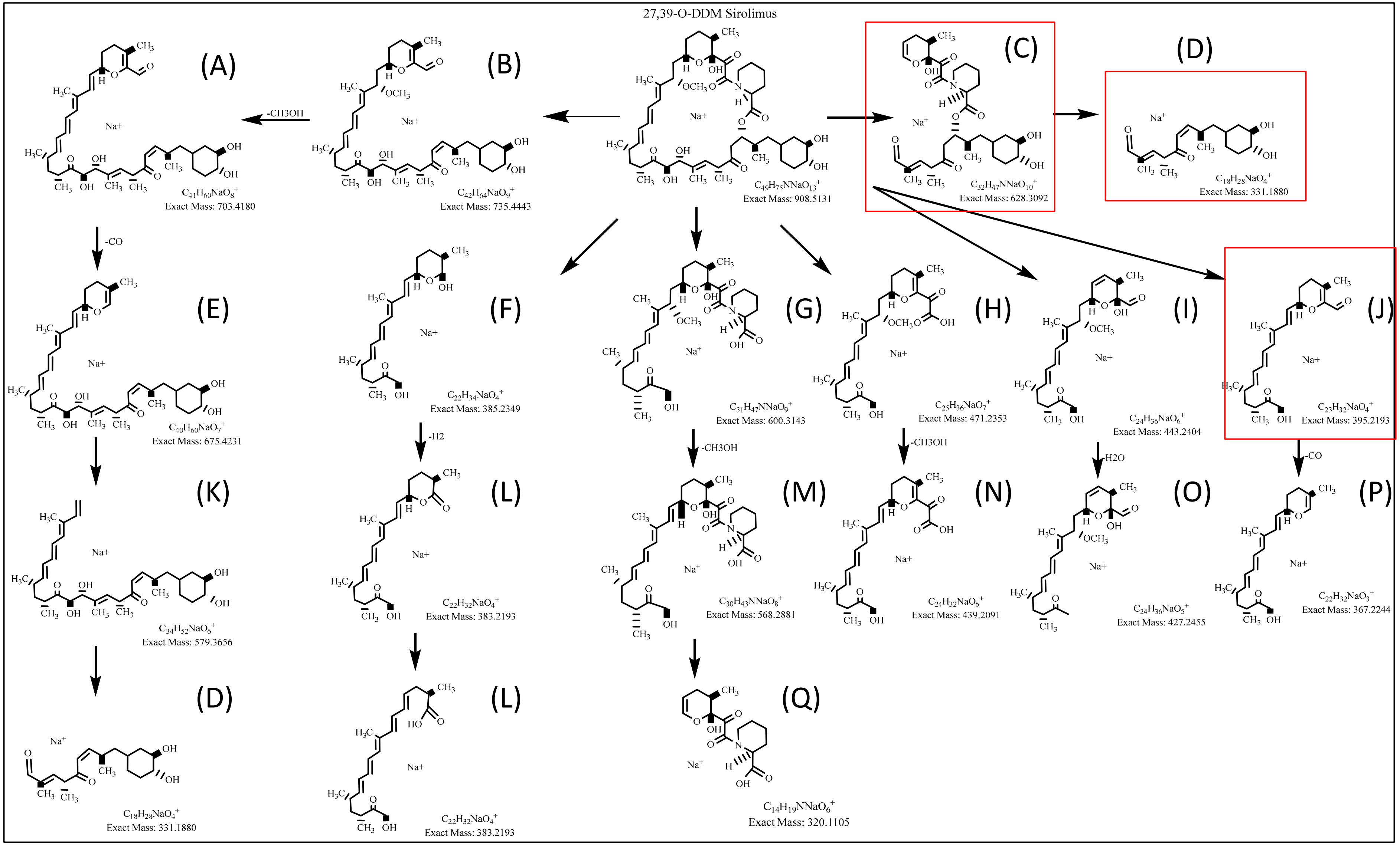

Δppm of 27,39-O-DDM Sirolimus Fragments

	Theoretical mass	Measured mass	Δppm
**27,39-DDM Sirolimus**	908.5131	908.5131	0.0
A	703.4180	703.4185	0.7
B	735.4443	735.4449	0.9
C	628.3092	628.3082	2.1
D	331.1880	331.1882	0.7
E	675.4231	675.4238	1.0
F	385.2349	385.2347	0.6
G	600.3143	600.3145	0.3
H	471.2353	471.2356	0.6
I	443.2404	443.2439	3.1
J	395.2193	395.2189	0.9
K	579.3656	579.3653	0.5
L	383.2193	383.2197	1.1
M	568.2881	568.2879	0.3
N	439.2091	439.2093	0.4
O	427.2455	427.2454	0.2
P	367.2244	367.2250	1.7
Q	320.1105	320.1107	0.7

27,39-O-Didesmethyl Sirolimus Comments

Fragment assignment	Sirolimus	27,39-DDM Sirolimus	Comment
A	731.4	703.4	Possible 16-27-39-ODM
B	763.5	735.5	Possible 16-27-39-ODM
C	642.3	628.3	Confirms 39-ODM
D	345.2	331.2	Confirms 39-ODM
E	703.5	675.5	Possible 16-27-39 ODM
F	399.3	385.2	Possible 16-27-ODM
G	614.3	600.3	Possible 16-27-ODM
H	485.3	471.2	Possible 16-27-ODM
I	459.3	445.2	Possible 16-27-ODM
J	409.2	395.2	Possible 16-27-ODM
K	607.4	579.3	Possible 27-39-ODM
L	397.2	383.2	Possible 16-27-ODM
M	582.3	568.2	Possible 16-27-ODM
N	453.2	439.2	Possible 16-27-ODM
O	441.3	427.2	Possible 16-27-ODM
P	381.2	367.2	Possible 16-27-ODM
Q	320.1	320.1	Not conclusive
**Determinant Patterns**		**331.2, 628.3, 367.2**	**Determinant Patterns for 39-ODM and 27-ODM**
			**16-ODM Characteristic fragments are not present**

Hydroxy Sirolimus Metabolites

m/z = 952.5393

Hydroxy Sirolimus Metabolites Structure, m/z=952

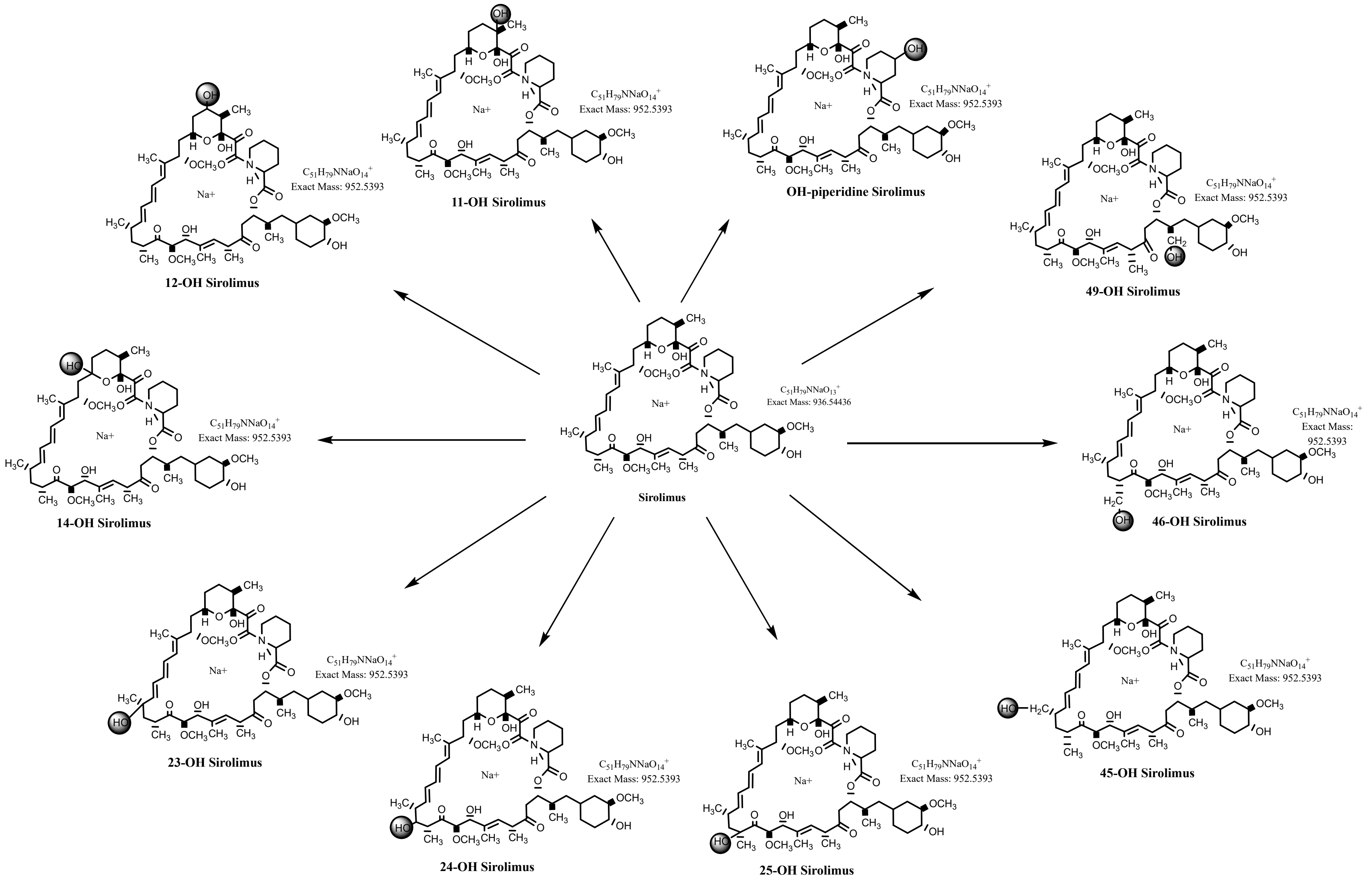

Hydroxy Sirolimus Metabolites
Total Ion Chromatogram, *m/z* = 952.0

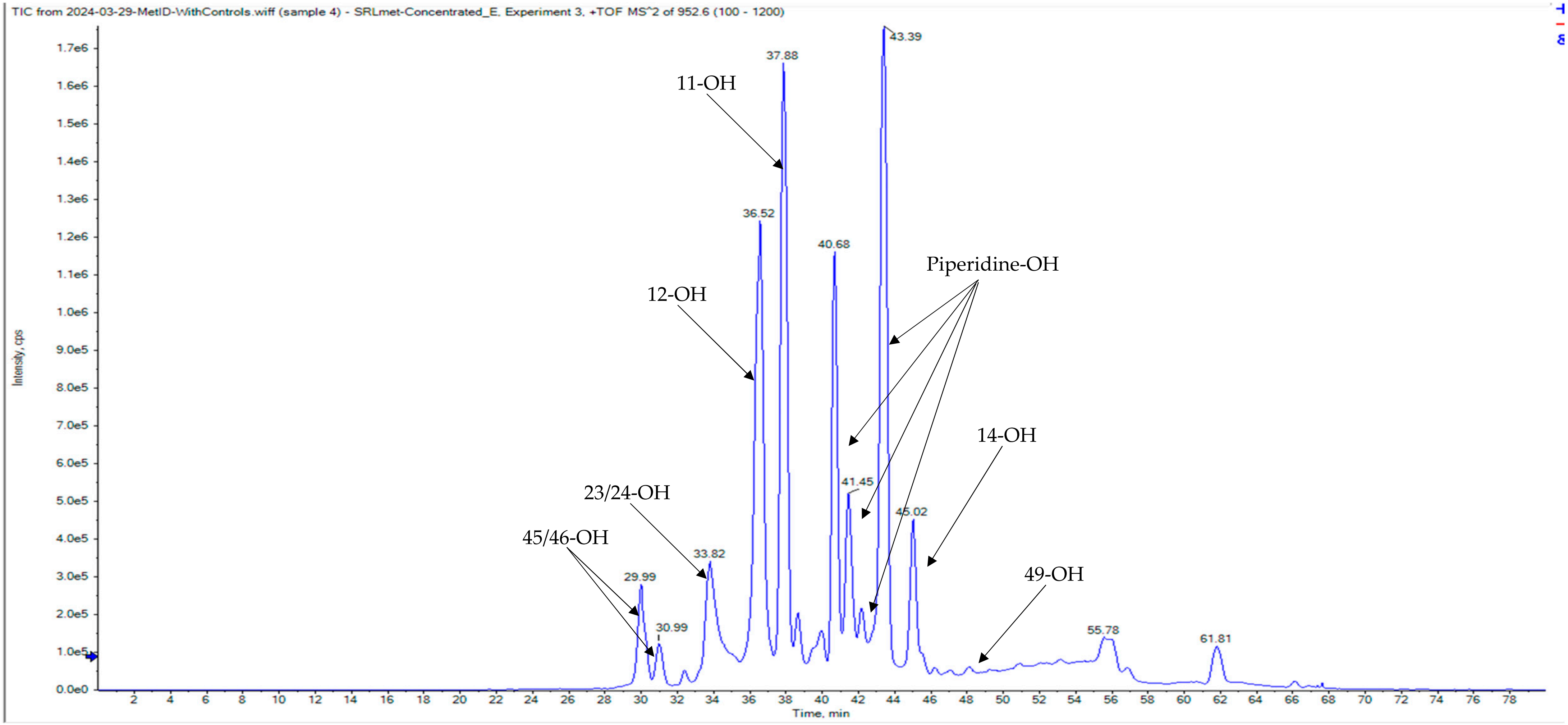

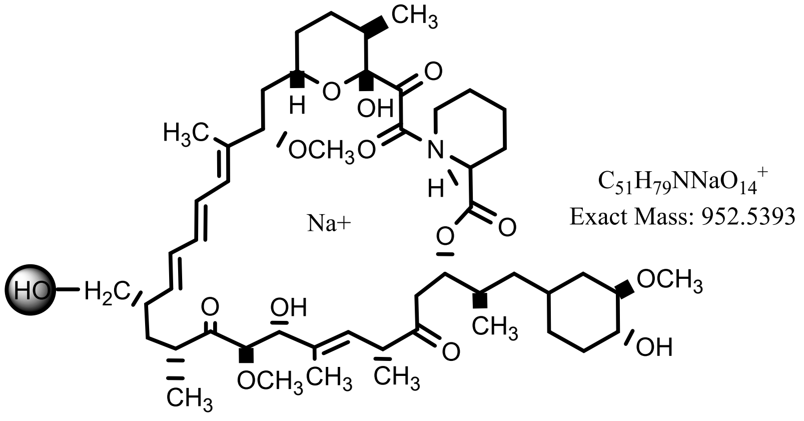

45-OH Sirolimus

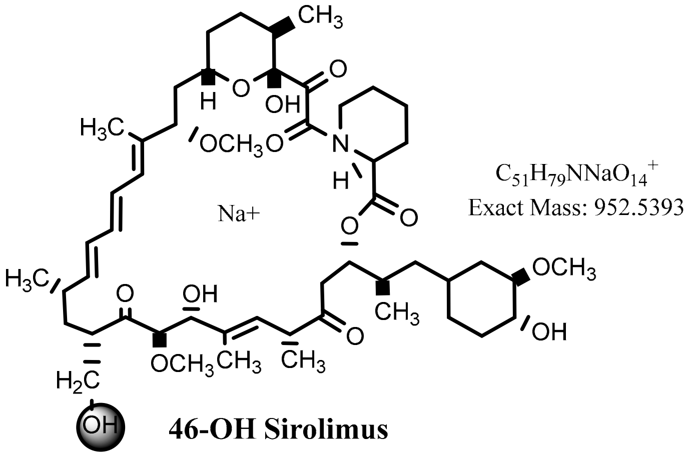

46-OH Sirolimus

45/46-Hydroxy Sirolimus (*m/z* = 952.5393)

Hydroxy Sirolimus Metabolites
Total Ion Chromatogram, *m/z* = 952.0

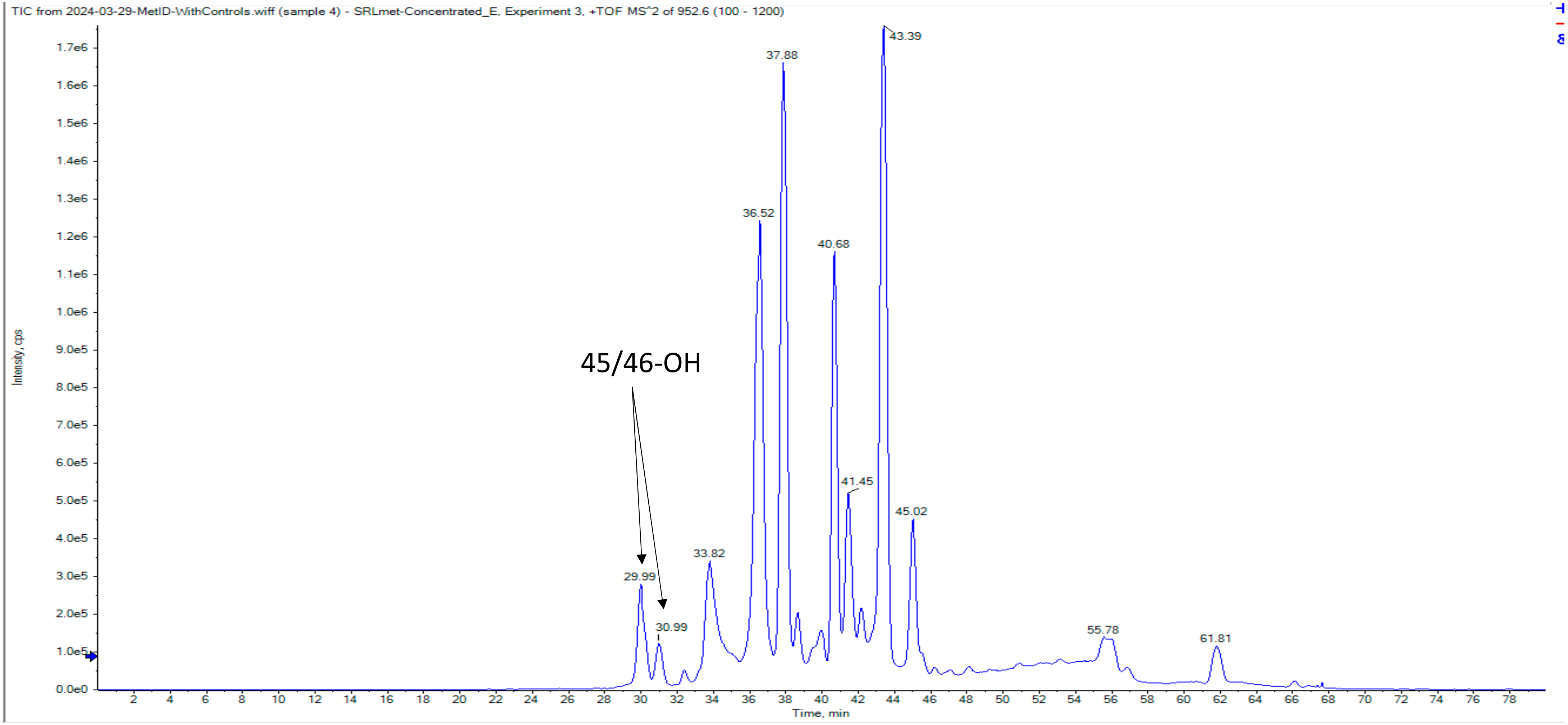

MS spectrum of 45- and 46-Hydroxy Sirolimus CE=65 eV, DP = 110 V

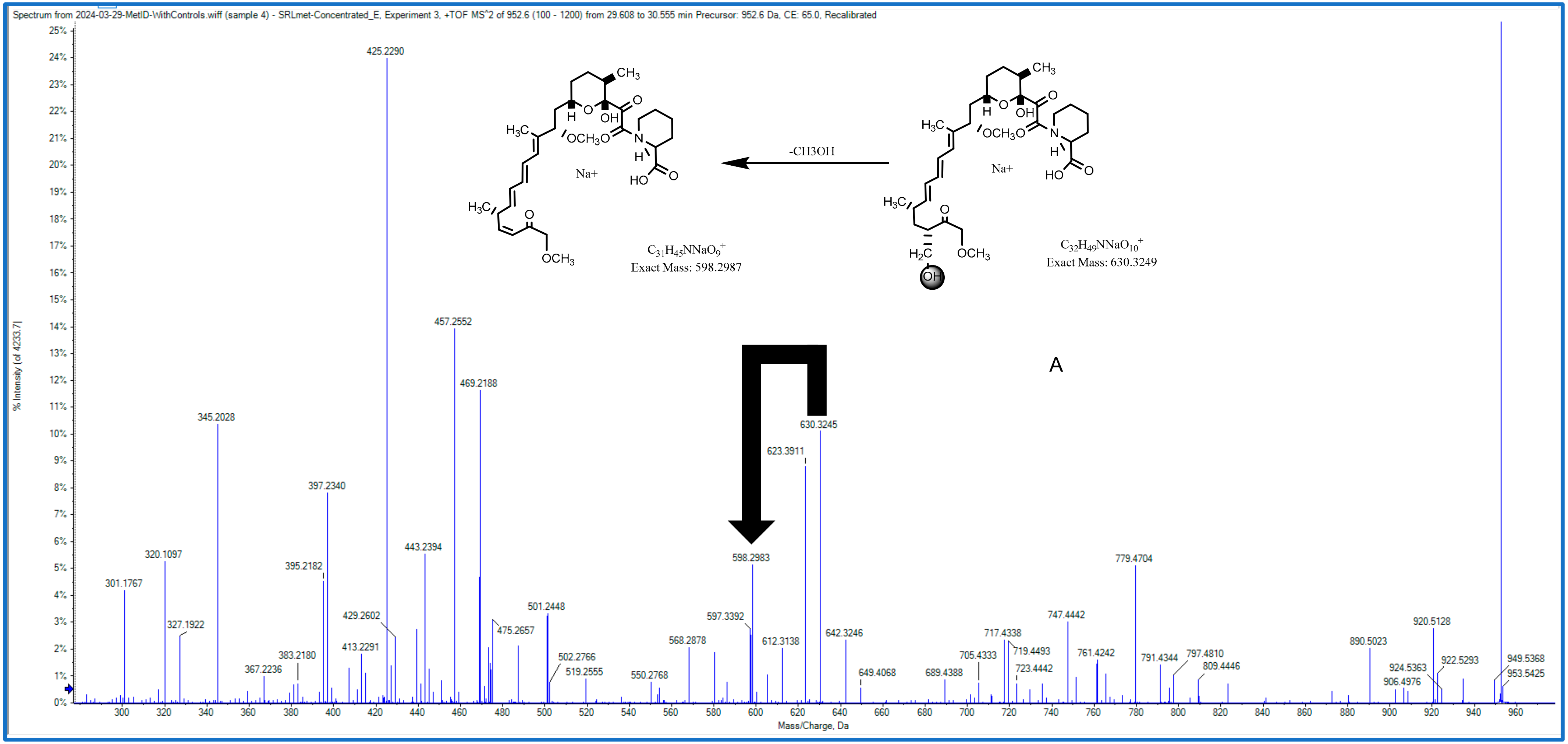

Δppm of 45- and 46-OH Sirolimus Fragments

Fragments	Theoretical mass	Measured mass	Dppm
**45/46-OHSirolimus**	952.5393	952.5393	0.0
A	747.4443	747.4442	0.1
B	779.4705	779.4704	0.1
C	642.3249	642.3246	0.5
D	345.2036	345.2028	2.3
E	719.4493	719.4493	0.0
F	415.2455	415.2448	1.7
G	630.3249	630.3245	0.4
H	501.2459	501.2448	2.0
I	475.2666	475.2657	1.9
J	425.2299	425.2290	2.0
K	623.3918	623.3911	1.2
L	413.2299	413.2291	1.8
M	598.2987	598.2983	0.6
N	469.2197	469.2188	1.6
O	457.2561	457.2552	1.9
P	397.2349	397.2340	0.9
Q	320.1105	320.1097	2.4
R	429.2611	429.2602	2.1

45-Hydroxy Sirolimus Fragmentation Pattern

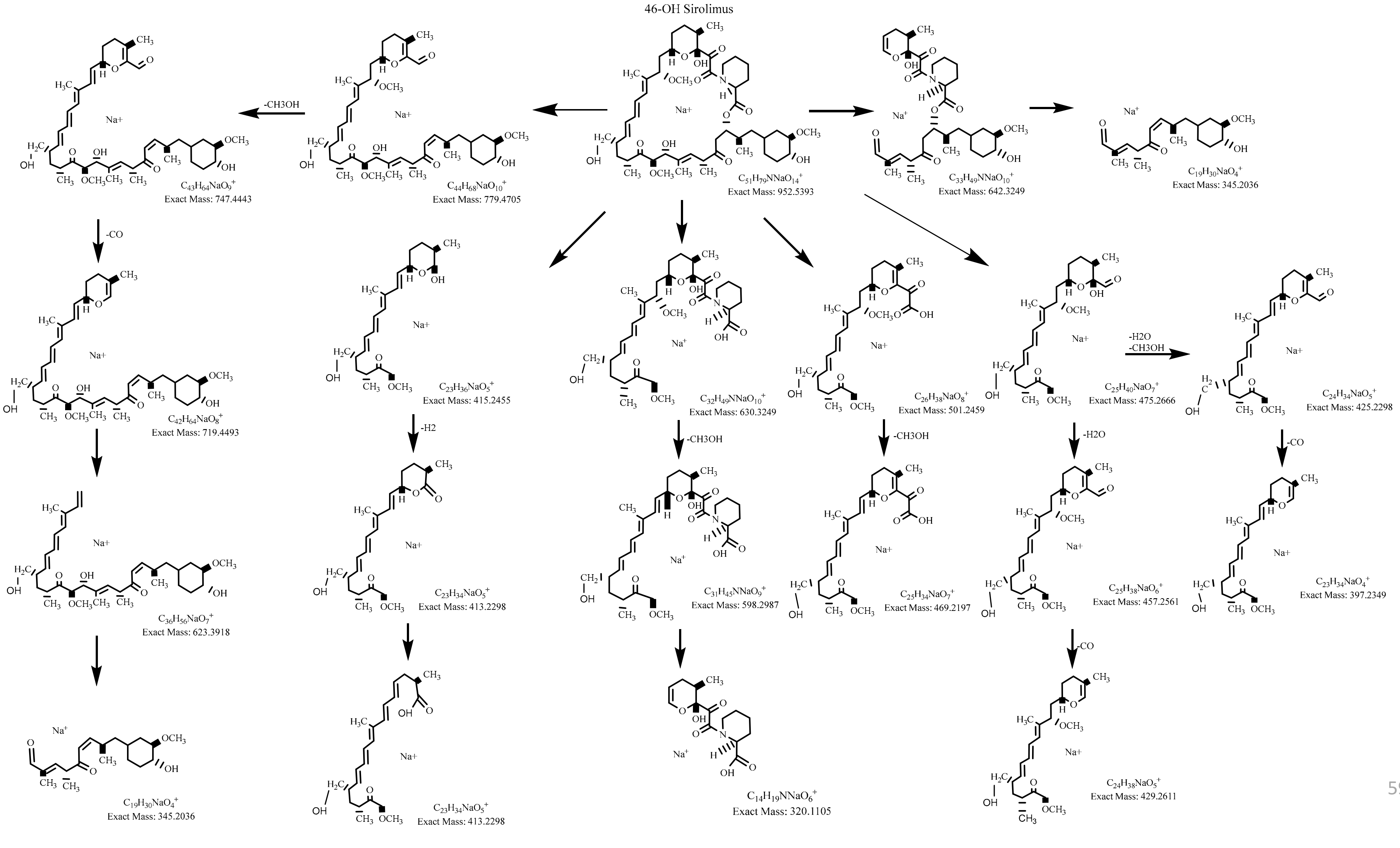

46-Hydroxy Sirolimus Fragmentation Pattern

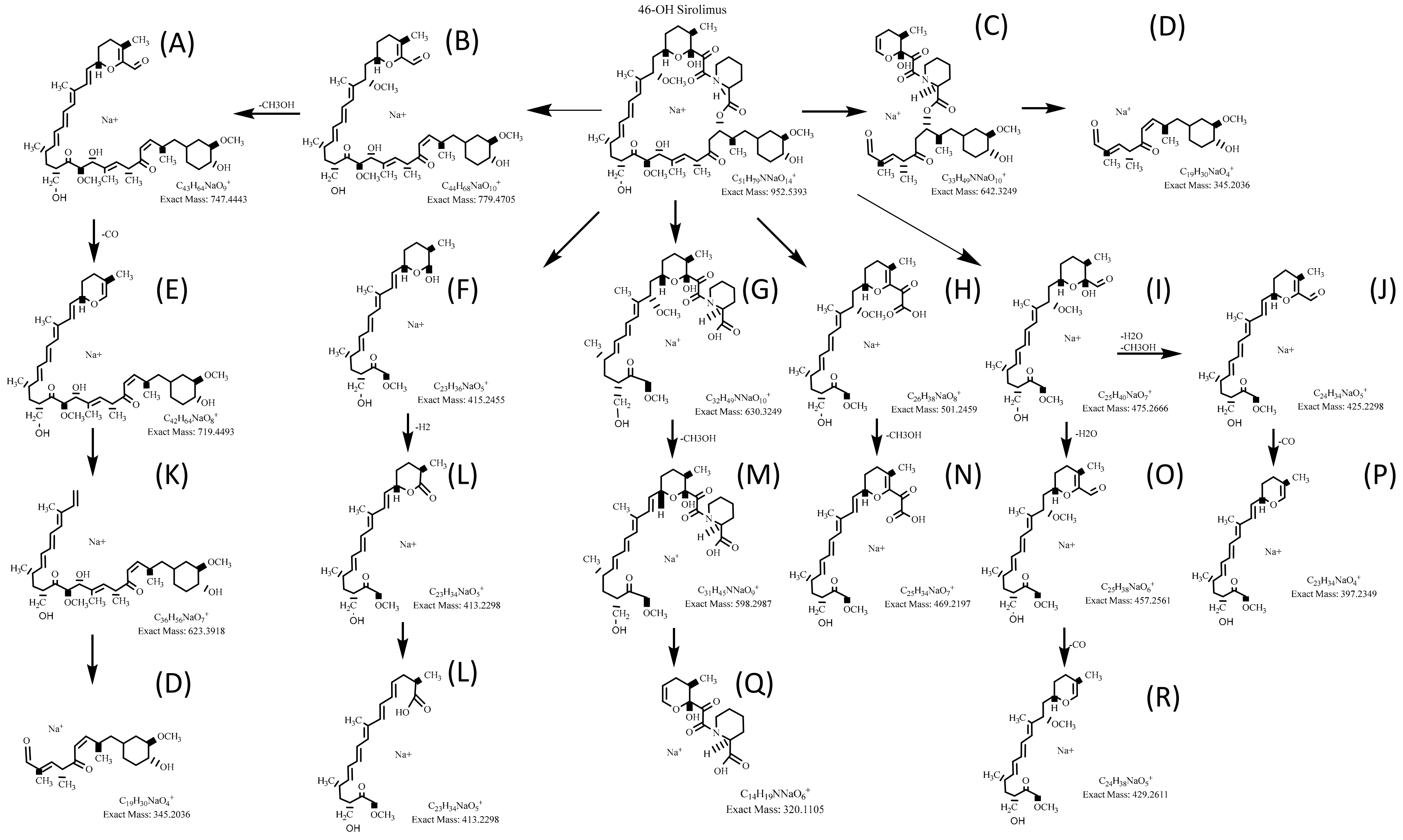

45/**46**-Hydroxy Sirolimus Comments

Assinment Fragment	Sirolimus	45/46-OHSirolimus	Comments	
**45/46-OHSirolimus**	936.5	952.5	Methanol Loss	
A	731.4	747.4		Excludes Piperidine-OH
B	763.5	779.5		Excludes Piperidine-OH
C	642.3	642.3		Possible 11,12,14,49, Piperidine-OH
D	345.2	345.2		Excludes 49-OH
E	703.5	719.4		Excludes Piperidine-OH
F	399.3	415.2		Excludes 49, Piperidine-OH
G	614.3	630.3	598.3	Excludes 49-OH
H	485.2	501.2	469.3	Excludes 49, Piperidine-OH
I	459.3	475.3		Excludes 49, Piperidine-OH
J	409.2	425.2		Excludes 49, Piperidine-OH
K	607.4	623.4		Excludes 11,12,14, Piperidine-OH
L	397.2	413.2		Excludes 49, Piperidine-OH
M	582.3	598.3		Excludes 49-OH
N	453.2	469.2		Excludes 49, Piperidine-OH
O	441.3	457.3	425.3	Excludes 49, Piperidine-OH
P	381.2	397.2		Excludes 49, Piperidine-OH
Q	320.1	320.1		Excludes 11,12,14, Piperidine-OH
R	413.3	429.3		Excludes 49, Piperidine-OH
**characteristic fragments**				**630-32=598, 457-32=425, 501-32=469**
**Determinant patterns**		methanol loss		**598.3 Fragment and other MeOH losses; indicative of 46-OH**

23/24-Hydroxy Sirolimus (*m/z* = 952.5393)

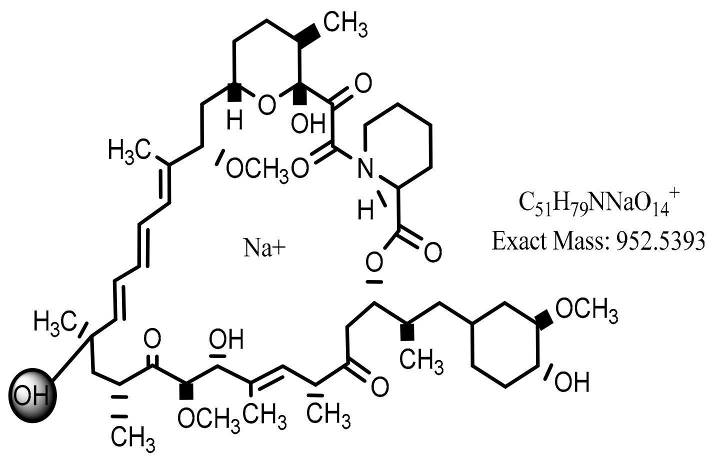

23-OH Sirolimus

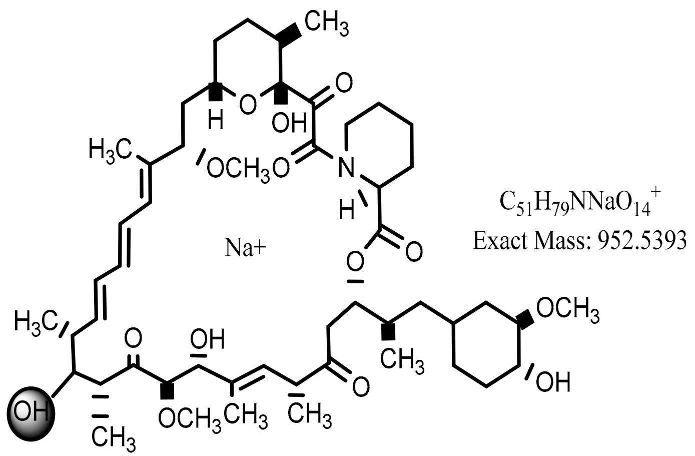

24-OH Sirolimus

23/24-Hydroxy Sirolimus (*m/z* = 952.5393)

Hydroxy Sirolimus Metabolites
Total Ion Chromatogram, *m/z* = 952.0

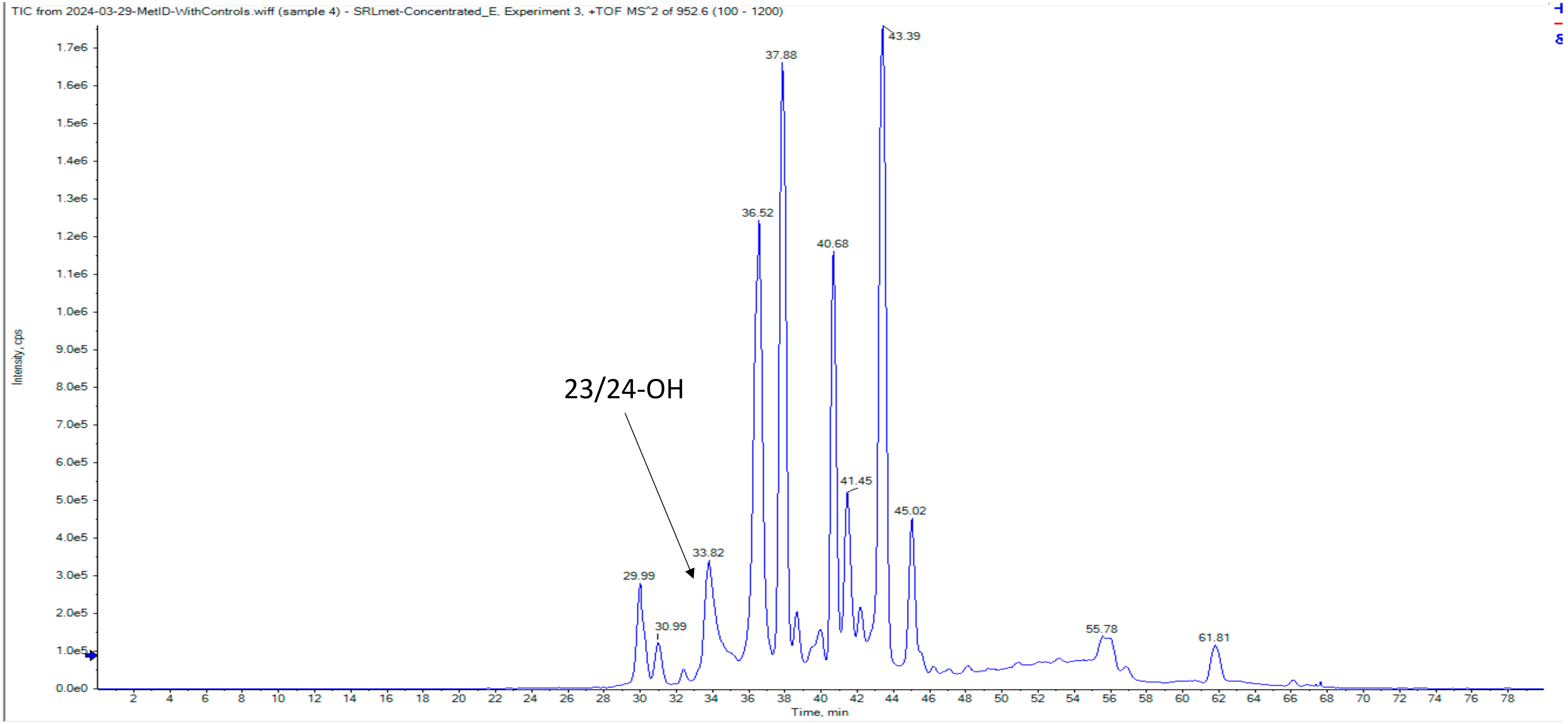

Mass Spectrum of 23- and 24-Hydroxy Sirolimus CE=65 eV, DP = 110 V

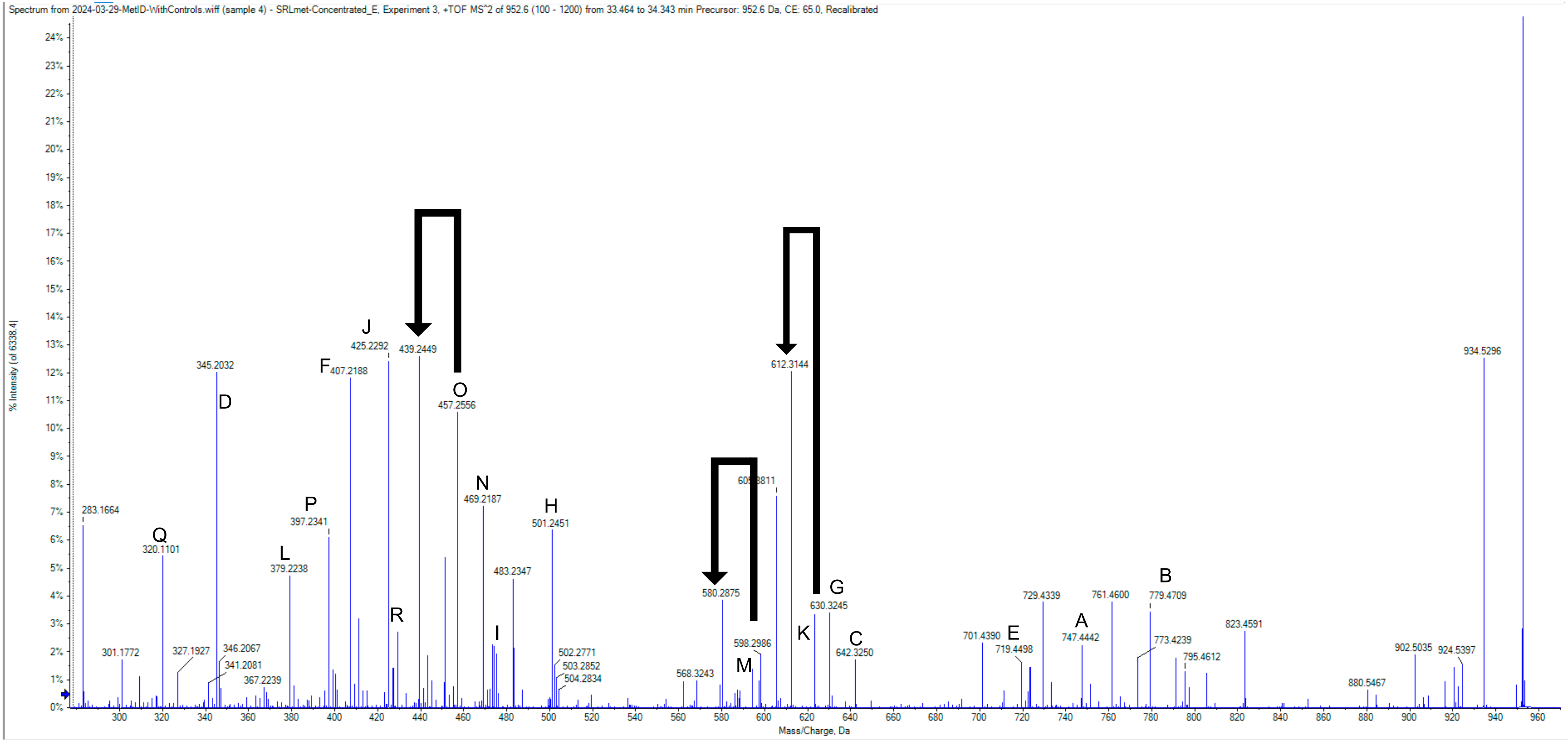

23-OH Fragmentation Pattern

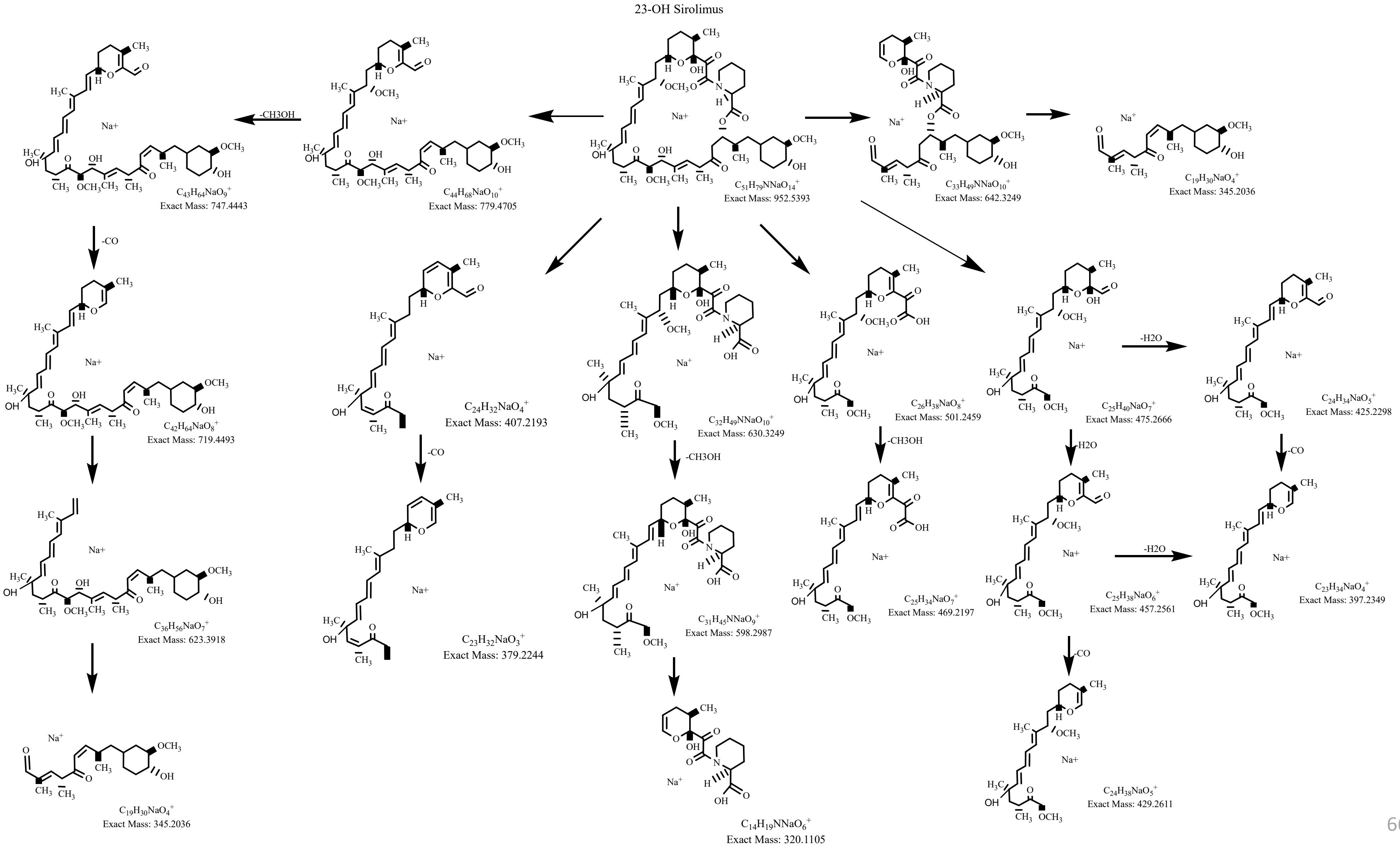

24-OH Fragmentation Pattern

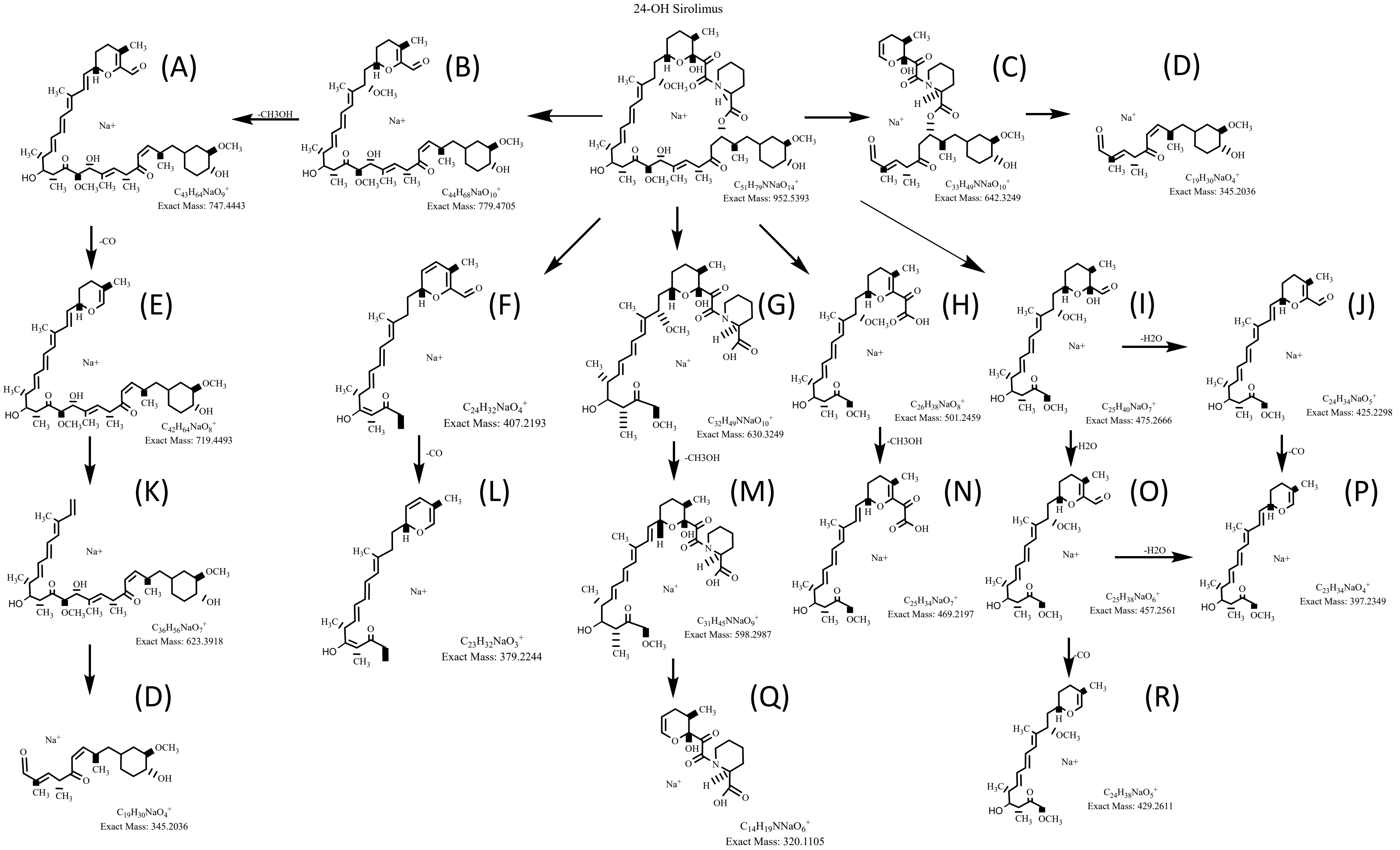

Characteristic Fragments of 24-OH Sirolimus

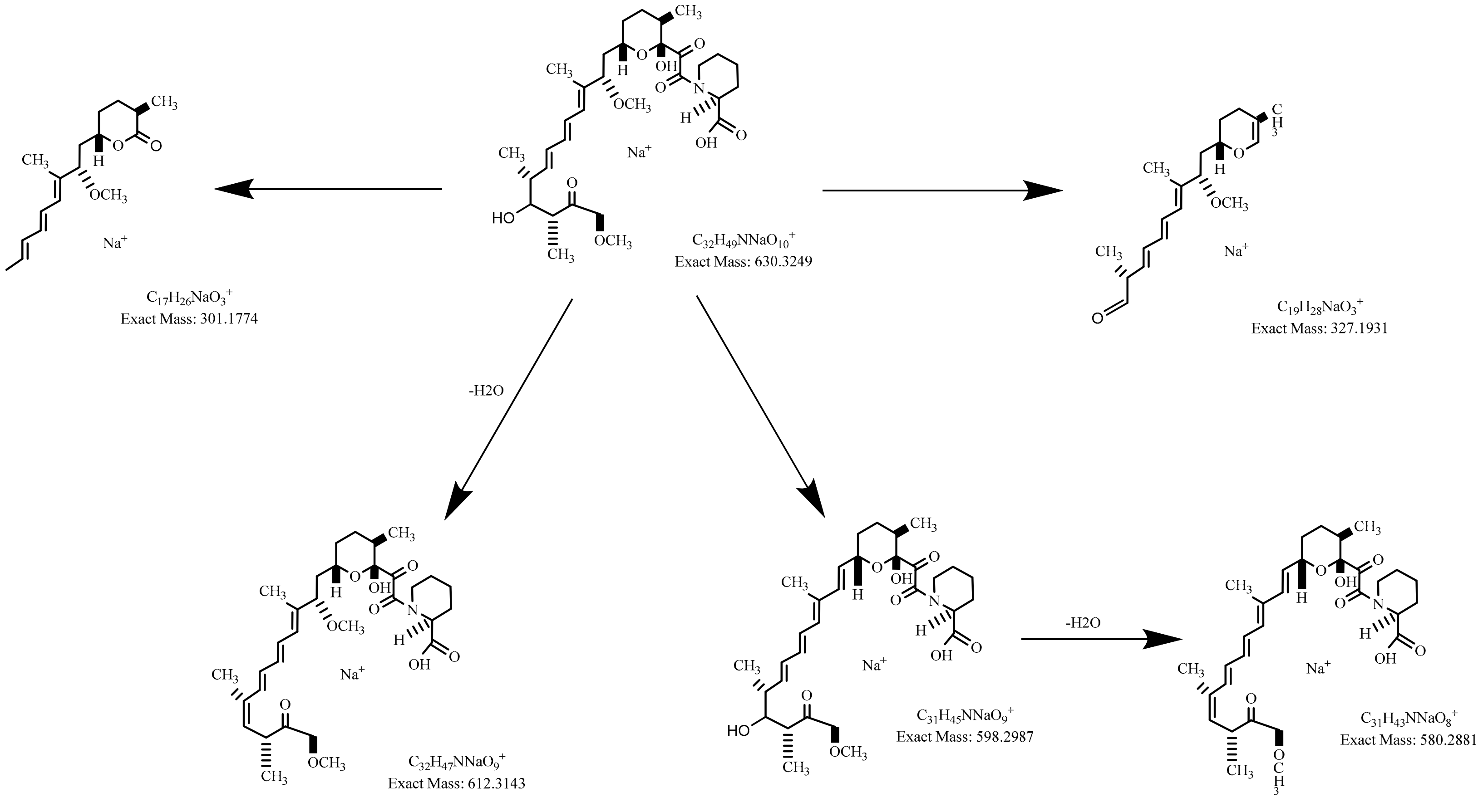

Δppm of 23/24-OH Sirolimus Fragments

	Theoretical mass	Measured mass	Δppm
**24-OHSirolimus**	952.5393	952.5393	0.0
A	747.4443	747.4442	0.1
B	779.4705	779.4709	0.6
C	642.3249	642.3250	0.2
D	345.2036	345.2032	1.2
E	719.4493	719.4498	0.6
F	407.2193	407.2188	1.2
G	630.3249	630.3245	0.6
H	501.2459	501.2451	1.6
I	475.2702	475.2689	2.7
J	425.2299	425.2292	1.5
K	623.3918	623.3916	0.4
L	379.2244	379.2238	1.6
M	598.2987	598.2986	0.1
N	469.2197	469.2187	2.1
O	457.2561	457.2556	1.0
P	397.2349	397.2341	2.1
Q	320.1105	320.1101	1.1
R	429.2611	429.2605	1.4

Characteristic Fragments

Theoretical mass	Measured mass	Δppm
612.3143	612.3144	0.2
598.2987	598.2986	0.2
580.2881	580.2875	1.0
327.1931	327.1927	1.2
301.1774	301.1772	0.7

23/24-Hydroxy Sirolimus Comments

Assinment Fragment	Sirolimus	23/ 24-OHSirolimus	H2O Loss	Comment
**24-OHSirolimus**	936.5	952.5		
A	731.4	747.4		Excludes Piperidine-OH
B	763.5	779.5		Excludes Piperidine-OH
C	642.3	642.3		Excludes 11,12,14,49, Piperidine-OH
D	345.2	345.2		Excludes 49-OH
E	703.5	719.4		Excludes Piperidine-OH
F	399.3	415.2		Excludes 49, Piperidine-OH
G	614.3	630.3	612.3	Excludes 49-OH
H	485.2	501.2		Excludes 49, piperidine-OH
I	459.3	475.3	457.3	Excludes 49, Piperidine-OH
J	409.2	425.2		Excludes 49, Piperidine-OH
K	607.4	623.4		Excludes 11,12,14, Piperidine-OH
L	397.2	413.2		Excludes 49, Piperidine-OH
M	582.3	598.3	580.0	Excludes 49-OH
N	453.2	469.2		Excludes 49, Piperidine-OH
O	441.3	457.3	439.3	Excludes 49, Piperidine-OH
P	381.2	397.2		Excludes 49, Piperidine-OH
Q	320.1	320.1		Excludes 11,12,14, Piperidine-OH
R	413.3	429.3		Excludes 49, Piperidine-OH
**Characteristic Fragments**				**612, 580, 327, 301**
**Determinant Patterns**			**All expected fragments yield additional high intensity fragments from water loss, a characteristic of 23, 24-OH due to resonance with the adjacent conjugated triene**	**Characteristic fragment for water loss (630 -18 = 612 m/z) of 23 and 24-OH**<br>**Characteristic fragment for water loss (598 -18 = 580 m/z) of 23 and 24-OH**

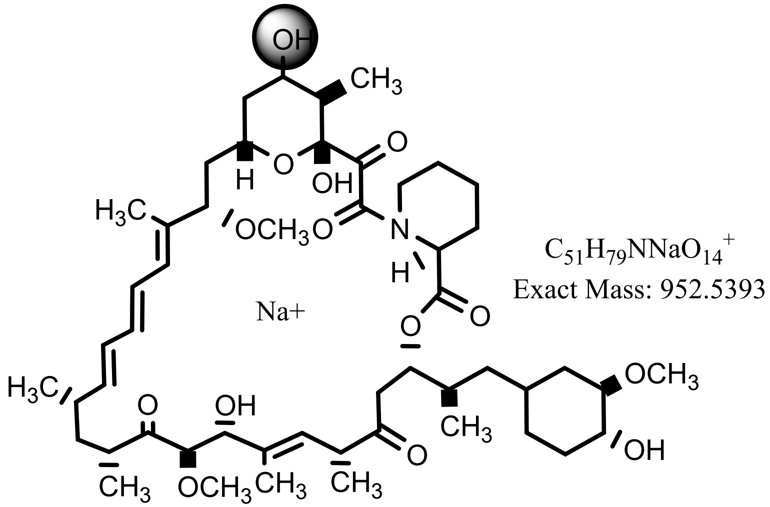

12-OH Sirolimus

12-Hydroxy Sirolimus (*m/z* = 952.5393)

Hydroxy Sirolimus Metabolites
Total Ion Chromatogram, *m/z* = 952.0

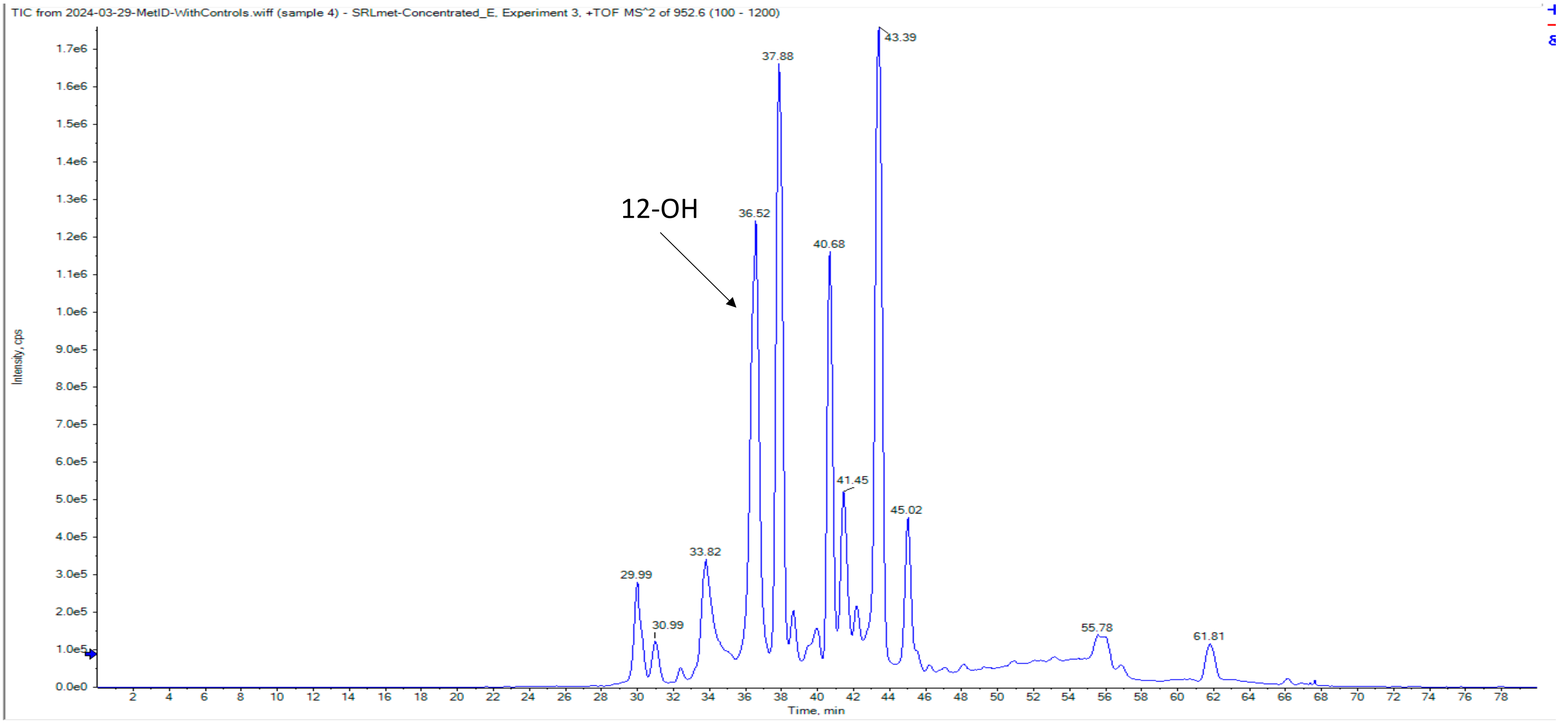

MS spectrum of 12-Hydroxy Sirolimus CE=65 eV, DP = 110 V

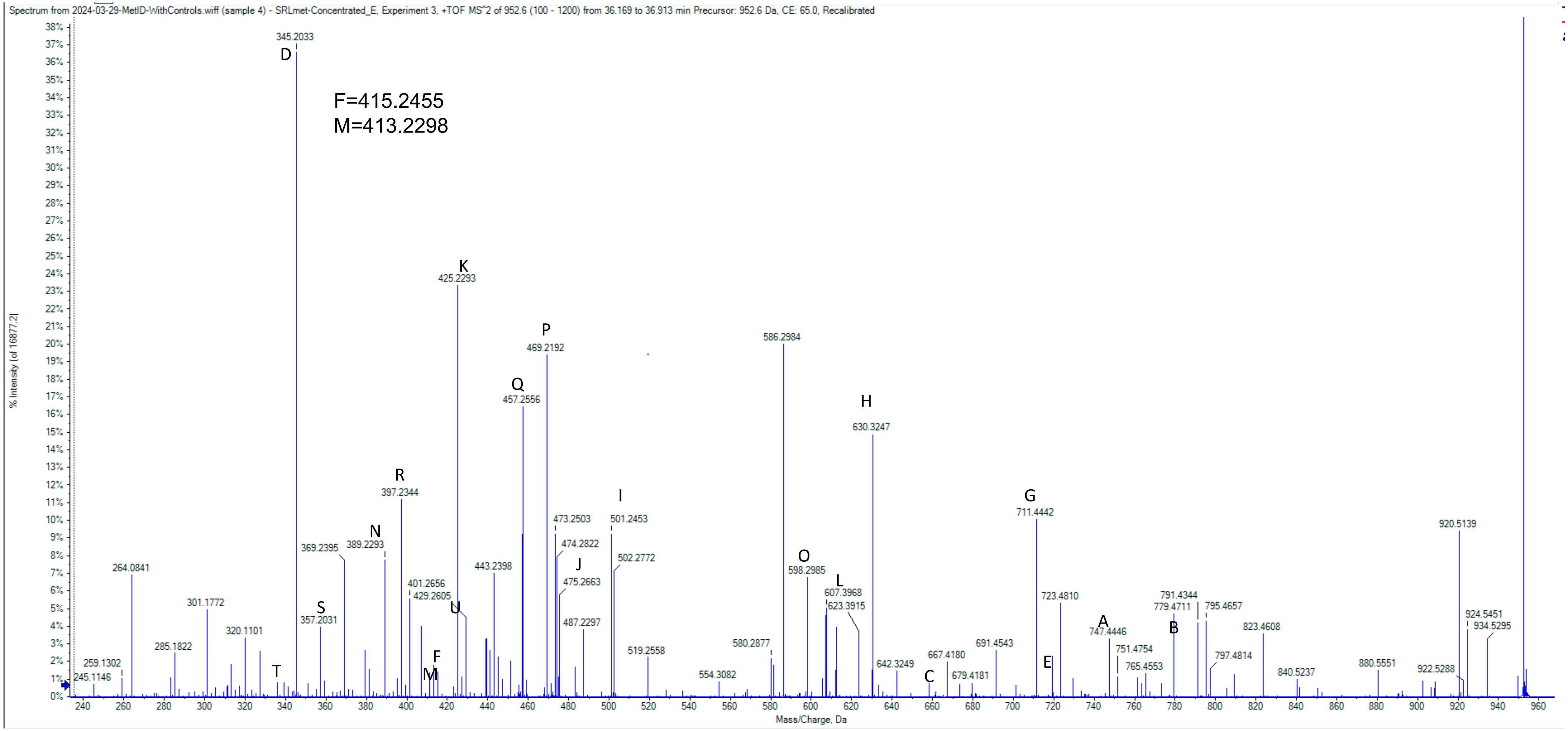

12-OH Fragmentation Pattern

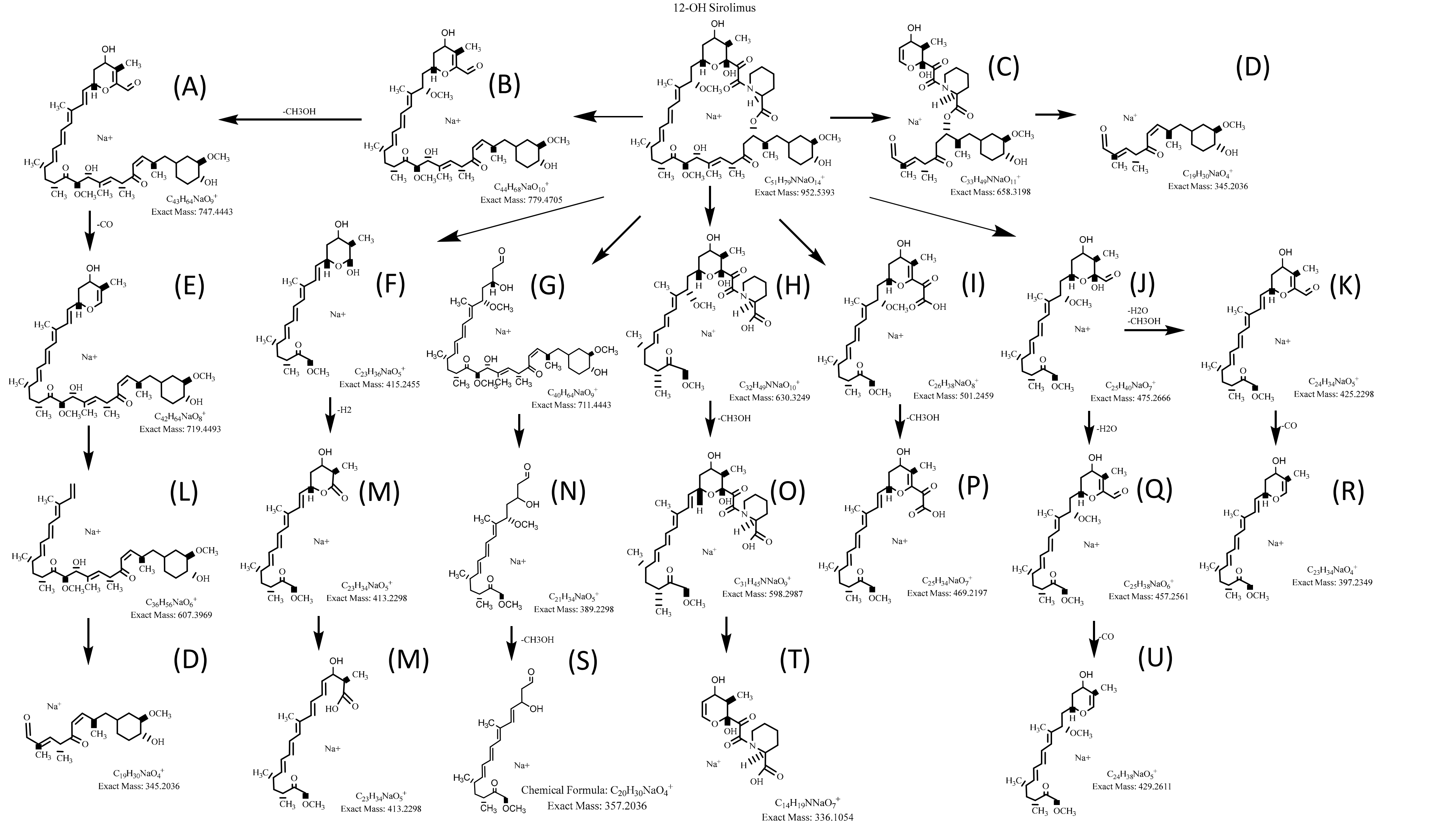

Characteristic Fragments of 12-OH Sirolimus

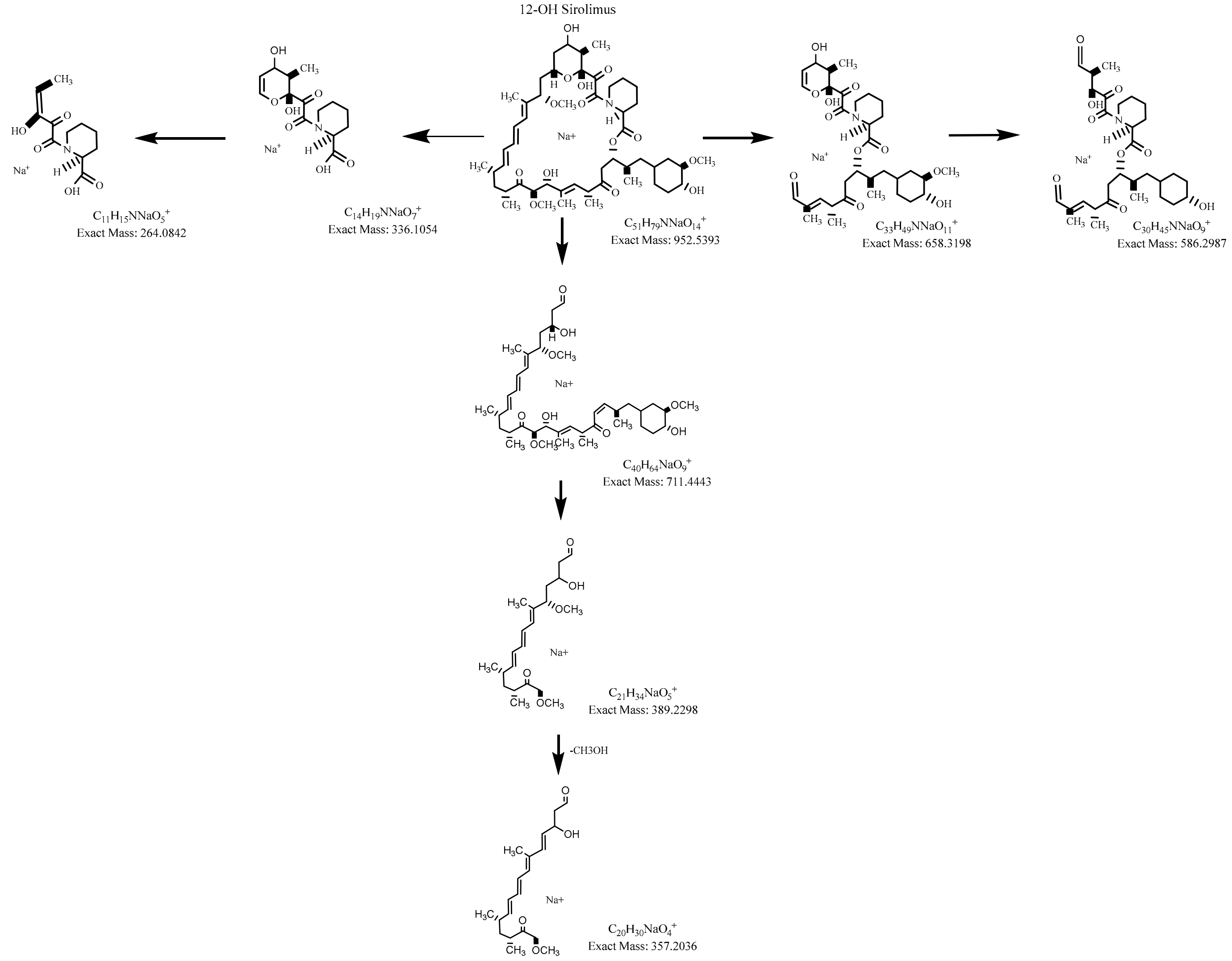

Δppm of 12-OH Sirolimus Fragments

	Theoretical mass	Measured mass	Δppm
**12-OH Sirolimus**	952.5393	952.5393	0.0
A	747.4443	747.4446	0.5
B	779.4705	779.4711	0.8
C	658.3198	658.3196	0.3
D	345.2036	345.2034	0.7
E	719.4493	719.4496	0.4
F	415.2455	415.2455	0.0
G	711.4443	711.4442	0.1
H	630.3249	630.3247	0.3
I	501.2459	501.2453	1.2
J	475.2666	475.2663	0.7
K	425.2299	425.2293	1.3
L	607.3969	607.3968	0.2
M	413.2299	413.2298	0.1
N	389.2298	389.2293	1.3
O	598.2987	598.2985	0.3
P	469.2197	469.2192	1.0
Q	457.2561	457.2556	1.0
R	397.2349	397.2344	1.3
S	357.2036	357.2031	1.4
T	336.1054	336.1048	1.7
U	429.2611	429.2605	1.4

12-Hydroxy Sirolimus Comments

Fragment assignment 12-OH Sirolimus	Sirolimus	12-OH Sirolimus	Comment
A	731.4	747.4	Possible 11,12,14,24,25,46, 49-OH
B	763.5	779.5	Possible 11,12,14,24,25,46, 49-OH
C	642.3	658.3	Excludes23,24,25,49-OH
D	345.2	345.2	Excludes 49-OH
E	703.5	719.4	Excludes Piperidine-OH
F	399.3	415.2	Excludes 49, Piperidine-OH
G	ND	711.4	Confirms 12-OH
H	614.3 (G)	630.3	Excludes 49-OH
I	485.2 (H)	501.2	Excludes 49, Piperidine-OH
J	459.3 (I)	475.3	Excludes 49, Piperidine-OH
K	409.2 (J)	425.2	Excludes 49, Piperidine-OH
L	607.4 (K)	607.4	Excludes23,24,25,46, 49-OH
M	397.2 (L)	413.2	Excludes 49, Piperidine-OH
N	ND	389.2	Confirms 12-OH
O	582.3 (M)	598.3	Excludes 49-OH
P	453.2 (N)	469.2	Excludes 49, Piperidine-OH
Q	441.3 (O)	457.3	Excludes 49, Piperidine-OH
R	381.2 (P)	397.2	Excludes 49, Piperidine-OH
S	ND	357.2	MeOH Loss from 389.2
T	320.1 (Q)	336.1	Excludes23,24,25,46, 49-OH
U	413.3 (R)	429.3	Excludes 49, Piperidine-OH
**G**		**711.4**	**Characteristic Fragments, Unique to 12-OH**
**N**		**389.2**	**Characteristic Fragments, Unique to 12-OH**
**S**		**357.2(MeOH loss from 389.2)**	**Characteristic Fragments, Unique to 12-OH**

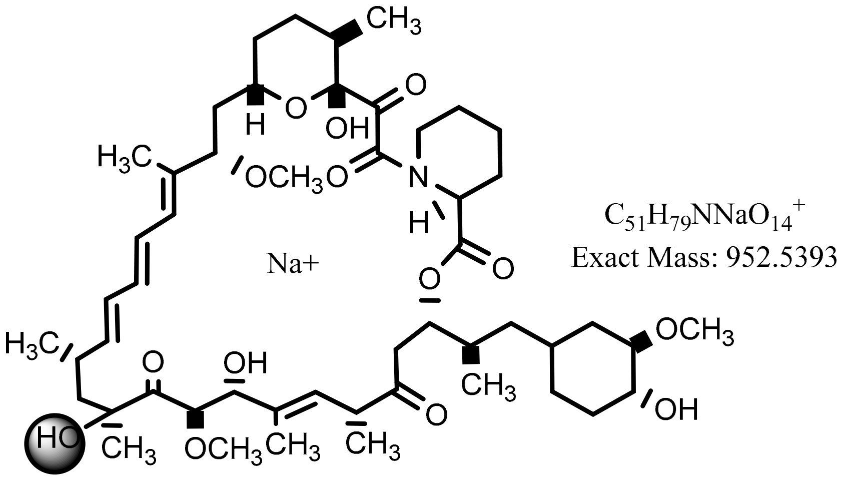

25-OH Sirolimus

25-Hydroxy Sirolimus (m/z = 952.5393)

Hydroxy Sirolimus Metabolites
Total Ion Chromatogram, *m/z* = 952.0

This metabolite co-eluted with 12-OH SRL and thus required a more isocratic approach to achieve better separation. Consequently, the separation method was adjusted by slightly altering the gradient, which yielded a distinct peak for 25-OH SRL and enabled its structural identification.

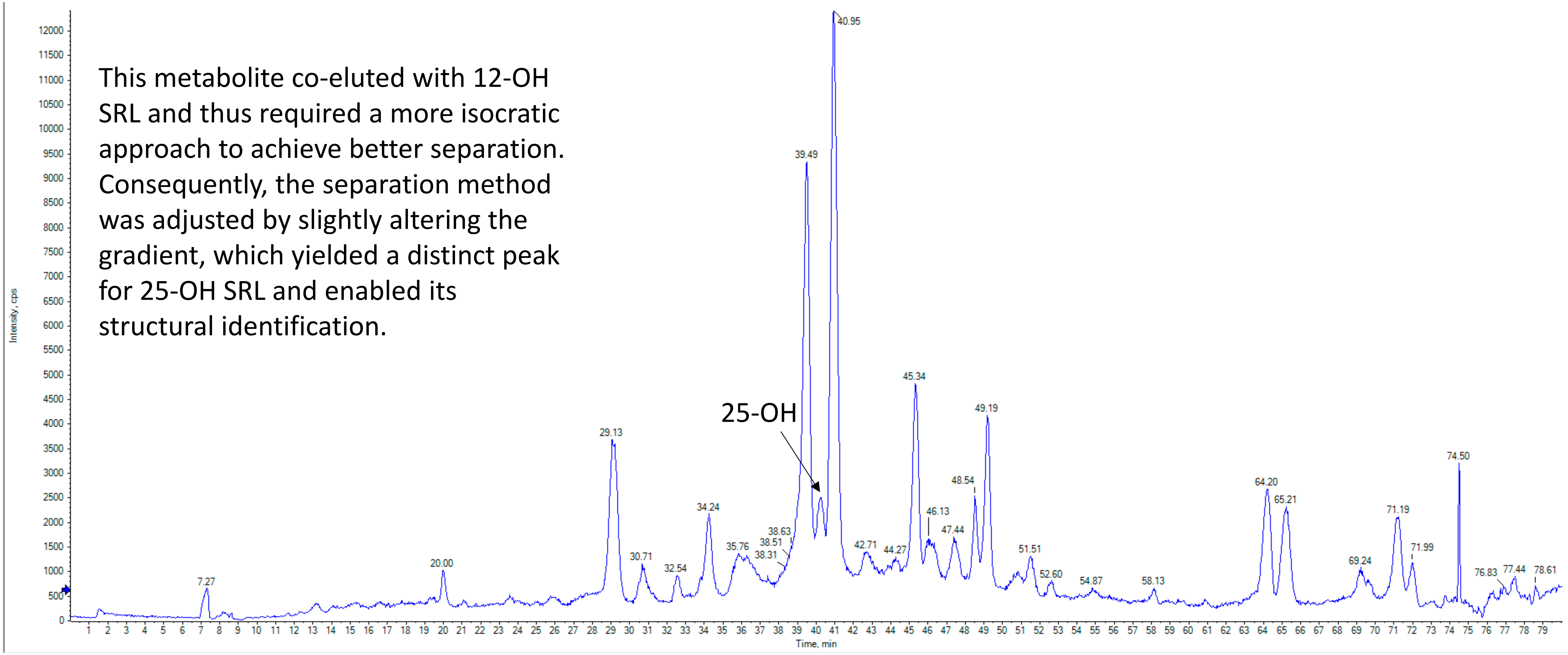

25-Hydroxy Sirolimus Chromatogram (Top)
Mass Spectrum, QTOF Fragmentation, (952.5393 Extracted) (Bottom)

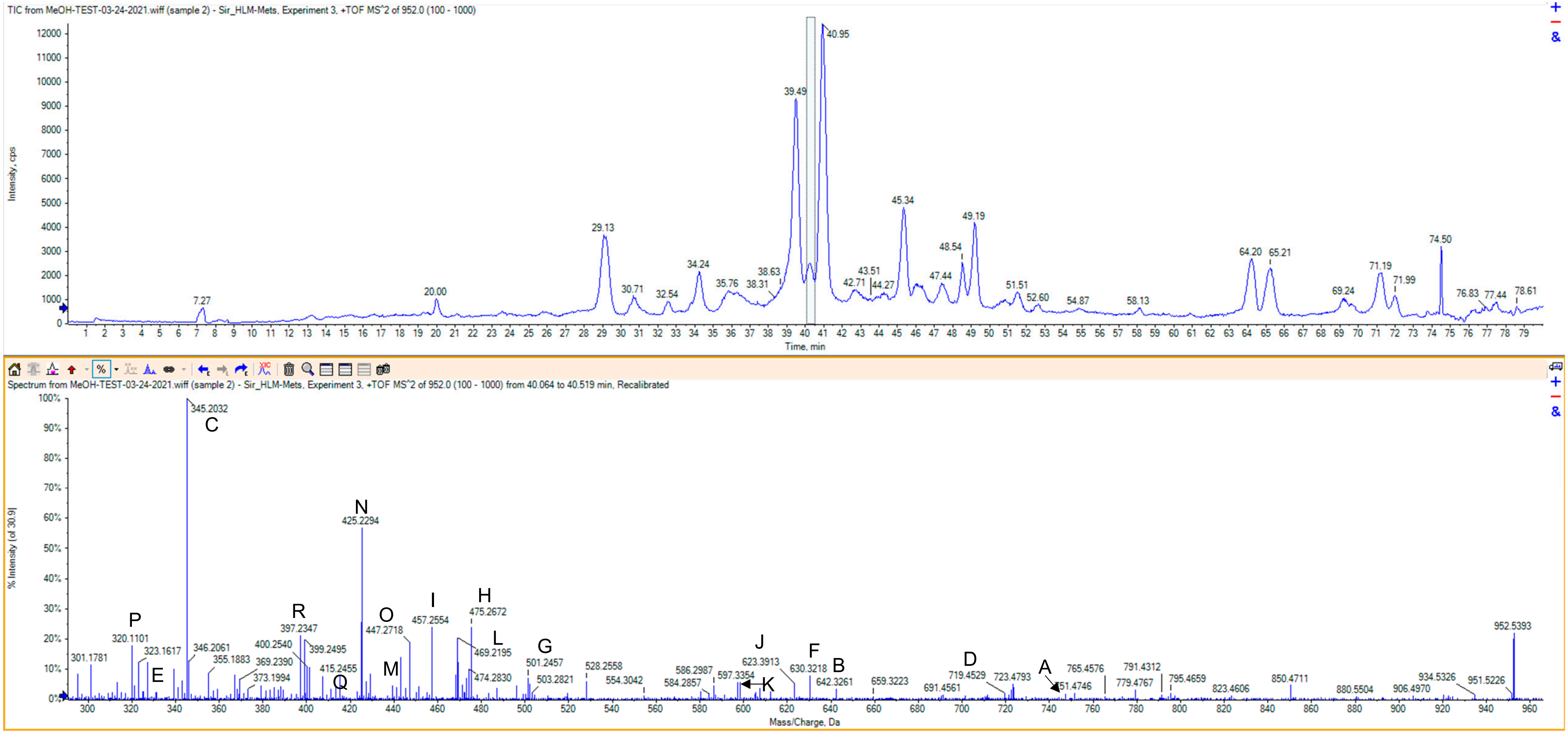

25-OH Fragmentation Pattern

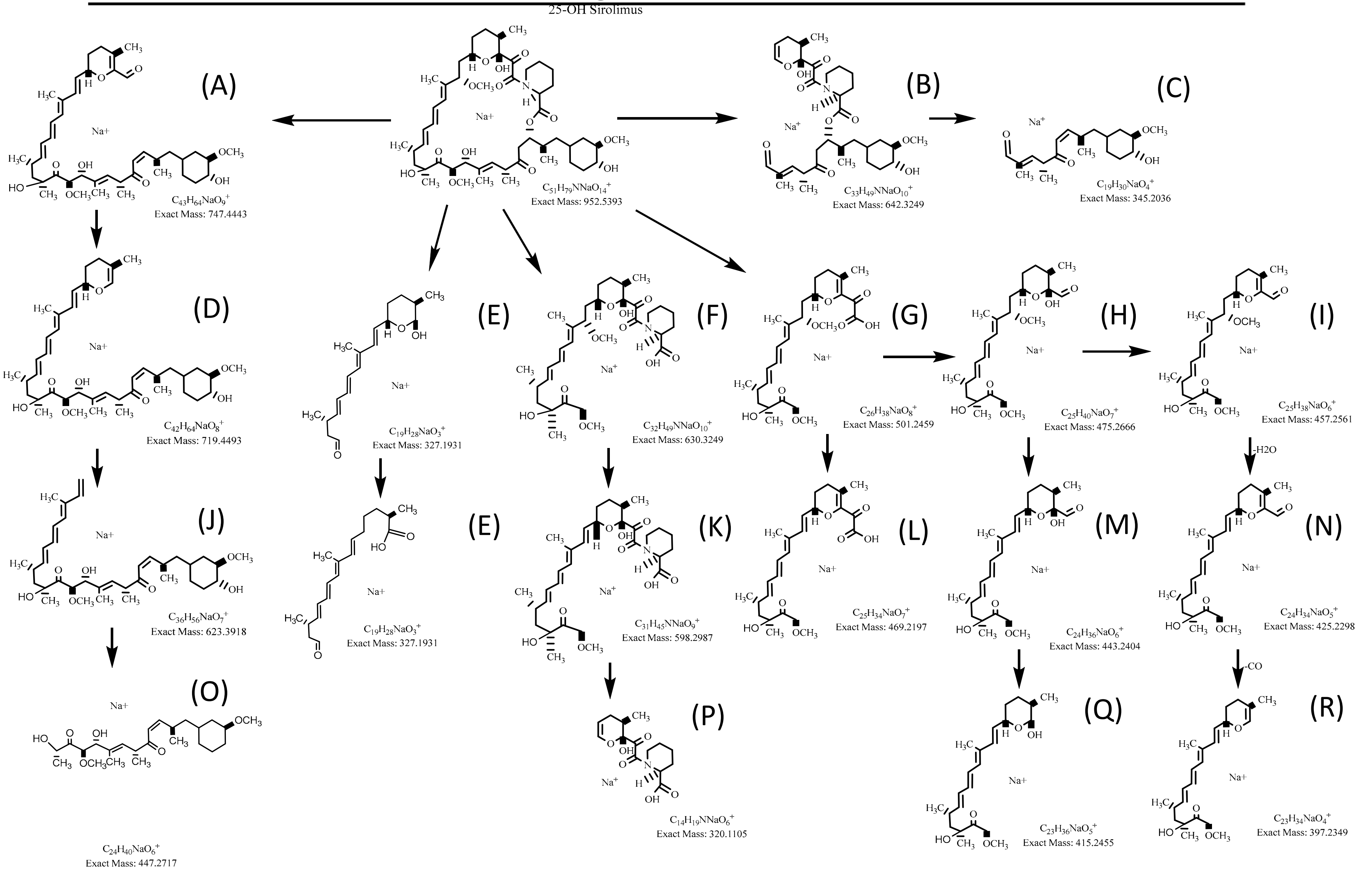

Characteristic Fragments of 25-OH Sirolimus

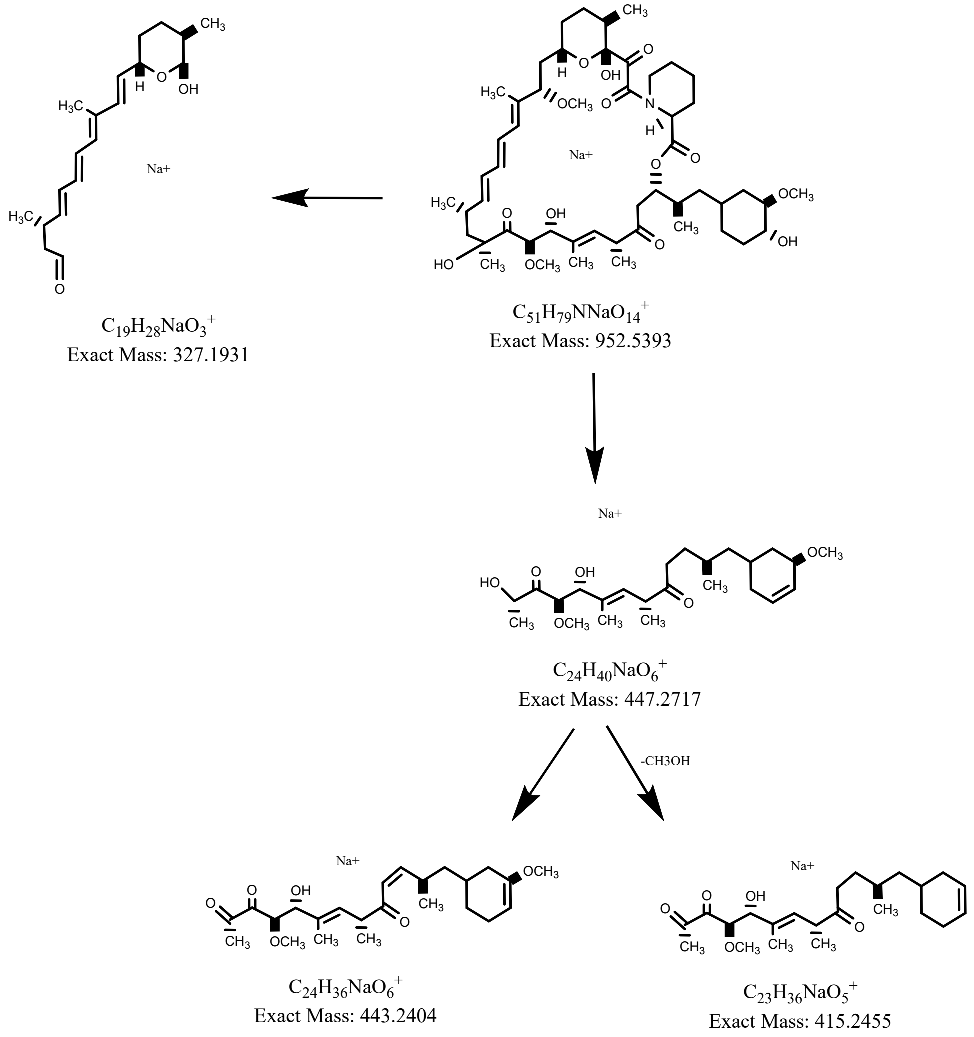

Δppm of 25-Hydroxy Sirolimus Fragments

	Theoretical mass	Measured mass	Δppm
**25-OH Sirolimus**	952.5393	952.5393	0.0
A	747.4443	747.4426	2.3
B	642.3249	642.3261	1.9
C	345.2036	345.2032	1.2
D	719.4493	719.4529	5.0
E	327.1931	327.1922	2.8
F	630.3249	630.3218	4.9
G	501.2459	501.2457	0.4
H	475.2666	475.2672	1.3
I	457.2561	457.2554	1.5
J	623.3918	623.3913	0.8
K	598.2987	598.2976	1.8
L	469.2197	469.2195	0.4
M	443.2404	443.2392	2.7
N	425.2298	425.2294	0.9
O	447.2717	447.2718	0.2
P	320.1105	320.1101	1.2
Q	415.2455	415.2455	0.0
R	397.2349	397.2347	0.5

25-Hydroxy Sirolimus Comments

Fragment assignment 25-OHSirolimus	Sirolimus	25-OHSirolimus	Comment
A	731.4	747.4	Possible 11,12,14,24,25,46, 49-OH
B	642.3 (C)	642.3	excludes piperidine, 11,12,14, 49-OH
C	345.2 (D)	345.2	Excludes 49-OH
D	703.5 (E)	719.4	Possible 11,12,14,24,25,46, 49-OH
E	ND	327.2	Could be Characteristic Fragments
F	614.3(G)	630.3	Excludes 49-OH
G	485.2 (H)	501.2	Possible 11,12,14,24,25,46-OH
H	459.3 (I)	475.3	Possible 11,12,14,24,25,46-OH
I	441.3 (O)	457.3	Possible 11,12,14,24,25,46-OH
J	607.4(K)	623.4	Possible 24,25,46, 49-OH
K	582.3 (M)	598.3	Excludes 49-OH
L	453.2 (N)	469.2	Possible 11,12,14,24,25,46-OH
M	ND	443.2	Possible 11,12,14,24,25,46-OH
N	409.2(J)	425.2	Possible 11,12,14,24,25,46-OH
O	ND	447.3	Characteristic Fragments
P	320.1(Q)	320.1	excludes piperidine, 11,12,14-OH
Q	ND	415.2	Possible 11,12,14,24,25,46-OH
R	381.2 (P)	397.2	Possible 11,12,14,24,25,46-OH
**O**		**447.3**	**Characteristic Fragments, Unique to 25-OH**
**E**		**327.2**	**Characteristic Fragments, Unique to 25-OH**

25-Hydroxy Sirolimus Characteristic Fragments

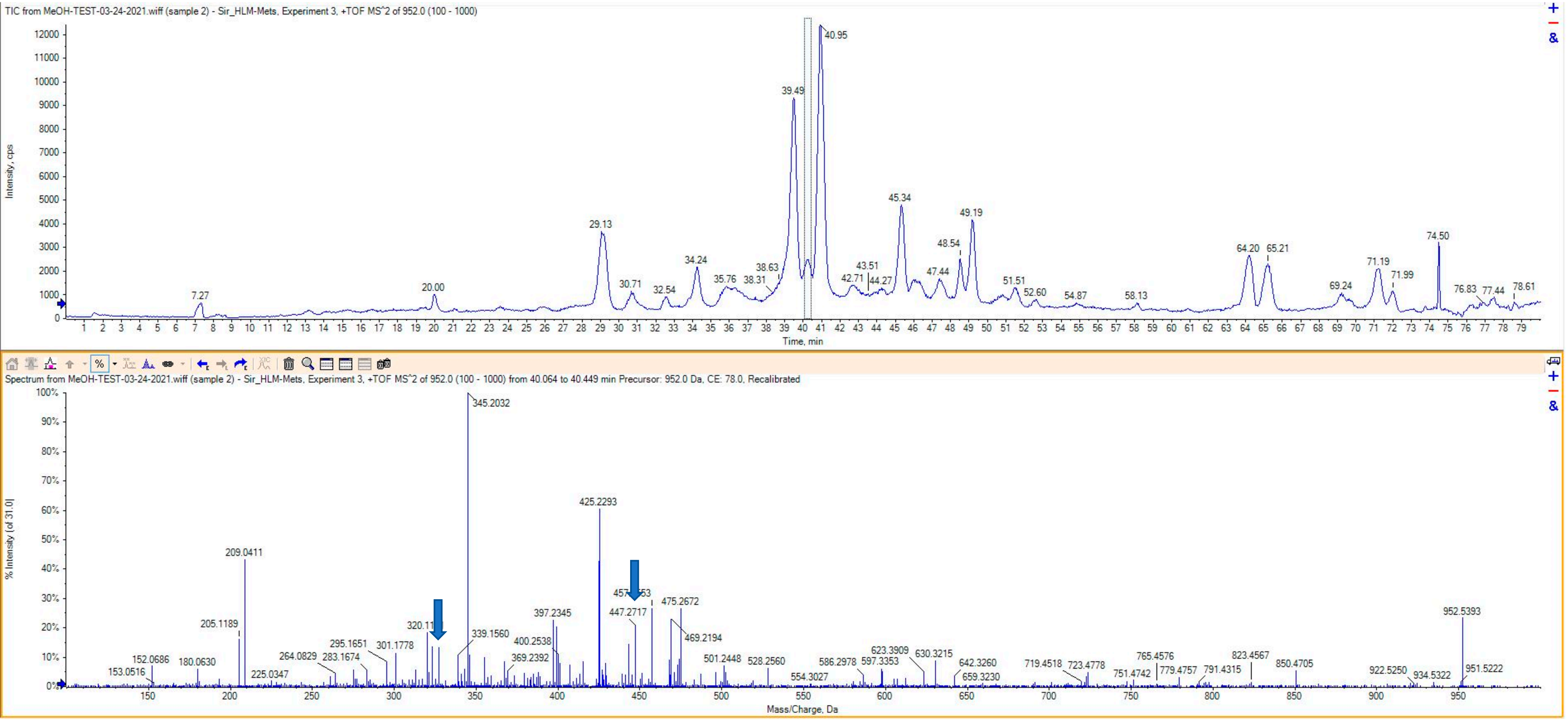

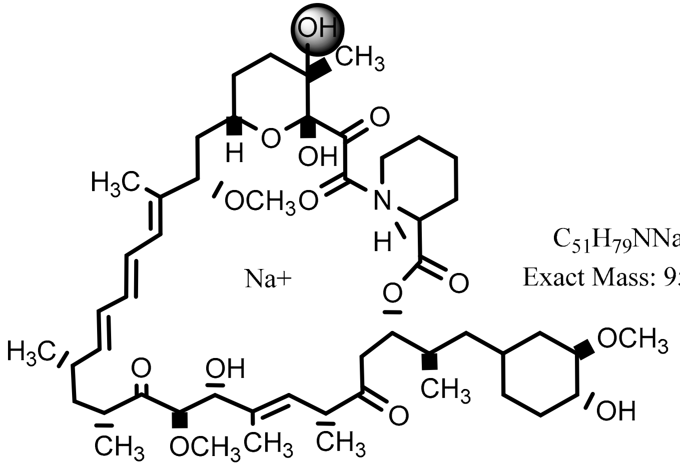

$C_{51}H_{79}NNaO_{14}^{+}$
Exact Mass: 952.5393

11-OH Sirolimus

11-Hydroxy Sirolimus (*m/z* = 952.5393)

Hydroxy Sirolimus Metabolites
Total Ion Chromatogram, *m/z* = 952.0

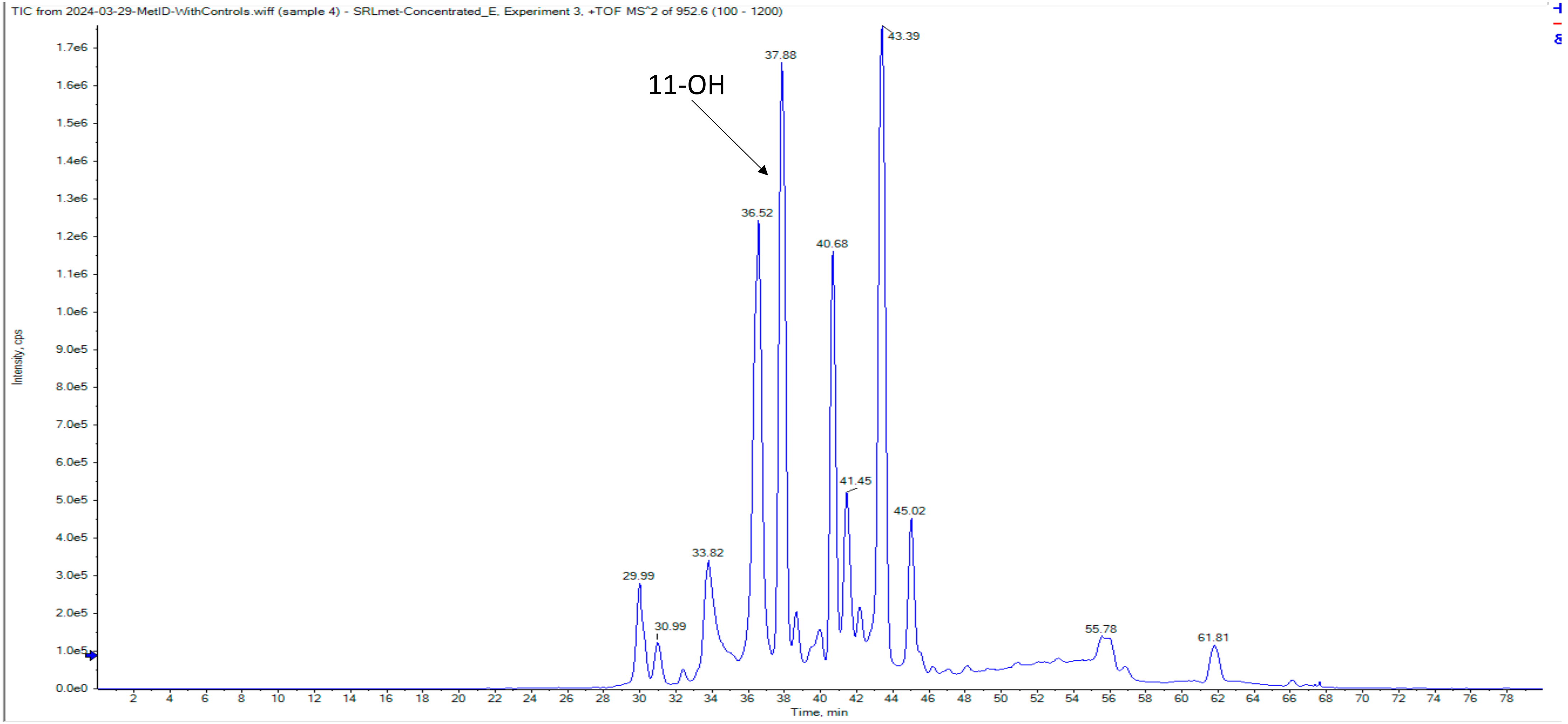

11-Hydroxy Sirolimus Chromatogram (Top)
Mass Spectrum, QTOF Fragmentation, (952.5393 Extracted) (Bottom)

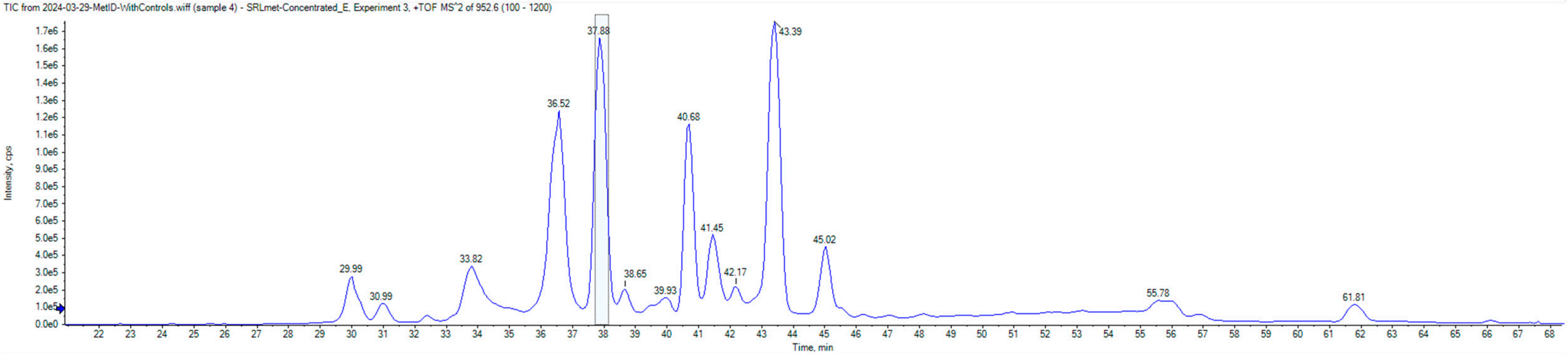

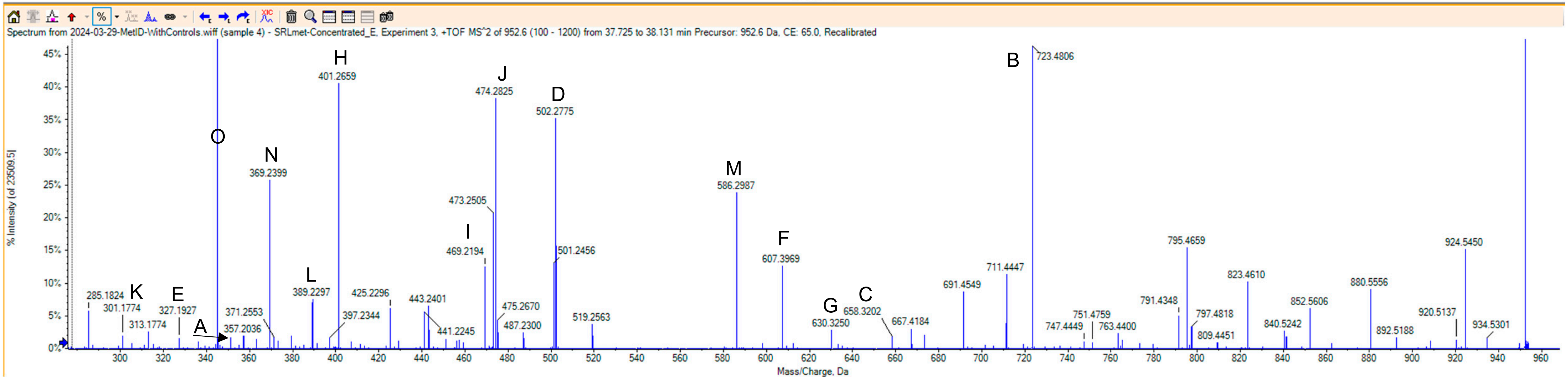

11-OH Fragmentation Pattern

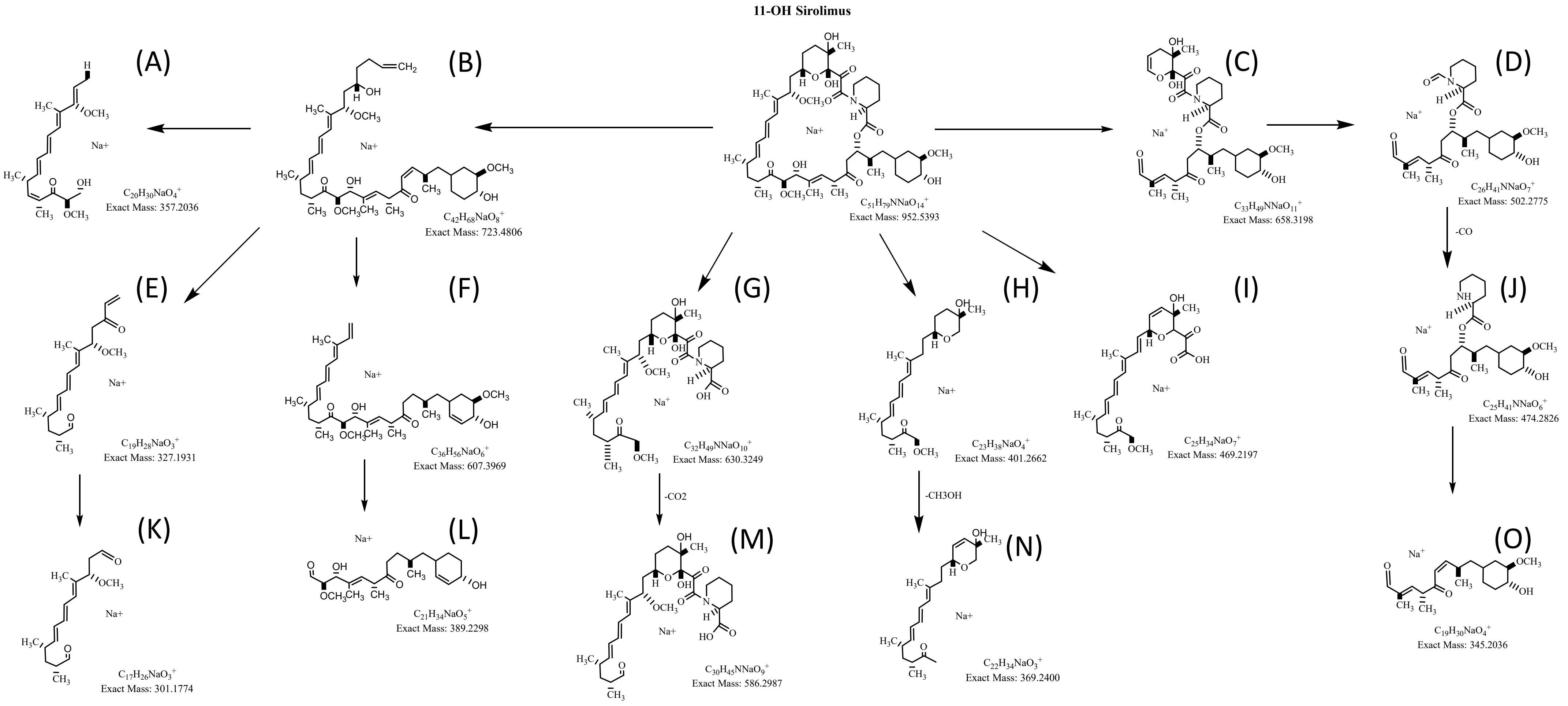

Characteristic Fragments of 11-OH Sirolimus

11-OH Sirolimus

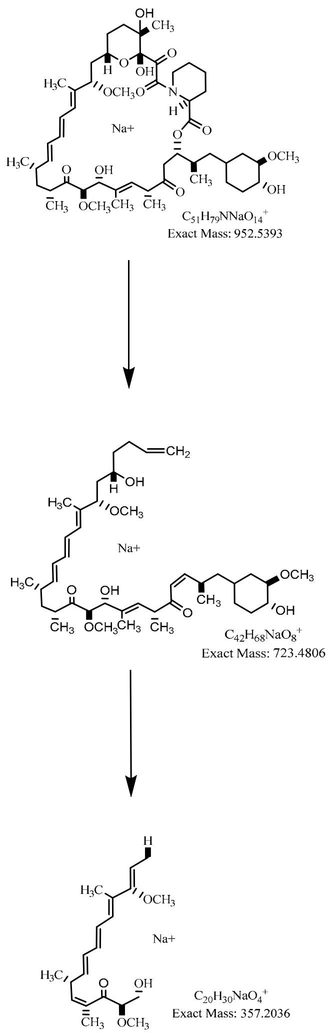

Δppm of 11-OH Sirolimus Fragments

	Theoretical mass	Measured mass	Δppm
**11-OH Sirolimus**	952.5393	952.5393	0.0
A	357.2036	357.2036	0.0
B	723.4806	723.4806	0.1
C	658.3198	658.3202	0.6
D	502.2775	502.2775	0.0
E	327.1931	327.1927	1.2
F	607.3969	607.3969	0.0
G	630.3249	630.3250	0.2
H	401.2662	401.2659	0.8
I	469.2197	469.2194	0.6
J	474.2826	474.2825	0.2
K	301.1774	301.1774	0.0
L	389.2299	389.2297	0.4
M	586.2986	586.2987	0.2
N	369.2400	369.2399	0.3
O	345.2036	345.2035	0.4

11-Hydroxy Sirolimus Comments

Assignment fragment 11-OH Sirolimus	Sirolimus	11-OHSirolimus	Comment
A	ND	357.2	Characteristic Fragments, Unique to 11-OH
B	ND	723.5	Characteristic Fragments, Unique to 11-OH
C	642.3	658.3	Possible piperidine, 11,12,14, ,49-OH
D	ND	502.3	Exclude piperidine, 49-OH
E	ND	327.2	Exclude piperidine, 49-OH
F	607.4 (K)	607.4	Excludes 24,25,46,49-OH
G	614.3	630.3	exclude 49-OH
H	ND	401.2	Possible 11,12,14,24,25,46-OH
I	453.2 (N)	469.2	Possible 11,12,14,24,25,46-OH
J	ND	474.3	Exclude piperidine, 49-OH
K	ND	301.1	Characteristic Fragments, Unique to 11-OH
L	ND	389.2	Exclude 49-OH
M	ND	586.2	Possible piperidine, 11,12,14,24,25-OH
N	ND	369.2	Possible 11,12,14,24,25, 46-OH
O	345.2 (D)	345.2	Excludes 49-OH
**A**		**357.2**	**Characteristic Fragments, Unique to 11-OH**
**B**		**723.5**	**Characteristic Fragments, Unique to 11-OH**

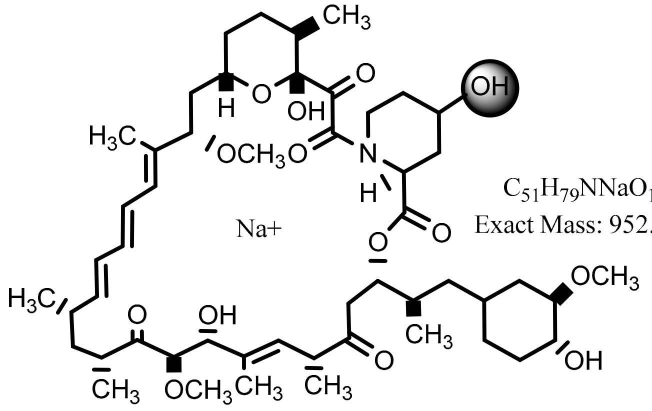

OH-piperidine Sirolimus

$C_{51}H_{79}NNaO_{14}^{+}$
Exact Mass: 952.5393

Piperidine Hydroxy Sirolimus (*m/z* = 952.5393)

Hydroxy Sirolimus Metabolites
Total Ion Chromatogram, *m/z* = 952.0

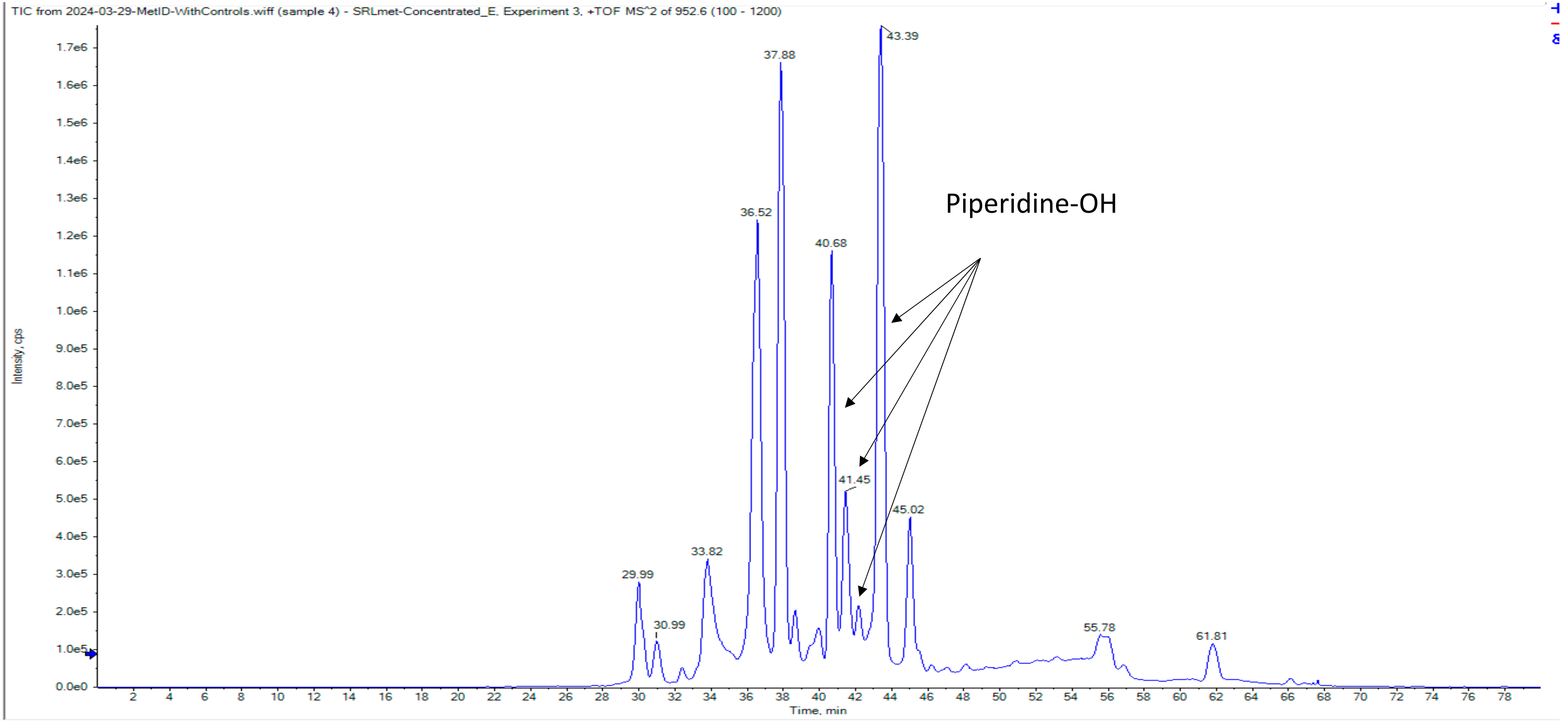

Piperidine-Hydroxy Sirolimus Chromatogram (Top) Mass Spectrum, QTOF Fragmentation, (952.5393 Extracted) (Bottom)

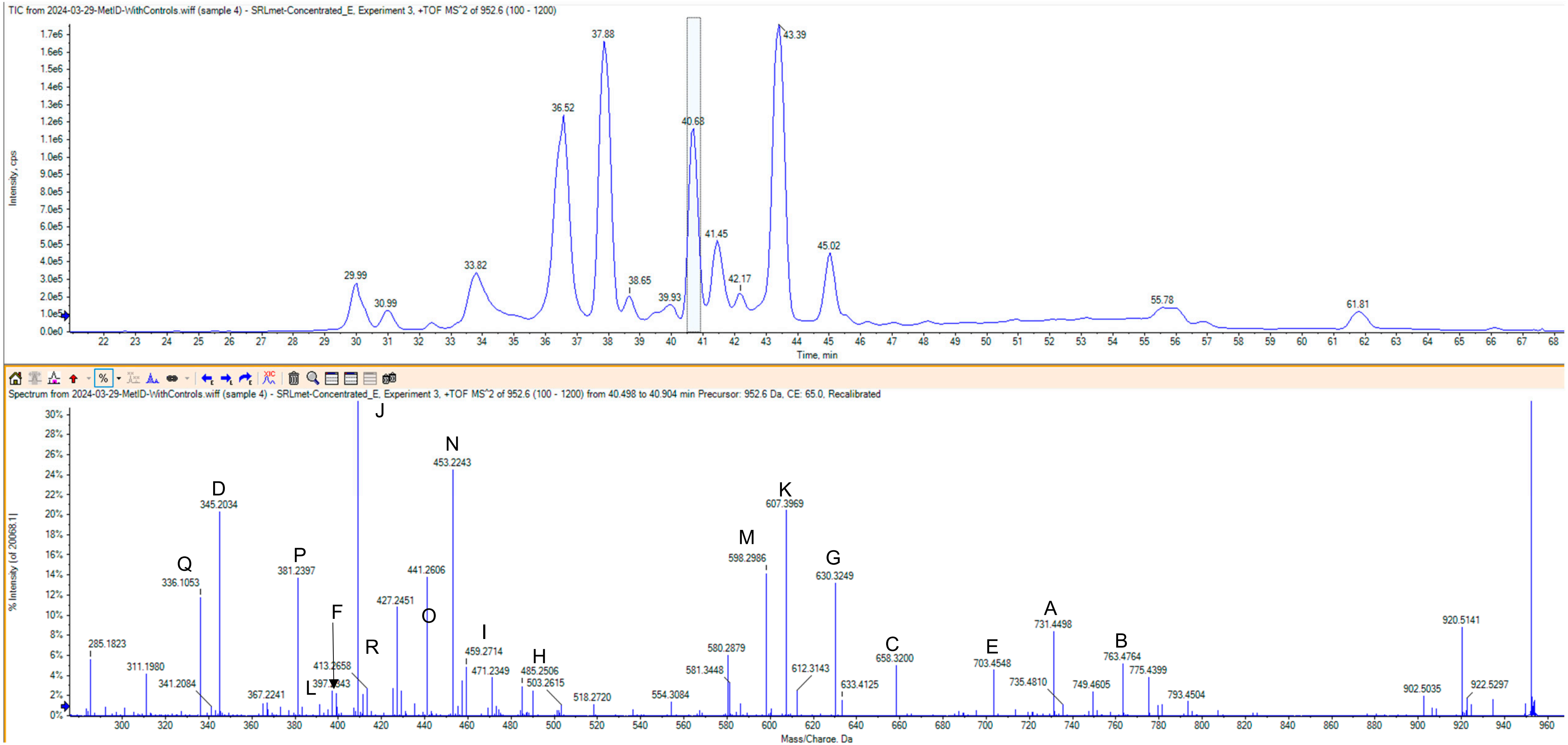

Piperidine-Hydroxy Fragmentation Pattern

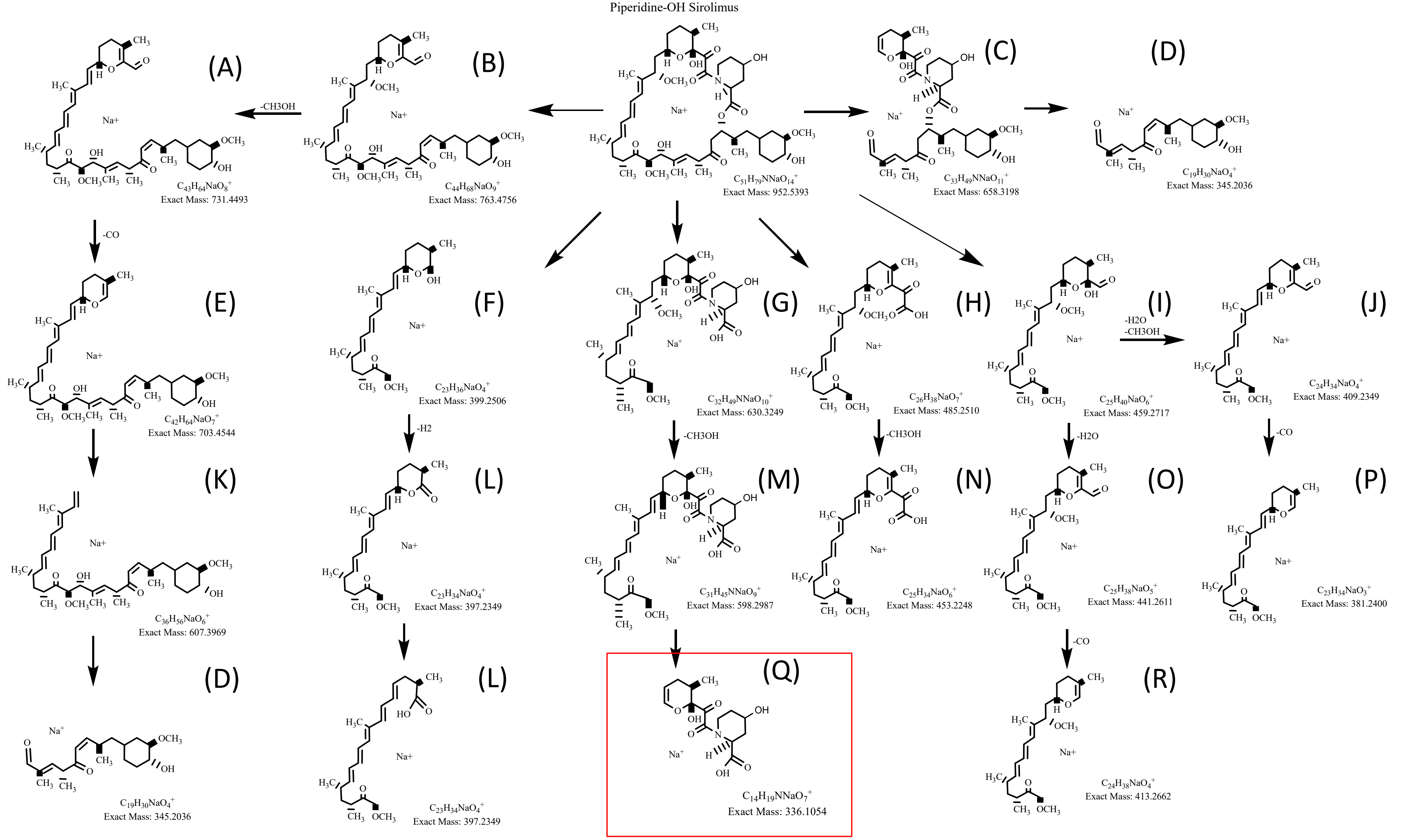

Δppm of Piperidine-Hydroxy Sirolimus Fragments

	Theoretical mass	Measured mass	Δppm
**Piperidine -OH Sirolimus**	952.5393	952.5393	0.0
A	731.4493	731.4498	0.6
B	763.4756	763.4764	1.1
C	658.3198	658.3200	0.3
D	345.2036	345.2034	0.7
E	703.4544	703.4548	0.5
F	399.2506	399.2496	2.5
G	630.3249	630.3249	0.0
H	485.2510	485.2506	0.8
I	459.2717	459.2714	0.7
J	409.2349	409.2341	2.0
K	607.3969	607.3969	0.0
L	397.2349	397.2343	1.6
M	598.2987	598.2986	0.1
N	453.2248	453.2243	1.0
O	441.2612	441.2606	1.2
P	381.2400	381.2397	0.8
Q	336.1054	336.1053	0.2
R	413.2662	413.2658	1.0

Piperidine-Hydroxy Sirolimus Comments

Assignment fragment Piperidine -OH Sirolimus	Sirolimus	Piperidine-OH	Comment
A	731.4	731.4	Excludes all, except Piperidine-OH
B	763.5	763.5	Excludes all, except Piperidine-OH
C	642.3	658.3	Possible 11,12,14,49, Piperidine-OH
D	345.2	345.2	Excludes 49-OH
E	703.5	703.5	Excludes all, except Piperidine-OH
F	399.3	399.2	Excludes 11,12,14,23, 24,25,46-OH
G	614.3	630.3	Excludes 49-OH
H	485.2	485.2	Excludes 11,12,14,23, 24,25,46-OH
I	459.3	459.3	Excludes 11,12,14,23, 24,25,46-OH
J	409.2	409.2	Excludes 11,12,14,23, 24,25,46-OH
K	607.4	607.4	Excludes 23, 24,25,46, 49-OH
L	397.2	397.2	Excludes 11,12,14,23,24,25,46-OH
M	582.3	598.3	Excludes 49-OH
N	453.2	453.2	Excludes 11,12,14,23,24,25,46-OH
O	441.3	441.3	Excludes 11,12,14,23,24,25,46-OH
P	381.2	381.2	Excludes 11,12,14,23,24,25,46-OH
Q	320.1	336.1	Possible 11,12,14,piperidine-OH
R	413.3	413.3	Excludes 11,12,14,23,24,25,46-OH
**Q**	320.1	336.1	**Wasn't present in 11,12,14-OH, possible OH-Piperidine**
**Presence of all these fragments exclude all others except the Piperidine-OH**			

Piperidine-Hydroxy Sirolimus Chromatogram (Top)
Mass Spectrum, QTOF Fragmentation, (952.5393 Extracted) (Bottom)

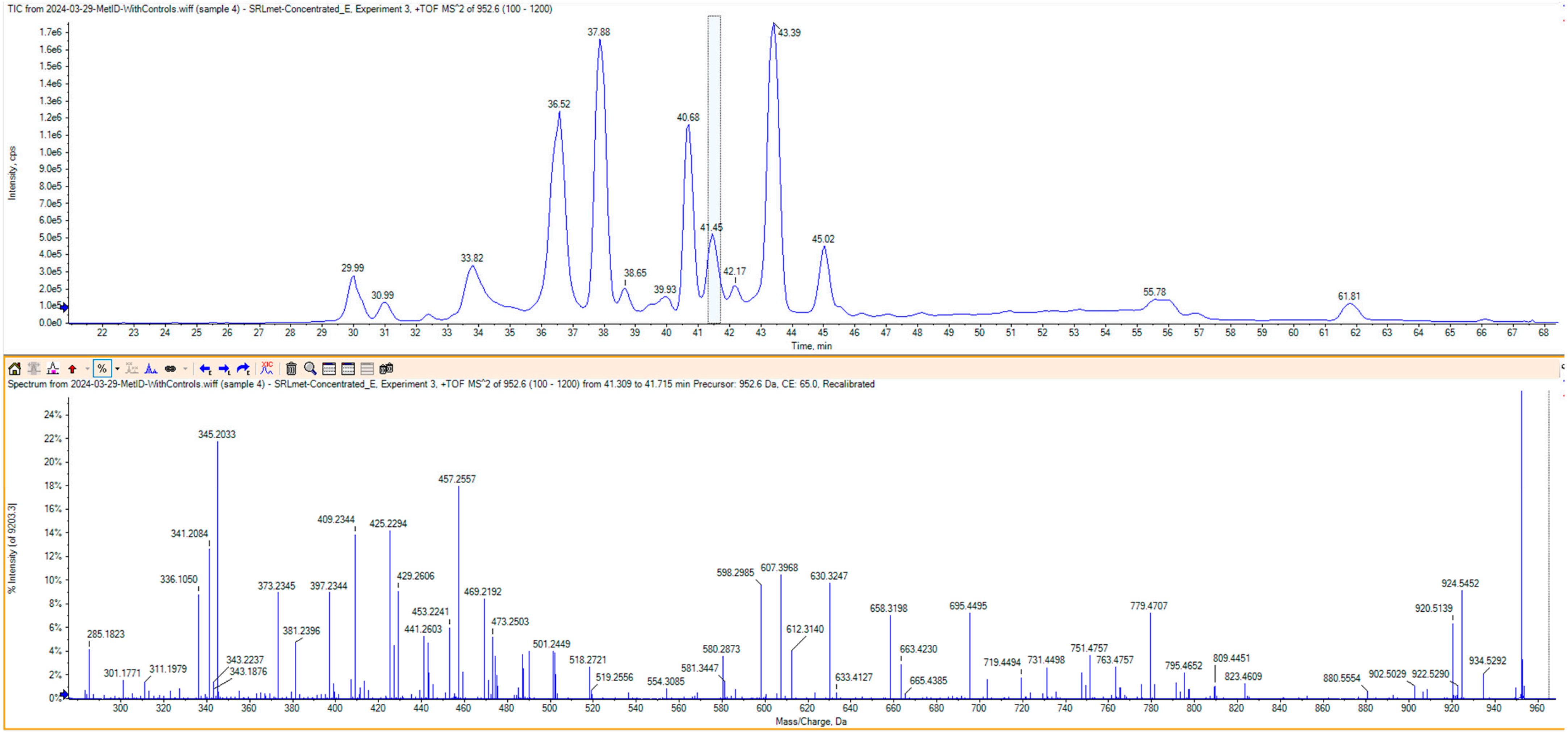

Piperidine-Hydroxy Sirolimus Chromatogram (Top)
Mass Spectrum, QTOF Fragmentation, (952.5393 Extracted) (Bottom)

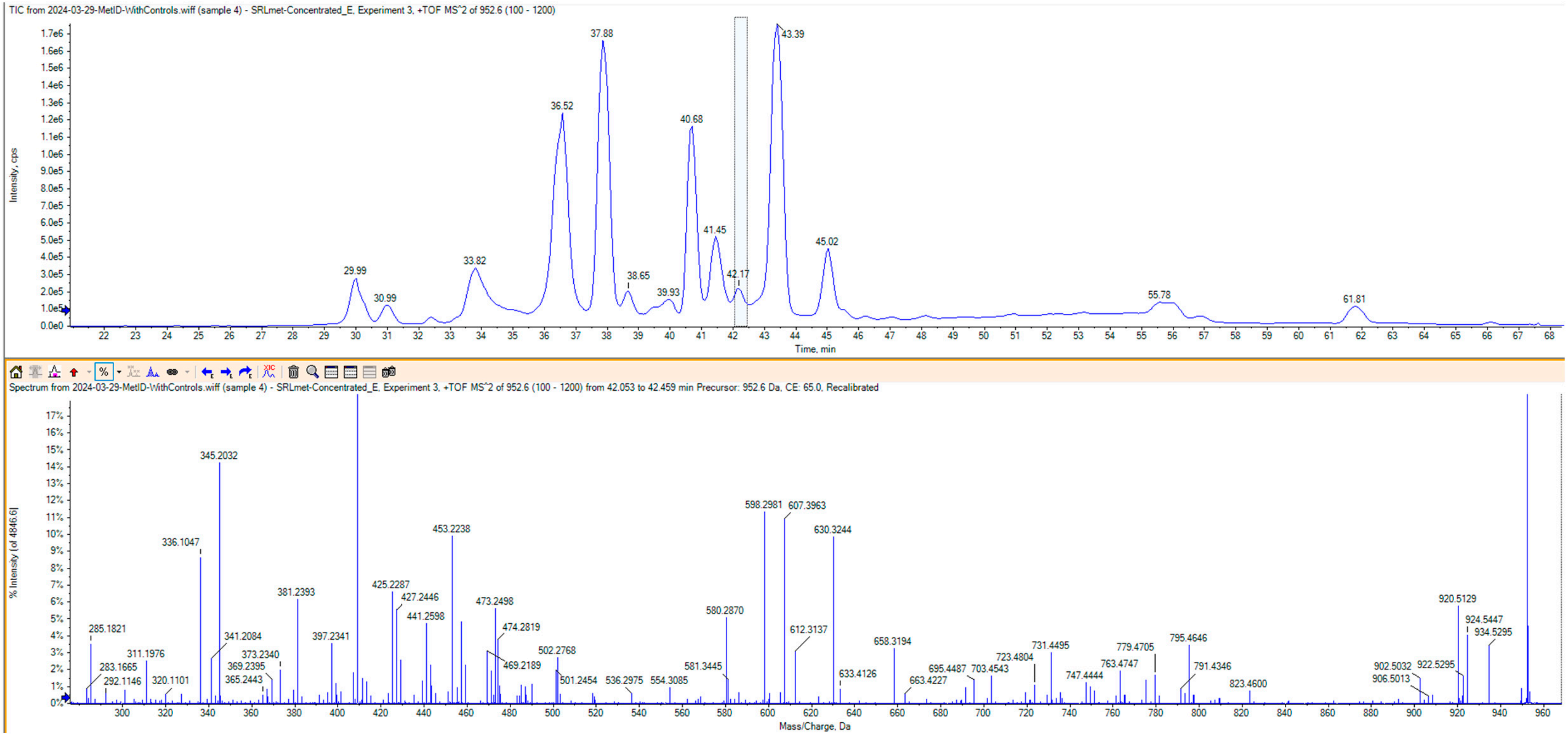

Piperidine-Hydroxy Sirolimus Chromatogram (Top)
Mass Spectrum, QTOF Fragmentation, (952.5393 Extracted) (Bottom)

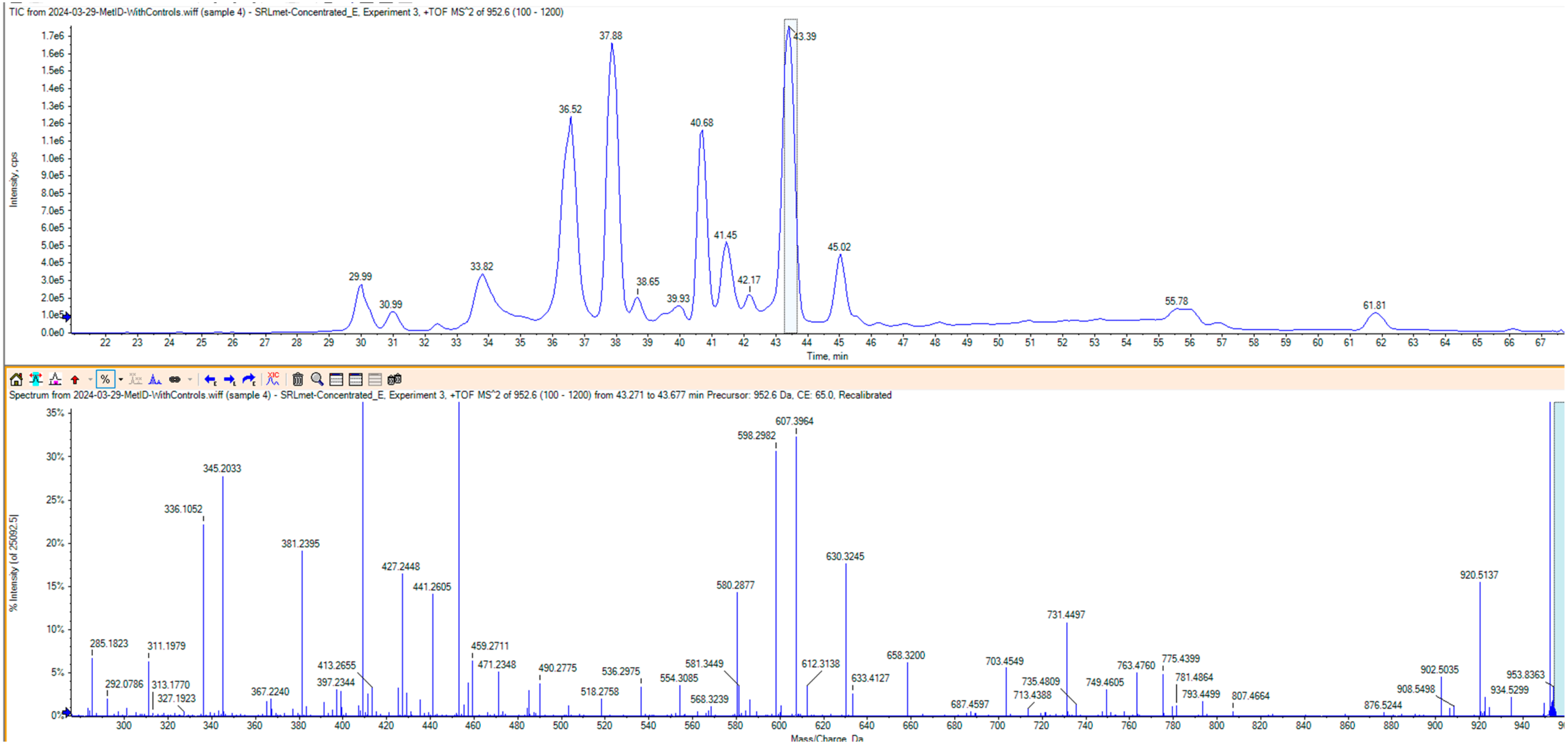

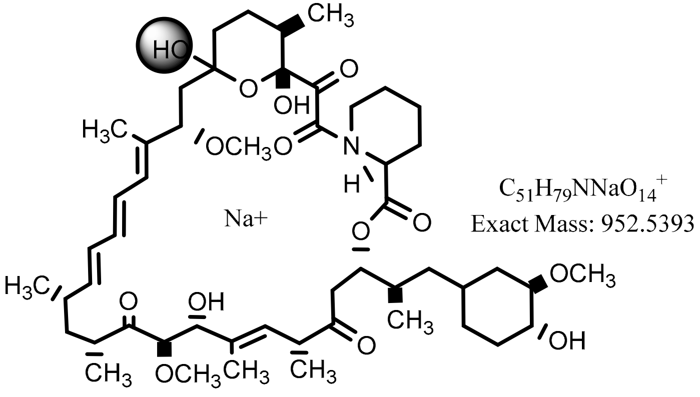

14-OH Sirolimus

14-Hydroxy Sirolimus (*m/z* = 952.5393)

Hydroxy Sirolimus Metabolites
Total Ion Chromatogram, *m/z* = 952.0

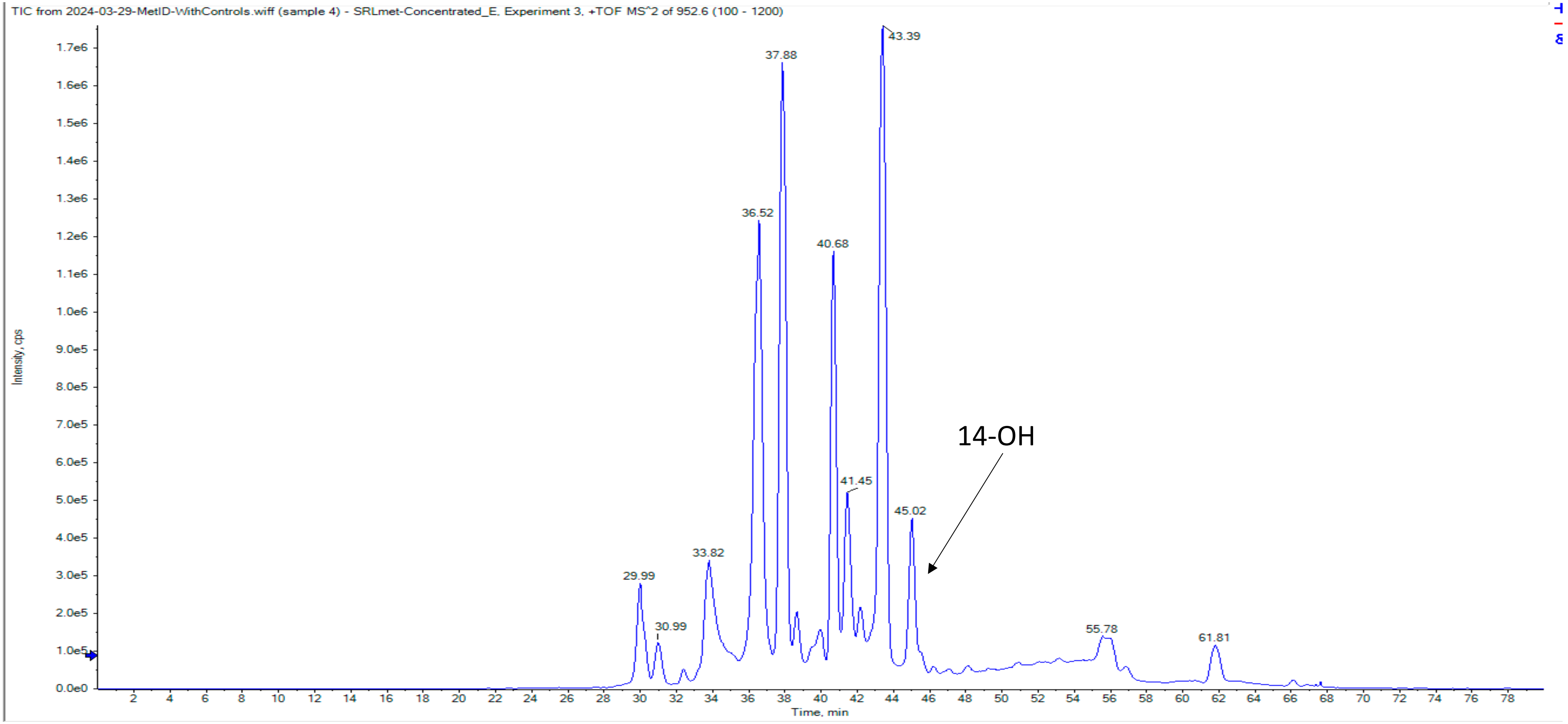

14-Hydroxy Sirolimus Chromatogram (Top)
Mass Spectrum, QTOF Fragmentation, (952.5393 Extracted) (Bottom)

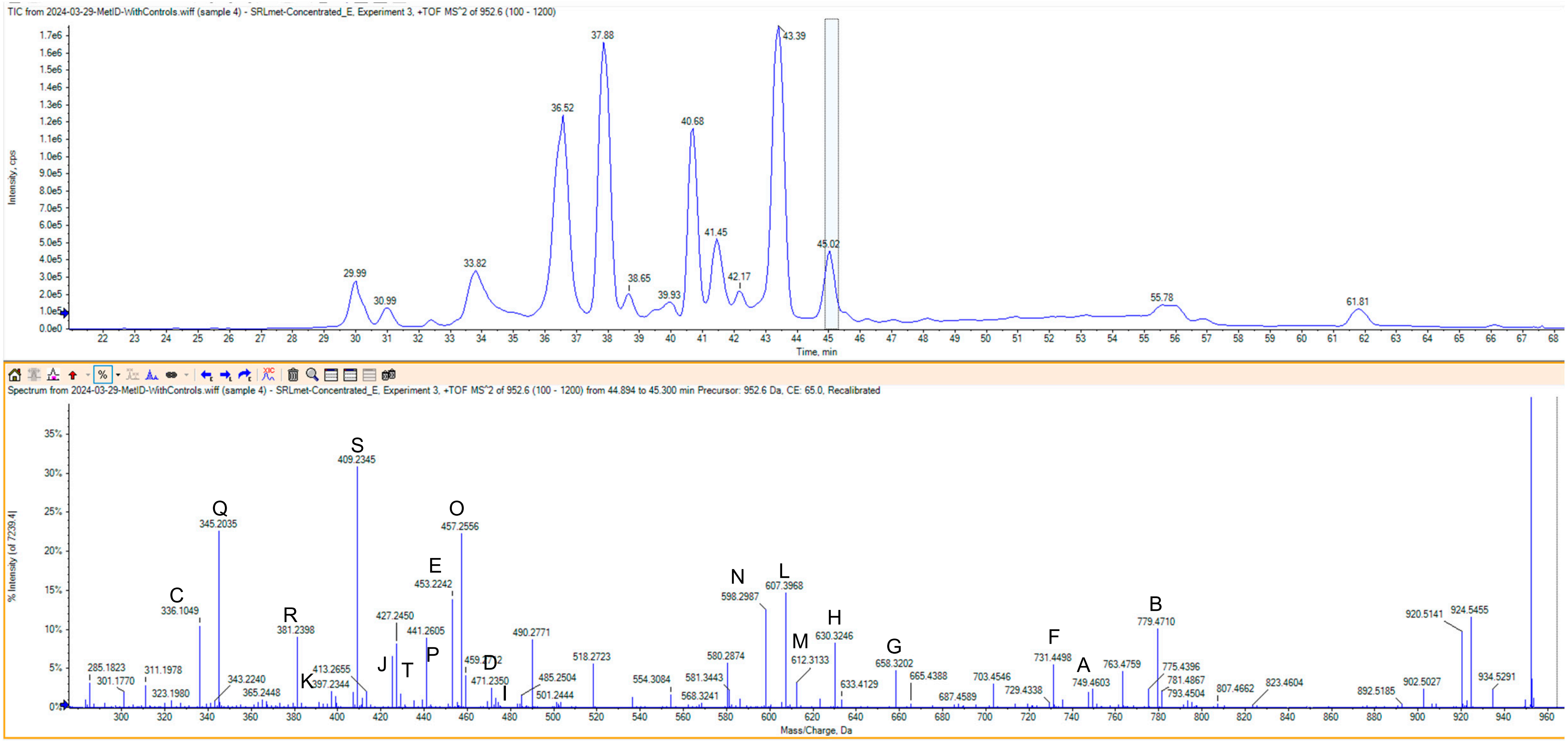

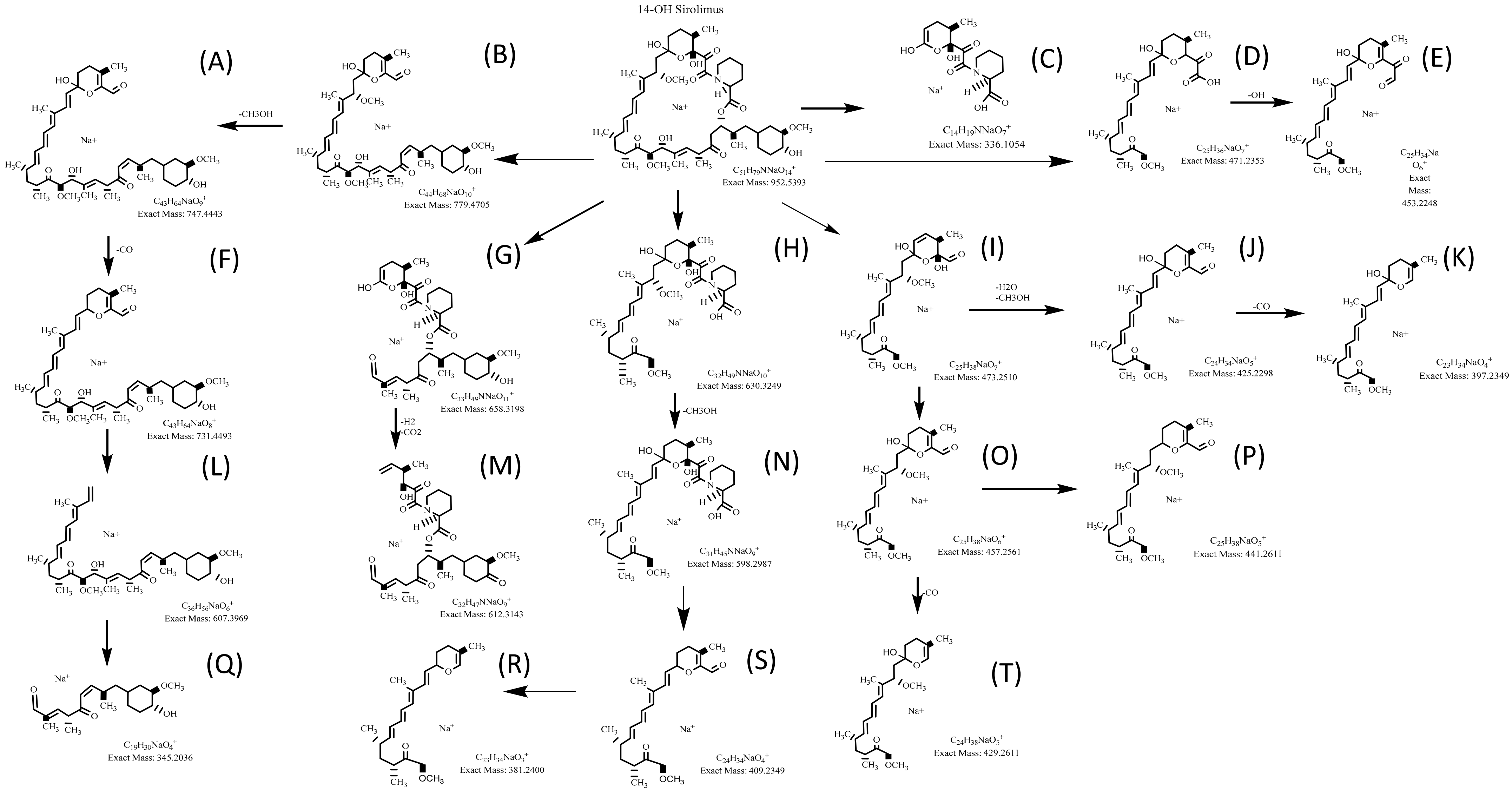
14-OH Sirolimus
C51H79NNaO14+
Exact Mass: 952.5393
(A)
C43H64NaO9+
Exact Mass: 747.4443
-CH3OH
(B)
C44H68NaO10+
Exact Mass: 779.4705
(C)
C14H19NNaO7+
Exact Mass: 336.1054
(D)
C25H36NaO7+
Exact Mass: 471.2353
-OH
(E)
C25H34Na O6+
Exact Mass: 453.2248
-CO
(F)
C43H64NaO8+
Exact Mass: 731.4493
(G)
C33H49NNaO11+
Exact Mass: 658.3198
-H2
-CO2
(H)
C32H49NNaO10+
Exact Mass: 630.3249
-CH3OH
(I)
C25H38NaO7+
Exact Mass: 473.2510
-H2O
-CH3OH
(J)
C24H34NaO5+
Exact Mass: 425.2298
-CO
(K)
C23H34NaO4+
Exact Mass: 397.2349
(L)
C36H56NaO6+
Exact Mass: 607.3969
(M)
C32H47NNaO9+
Exact Mass: 612.3143
(N)
C31H45NNaO9+
Exact Mass: 598.2987
(O)
C25H38NaO6+
Exact Mass: 457.2561
(P)
C25H38NaO5+
Exact Mass: 441.2611
-CO
(Q)
C19H30NaO4+
Exact Mass: 345.2036
(R)
C23H34NaO3+
Exact Mass: 381.2400
(S)
C24H34NaO4+
Exact Mass: 409.2349
(T)
C24H38NaO5+
Exact Mass: 429.2611

Characteristic Fragments of 14-OH Sirolimus

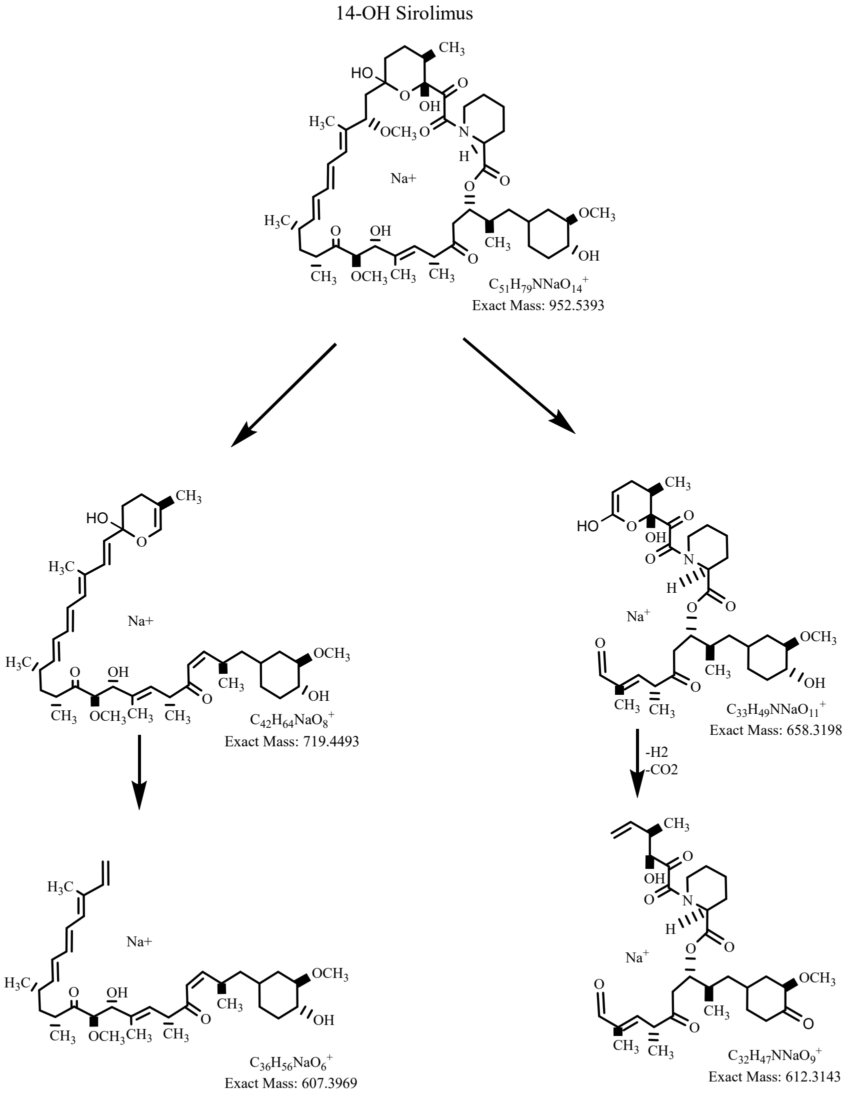

Δppm of 14-Hydroxy Sirolimus Fragments

	**Theoretical mass**	**Measured mass**	**Δppm**
**14 -OH Sirolimus**	952.5393	952.5393	0.0
A	747.4443	747.4453	1.3
B	779.4705	779.4710	0.6
C	336.1054	336.1049	1.5
D	469.2197	469.2187	2.1
E	453.2248	453.2242	1.3
F	731.4493	731.4498	0.7
G	658.3198	658.3202	0.6
H	630.3249	630.3246	0.5
I	473.2510	473.2503	1.5
J	425.2298	425.2290	1.9
K	397.2349	397.2344	1.3
L	607.3969	607.3968	0.2
M	612.3143	612.3133	1.6
N	598.2987	598.2987	0.0
O	457.2561	457.2556	1.1
P	441.2611	441.2605	1.4
Q	345.2036	345.2035	0.3
R	381.2400	381.2398	0.5
S	409.2349	409.2345	1.0
T	427.2455	427.2450	1.2

14-Hydroxy Sirolimus Comments

Assignment fragment<br>14 -OH Sirolimus	Sirolimus	14-OH	Comment
A	731.4	747.4	Excludes piperidine-OH, Rest are possible
B	763.5	779.5	Excludes piperidine-OH, Rest are possible
C	320.1(Q)	336.1	Possible 11,12,14,piperidine-OH
D	453.2(N)	469.2	Excludes piperidine-OH,49-OH, Rest are possible
E	ND	453.2	Excludes piperidine-OH,49-OH, Rest are possible
F	703.4 (E)	719.5	Excludes piperidine-OH, Rest are possible
G	642.3(C)	658.3	Excludes 24,25,46-OH
H	614.3(G)	630.3	Excludes 49-OH
I	459.2(I)	473.3	Possible 11,12,14,24,25,46-OH
J	409.2(J)	425.2	Possible 11,12,14,24,25,46-OH
K	ND	397.2	Possible 11,12,14,24,25,46-OH
L	607.3(K)	607.4	Excludes23,24,25,46,49-OH
M	ND	612.3	Excludes piperidine,11,12,49-OH
N	582.3(M)	598.3	Excludes 49-OH
O	441.2(O)	457.3	Excludes piperidine-OH,49-OH
P	ND	439.2	Excludes piperidine-OH,49-OH
Q	345.2(D)	345.2	Excludes 49-OH
R	381.2(P)	381.2	Excludes piperidine-OH,49-OH
S	409.2(J)	409.2	Excludes piperidine-OH,49-OH
T	413.2(R)	429.3	Excludes piperidine-OH,49-OH
**L**		**607.4**	**Characteristic fragment**
**M**		**612.3**	**Characteristic fragment**

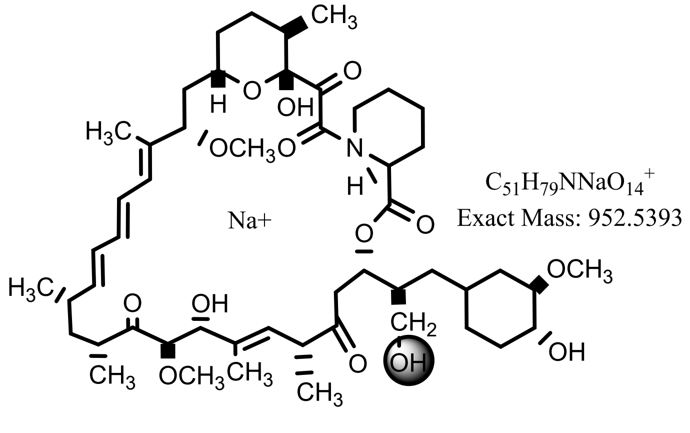

49-OH Sirolimus

49-Hydroxy Sirolimus (*m/z* = 952.5393)

Hydroxy Sirolimus Metabolites
Total Ion Chromatogram, m/z = 952.0

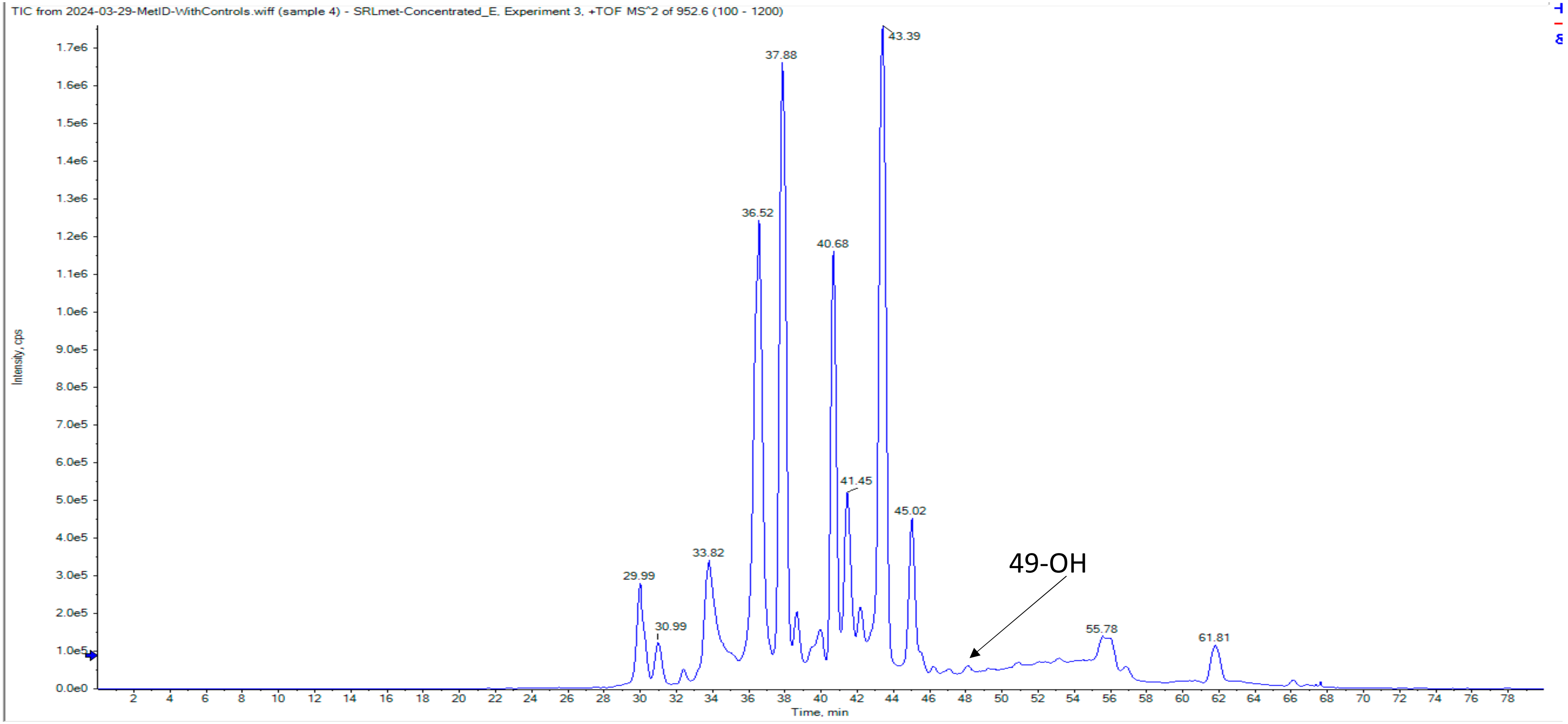

49-Hydroxy Sirolimus Chromatogram (Top)
Mass Spectrum, QTOF Fragmentation, (952.5393 Extracted) (Bottom)

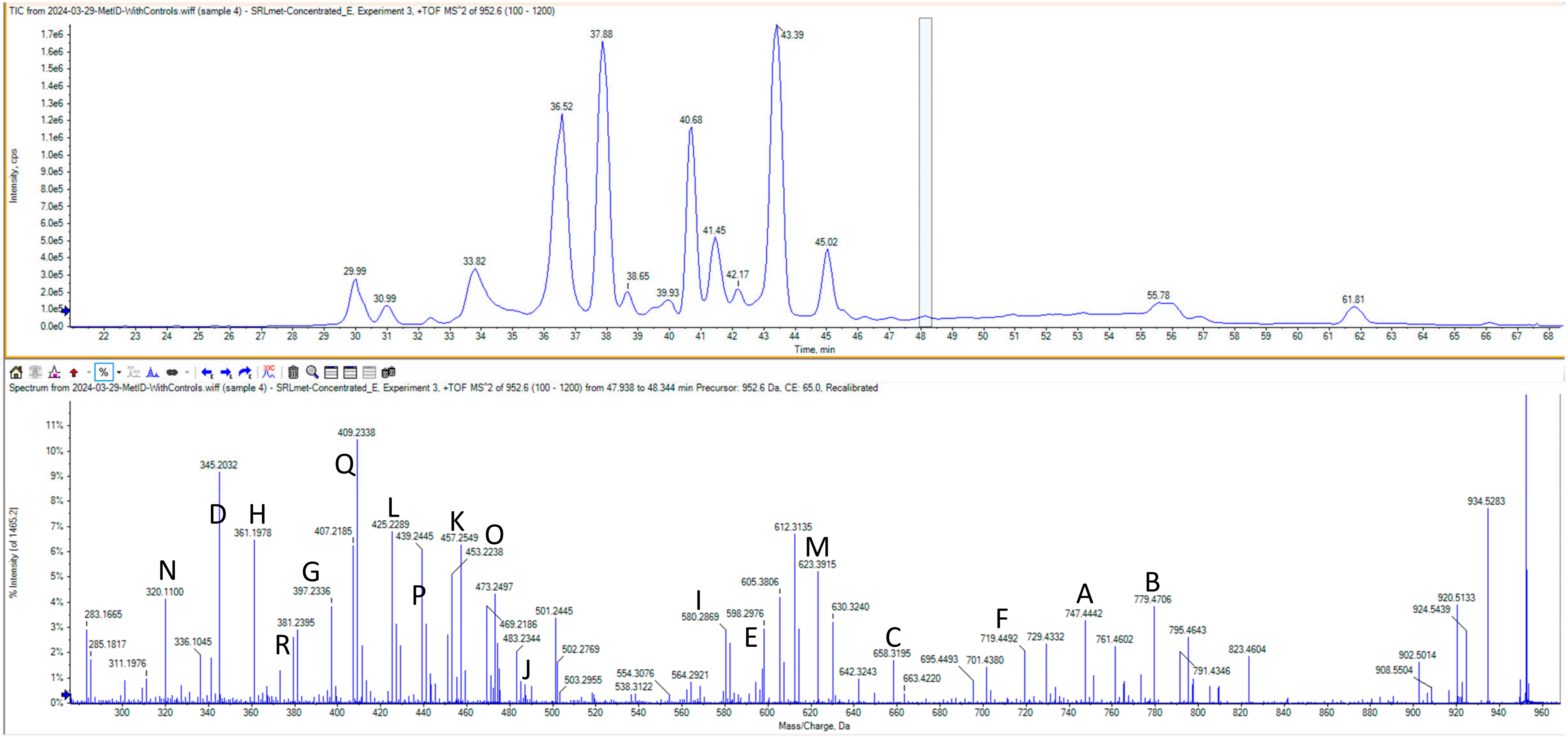

49-Hydroxy Fragmentation Pattern

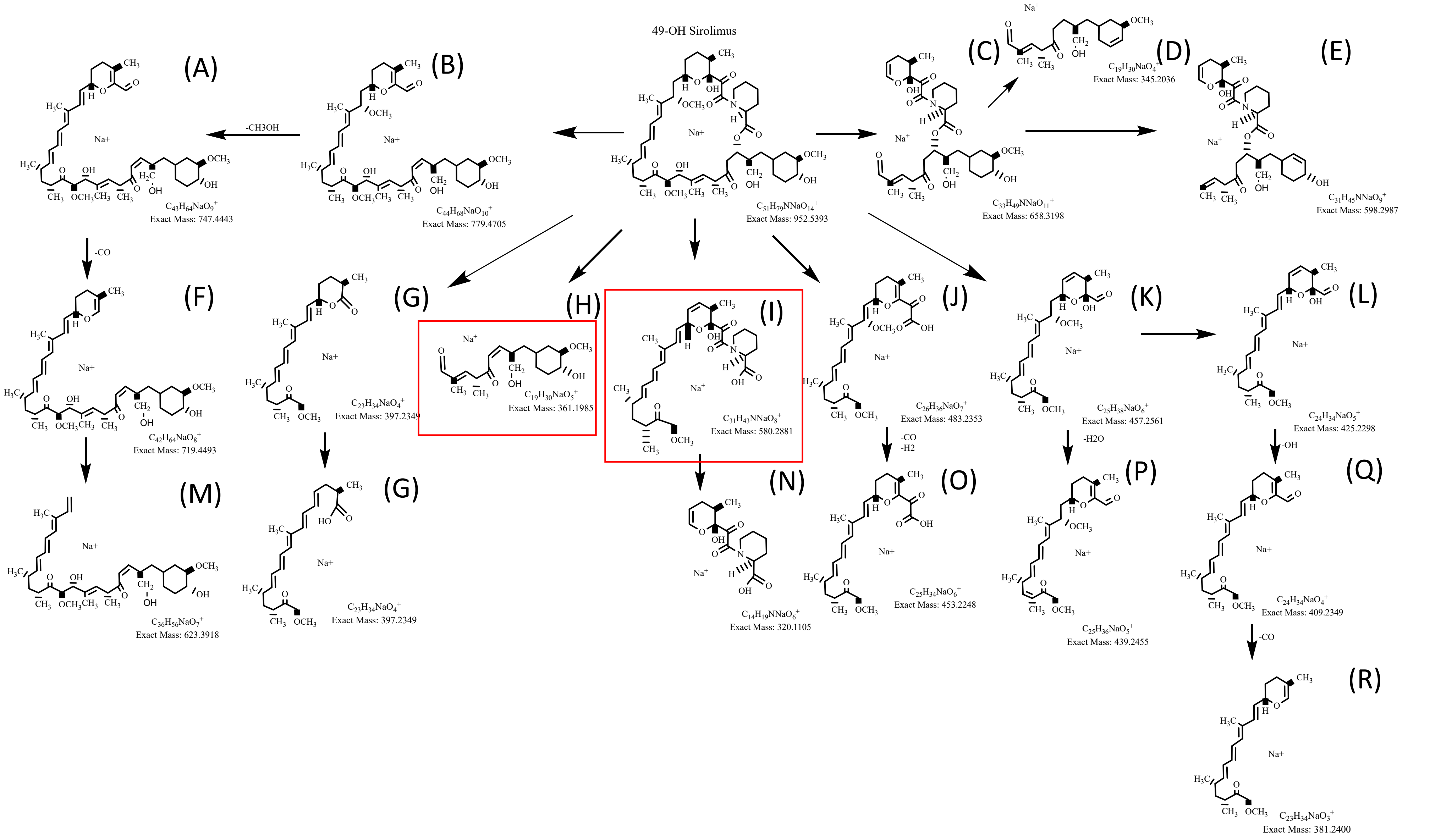

Characteristic Fragments of 49-OH Sirolimus

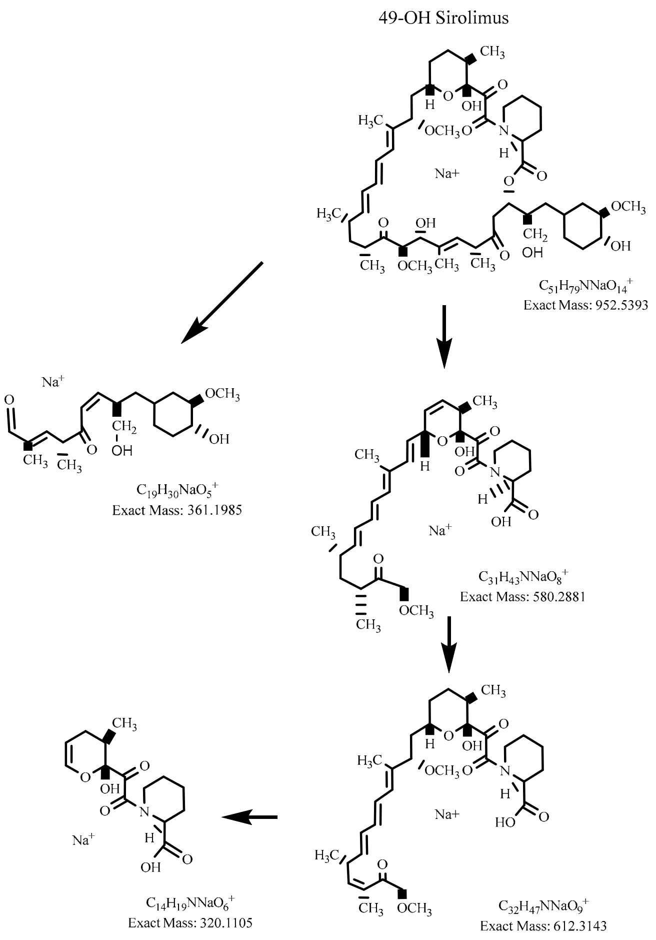

Δppm of 49-Hydroxy Sirolimus Fragments

	Theoretical mass	Measured mass	Δppm
**49 -OH Sirolimus**	952.5393	952.5393	0.0
A	747.4443	747.4442	0.1
B	779.4705	779.4706	0.2
C	658.3198	658.3195	0.4
D	345.2036	345.2032	1.2
E	598.2987	598.2976	1.8
F	719.4493	719.4492	0.2
G	397.2349	397.2336	3.3
H	361.1985	361.1978	1.9
I	580.2881	580.2869	2.1
J	483.2353	483.2344	1.9
K	457.2561	457.2549	2.6
L	425.2298	425.2289	2.1
M	623.3918	623.3915	0.5
N	320.1105	320.1100	1.6
O	453.2248	453.2238	2.2
P	439.2455	439.2445	2.3
Q	409.2349	409.2338	2.7
R	381.2400	381.2395	1.3

49-Hydroxy Sirolimus Comments

Assingment fragment 49 -OH Sirolimus	Sirolimus	49-OH	Comment
A	731.4	747.4	Excludes piperidine-OH, rest are possible
B	763.5	779.5	Excludes piperidine-OH, rest are possible
C	642.3	658.3	Excludes 23,24,25,46-OH
D	345.2	345.2	Possible 49-OH
E	ND	598.3	Excludes 23,24,25,46-OH
F	703.4(E)	719.5	Excludes piperidine-OH, rest are possible
G	397.2(L)	397.2	Excludes 11,12,14,23,24,25,46--OH
H	345.2(D)	361.2	Confirms 49-OH
I	582.3(M)	580.3	Excludes all but 49-OH, confirms 49-OH
J	485.2(H)	483.2	Excludes 11,12,14,23,24,25,46-OH
K	459.2(I)	457.3	Excludes 11,12,14,23,24,25,46-OH
L	ND	425.2	Excludes 11,12,14,23,24,25,46-OH
M	607.3(K)	623.4	Excludes OH-piperidine,11,12,14-OH
N	320.1(Q)	320.1	Excludes piperidine-OH
O	453.2(N)	453.2	Excludes 11,12,14,23,24,25,46-OH
P	441.2(O)	439.2	Excludes 11,12,14,23,24,25,46-OH
Q	409.2(J)	409.2	Excludes 11,12,14,23,24,25,46-OH
R	381.2(P)	381.2	Excludes 11,12,14,23,24,25,46-OH
**H**		**361.2**	**Characteristic fragment**
**I**		**580.3**	**Characteristic fragment**

Dihydroxy Sirolimus Metabolites

m/z = 968.5342

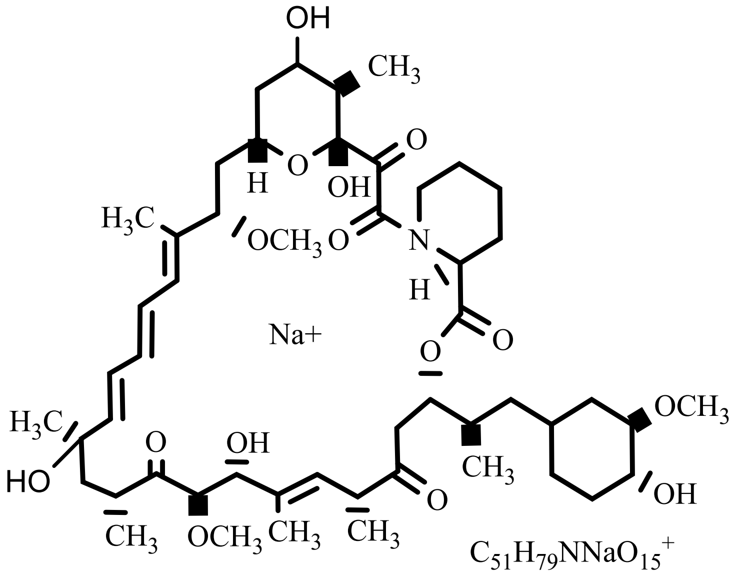

$C_{51}H_{79}NNaO_{15}^{+}$
Exact Mass: 968.5342

12, 23-OH Sirolimus

12,23-OH Dihydroxy Sirolimus (*m/z* = 968.5342)

12,23/24-Dihydroxy Sirolimus Chromatogram (Top)
Mass Spectrum, QTOF Fragmentation, (968.5342 Extracted) (Bottom)

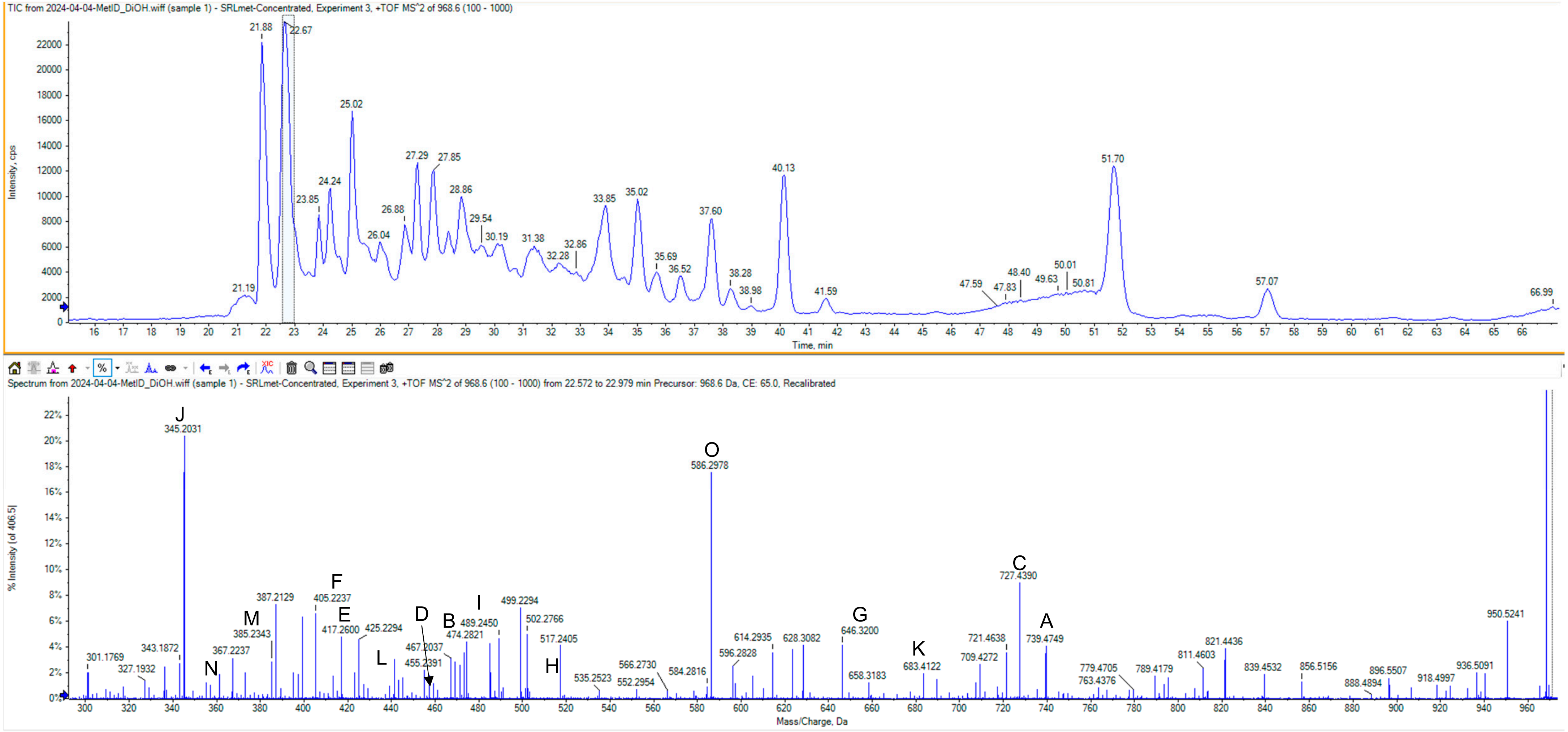

12, 23/24-Dihydroxy FragmentationPattern (12,24 Dihydroxy SRL is shown)

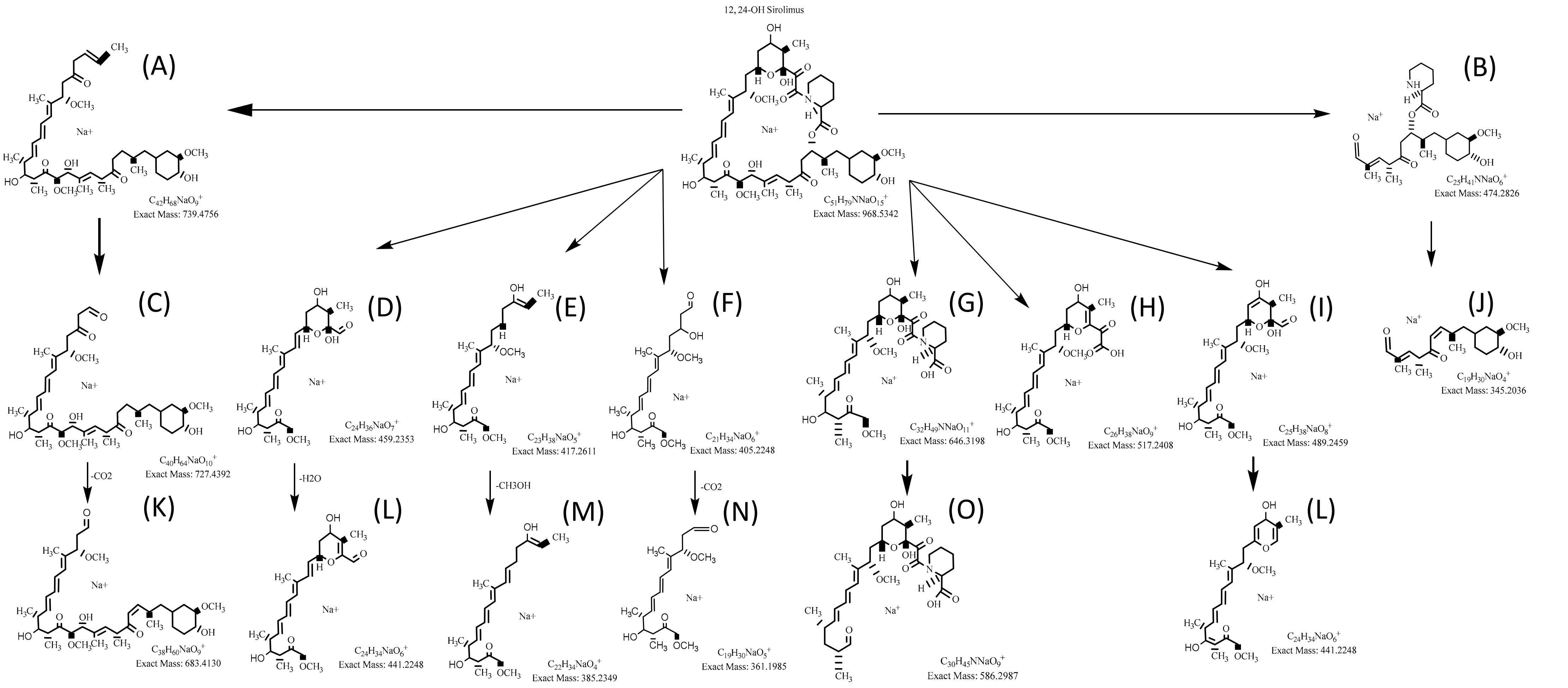

12,23/24-Dihydroxy FragmentationPattern (12,23 Dihydroxy SRL is shown)

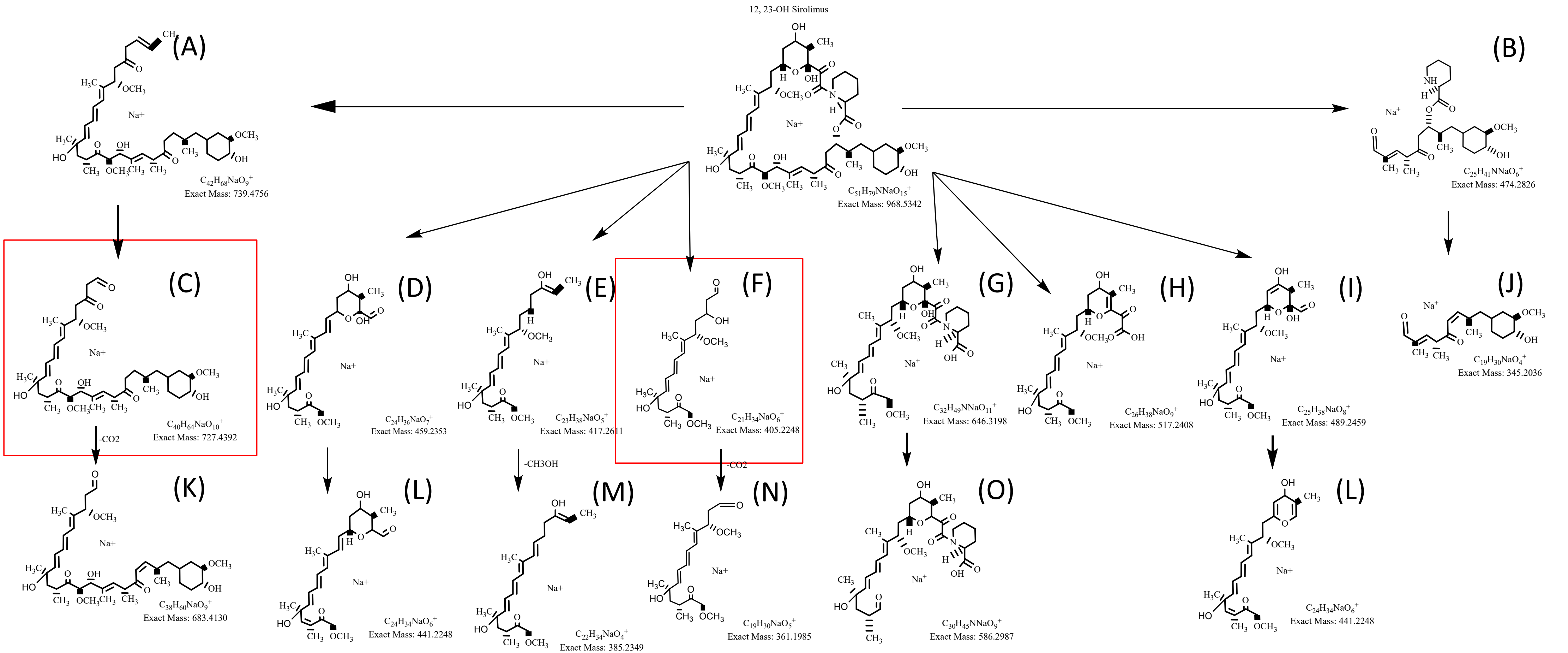

Δppm of 12,23/24-Dihydroxy Sirolimus Fragments

	Theoretical mass	Measured mass	∆ppm
**12-OH, 24-OH Sirolimus**	968.5342	968.5342	0.0
A	739.4756	739.4749	0.9
B	474.2826	474.2821	1.1
C	727.4392	727.4390	0.3
D	459.2353	459.2351	0.4
E	417.2611	417.2600	2.6
F	405.2248	405.2237	2.7
G	646.3198	646.3200	0.3
H	517.2408	517.2405	0.6
I	489.2459	489.2450	1.8
J	345.2036	345.2031	1.4
K	683.4130	683.4122	1.2
L	441.2248	441.2240	1.8
M	385.2349	385.2343	1.6
N	361.1985	361.1986	0.3
O	586.2987	586.2978	1.5

12,23/24-Dihydroxy Sirolimus Comments

Assignment fragments 12,24 - OH Dihydroxy Sirolimus	12,24 -OH Dihydroxy Sirolimus	Comment
A	739.5	Excludes piperidine-OH, rest are possible
B	474.3	Excludes49, piperidine-OH
C	727.4	Excludes piperidine-OH, rest are possible
D	459.2	Excludes49, piperidine-OH
E	417.3	Excludes piperidine-OH, rest are possible
F	405.2	Excludes49, piperidine-OH
G	646.3	Excludes 49-OH
H	517.2	Excludes49, piperidine-OH
I	489.2	Excludes49, piperidine-OH
J	345.2	Excludes 49-OH
K	683.4	Possible 24,25,46,49-OH
L	441.2	Excludes49, piperidine-OH
M	385.2	Excludes49, piperidine-OH
N	361.2	Possible 24,25,46-OH
O	586.2	Excludes49, piperidine-OH
**C,F**	(711.4+16 and 389.2+16)	**12-OH characteristic fragment was present**
		**All other fragment suggest 24-OH**

Hydroxy, O-Desmethyl Sirolimus Metabolites

m/z = 938.5236

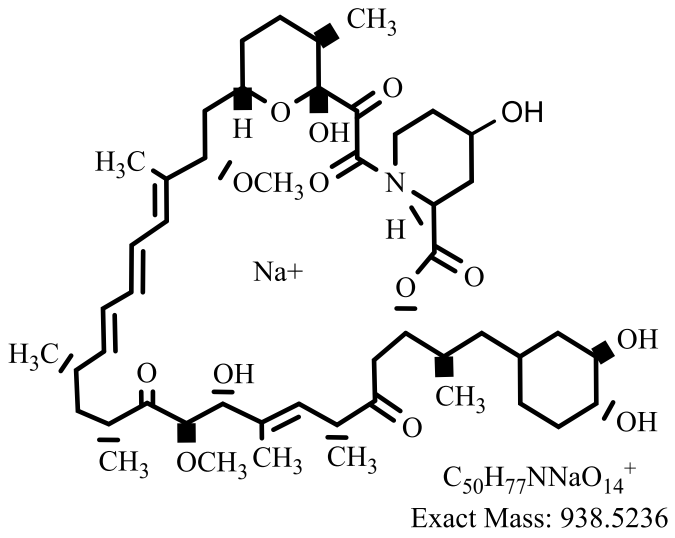

Piperidine-Hydroxy, 39-O-Desmethyl Sirolimus (*m/z* = 938.5236)

Hydroxy-piperidine, 39-O-Desmethyl Sirolimus Chromatogram (Top) Mass Spectrum, QTOF Fragmentation, (938.5236 Extracted) (Bottom)

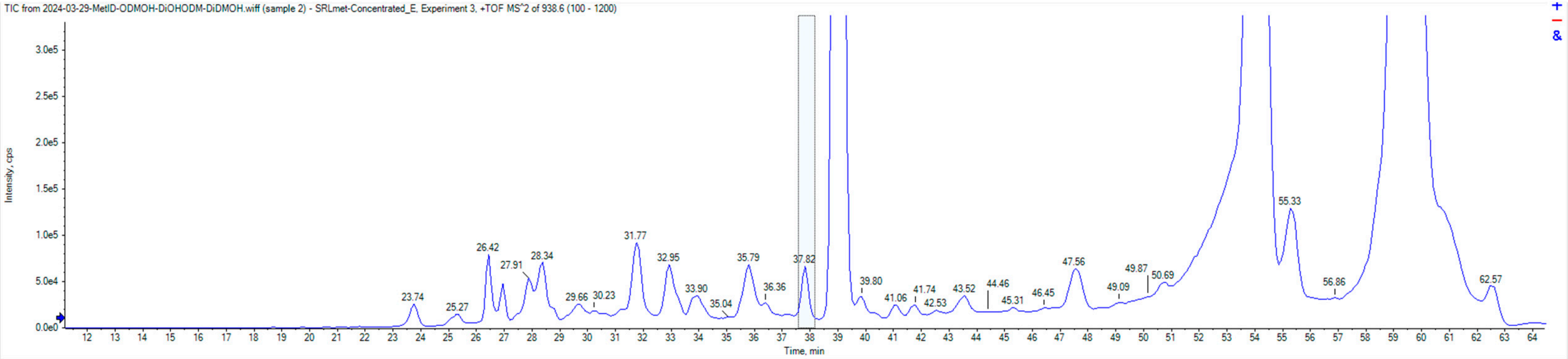

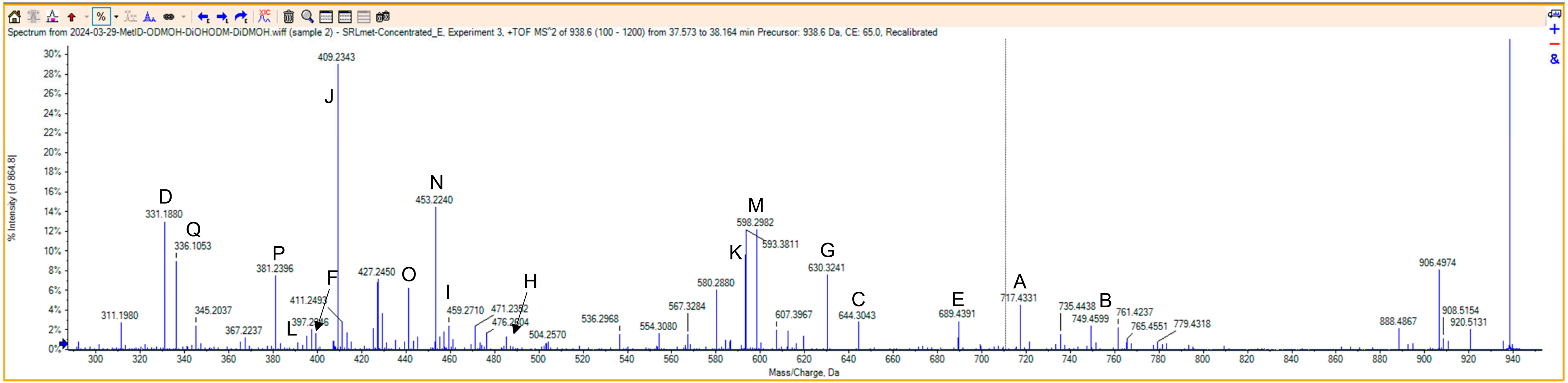

Piperidine-OH, 39-O-Desmethyl Fragmentation Pattern

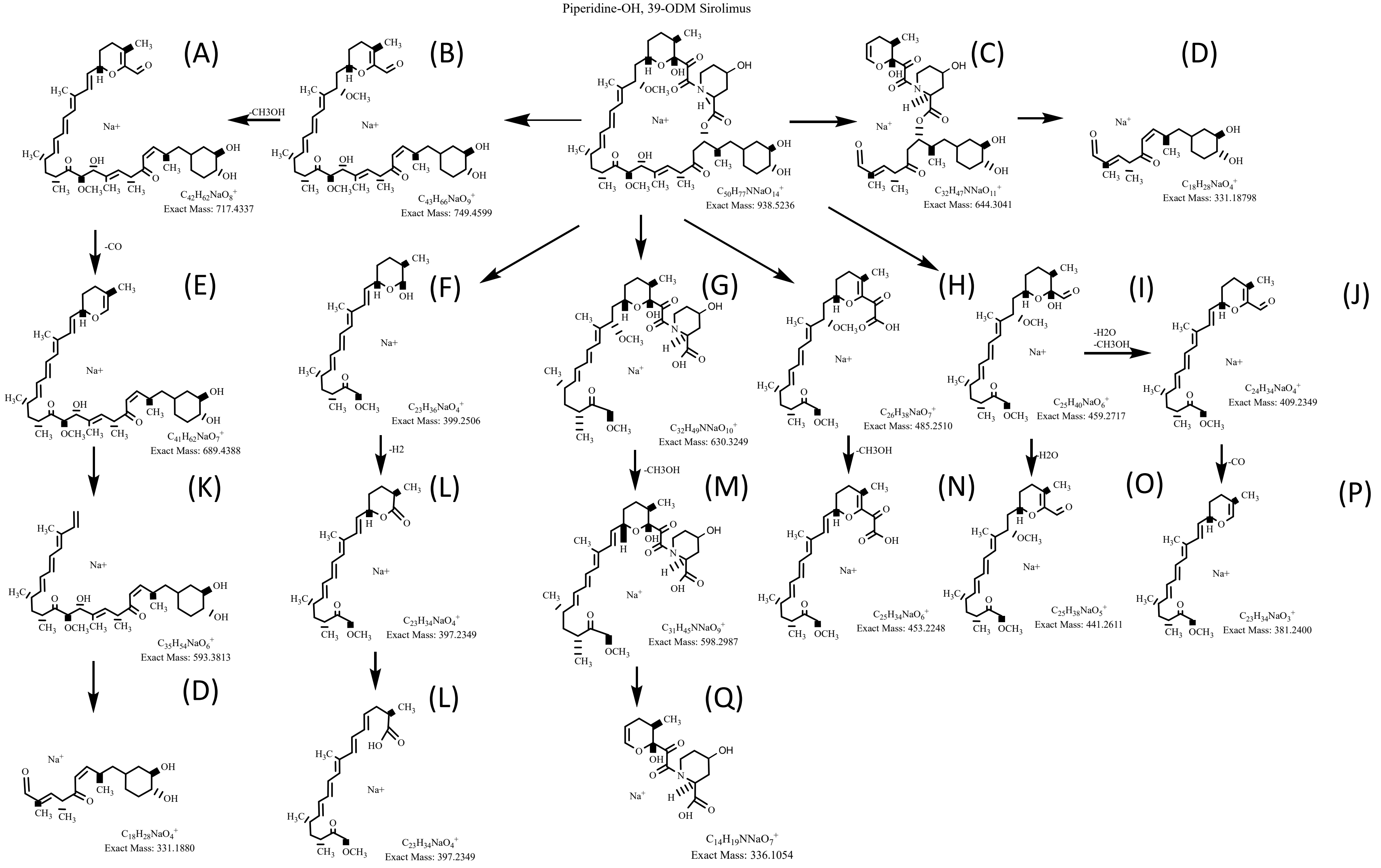

Δppm of Piperidine-OH,39-ODM Sirolimus Fragments

	Theoretical mass	Measured mass	Δppm
**Piperidine-OH, 39-ODM Sirolimus**	938.5236	938.5236	0.0
A	717.4337	717.4331	0.8
B	749.4599	749.4599	0.0
C	644.3041	644.3043	0.3
D	331.1880	331.1880	0.1
E	689.4388	689.4391	0.5
F	399.2506	399.2499	1.7
G	630.3249	630.3241	1.2
H	485.2510	485.2513	0.7
I	459.2717	459.2710	1.5
J	409.2341	409.2343	0.5
K	593.3813	593.3811	0.3
L	397.2349	397.2346	0.8
M	598.2987	598.2982	0.8
N	453.2248	453.2240	1.7
O	441.2612	441.2609	0.6
P	381.2400	381.2396	1.1
Q	336.1054	336.1053	0.2

Piperidine-OH, 39-O-Desmethyl Sirolimus Comments

	**Piperidine-OH, 39-ODM**	**Comment**
**Piperidine-OH, 39-ODM Sirolimus**	938.5	
A	717.4	Confirms Piperidine-OH
B	749.5	Confirms Piperidine-OH
C	644.3	Confirms 39-ODM
D	331.2	Confirms 39-ODM
E	689.4	Confirms Piperidine-OH
F	399.3	Excludes 11,12,14,24,25,46-OH
G	630.3	Confirms 39-ODM
H	485.3	Confirms 39-ODM
I	459.3	Confirms 39-ODM
J	409.2	Confirms Piperidine-OH
K	593.4	Excludes 24,25,46-OH
L	397.2	Excludes 11,12,14,24,25,46-OH
M	598.3	Excludes 49-OH
N	453.2	Confirms Piperidine-OH
O	441.3	Confirms Piperidine-OH
P	381.2	Confirms Piperidine-OH
Q	336.1	Confirms Piperidine-OH
**D**	331.2	**39-ODM characteristic fragment**
**Q**	336.1	**OH-Piperidine characteristic fragment**

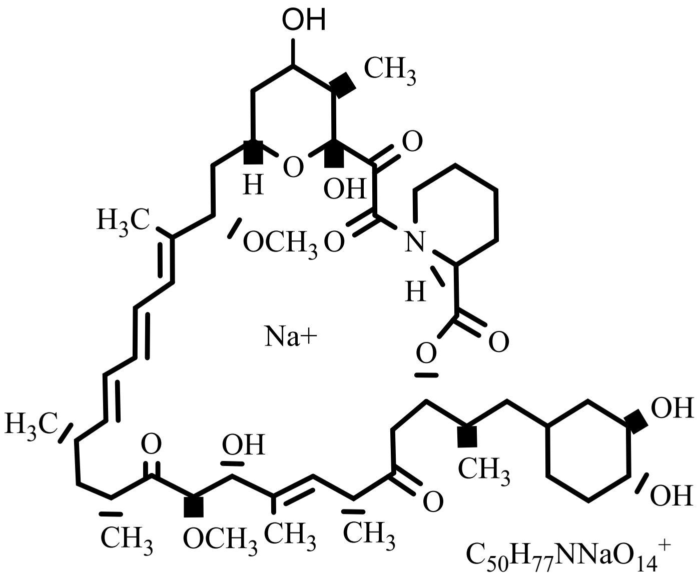

$C_{50}H_{77}NNaO_{14}^{+}$
Exact Mass: 938.5236

12-OH, 39-ODM Sirolimus

12-Hydroxy, 39-O-Desmethyl Sirolimus (*m/z* = 938.5236)

12-Hydroxy, 39-O-Desmethyl Sirolimus Chromatogram (Top) Mass Spectrum, QTOF Fragmentation, (938.5236 Extracted) (Bottom)

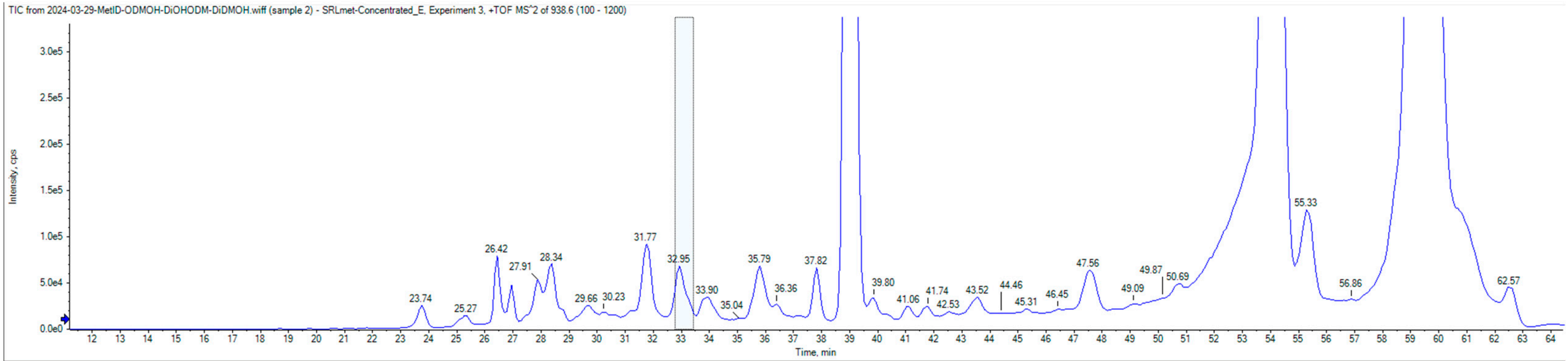

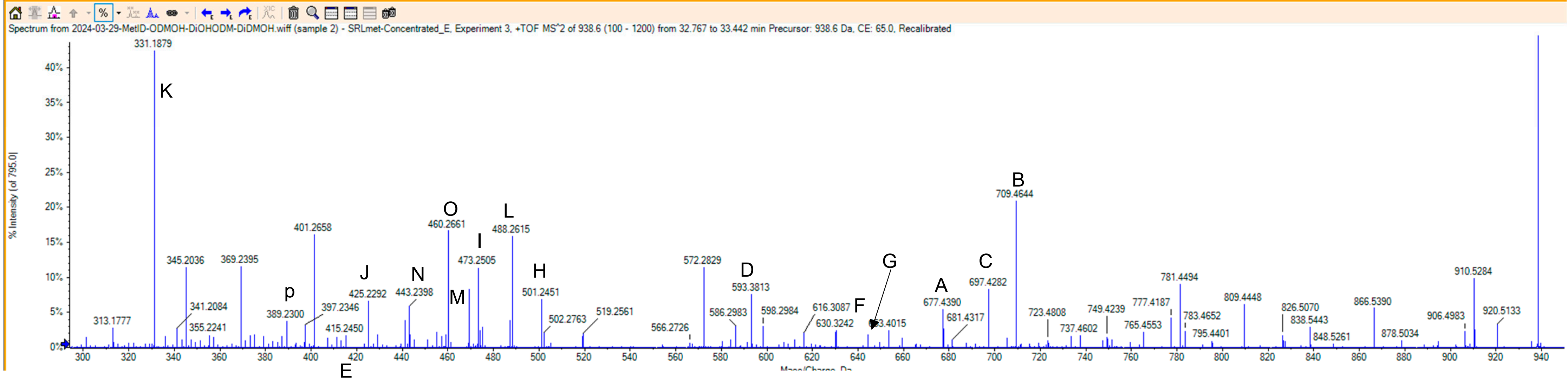

12-OH, 39-O-Desmethyl Fragmentation Pattern

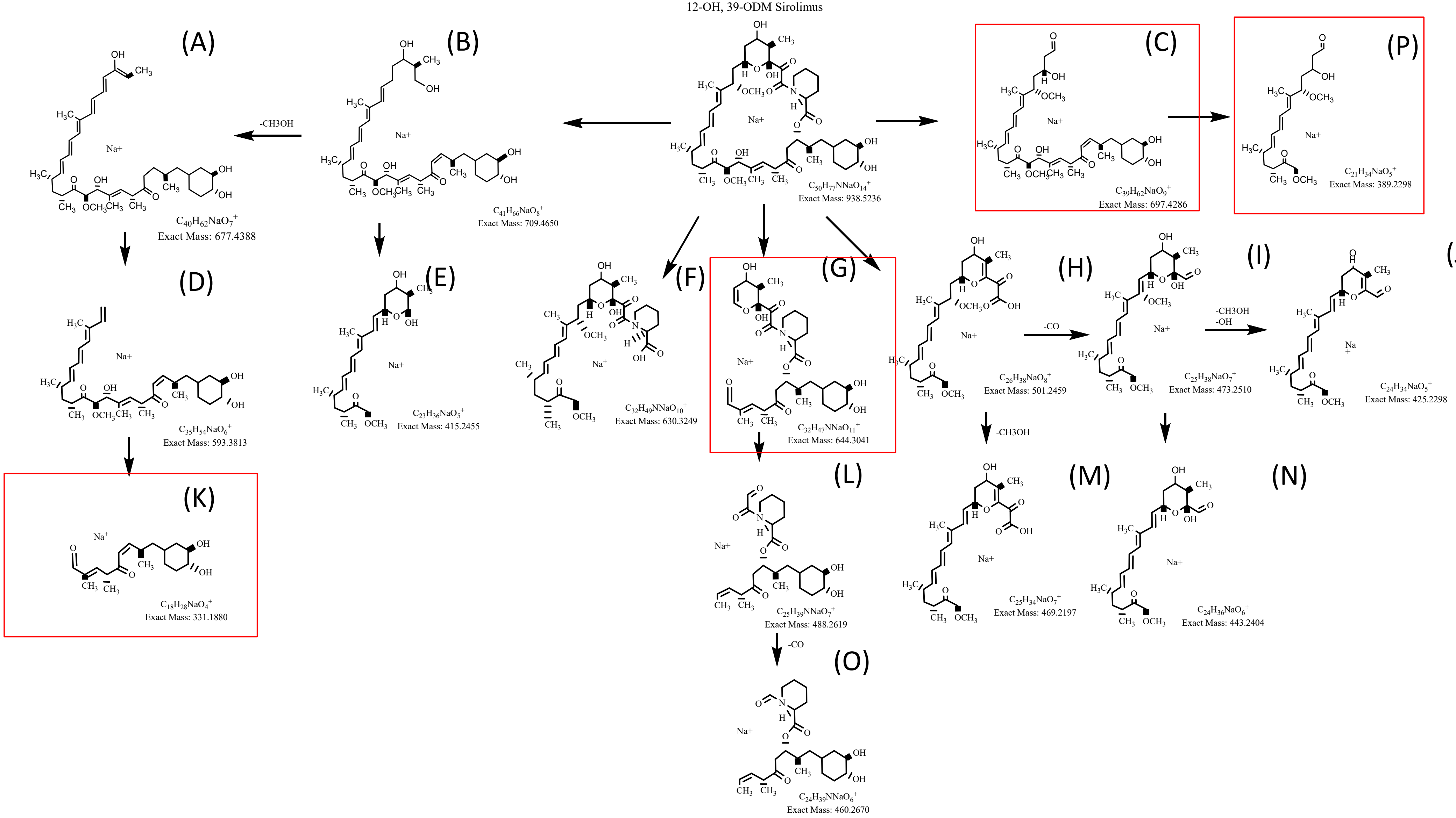

Δppm of 12-Hydroxy, 39-O-Desmethyl Sirolimus Fragments

	Theoretical mass	Measured mass	∆ppm
**12-OH, 39-ODM Sirolimus**	938.5236	938.5236	0.0
A	677.4388	677.4390	0.3
B	709.4650	709.4644	0.8
C	697.4286	697.4282	0.6
D	593.3813	593.3813	0.0
E	415.2455	415.2450	1.2
F	630.3249	630.3242	1.1
G	644.3041	644.3029	1.9
H	501.2459	501.2451	1.6
I	473.2510	473.2505	1.1
J	425.2298	425.2292	1.4
K	331.1880	331.1879	0.3
L	488.2619	488.2615	0.8
M	469.2197	469.2203	1.3
N	443.2404	443.2398	1.4
O	460.2670	460.2661	2.0
P	389.22980	389.23000	0.5

12-Hydroxy, 39-O-Desmethyl Sirolimus Comments

	Sirolimus	12-OH, 39-ODM	Comment
**12-OH, 39-ODM Sirolimus**	936.5	938.5	
A	ND	677.4	Excludes Piperidine-OH
B	703.5	709.5	Excludes Piperidine-OH
C	ND	697.4	Confirms 12-OH
D	607.4	593.4	Excludes11,12,14, Piperidine-OH
E	399.3	415.2	Excludes Piperidine-OH
F	614.3	630.3	Confirms 39-ODM
G	ND	644.3	Confirms 39-ODM
H	485.2	501.2	Confirms 39-ODM, excludes piperidine, 49-OH
I	459.2	473.3	Confirms 39-ODM, excludes piperidine, 49-OH
J	409.2	425.2	Excludes49, Piperidine-OH
K	345.2	331.2	Confirms 39-ODM
L	ND	488.3	Confirms 39-ODM
M	453.2	469.2	Excludes49, Piperidine-OH
N	441.3	443.2	Excludes49, Piperidine-OH
O	ND	460.3	Excludes49, Piperidine-OH
P	ND	389.2	Confirms 12-OH
**331 and 628.3 (39-ODM characteristic fragments are present)628.3 turned to 644.3 as the result of 12-OH**			
**389,711 (12-OH characteristic fragments)are present. 711 turned to 697.4 as the result of 39-ODM**			

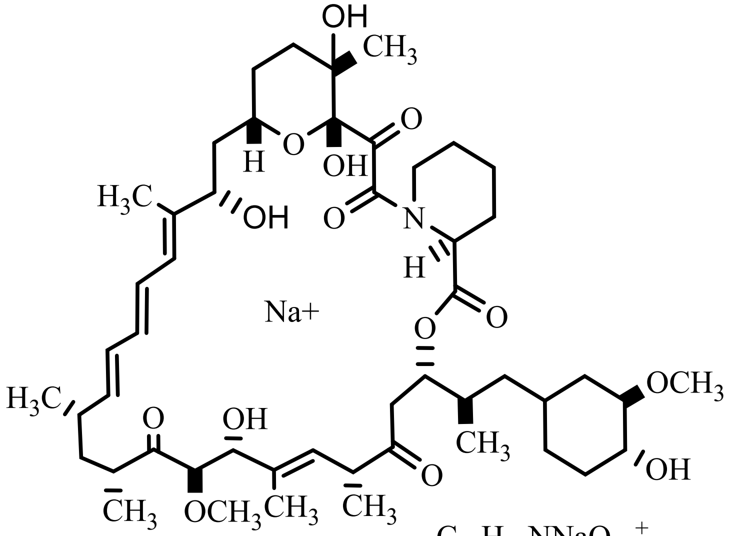

$C_{50}H_{77}NNaO_{14}^{+}$
Exact Mass: 938.5236

11-Hydroxy,16-O-Desmethyl Sirolimus (*m/z* = 938.5236)

11-Hydroxy, 16-O-Desmethyl Sirolimus Chromatogram (Top) Mass Spectrum, QTOF Fragmentation, (938.5236 Extracted) (Bottom)

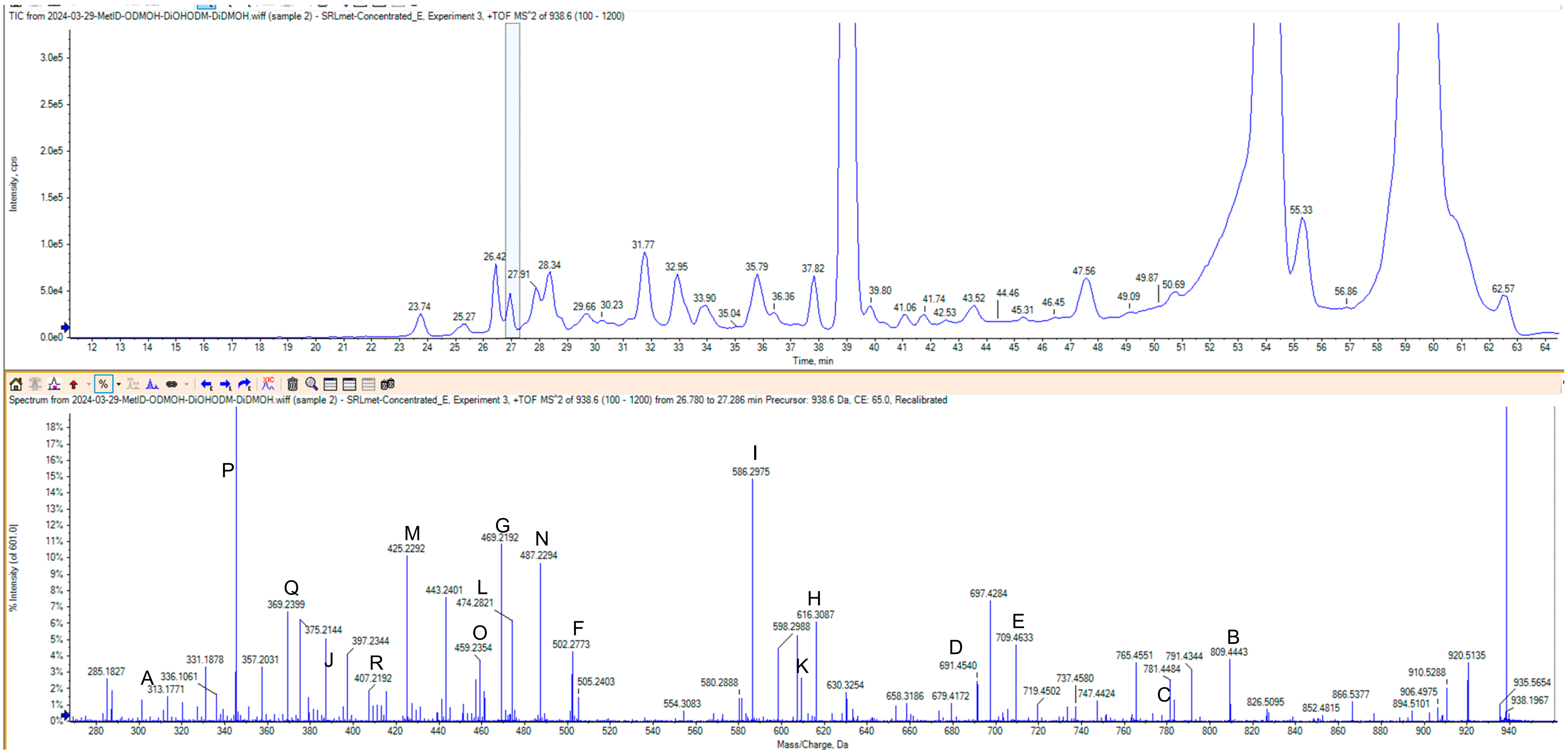

11-Hydroxy, 16-O-Desmethyl Sirolimus Fragmentation Pattern

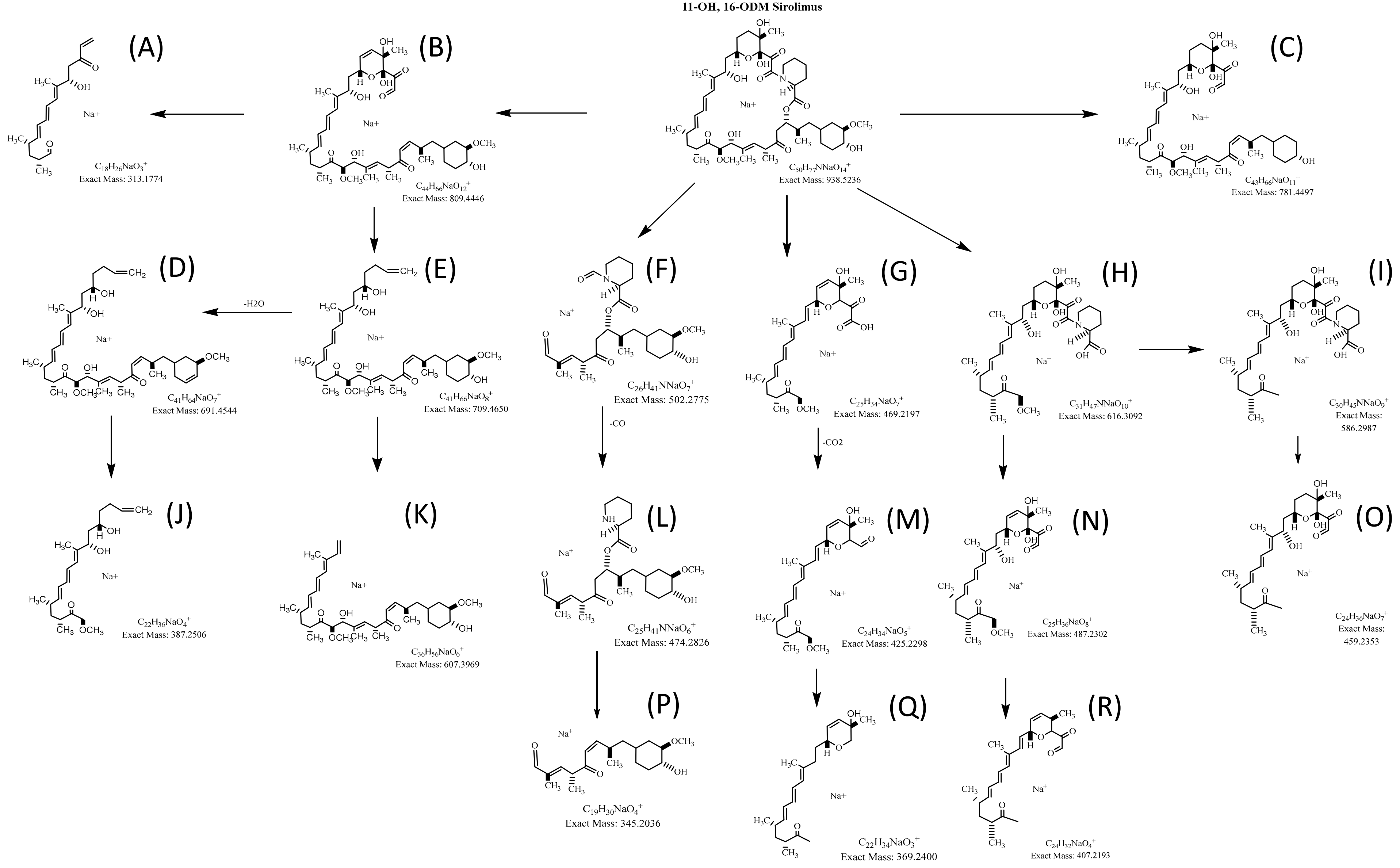

Δppm of 11-Hydroxy,16-O-Desmethyl Sirolimus Fragments

	Theoretical mass	Measured mass	∆ppm
**11-OH, 16-ODM**	938.5236	938.5236	0.0
A	313.1774	313.1771	1.0
B	809.4446	809.4443	0.4
C	781.4497	781.4484	1.7
D	691.4544	691.4540	0.6
E	709.465	709.4633	2.4
F	502.2775	502.2773	0.4
G	469.2197	469.2192	1.1
H	616.3092	616.3087	0.8
I	586.2987	586.2975	2.0
J	387.2506	387.2500	1.5
K	607.3969	607.3971	0.3
L	474.2826	474.2821	1.1
M	425.2298	425.2292	1.4
N	487.2302	487.2294	1.6
O	459.2353	459.2354	0.2
P	345.2036	345.2034	0.6
Q	369.24	369.2399	0.3
R	407.2193	407.2192	0.2

11-Hydroxy,16-O-Desmethyl Sirolimus Comments

	Sirolimus	11-OH, 16-ODM	Comment
**11-OH, 16-ODM**	936.5	938.5	
A	ND	313.2	Confirms16-ODM
B	ND	809.4	Excludesd piperidine-OH
C	763.5	781.4	Exclude piperidine-OH
D	ND	691.5	confirms 11-OH
E	ND	709.5	confirms 11-OH
F	ND	502.3	Excludes piperidine,49-OH,39ODM
G	453.2	469.2	Excludes 39-ODM and piperidine-OH
H	614.3	616.3	Excludes 39-ODM
I	582.3	586.3	Excludes 39-ODM
J	ND	387.3	confirms 11-OH
K	607.4	607.4	confirms 16-ODM
L	ND	474.3	Excludes 49-OH and piperidine-OH
M	409.2	425.2	Excludes piperidine,49-OH,39-ODM
N	485.3	487.2	Excludes piperidine,49-OH,39-ODM
O	ND	459.2	Excludes piperidine,49-OH,39-ODM
P	345.2	345.2	Excludes 49-OH,39ODM
Q	ND	369.2	Confirms16-ODM
R	ND	407.2	Confirms16-ODM
**723.3(11-Ohcharacteristic fragment is present) it turned to 709.5 as the result of 16-ODM**			
**Fragments A, K, Q, and R further confirm 16-ODM**			

Comparison of IUPAC vs Common Nomenclature of Sirolimus Structure

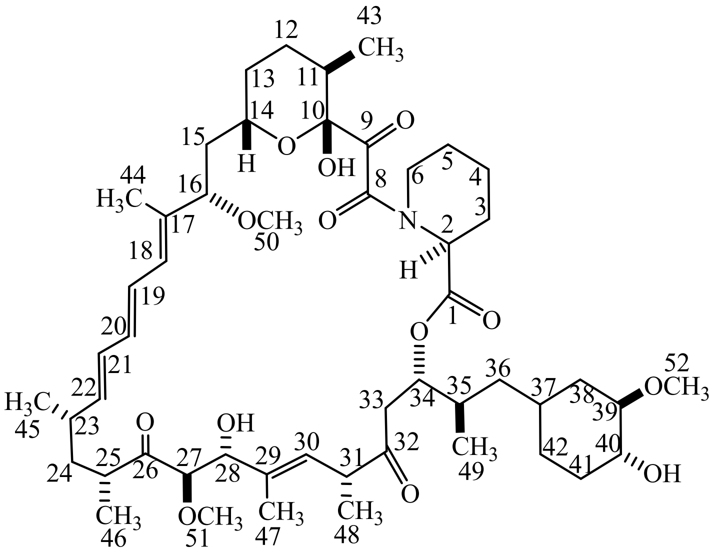

Structures of sirolimus.
Atom numbering follows the IUPAC (International Union of Pure and Applied Chemistry) nomenclature.

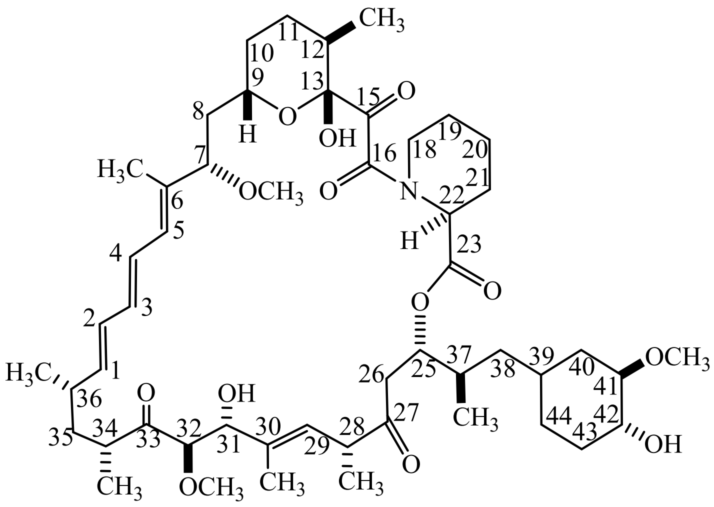

Structures of sirolimus.
Atom numbering follows the traditional nomenclature.

Comparison of IUPAC vs Common Nomenclature of Sirolimus Structure

IUPAC numenclature	Traditional Numenclature
45/46-Hydroxy sirolimus	47/48-Hydroxy sirolimus
23/24-Hydroxy sirolimus	35/36-Hydroxy sirolimus
12-Hydroxy sirolimus	11-Hydroxy sirolimus
25-Hydroxy sirolimus	34-Hydroxy sirolimus
11-Hydroxy sirolimus	12-Hydroxy sirolimus
Hydroxy Piperidine sirolimus	Hydroxy Piperidine sirolimus
14-Hydroxy sirolimus	9-Hydroxy sirolimus
49-Hydroxy sirolimus	45-Hydroxy sirolimus
16-O-Desmethyl sirolimus	7-O-Desmethyl sirolimus
27-O-Desmethyl sirolimus	32-O-Desmethyl sirolimus
39-O-Desmethyl sirolimus	41-O-Desmethyl sirolimus

Metabolites structurally identified in the present study based on IUPAC nomenclature	Metabolites detected in human blood sample or in vitro, labeled based on IUPAC nomenclature, NO back up data for structural identification	Metabolites identified in the previous studies based on Chemical Abstract numbering nomenclature
45/46-Hydroxy sirolimus	Detected/ Requires further structural identification	
23/24-Hydroxy sirolimus	Detected/ Reqires furthur strauctural identification	
12-Hydroxy sirolimus	Not identified/Not Detected	
25-Hydroxy sirolimus	Detected/ Requires further structural identification	
11-Hydroxy sirolimus	Detected/ Requires further structural identification	
Hydroxy Piperidine sirolimus	Detected in infant blood/Requires further structural identification	
14-Hydroxy sirolimus	Not identified/Not Detected	
49-Hydroxy sirolimus	Not identified/Not Detected	
(12,24)Dihydroxy sirolimus	Detected but not specified/Requires further structural identification	Detected but not specified
16-O-Desmethyl sirolimus	Not identified/Not Detected	7-O-Desmethyl sirolimus
27-O-Desmethyl sirolimus	Detected in vitro/Requires further structural identification	Not Detected
39-O-Desmethyl sirolimus	Detected/ Requires further structural identification	41-O-Desmethyl sirolimus
27,39-O-Didesmethyl sirolimus	Detected in vitro/Requires further structural identification	
16,39-O-Didesmethyl sirolimus	Detected in vitro/Requires further structural identification	
Piperidine-Hydroxy-39-O-desmethyl sirolimus	Not identified/Not Detected	
12-Hydroxy-39-O-desmethyl sirolimus	Not identified/Not Detected	
11-Hydroxy-16-O-desmethyl sirolimus	Not identified/Not Detected	

A brief comparison of the metabolites identified in this study, numbered according to IUPAC nomenclature, with previously identified metabolites, which are numbered based on both IUPAC nomenclature and the old numbering system.

Molecular Dynamic Simulations Hydroxylated Metabolites

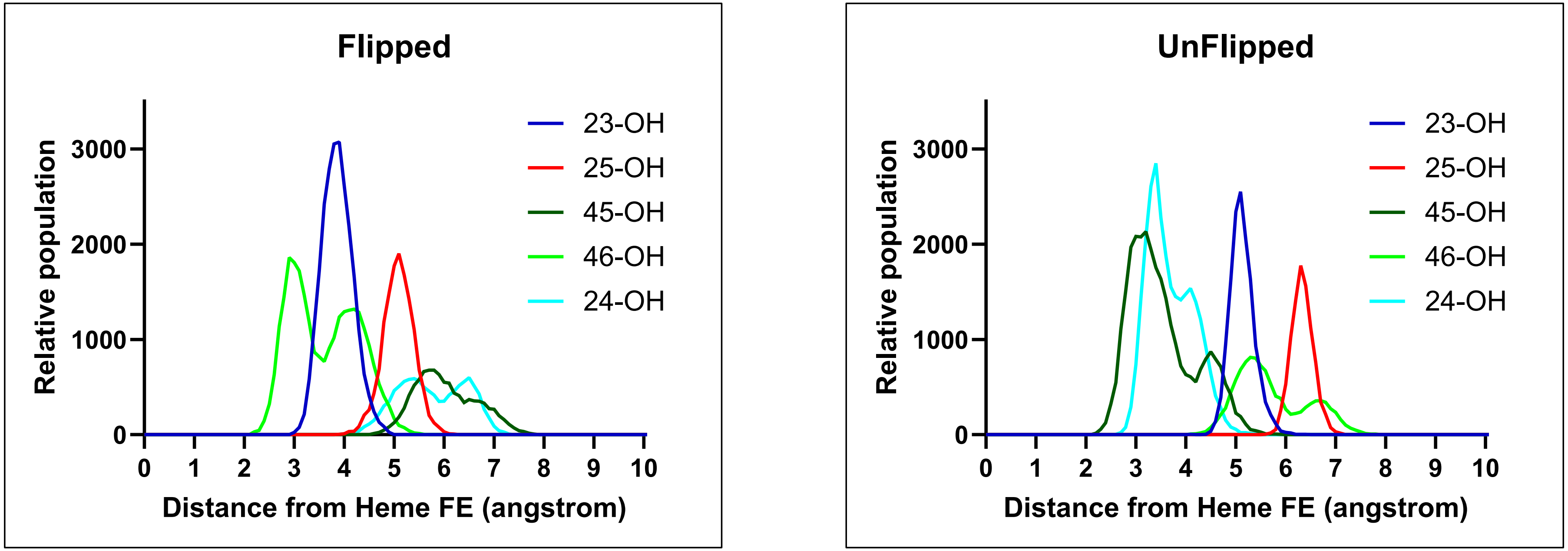

Distribution of the distance order for each site of metabolism according to the docking poses with the CYP3A4 Fe. Flipped orientation is chosen since it is decisive for the location of the functional group towards the heme.

Molecular Dynamic Simulations Demethylated Metabolites

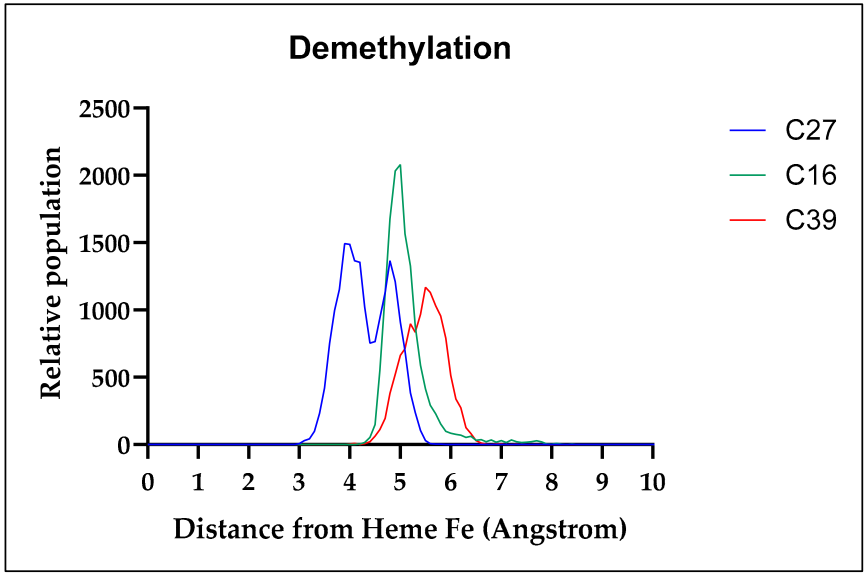

Comparison of Demethylation Site Distances from Heme (Fe):
A comparison of the distances of each demethylation site from the heme iron (Fe) suggests the following trend at first glance: C27 > C16 > C39
However, a more detailed analysis reveals that C27 exhibits a bimodal distribution, making its effective distance comparable to that of C16. This distribution indicates potential variability in accessibility due to molecular flexibility or enzyme dynamics.
Despite the apparent order of distances, C39 and C16 undergo demethylation at a higher rate than C27. This observation aligns with DFT (Density Functional Theory) calculations, which indicate that the Gibbs free energy (ΔG) favors demethylation at C39 and C16 over C27.
Additionally, it is important to consider that Fe has a relatively large ionic radius, meaning that actual catalytic distances may be shorter than they appear, potentially influencing the reaction efficiency at these sites. These findings suggest that while spatial proximity plays a role, thermodynamic and kinetic factors significantly contribute to the preferential demethylation of C39 and C16 over C27.

DFT Hydroxylation Data

Hydroxylaytion Site	MH ---> M· + ·H<br>ΔG_hydrogen-abstraction (kcal/mol)	M· + · OH --> MOH<br>ΔG_hydroxylation (kcal/mol)	MH + ·OH ---> MOH + ·H<br>ΔG_sum (kcal/mol)
11-OH R	108.81	-95.48	13.33
11-OH S		-97.20	11.61
12-OH R	113.50	-95.18	18.32
12-OH S		-102.51	10.98
13-OH R	111.11	-102.66	8.46
13-OH S		-106.36	4.76
14-OH R	73.89	-66.08	7.81
14-OH S		-71.59	2.29
15-OH R	108.46	-98.88	9.58
15-OH S		-92.38	16.08
16-OH R	82.63	-63.94	18.69
16-OH S		-69.56	13.07
18-OH	102.59	-94.76	7.83
19-OH	109.25	-101.03	8.23
20-OH	111.62	-108.19	3.42
21-OH	100.40	-94.00	6.40
22-OH	113.11	-101.94	11.17
23-OH R	70.91	-53.78	17.13
23-OH S		-55.10	15.81
24-OH R	109.63	-91.41	18.22
24-OH S		-88.86	20.76
25-OH R	85.81	-65.55	20.27
25-OH S		-68.28	17.54
45-OH	116.00	-104.86	11.14
46-OH	113.25	-106.66	6.59
49-OH	106.73	-91.26	15.47

Calculated energy of a hydrogen abstraction at various carbon centers, energy resulting from hydroxylation , and overall reaction Gibbs free energy (ΔG).
